# Cardiac stress T1-mapping response and extracellular volume stability of MOLLI-based T1-mapping methods

**DOI:** 10.1038/s41598-021-92923-4

**Published:** 2021-06-30

**Authors:** Matthew K. Burrage, Mayooran Shanmuganathan, Qiang Zhang, Evan Hann, Iulia A. Popescu, Rajkumar Soundarajan, Kelvin Chow, Stefan Neubauer, Vanessa M. Ferreira, Stefan K. Piechnik

**Affiliations:** 1 University of Oxford Centre for Clinical Magnetic Resonance Research (OCMR), Division of Cardiovascular Medicine, Radcliffe Department of Medicine, University of Oxford, John Radcliffe Hospital, Level 0, Oxford, OX3 9DU UK; 2 Cardiovascular MR R&D, Siemens Medical Solutions USA, Inc., Chicago, IL USA

**Keywords:** Cardiology, Medical research

## Abstract

Stress and rest T1-mapping may assess for myocardial ischemia and extracellular volume (ECV). However, the stress T1 response is method-dependent, and underestimation may lead to misdiagnosis. Further, ECV quantification may be affected by time, as well as the number and dosage of gadolinium (Gd) contrast administered. We compared two commonly available T1-mapping approaches in their stress T1 response and ECV measurement stability. Healthy subjects (n = 10, 50% female, 35 ± 8 years) underwent regadenoson stress CMR (1.5 T) on two separate days. Prototype ShMOLLI 5(1)1(1)1 sequence was used to acquire consecutive mid-ventricular T1-maps at rest, stress and post-Gd contrast to track the T1 time evolution. For comparison, standard MOLLI sequences were used: MOLLI 5(3)3 Low (256 matrix) & High (192 matrix) Heart Rate (HR) to acquire rest and stress T1-maps, and MOLLI 4(1)3(1)2 Low & High HR for post-contrast T1-maps. Stress and rest myocardial blood flow (MBF) maps were acquired after IV Gd contrast (0.05 mmol/kg each). Stress T1 reactivity (delta T1) was defined as the relative percentage increase in native T1 between rest and stress. Myocardial T1 values for delta T1 (dT1) and ECV were calculated. Residuals from the identified time dependencies were used to assess intra-method variability. ShMOLLI achieved a greater stress T1 response compared to MOLLI Low and High HR (peak dT1 = 6.4 ± 1.7% vs. 4.8 ± 1.3% vs. 3.8 ± 1.0%, respectively; both p < 0.0001). ShMOLLI dT1 correlated strongly with stress MBF (r = 0.77, p < 0.001), compared to MOLLI Low HR (r = 0.65, p < 0.01) and MOLLI High HR (r = 0.43, p = 0.07). ShMOLLI ECV was more stable to gadolinium dose with less time drift (0.006–0.04% per minute) than MOLLI variants. Overall, ShMOLLI demonstrated less intra-individual variability than MOLLI variants for stress T1 and ECV quantification. Power calculations indicate up to a fourfold (stress T1) and 7.5-fold (ECV) advantage in sample-size reduction using ShMOLLI. Our results indicate that ShMOLLI correlates strongly with increased MBF during regadenoson stress and achieves a significantly higher stress T1 response, greater effect size, and greater ECV measurement stability compared with the MOLLI variants tested.

## Introduction

Quantitative T1-mapping allows in-vivo myocardial tissue characterization in detecting a variety of cardiovascular diseases^1–7^. Native (pre-contrast) T1 values are prolonged by increased tissue free water content, and are dependent on blood T1 and myocardial blood volume (MBV) via partial volume effects^8^. Changes in MBV from coronary vasodilatation during stress may thus be detectable by cardiovascular magnetic resonance (CMR) T1-mapping^9^. Several studies have used the change in myocardial T1 during stress (T1 reactivity) to differentiate ischemic, infarcted, remote, and normal myocardium in patients with obstructive coronary artery disease (CAD)^10–15^, and impaired vasodilatory reserve in diseases with non-obstructive CAD^16,17^. Stress T1-mapping holds potential for translation into clinical applications as a non-invasive method for assessing coronary vasoreactivity.

Several approaches for measuring myocardial T1 have been described^8^. Despite excellent T1 precision, the original modified Look-Locker inversion recovery (MOLLI) 3(3)3(3)5 sequence^18^ required a 17-heart-beat breath-hold for acquisition and was limited by heart-rate sensitivity. Sequence selection is an important consideration for stress T1-mapping applications as clinical patients with cardiopulmonary disease may not tolerate such long breath-holds during stress. Newer sequences, such as ShMOLLI 5(1)1(1)1^19^ and MOLLI 5(3)3^20^, allow shorter breath-holds and are widely used in clinical practice. ShMOLLI uses a 9 heart-beat breath-hold for acquisition, is largely heart-rate independent due to its in-built conditional reconstruction algorithm, and is potentially a ‘one-stop-shop’ T1-mapping sequence for native, stress and post-contrast T1-mapping^19,21^. More recent front-loaded MOLLI 5(3)3 improves on but does not eliminate earlier recognized heart-rate sensitivity, which may explain lower stress T1 responses^9^. As MOLLI 5(3)3 is suboptimal for short T1 ranges, separate variants, such as MOLLI 2(2)2(2)4 or MOLLI 4(1)3(1)2, have been designed for post-contrast acquisitions^20,22^. Independently of the chosen T1-mapping method, drifts in post-contrast T1 and, to a lesser degree, in extracellular volume (ECV) over time have been reported^23–25^.

Underestimation of stress T1 reactivity due to method-dependent heart-rate sensitivity, along with related impacts on measuring post-contrast T1 and ECV after vasodilator stress, has potentially important clinical implications. There have been no head-to-head studies to determine the optimal stress T1-mapping method and thus pave the way for larger scale studies. The current study therefore sought to determine the relationship between the two most widely available short breath-hold T1-mapping methods in normal physiology and has two main aims: 1) to directly compare the relationship between ShMOLLI and MOLLI in terms of their stress T1 response, variability, and effect size; and 2) compare their response with regards to ECV measurement stability over time.

## Methods

### Study population

Eleven healthy volunteers with no use of cardiovascular medication, no history of cardiovascular or systemic disease, no cardiovascular risk factors, and no previous history of cardiovascular symptoms were prospectively recruited. All subjects abstained from caffeine for 24 hours before undergoing CMR examination. Blood hematocrit testing was performed immediately before each CMR study.

### Image acquisition

**Figure 1:**
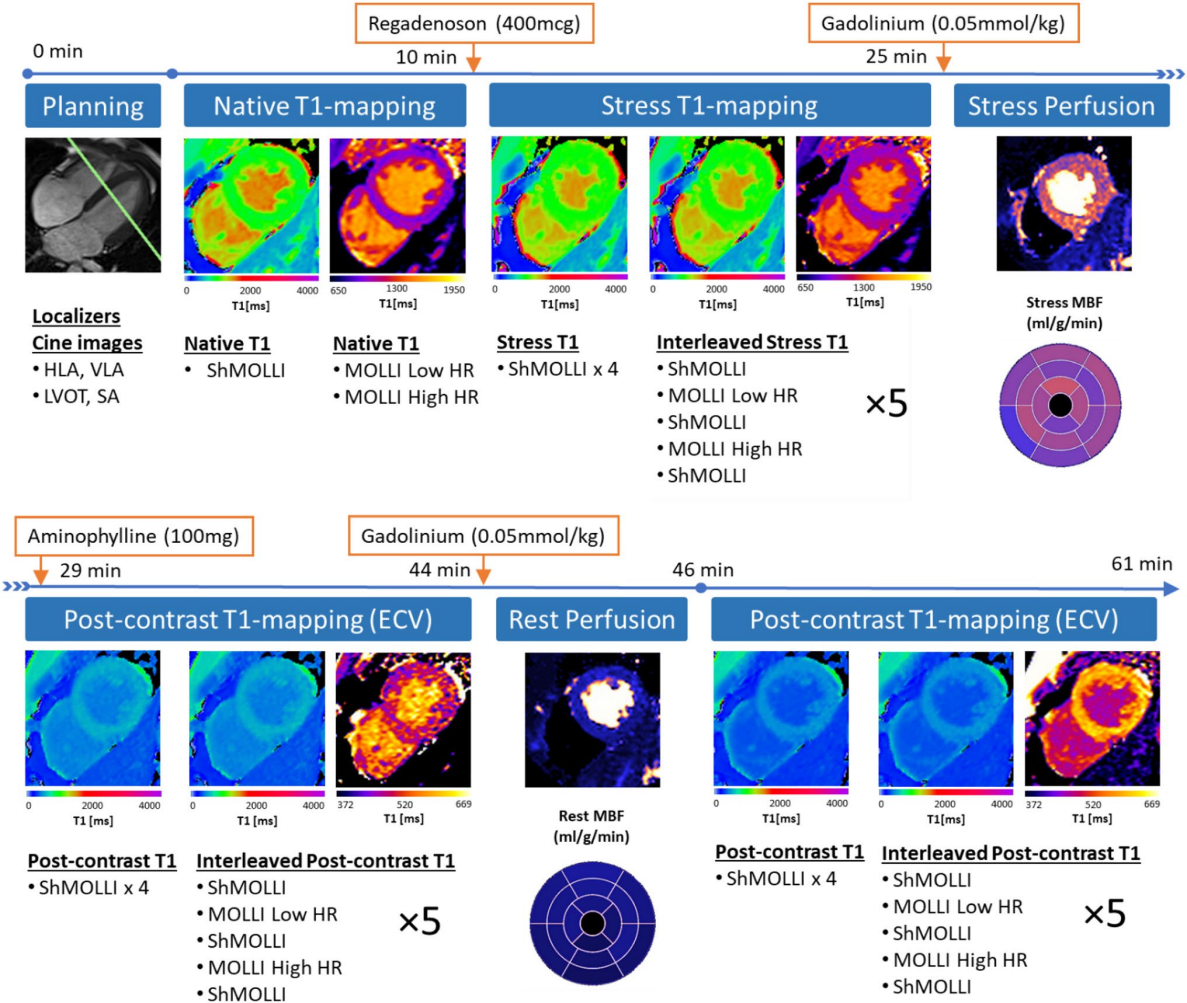
CMR scanning protocol for assessing T1 time dependencies. After planning, native T1-mapping is performed at rest using ShMOLLI, MOLLI Low HR, and MOLLI High HR. Stress T1-mapping is performed following administration of regadenoson (IV 400 mcg). This consists of four ShMOLLI measurements to cover the initial transition period and peak stress, followed by blocks of interleaved ShMOLLI and MOLLI variants. First-pass perfusion imaging is performed after gadolinium injection (0.05 mmol/kg). Allowing 4 min for the reconstruction of MBF data, stress is reversed with aminophylline (IV 100 mg) followed immediately by the same pattern of ShMOLLI and MOLLI acquisitions. Rest perfusion is performed after a second dose of gadolinium (0.05 mmol/kg), followed immediately by T1-mapping.

CMR imaging was performed using a 1.5-Tesla MRI scanner (AvantoFit, software version VE11C, Siemens Healthcare, Erlangen, Germany) using an 18-channel phased-array coil with the participant supine. Figure 1 shows a timeline of the CMR scanning protocol, with further detail provided in the Online supplement. Cine imaging was performed in three long-axis views and in short-axis slices covering the entire left ventricle (LV), using retrospectively ECG-gated balanced steady-state free precession (bSSFP) imaging ^26,27^. T1-maps were acquired based on the ShMOLLI sequence as previously published^19^, prototype version WIP1048. Quality assessment of ShMOLLI T1-maps using parametric goodness-of-fit (R^2^) maps were available in-line at time of acquisition^19,28^, based on open source reconstruction^29^. MOLLI (MyoMaps, Siemens Healthcare, Erlangen, Germany) T1-maps were acquired from vendor-provided product protocols: “Long T1” MOLLI 5(3)3 was used for rest and stress native T1-mapping and “Short T1” MOLLI 4(1)3(1)2 was used for post-contrast T1-mapping. Both sampling scheme protocols had “Low HR” (256 matrix) and “High HR” (192 matrix) variants, where the latter is recommended for heart rates greater than 80 bpm to reduce cardiac motion blurring.

The main study protocol (Fig. 1) included ninety-two T1-maps (60 ShMOLLI, 32 MOLLI) per participant from a single mid-ventricular slice. Systolic imaging (trigger delay (TD) of zero (0) ms) was used for T1-mapping sequences to standardize measurements and reduce the risk of mistriggering during tachycardia^30^. With exception of the initial rapid transition periods, stress and post-contrast T1-maps were acquired using repeated “blocks” alternating five T1-mapping sequences to allow interpolation of T1 measurements for direct comparison between the T1-mapping techniques under dynamic conditions.

Stress T1-maps were acquired consecutively, commencing immediately upon intravenous (IV) injection of regadenoson (Rapiscan, GE Healthcare AS, Oslo, Norway; 400 mcg over 5–10 s followed by a 10 ml 0.9% sodium chloride saline flush over 10 s). Heart rate and blood pressure were recorded throughout. First-pass perfusion imaging was performed after completion of the stress T1-mapping protocol (~ 15 min after injection of regadenoson) with an IV bolus injection of gadolinium (Gd) contrast (0.05 mmol/kg, gadoterate meglumine, Dotarem, Guerbet SA, Paris, France) followed by a 15–20 ml saline flush, both administered at 4-6 ml/sec. Pixel-wise perfusion maps were generated automatically using inline perfusion mapping software as previously described, to allow quantitative assessment of myocardial blood flow (MBF)^31^.

After a four minute delay (to allow for online reconstruction of pixel-wise perfusion maps), pharmacological stress was reversed (aminophylline 100 mg IV over 5–10 s followed by a 10 ml saline flush). Post-contrast T1-mapping was then performed with the same alternating T1-mapping sequence blocks (Fig. 1) immediately after injection of aminophylline. Rest first-pass perfusion imaging was then performed as described above. The post-contrast T1-mapping protocol was then repeated to allow assessment of the prolonged effects of time and a second dose of gadolinium on post-contrast T1 values. For simplicity, the time epochs following the first dose of gadolinium are referred to as “half-dose Gd” (i.e. 0.05 mmol/kg) while those after the second dose are referred to as “whole-dose Gd” (i.e. 0.1 mmol/kg).

**Figure 2:**
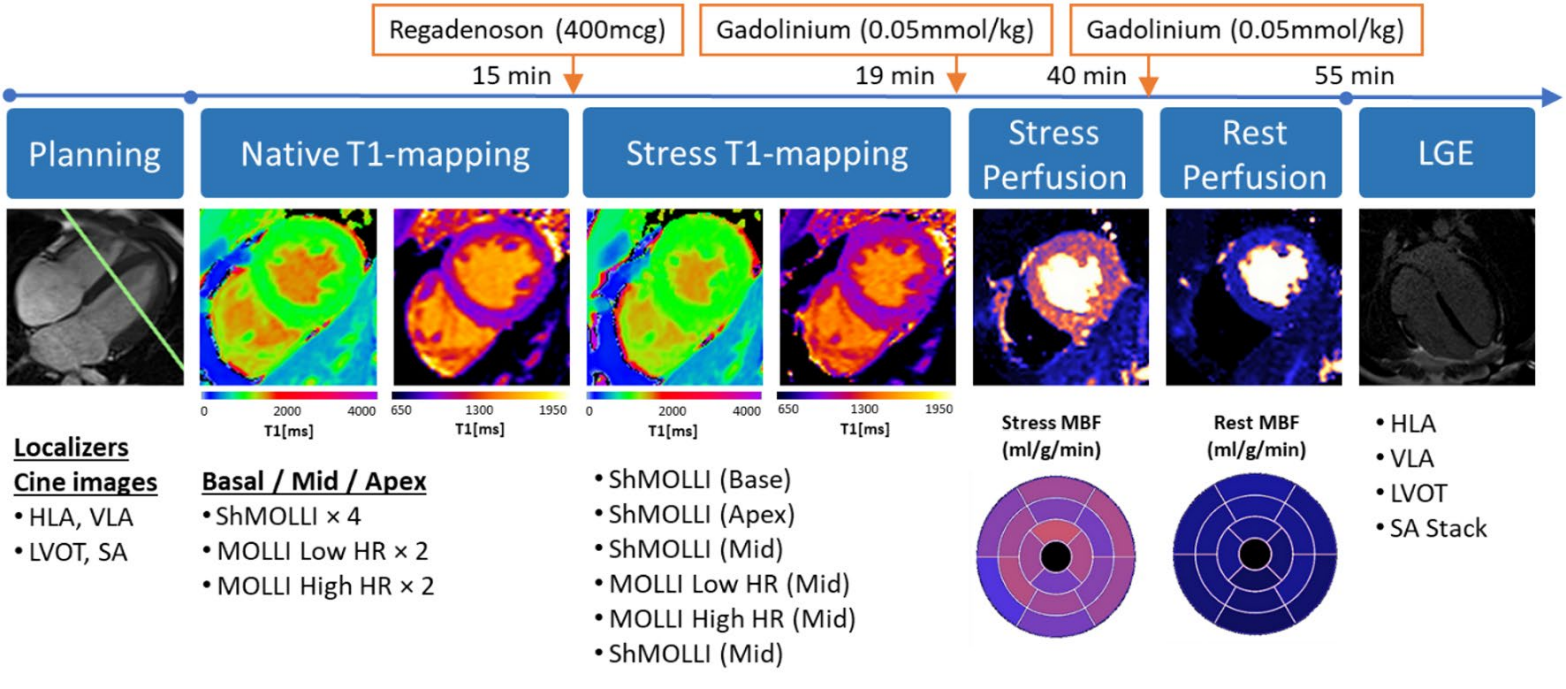
Modified second CMR scanning protocol for assessing stress T1 and MBF. Native T1-mapping is performed at rest using ShMOLLI, MOLLI Low HR, and MOLLI High HR in basal, mid-ventricular, and apical slice positions. Stress T1-mapping is performed following administration of regadenoson (IV 400 mcg) in the basal, apical, and mid-ventricular slices as illustrated. Stress is reversed with aminophylline (IV 100 mg) at ~ 25 min. Rest perfusion and late gadolinium enhancement images are acquired.

All participants underwent a second (Fig. 2) regadenoson stress CMR (median interval 7 days), which provided additional stress T1 and MBF values (analysis performed only for mid-ventricular slices) for comparison at a time point closer to peak stress (within 1.5–4 min of regadenoson administration). Late gadolinium enhancement (LGE) imaging was performed in long- and short-axis views to exclude myocardial infarction or other types of scarring, as per SCMR guidelines^26,27^.

### Image analysis

Image analysis for biventricular indices was performed offline in accordance with SCMR guidelines^26^, using cmr42 post-processing software (version 5.10.1, Circle Cardiovascular Imaging Inc., Calgary, Canada). Offline post-processing of T1-maps involved endocardial and epicardial contouring using MC-ROI (dedicated inhouse software developed by SKP in Interactive Data Language v6.1, Exelis Visual Information Solutions, Boulder, Colorado, USA). Automated contours were placed^32,33^ and manually checked for errors and corrected in compliance with internal training standards^34^. All image analysis was performed by a single experienced operator (MKB, 3 years CMR experience). T1-mapping results were based on quantitative analysis of all T1-maps, with global slice average T1 values of all myocardial segments per slice. The ECV fraction was calculated as previously described^27^. Stress and rest MBF were recorded as the mid-ventricular slice average for the respective perfusion sequences, acquired from inline perfusion mapping software^31^.

### Data modelling and statistical analysis

To enable direct comparison of the different T1-mapping sequences for the dynamic stress response, ShMOLLI T1 values were linearly interpolated for each MOLLI time point.

Relative stress responses were calculated for heart rate, MBF, and T1, as shown for stress T1 reactivity (dT1) on per-slice basis below:

$$d{\text{T}}1 = \frac{{stress~{\text{T}}1 - rest~{\text{T}}1}}{{rest~{\text{T}}1}}{\text{*}}100{\text{\% }}$$

The relationships between ShMOLLI and MOLLI variants for stress T1, dT1, post-contrast T1, and ECV were assessed with Pearson’s linear correlation coefficient (r), as well as the slope and intercept of the regression lines. A mono-exponential decay model was fitted to all subjects combined, starting after 1.5 min post-regadenoson stress for ShMOLLI dT1, being the method of choice with most samples acquired. Given the dynamic nature of this stress study, datapoints were grouped in 1-min intervals so as to better present the effects of time on T1 evolution. Independent fitting of MOLLI data was found suboptimal due to narrower dynamic range, and thus only the scale was fitted to match, in keeping with presumption that ShMOLLI and MOLLI follow the same decay pattern. Data modelling was performed offline after image analysis.

Residual errors from linear and non-linear modelling were calculated from regression equations as the difference between the predicted values and individual means. To correct for inter-subject variability, individual linear (ECV) and non-linear (dT1) models were fitted. Residual errors were pooled and analyzed, based on the standard deviation of the residuals (root mean square error; RMSE), to assess intra-individual measurement variance between T1-mapping methods. Intergroup differences were assessed using ANOVA with post-hoc testing as appropriate. Given non-normal distribution of residuals, statistical differences in variability between T1-mapping methods were assessed using Levene’s test. Paired tests were used whenever possible. P < 0.05 is considered statistically significant. Statistical analysis and data modelling were performed using R Studio (RStudio Team (2018). RStudio: Integrated Development for R. RStudio, Inc., Boston, MA).

### Ethics approval

Participation was voluntary and all participants signed a written informed consent. The study received ethical approval from the South Central-Oxford A Research Ethics Committee (13/SC/0376) and was performed in accordance with relevant guidelines and regulations.

## Results

### Study population

All participants completed the study protocol and tolerated regadenoson without complications. One participant was excluded after CMR detection of a large, incidental myocardial infarction on LGE imaging, leaving a total of ten healthy participants (50% female; mean age 35 ± 8 years) included in the study. All 10 healthy participants had structurally normal hearts, with normal resting cardiac volumes (LVEDVi 79 ± 15 ml/m^2^) and systolic function (LVEF 60 ± 3%) and no LGE. One participant did not undergo first-pass perfusion and MBF quantification due to a sequence malfunction on the day of study visit.

### Effect of regadenoson stress on heart rate (HR) and MBF

**Figure 3:**
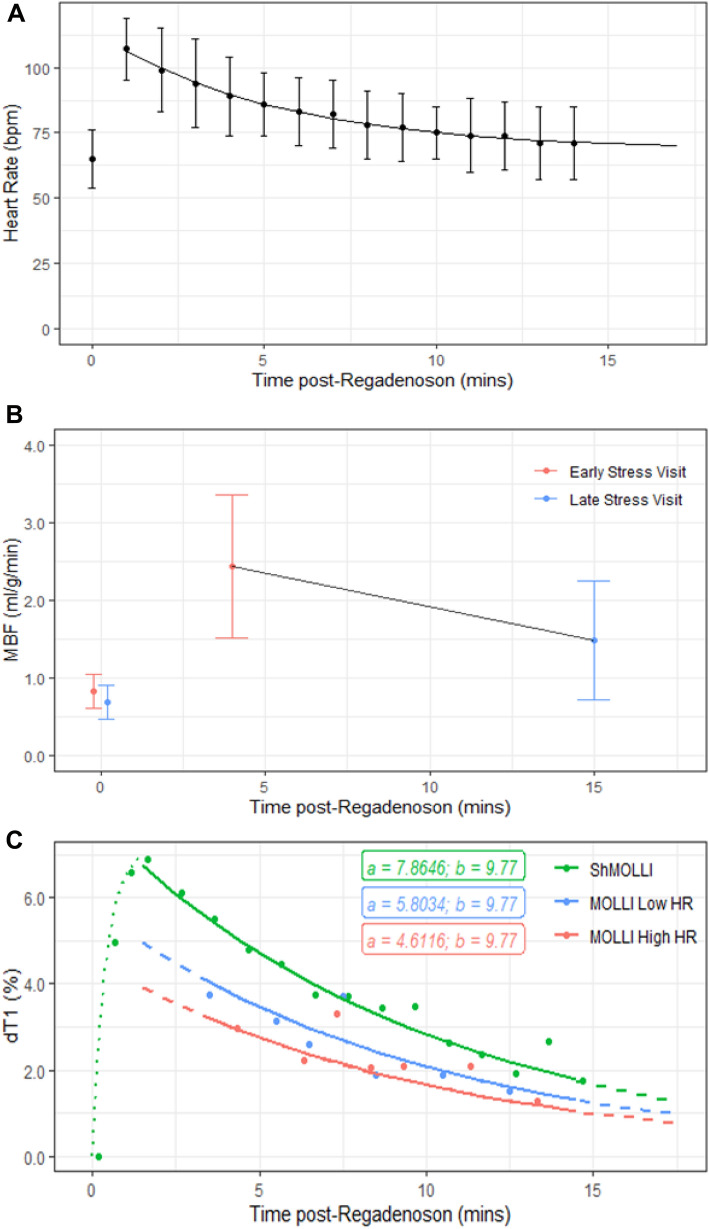
Regadenoson stress effects on heart rate (**A**), MBF (**B**) and dT1 (**C**) with overlaid mono-exponential decay models $y=a*exp(-x/b)$*.* In panel (**B**), time point 0 corresponds to the baseline conditions and is included to illustrate MBF at rest. In panel (**C**) the decay time b has been established for ShMOLLI only, and only amplitudes scaled to best fit other MOLLI variants; the dashed lines mark the extrapolation of the MOLLI models beyond the respective data ranges. The rising part of the T1 response to stress (dotted green line), HR and MBF responses (fitted to relative changes and transposed) are to guide the eye only.

Clear physiological effects of regadenoson were seen on all variables of interest (Fig. 3). Heart rate increased from baseline 66 ± 13 bpm to 107 ± 12 bpm (p < 0.0001) recorded first after 1 min of regadenoson injection. MBF was significantly elevated when measured at 4 min (stress MBF = 2.43 ± 0.92 ml/g/min compared to rest MBF = 0.83 ± 0.22 ml/g/min; p < 0.0001) and at 15 min (stress MBF = 1.48 ± 0.76 ml/g/min; rest MBF = 0.69 ± 0.22 ml/g/min; p < 0.01) post-regadenoson. Stress MBF at 4 min was significantly greater than stress MBF at 15 min (p < 0.0001).

### Regadenoson stress T1 response between ShMOLLI and MOLLI variants

**Figure 4:**
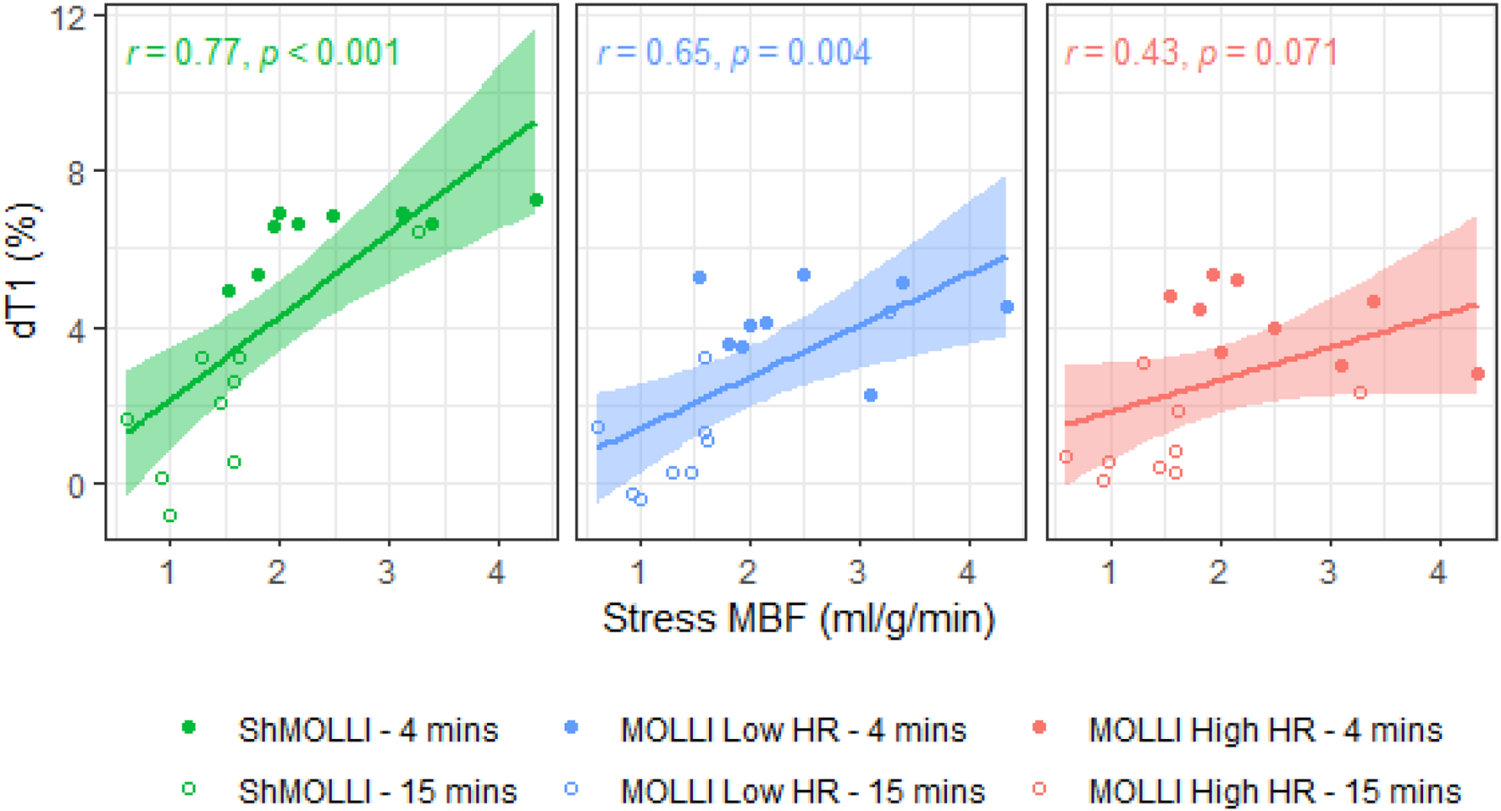
Correlation between stress MBF and dT1 during regadenoson stress. Stress MBF measured at ~ 4 (early stress) and ~ 15 min (late stress) after regadenoson in the same individuals on two separate days. Comparisons between dT1 and stress MBF are made for measured dT1 at the time point closest to MBF (all stress T1 data points measured within 90 s prior to MBF acquisition). Confidence intervals for the linear regressions are included (shaded ranges).

After regadenoson injection, ShMOLLI peak dT1 rose an average of 6.4 ± 1.7% within 30–120 s (response amplitude derived from averages of sample points 2–5; see Fig. 3C). This was significantly higher than extrapolated peak dT1 values for MOLLI Low HR (4.8 ± 1.3%; p < 0.0001) and MOLLI High HR (3.8 ± 1.0%; p < 0.0001). ShMOLLI dT1 also demonstrated the greatest slope and a strong linear correlation with stress MBF (Fig. 4) for equivalent time points (r = 0.77, p < 0.001) compared to MOLLI Low HR (r = 0.65, p < 0.01) and MOLLI High HR (r = 0.43, p = 0.07). Rest and stress native T1 values, along with post-contrast T1 values, are presented in Table 1.

**Table 1:** Differences in T1 measurements between methods for rest, stress, and post-contrast T1.

Variant	Rest T1 (ms) (mean ± SD)	Peak Stress T1 (ms) (mean ± SD)	Half-Dose Gd T1 (ms) (mean ± SD)	Whole-Dose Gd T1 (ms) (mean ± SD)
ShMOLLI	921 ± 19	979 ± 28	678 ± 41	556 ± 48
MOLLI Low HR	1001 ± 23*	1049 ± 23*^$^	723 ± 42*	602 ± 53*
MOLLI High HR	990 ± 23*^†^	1027 ± 24*^‡$^	724 ± 39*^†^	611 ± 48*^†^

* = P < 0.001 compared with ShMOLLI. † = P > 0.05 compared with MOLLI Low HR. ‡ = P < 0.001 compared with MOLLI Low HR. ^$^Stress T1 data for MOLLI Low and High HR from extrapolated model-predicted dT1. Post-contrast T1 reflects the average T1 across each epoch, with statistical comparisons performed at N = 44 samples (based on the maximum number of MOLLI samples per epoch) to assure fair inter-method comparison.

### ECV measurement stability after dual gadolinium dose administration

**Figure 5:**
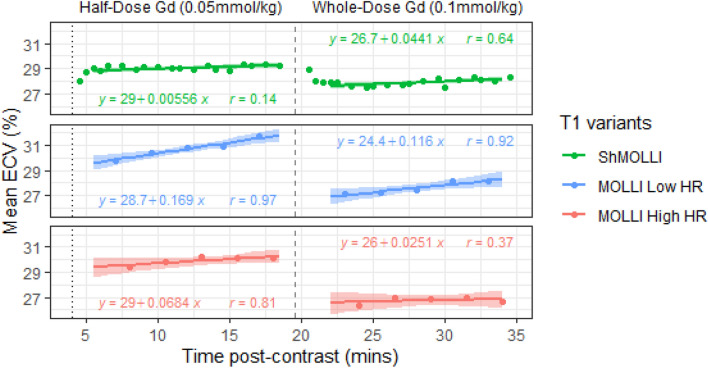
Effect of time and gadolinium dose on ECV. Linear regressions are shown for half-dose and whole-dose Gd epochs. Stress was reversed at 4 min (dotted vertical line); Gd was given at 0 min and a second dose at 19 min (dashed vertical line). The early rapid variation in ECV estimates after Gd seen in the ShMOLLI panel (data points outside the ranges marked by regression models) were excluded from analysis to allow fair inter-method comparisons. ShMOLLI ECV demonstrates little change in ECV with time, whereas MOLLI ECV (particularly MOLLI Low HR) is subject to linear time drift. Confidence intervals for the linear regressions are included (shaded ranges).

Following the first “half-dose” Gd-contrast injection (0.05 mmol/kg), ShMOLLI ECV rapidly achieved a steady-state equilibrium and remained stable over ~ 20 min, while MOLLI measurements showed clear positive trends in ECV over time, particularly for the Low HR variant (Fig. 5). Over the first Gd-contrast epoch, ShMOLLI ECV demonstrated little change with time (ECV trend = 0.006% per minute) and was significantly more stable over time than ECV measured with both MOLLI Low HR (0.17% per minute; p < 0.0001) and MOLLI High HR (0.07% per minute; p < 0.001). Over the second “whole-dose” Gd-contrast epoch (another 0.05 mmol/kg), the ShMOLLI ECV trend was again significantly more stable than MOLLI Low HR (0.04% per minute vs 0.12% per minute; p < 0.001) but was not different compared with MOLLI High HR (0.04% per minute vs 0.03% per minute; p = 0.18).

Furthermore, there were significant gadolinium dose dependencies for MOLLI ECV, with both Low HR and High HR variants yielding ECV 3.01–3.11% lower with whole-dose compared to half-dose Gd-contrast (p < 0.001; Table 2). ShMOLLI ECV was less sensitive to the second Gd dose, with a drop in ECV of ~ 1.2% at trend level significance (p = 0.06).

**Table 2:** Effect of gadolinium dose on ECV.

Variant	Half-Dose Gd (mean ± SD)	Whole-Dose Gd (mean ± SD)	P value^§^
ShMOLLI	29.10 ± 2.96	27.93 ± 2.79	0.06
MOLLI Low HR	30.84 ± 3.12*	27.83 ± 2.72^†^	< 0.001
MOLLI High HR	30.00 ± 3.34^†‡^	26.89 ± 2.93^†‡^	< 0.001

* = P < 0.05 compared with ShMOLLI. † = P > 0.05 compared with ShMOLLI. ‡ = P > 0.05 compared with MOLLI Low HR. ^§^ = all P values reported at N = 44 samples (based on the maximum number of MOLLI samples per epoch) to assure fair inter-method comparison. ECV calculated as the cohort average per epoch.

### Relationship between T1 measured by ShMOLLI and MOLLI variants

**Figure 6:**
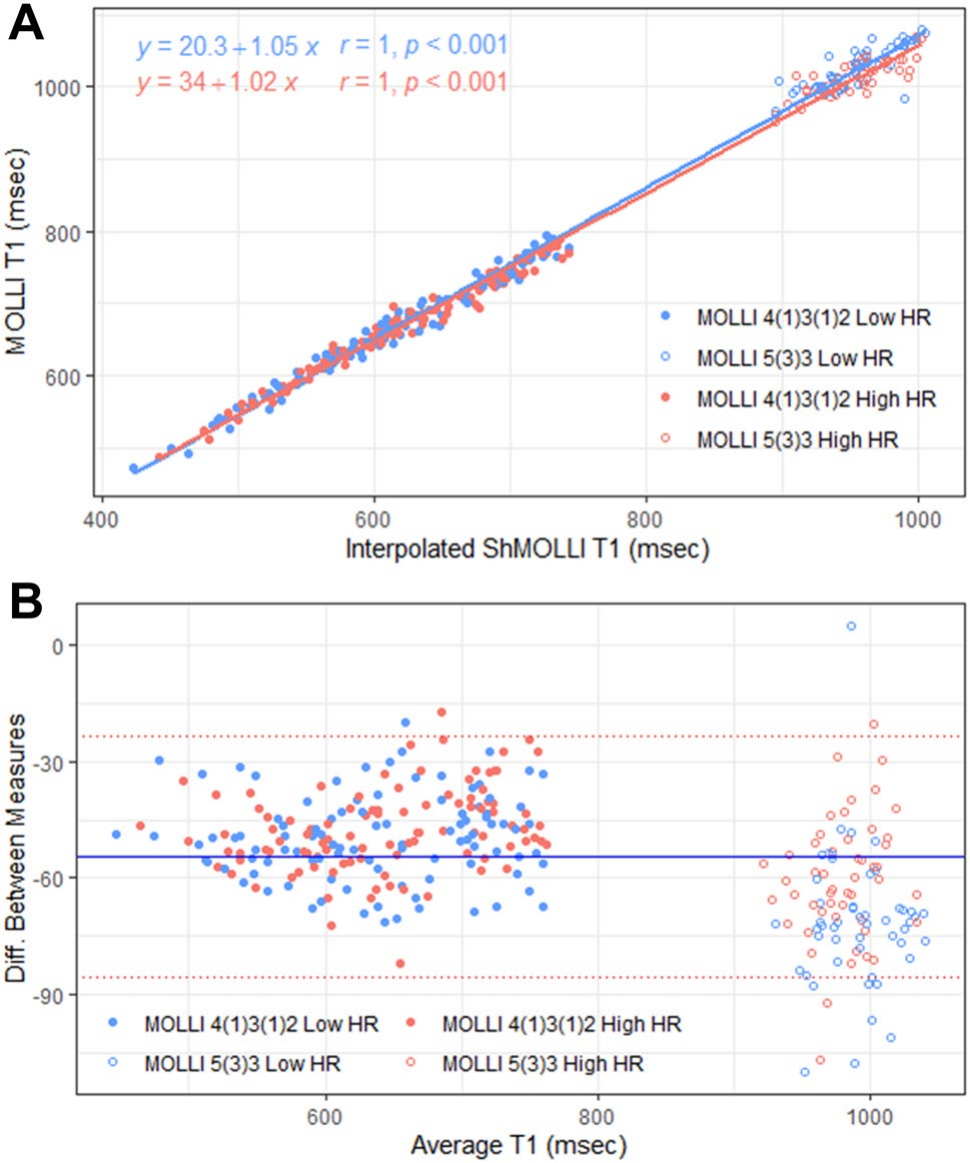
Relationship between T1-mapping variants. Linear fit for T1-mapping variants on native rest, stress, and post-contrast T1 (A) and analysis of differences plot comparing the results from MOLLI variants against all interpolated ShMOLLI values (B). The blue solid line (B) indicates the mean difference between ShMOLLI and MOLLI. The red dotted lines (B) indicate the 95% limit of agreement for all pooled MOLLI compared to ShMOLLI. Post-contrast T1 values are measured using the MOLLI 4(1)3(1)2 sequence with Low HR and High HR sub-variants; stress T1 values are measured using the MOLLI 5(3)3 sequence and Low HR and High HR sub-variants.

There were very strong linear relationships between ShMOLLI and both MOLLI Low HR and MOLLI High HR (both *r* > 0.99, p < 0.00001), across native (pre-contrast) stress and post-contrast T1 values (Fig. 6A). However, analysis of differences (Fig. 6B) showed obvious departures from a single linear relationship depending on the T1 range (particularly for the long T1 range during stress) between the methods.

**Figure 7:**
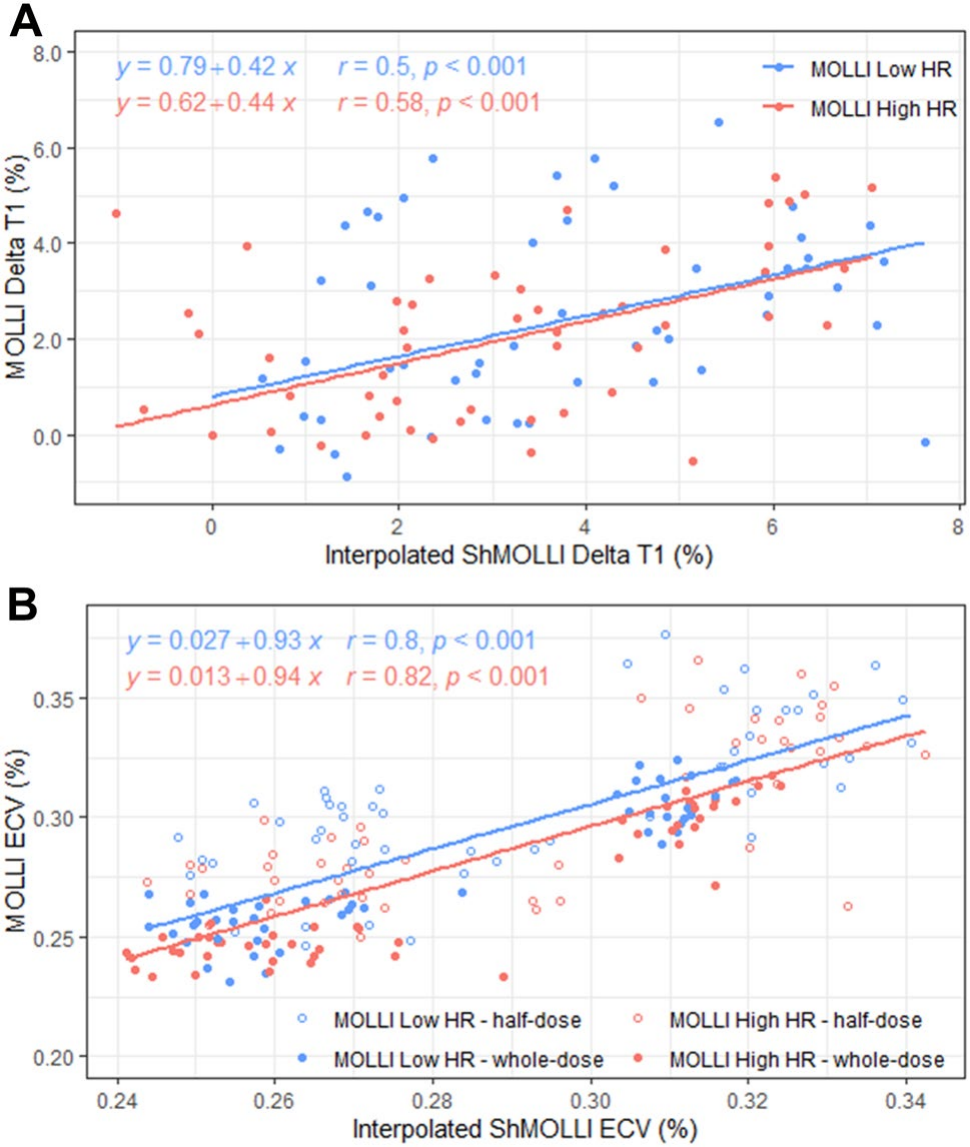
Impacts of MOLLI choice on estimation of stress delta T1 and ECV. Only modest correlations between methods are seen for MOLLI Low HR (r = 0.5) and MOLLI High HR (r = 0.58) respectively for delta T1 (**A**). Relationships are stronger for ECV for MOLLI Low HR (r = 0.8) and MOLLI High HR (r = 0.81) with distributions also shown for half-dose and whole-dose gadolinium (**B**). MOLLI variant samples correspond to time equivalent interpolated ShMOLLI.

Stress responses (dT1) for MOLLI Low HR and MOLLI High HR were modestly correlated with those measured by ShMOLLI (r = 0.5–0.58, Fig. 7A). Relationships for ECV between the T1 methods were stronger (r = 0.8–0.81), with nearly identical slopes for MOLLI Low HR and MOLLI High HR, but intercepts were offset by 1.5% between the variants (Fig. 7B). Similar trends were observed for ECV stratified by half-dose (0.05 mmol/kg) and whole-dose (0.1 mmol/kg) gadolinium concentrations (Fig. 7B, regression lines not shown for clarity).

### Intra-individual and intra-method variability between T1-mapping variants

For stress T1-mapping, ShMOLLI showed significantly less intra-individual variability than MOLLI Low HR (p < 0.0001), with a lower coefficient of variance than MOLLI High HR, although not statistically significant (p = 0.24) at the numbers available (Table 3).

**Table 3:** Measures of intra-individual variability across different T1-mapping variants measured by RMSE[CV].

Variability	dT1 RMSE [CV]	ECV – depending on Gd Dose RMSE [CV]		
		Half-dose	Whole-dose	P value
ShMOLLI	1.30 [0.37]	0.35 [0.012]	0.22 [0.008]	< 0.001
MOLLI Low HR	1.92 [0.74]*	0.79 [0.026]*	0.61 [0.022]*	< 0.001
MOLLI High HR	1.44 [0.65]^†^	0.46 [0.016]^†‡^	0.34 [0.013]*^‡^	< 0.01

* = P < 0.05 compared with ShMOLLI. † = P > 0.05 compared with ShMOLLI. ‡ = P < 0.05 compared with MOLLI Low HR. RMSE = Root Mean Square Error and refers to the standard deviation of the residuals. CV = Coefficient of Variance (RMSE/mean).

For ECV estimation at half-dose Gd concentration, ShMOLLI ECV was significantly less variable than MOLLI Low HR (P < 0.0001). ShMOLLI ECV also showed significantly less intra-individual variability than both MOLLI Low HR (p < 0.0001) and MOLLI High HR (p < 0.05) at the whole-dose Gd concentration. There was significantly less variability within individuals at the whole-dose Gd concentration compared to half-dose (p < 0.01) for all T1-mapping variants (Table 3).

## Discussion

This is the first systematic study to characterize regadenoson stress T1 dynamics over time, and to directly compare the relationship and variability between MOLLI variants for stress and post-contrast T1-mapping. We demonstrate that: (1) the regadenoson effect can be measured by stress T1-mapping, paving the way to clinical applications similar to those demonstrated previously with adenosine^10,13,35,36^; (2) ShMOLLI stress T1-mapping correlates strongly with MBF and achieves a greater overall stress T1 response compared to the MOLLI variants tested; and (3) ShMOLLI ECV can be robustly estimated at lower Gd doses and a wide range of times, and quickly achieves a post-Gd steady-state equilibrium with greater measurement stability over time and less variability compared with MOLLI variants.

### Dynamic response of T1-mapping during regadenoson stress

Our study demonstrated a stress T1 dynamic profile consistent with known regadenoson pharmacokinetics, with peak effects typically seen within 30–120 s followed by a long terminal elimination phase. This is similar to results from Lieu et al., with invasive coronary blood flow velocity measurements increasing by > 2.5-fold (3.1 ± 0.52) fold at regadenoson 400 mcg) for a sustained duration of 2.3 min (comparable to the response induced by gold-standard intracoronary adenosine), and accompanied by a rise in heart rate^37^. In contrast, an abolished stress T1 response may be seen in areas of pathology. We have recently shown that the normal regadenoson stress T1 response can be characterized across separate myocardial slices and individual myocardial segments (compared to the global T1 values reported in the current study), with sufficient signal-to-noise ratio to differentiate between infarcted, ischaemic, normal and remote myocardium in clinical patients, as validated by regions of interest co-localized to first-pass perfusion abnormalities and LGE images and corroborated against invasive coronary angiography^15^.

Stress T1 estimation is a crucial progress and has potential implications for contrast-free assessments of MBF and MBV. Nickander et al. recently demonstrated that increases in native myocardial T1 during vasodilatory stress correlate strongly with increases in MBV and MBF^38^. Although it is likely that change in MBV is more directly related to the underlying mechanism of T1 reactivity than change in MBF, we similarly observed strong linear correlations between ShMOLLI dT1 and MBF measured across two separate stress time points (~ 4 min and ~ 15 min), with a greater *r* than seen with the MOLLI variants. We saw lower stress MBF results in our healthy controls than reported elsewhere^39^. This likely reflects the timings of the first-pass perfusion acquisitions, which were not performed at peak stress in this study (i.e. acquired at 4 min and 15 min vs. typically 1–2 min in clinical practice) rather than the choice of vasodilator agent, given recent animal studies have shown no significant differences in MBF when comparing adenosine and regadenoson^40^. Our data supports that there are clear differences in stress T1 reactivity between the methods tested, with ShMOLLI demonstrating a significantly greater stress T1 response and effect size.

ShMOLLI and MOLLI variants may demonstrate different sensitivities to heart rate^20^, which may result in differences in dT1 values during stress T1-mapping protocols. Our head-to-head comparisons are broadly consistent with prior observations of lower stress T1 responses seen with MOLLI 5(3)3 (4.3 ± 2.8%^35^; 4.79 ± 3.14%^36^; 5.4 ± 2.4%^41^) compared with results achieved using ShMOLLI (6.2 ± 0.5%^10^; 7.1 ± 3.8%^13^; 6.4 ± 1.7% in the current study). The MOLLI 5s(3s)3s research prototype scheme with the guaranteed minimum duration of acquisition epochs using a sampling scheme measured in seconds (rather than in heartbeats)^20^ was previously shown to have similar adenosine reactivity as ShMOLLI (~ 6.2%, based on reported T1 values in^38^). However, the MOLLI 5s(3s)3s variant was not tested here due to its minimum breath holds of ~ 12–15 s, i.e. 2–3 times longer than any tested MOLLI variant in this study, which required ~ 5 s at observed peak HR > 100 bpm. The use of sequences with short breath hold requirements (as used in this study) is more practical for implementation of stress T1-mapping in the clinical setting and is likely to result in less cardiac motion artefacts compared with those dependent on longer patient breath holds^9,35^. Kuijpers et al.^35^ had reported substantial motion artefacts using MOLLI 5(3)3 for stress T1-mapping, and these are likely to worsen using MOLLI 5s(3s)3s due to even longer breath hold requirements.

### ECV quantification depends on gadolinium dosage, post-contrast time and T1 method

Post-contrast estimation of ECV relies on a steady-state Gd-equilibrium between the intravascular and interstitial compartments. We demonstrated that ShMOLLI ECV achieved a time-independent equilibrium early (within 7–10 min) following Gd administration, whereas MOLLI 4(1)3(1)2 variants (particularly MOLLI Low HR) yielded ECV that continued to increase over time. Schelbert et al. (0.6% rise in ECV over 30 min) and Kawel et al. (3% rise in ECV over ~ 40 min) also reported linear increases in ECV over time using MOLLI 3(3)5^23,24^. Weingartner et al. similarly demonstrated method dependencies in ECV estimation at 3 T, showing an absolute ~ 1.5% rise in MOLLI 4(1)3(1)2 ECV over a 10-min period (between 15–25 min after Gd), compared to much smaller changes using saturation recovery techniques (SAPPHIRE ~ 0.8%; SASHA ~ 0.6%)^25^. Further, our data demonstrated ECV dependencies on Gd dosage, with ShMOLLI ECV nearly threefold less affected than MOLLI ECV (1.2% vs. 3.01–3.11%). Caballeros et al., who recorded ECV 15 min after a first (0.1 mmol/kg) and then second (0.1 mmol/kg) dose of Gd, similarly showed that MOLLI 4(1)3(1)2 ECV dropped by 2.3 ± 1.1% with a second Gd-dose^42^.

Given that post-contrast T1 values in our study were generally > 600 ms during the half-dose Gd epoch, this may affect MOLLI 4(1)3(1)2 T1, being subject to increased underestimation error outside the validated application ranges^20^. However, as no gold standard T1 or ECV measurements were available, the underlying reason for the differences between ShMOLLI and MOLLI T1 changes over time cannot be concluded from this study alone. Larger case numbers, lower Gd-dosage and extended post-contrast observation times may be required to clarify the mechanism of the observed differences. Further research may be warranted, given that the ability to measure ECV at earlier time points and with lower Gd doses has potential benefits in reducing scanner time and costs.

### Pre-contrast, stress and post-contrast T1-mapping using different MOLLI variants

Despite strong correlations between ShMOLLI and MOLLI T1-mapping variants, the overall relationship is not strictly linear, with between-method differences and residuals showing trends which differ between MOLLI variants and for pre- and post-contrast and stress conditions. Given different biases between the different protocols (as evidenced by different rest T1 values), they should not be used interchangeably or combined at all. This adds a layer of further complexity above the known wide range of T1 offsets within the MOLLI family^43–45^.

### Intra-individual and intra-method variability between T1-mapping variants and their impact on power calculations for clinical studies

Noise and relative effect sizes are a critical factor in sample size estimation for clinical studies. Repeat variability, normalized to observed effects, is an adequate measure to compare power calculations between methods. For stress T1-mapping, we observed 1.75 to twofold greater relative differences in effect sizes for ShMOLLI compared to MOLLI. Assuming that severe pathology (e.g. ischemia due to obstructive CAD) leads to a near-abolished or nullified stress T1 response^10,35^, this would translate to an estimated reduction in case numbers of at least 3 to fourfold for the same study power when using ShMOLLI. We also demonstrated important differences in intra-method consistency across T1-mapping variants for ECV estimation. We saw greater relative differences in effect sizes of 1.3 to 2.2-fold (half-dose Gd epoch) and 1.6 to 2.75-fold (whole-dose Gd epoch) for ShMOLLI compared to MOLLI. Assuming the scale of pathological ECV effects to be in order of its absolute value, similar reasoning would lead to an expected 1.8 to 7.5-fold reduction in case numbers to achieve the same study power when using ShMOLLI.

## Limitations

This study was conducted in a small cohort of 10 healthy controls and excluded one silent major MI case, highlighting that small groups can suffer from significant sampling bias. One female participant in our cohort demonstrated a sustained stress response, with dT1 persisting near peak over the entire stress period. Although we did not specifically analyze sex differences, this may have contributed, with Nickander et al. recently showing higher stress MBV in females compared with males^46^. Bodyweight may also be a consideration (62 kg; BMI 23.9 in this case), given the one-size-fits-all regadenoson dosing protocol. Although stress T1-mapping typically has a relatively small effect size, and we were able to reliably measure the effect in all our healthy subjects, larger studies are needed to confirm the reliability of this technique in future.

Given the limited sampling period to about 2 measurements per minute, the findings are limited to a single mid-ventricular short-axis slice, which is sufficient for an initial proof of concept study on T1 time evolution. Due to different number of preparations and matrix-size, the choice of TD = 0 led to slightly different cardiac phases, i.e. trigger time (272.5 ms for ShMOLLI, 185 ms for MOLLI), depending on the variant. While there were no visual differences in image quality, this may need further investigation. As it is impossible to acquire different T1-mapping methods simultaneously, we relied on ShMOLLI to enable comparisons and establish relationships between methods. This was based on shorter breath-holds (lesser burden to subjects) and the excellent intra-individual, intra-scanner, and inter-scanner variability of ShMOLLI (< 2%)^21^. Repeating the experiments using MOLLI as the baseline modelling technique would not be a practical use of resources given the demonstrated noise ratios and power calculations. Given the vast cost and complexity of the protocols, the benefit of increasing subject numbers in further studies, preferably multi-site and multivendor, must be carefully weighed against consistency of evidence in published literature. Whilst multiple T1-mapping variants exist, for this study we chose to compare ShMOLLI with Siemens product MOLLI 5(3)3 variants, based on similarly short breath-holds for stress applications, more widespread sequence availability, and thus greater generalizability.

Further work is required to characterize the underlying mechanism for the observed differences in stress T1 response between the sequences tested. ShMOLLI and MOLLI are both known to be sensitive to a number of factors such as heart rate, T2, motion, inflow and magnetization transfer (MT) effects in a complex manner, which are difficult to control in an in-vivo study. For example, heart rate and motion are both increased during stress and differing sensitivities to these between ShMOLLI and MOLLI may contribute to their stress T1 response difference. Additionally, the MOLLI family of pulse sequences have a strong T2 dependence^47^, which may also influence the change in T1 with stress, particularly in the presence of an already elevated T2 as may be seen in some pathologies. However, as these confounders are intrinsic to the physiological stress response, their effects cannot be separated. Phantom experiments that can to some degree control for T1 and T2 effects may provide some insight into these mechanisms but are not expected to be able to fully simulate the complex interrelations between heart rate and stress-induced changes in T1, T2, T2*, and vascular volumes. Further study with comparison to a T1 mapping sequence less sensitive to these factors such as SASHA may also help to clarify the contribution of these confounders to the ShMOLLI and MOLLI stress T1 response.

## Conclusion

Like adenosine, regadenoson stress causes significant dynamically changing responses in myocardial T1 relaxation time measured using CMR. While myocardial T1 measurements by different techniques correlate strongly with each other, ECV and the stress T1 response show significant differences depending on the T1-mapping method, even within the related family of inversion recovery based bSSFP MOLLI variants. Our results indicate that ShMOLLI correlates strongly with increased MBF during regadenoson stress and achieves a significantly higher stress T1 response, greater effect size, and greater ECV measurement stability over time compared with the MOLLI variants tested, with critical differences in intra-individual variability and effect sizes suggesting potential significant impact on power calculations depending on the T1 method used.

## Supplementary Information

Supplementary Information 1.

Supplementary Information 2.

## Data Availability

The datasets used and/or analyzed during the current study are available from the corresponding author on reasonable request.

## Abbreviations

ANOVA: Analysis of variance
BMI: Body mass index
bSSFP: Balanced steady-state free precession
CAD: Coronary artery disease
CMR: Cardiovascular magnetic resonance
ECV: Extracellular volume
Gd: Gadolinium
HLA: Horizontal long axis
HR: Heart rate
IV: Intravenous
LGE: Late gadolinium enhancement
LV: Left ventricle/left ventricular
LVEDVi: Left ventricular end-diastolic volume index
LVEF: Left ventricular ejection fraction
LVOT: Left ventricular outflow tract
MOLLI: MOdified Look Locker Inversion recovery
MBF: Myocardial blood flow
MBV: Myocardial blood volume
MT: Magnetization transfer
RMSE: Root mean square error
SA: Short axis
SAPPHIRE: Saturation pulse prepared heart rate independent inversion recovery
SASHA: Saturation recovery single-shot acquisition
SCMR: Society for Cardiovascular Magnetic Resonance
ShMOLLI: Shortened modified Look Locker inversion recovery
TD: Trigger delay
VLA: Vertical long axis
