# Supplementary material for: Cardiac stress T1-mapping response and extracellular volume stability of MOLLI-based T1-mapping methods
Source: Sci Rep. 2021 Jun 30;11:13568. doi: 10.1038/s41598-021-92923-4 (PMC8245629; doi:10.1038/s41598-021-92923-4)
Supplement: Supplementary file 1 — Supplementary Information 1. [file 41598_2021_92923_MOESM1_ESM.pdf]

Table of contents

\\USER
Cardiac Research Protocols
Rapi-STRESS T1
Rapi-STRESS T1 (V2)
trufi_loc_multi_iPAT
trufi_loc_multi_iPAT@c
trufi_2-chamber_iPAT
trufi_4-chamber_iPAT
trufi_shortaxis_iPAT
HLA tf2d15_retro_iPAT3
VLA tf2d15_retro_iPAT3
LVOT tf2d15_retro_iPAT3
SA MID ONLY tf2d15_retro_iPAT3
T2Map_TrueFISP
TEST SSFP_Perf_MBF_MBV_aif_r3 (KELLMAN)
MidShMOLLI_192i_d11_nFilt (TD=0)
T1Map_LongT1 (TD=0)LowHR
ShMOLLI_192i_d11_nFilt (TD=0)
T1Map_LongT1_TD0_HighHR
ShMOLLI_192i_d11_nFilt (TD=0)
MidShMOLLI_192i_d11_nFilt (TD=0)
ShMOLLI_192i_d11_nFilt (TD=0)
ShMOLLI_192i_d11_nFilt (TD=0)
ShMOLLI_192i_d11_nFilt (TD=0)
MidShMOLLI_192i_d11_nFilt (TD=0)
T1Map_LongT1 (TD=0)LowHR
ShMOLLI_192i_d11_nFilt (TD=0)
T1Map_LongT1_TD0_HighHR
ShMOLLI_192i_d11_nFilt (TD=0)
MidShMOLLI_192i_d11_nFilt (TD=0)
T1Map_LongT1 (TD=0)LowHR
ShMOLLI_192i_d11_nFilt (TD=0)
T1Map_LongT1_TD0_HighHR
ShMOLLI_192i_d11_nFilt (TD=0)
MidShMOLLI_192i_d11_nFilt (TD=0)
T1Map_LongT1 (TD=0)LowHR
ShMOLLI_192i_d11_nFilt (TD=0)
T1Map_LongT1_TD0_HighHR
ShMOLLI_192i_d11_nFilt (TD=0)
MidShMOLLI_192i_d11_nFilt (TD=0)
T1Map_LongT1 (TD=0)LowHR
ShMOLLI_192i_d11_nFilt (TD=0)
T1Map_LongT1_TD0_HighHR
ShMOLLI_192i_d11_nFilt (TD=0)
MidShMOLLI_192i_d11_nFilt (TD=0)
T1Map_LongT1 (TD=0)LowHR
ShMOLLI_192i_d11_nFilt (TD=0)

				T1Map_LongT1_TD0_HighHR<br>ShMOLLI_192i_d11_nFilt (TD=0)<br>STRESS SSFP_Perf_MBF_MBV_aif_r3 (KELLMAN)<br>MidShMOLLI_192i_d11_nFilt (TD=0)<br>ShMOLLI_192i_d11_nFilt (TD=0)<br>ShMOLLI_192i_d11_nFilt (TD=0)<br>ShMOLLI_192i_d11_nFilt (TD=0)<br>MidShMOLLI_192i_d11_nFilt (TD=0)<br>T1Map_ShortT1(TD=0)lowHR<br>ShMOLLI_192i_d11_nFilt (TD=0)<br>T1Map_ShortT1(TD=0)highHR<br>ShMOLLI_192i_d11_nFilt (TD=0)<br>MidShMOLLI_192i_d11_nFilt (TD=0)<br>T1Map_ShortT1(TD=0)lowHR<br>ShMOLLI_192i_d11_nFilt (TD=0)<br>T1Map_ShortT1(TD=0)highHR<br>ShMOLLI_192i_d11_nFilt (TD=0)<br>MidShMOLLI_192i_d11_nFilt (TD=0)<br>T1Map_ShortT1(TD=0)lowHR<br>ShMOLLI_192i_d11_nFilt (TD=0)<br>T1Map_ShortT1(TD=0)highHR<br>ShMOLLI_192i_d11_nFilt (TD=0)<br>MidShMOLLI_192i_d11_nFilt (TD=0)<br>T1Map_ShortT1(TD=0)lowHR<br>ShMOLLI_192i_d11_nFilt (TD=0)<br>T1Map_ShortT1(TD=0)highHR<br>ShMOLLI_192i_d11_nFilt (TD=0)<br>MidShMOLLI_192i_d11_nFilt (TD=0)<br>T1Map_ShortT1(TD=0)lowHR<br>ShMOLLI_192i_d11_nFilt (TD=0)<br>T1Map_ShortT1(TD=0)highHR<br>ShMOLLI_192i_d11_nFilt (TD=0)<br>REST SSFP_Perf_MBF_MBV_aif_r3 (KELLMAN)<br>MidShMOLLI_192i_d11_nFilt (TD=0)<br>ShMOLLI_192i_d11_nFilt (TD=0)<br>ShMOLLI_192i_d11_nFilt (TD=0)<br>ShMOLLI_192i_d11_nFilt (TD=0)<br>MidShMOLLI_192i_d11_nFilt (TD=0)<br>T1Map_ShortT1(TD=0)lowHR<br>ShMOLLI_192i_d11_nFilt (TD=0)<br>T1Map_ShortT1(TD=0)highHR<br>ShMOLLI_192i_d11_nFilt (TD=0)<br>MidShMOLLI_192i_d11_nFilt (TD=0)<br>T1Map_ShortT1(TD=0)lowHR<br>ShMOLLI_192i_d11_nFilt (TD=0)<br>T1Map_ShortT1(TD=0)highHR<br>ShMOLLI_192i_d11_nFilt (TD=0)<br>MidShMOLLI_192i_d11_nFilt (TD=0)<br>T1Map_ShortT1(TD=0)lowHR<br>ShMOLLI_192i_d11_nFilt (TD=0)<br>T1Map_ShortT1(TD=0)highHR

				ShMOLLI_192i_d11_nFilt (TD=0)<br>MidShMOLLI_192i_d11_nFilt (TD=0)<br>T1Map_ShortT1(TD=0)lowHR<br>ShMOLLI_192i_d11_nFilt (TD=0)<br>T1Map_ShortT1(TD=0)highHR<br>ShMOLLI_192i_d11_nFilt (TD=0)<br>MidShMOLLI_192i_d11_nFilt (TD=0)<br>T1Map_ShortT1(TD=0)lowHR<br>ShMOLLI_192i_d11_nFilt (TD=0)<br>T1Map_ShortT1(TD=0)highHR<br>ShMOLLI_192i_d11_nFilt (TD=0)

\\USER\Cardiac Research Protocols\Rapi-STRESS T1\Rapi-STRESS T1 (V2)\trufi_loc_multi_iPAT

TA: 0:14 PM: REF Voxel size: 1.6×1.6×8.0 mmPAT: 2 Rel. SNR: 1.00 : tfi

Properties

Prio recon	Off
Load images to viewer	On
Inline movie	Off
Auto store images	On
Load images to stamp segments	Off
Load images to graphic segments	On
Auto open inline display	Off
Auto close inline display	Off
Start measurement without further preparation	On
Wait for user to start	Off
Start measurements	Single measurement

Routine

Slice group	1
Slices	3
Dist. factor	300 %
Position	L0.0 A30.0 H0.0 mm
Orientation	Transversal
Phase enc. dir.	A >> P
Slice group	2
Slices	3
Dist. factor	300 %
Position	L30.0 P0.0 H0.0 mm
Orientation	Sagittal
Phase enc. dir.	A >> P
Slice group	3
Slices	3
Dist. factor	300 %
Position	L0.0 P10.0 H0.0 mm
Orientation	Coronal
Phase enc. dir.	R >> L
AutoAlign	---
Phase oversampling	0 %
FoV read	400 mm
FoV phase	100.0 %
Slice thickness	8.0 mm
TR	288.36 ms
TE	1.14 ms
Averages	1
Concatenations	9
Filter	Distortion Corr.(2D), Prescan Normalize
Coil elements	BO1-3;SP1-3

Contrast - Common

TR	288.36 ms
TE	1.14 ms
TD	0 ms
Magn. preparation	None
Flip angle	80 deg
Fat suppr.	None
Wrap-up Magn.	Restore

Contrast - Dynamic

Averages	1
Averaging mode	Short term
Reconstruction	Magnitude
Measurements	1
Multiple series	Each measurement

Resolution - Common

FoV read	400 mm
FoV phase	100.0 %
Slice thickness	8.0 mm
Base resolution	256
Phase resolution	66 %
Phase partial Fourier	Off
Trajectory	Cartesian
Interpolation	Off

Resolution - iPAT

PAT mode	GRAPPA
Accel. factor PE	2
Ref. lines PE	24
Reference scan mode	Integrated

Resolution - Filter Image

Image Filter	Off
Distortion Corr.	On
Mode	2D
Unfiltered images	Off
Prescan Normalize	On
Unfiltered images	Off
Normalize	Off
B1 filter	Off

Resolution - Filter Rawdata

Raw filter	Off
Elliptical filter	Off
POCS	Off

Geometry - Common

Slice group	1
Slices	3
Dist. factor	300 %
Position	L0.0 A30.0 H0.0 mm
Orientation	Transversal
Phase enc. dir.	A >> P
Slice group	2
Slices	3
Dist. factor	300 %
Position	L30.0 P0.0 H0.0 mm
Orientation	Sagittal
Phase enc. dir.	A >> P
Slice group	3
Slices	3
Dist. factor	300 %
Position	L0.0 P10.0 H0.0 mm
Orientation	Coronal
Phase enc. dir.	R >> L
FoV read	400 mm
FoV phase	100.0 %
Slice thickness	8.0 mm
TR	288.36 ms
Multi-slice mode	Sequential
Series	Descending
Concatenations	9

Geometry - AutoAlign

Slice group	1
Position	L0.0 A30.0 H0.0 mm

Geometry - AutoAlign

Orientation	Transversal
Phase enc. dir.	A >> P
Slice group	2
Position	L30.0 P0.0 H0.0 mm
Orientation	Sagittal
Phase enc. dir.	A >> P
Slice group	3
Position	L0.0 P10.0 H0.0 mm
Orientation	Coronal
Phase enc. dir.	R >> L
AutoAlign	---
Initial Position	L0.0 A30.0 H0.0
Phase	-30.0 mm
Read	0.0 mm
Shift	0.0 mm
Initial Rotation	0.00 deg
Initial Orientation	Transversal

Geometry - Saturation

Fat suppr.	None
Wrap-up Magn.	Restore
Special sat.	None

Geometry - Navigator

System - Miscellaneous

Positioning mode	REF
Table position	H
Table position	0 mm
MSMA	S - C - T
Sagittal	R >> L
Coronal	A >> P
Transversal	F >> H
Coil Combine Mode	Adaptive Combine
Save uncombined	Off
Matrix Optimization	Off
Coil Focus	Flat
AutoAlign	---
Coil Select Mode	Default

System - Adjustments

B0 Shim mode	Tune up
Adjust with body coil	Off
Confirm freq. adjustment	Off
Assume Dominant Fat	Off
Assume Silicone	Off
Adjustment Tolerance	Auto

System - Adjust Volume

Position	Isocenter
Orientation	Transversal
Rotation	0.00 deg
A >> P	263 mm
R >> L	350 mm
F >> H	350 mm
Reset	Off

System - Tx/Rx

Frequency 1H	63.683250 MHz
Correction factor	1
Gain	High
Img. Scale Cor.	1.000
Reset	Off
? Ref. amplitude 1H	0.000 V

Physio - Signal1

1st Signal/Mode	ECG/Trigger
Average cycle	787 ± 129 ms
Average cycle	No Signal ms
Captured cycle	787 ± 129 ms
Acquisition window	800 ms
Trigger pulse	1
Trigger delay	400 ms
TR	288.36 ms
Concatenations	9
Segments	96
Phases	1
Adaptive Triggering	Off

Physio - Cardiac

Tagging	None
Magn. preparation	None
Fat suppr.	None
Dark blood	Off
FoV read	400 mm
FoV phase	100.0 %
Phase resolution	66 %
Cine	Off
Trajectory	Cartesian
Dummy heartbeats	0

Physio - PACE

Resp. control	Off
Concatenations	9

Inline - Common

Subtract	Off
Measurements	1
StdDev	Off
Save original images	On

Inline - Cardiac

Inline Evaluation	Off
Magn. preparation	None
Contrasts	1
TE	1.14 ms
TR	288.36 ms
Save original images	On

Inline - MIP

MIP-Sag	Off
MIP-Cor	Off
MIP-Tra	Off
MIP-Time	Off
Save original images	On

Inline - Composing

Inline Composing	Off
Distortion Corr.	On
Mode	2D
Unfiltered images	Off

Sequence - Part 1

Introduction	Off
Dimension	2D
Reordering	Linear
Asymmetric echo	Weak
Contrasts	1
Optimization	Min. TE

Sequence - Part 1

Multi-slice mode	Sequential
Echo spacing	2.6 ms
Sequence type	Trufi
Bandwidth	1149 Hz/Px

Sequence - Part 2

Define	Shots
Shots per slice	1
Segments	96
Trufi delta freq.	0 Hz
RF pulse type	Fast
Gradient mode	Fast
Excitation	Slice-sel.
Flip angle mode	Constant
Cine	Off

Sequence - Assistant

Mode	Min flip angle
Min flip angle	50 deg
Allowed delay	0 s

\\USER\Cardiac Research Protocols\Rapi-STRESS T1\Rapi-STRESS T1 (V2)\trufi_loc_multi_iPAT@c

TA: 0:14 PM: ISO Voxel size: 1.6×1.6×8.0 mmPAT: 2 Rel. SNR: 1.00 : tfi

Properties

Prio recon	Off
Load images to viewer	On
Inline movie	Off
Auto store images	On
Load images to stamp segments	On
Load images to graphic segments	On
Auto open inline display	Off
Auto close inline display	Off
Start measurement without further preparation	Off
Wait for user to start	Off
Start measurements	Single measurement

Routine

Slice group	1
Slices	3
Dist. factor	300 %
Position	L0.0 A30.0 H0.0 mm
Orientation	Transversal
Phase enc. dir.	A >> P
Slice group	2
Slices	3
Dist. factor	300 %
Position	L30.0 P0.0 H0.0 mm
Orientation	Sagittal
Phase enc. dir.	A >> P
Slice group	3
Slices	3
Dist. factor	300 %
Position	L0.0 P10.0 H0.0 mm
Orientation	Coronal
Phase enc. dir.	R >> L
AutoAlign	---
Phase oversampling	0 %
FoV read	400 mm
FoV phase	100.0 %
Slice thickness	8.0 mm
TR	288.36 ms
TE	1.14 ms
Averages	1
Concatenations	9
Filter	Distortion Corr.(2D), Prescan Normalize
Coil elements	BO1-3;SP1-3

Contrast - Common

TR	288.36 ms
TE	1.14 ms
TD	0 ms
Magn. preparation	None
Flip angle	80 deg
Fat suppr.	None
Wrap-up Magn.	Restore

Contrast - Dynamic

Averages	1
Averaging mode	Short term
Reconstruction	Magnitude
Measurements	1
Multiple series	Each measurement

Resolution - Common

FoV read	400 mm
FoV phase	100.0 %
Slice thickness	8.0 mm
Base resolution	256
Phase resolution	66 %
Phase partial Fourier	Off
Trajectory	Cartesian
Interpolation	Off

Resolution - iPAT

PAT mode	GRAPPA
Accel. factor PE	2
Ref. lines PE	24
Reference scan mode	Integrated

Resolution - Filter Image

Image Filter	Off
Distortion Corr.	On
Mode	2D
Unfiltered images	Off
Prescan Normalize	On
Unfiltered images	Off
Normalize	Off
B1 filter	Off

Resolution - Filter Rawdata

Raw filter	Off
Elliptical filter	Off
POCS	Off

Geometry - Common

Slice group	1
Slices	3
Dist. factor	300 %
Position	L0.0 A30.0 H0.0 mm
Orientation	Transversal
Phase enc. dir.	A >> P
Slice group	2
Slices	3
Dist. factor	300 %
Position	L30.0 P0.0 H0.0 mm
Orientation	Sagittal
Phase enc. dir.	A >> P
Slice group	3
Slices	3
Dist. factor	300 %
Position	L0.0 P10.0 H0.0 mm
Orientation	Coronal
Phase enc. dir.	R >> L
FoV read	400 mm
FoV phase	100.0 %
Slice thickness	8.0 mm
TR	288.36 ms
Multi-slice mode	Sequential
Series	Descending
Concatenations	9

Geometry - AutoAlign

Slice group	1
Position	L0.0 A30.0 H0.0 mm

Geometry - AutoAlign

Orientation	Transversal
Phase enc. dir.	A >> P
Slice group	2
Position	L30.0 P0.0 H0.0 mm
Orientation	Sagittal
Phase enc. dir.	A >> P
Slice group	3
Position	L0.0 P10.0 H0.0 mm
Orientation	Coronal
Phase enc. dir.	R >> L
AutoAlign	---
Initial Position	L0.0 A30.0 H0.0
Phase	-30.0 mm
Read	0.0 mm
Shift	0.0 mm
Initial Rotation	0.00 deg
Initial Orientation	Transversal

Geometry - Saturation

Fat suppr.	None
Wrap-up Magn.	Restore
Special sat.	None

Geometry - Navigator

System - Miscellaneous

Positioning mode	ISO
Table position	H
Table position	0 mm
MSMA	S - C - T
Sagittal	R >> L
Coronal	A >> P
Transversal	F >> H
Coil Combine Mode	Adaptive Combine
Save uncombined	Off
Matrix Optimization	Off
Coil Focus	Flat
AutoAlign	---
Coil Select Mode	Default

System - Adjustments

B0 Shim mode	Tune up
Adjust with body coil	Off
Confirm freq. adjustment	Off
Assume Dominant Fat	Off
Assume Silicone	Off
Adjustment Tolerance	Auto

System - Adjust Volume

Position	Isocenter
Orientation	Transversal
Rotation	0.00 deg
A >> P	263 mm
R >> L	350 mm
F >> H	350 mm
Reset	Off

System - Tx/Rx

Frequency 1H	63.683250 MHz
Correction factor	1
Gain	High
Img. Scale Cor.	1.000
Reset	Off
? Ref. amplitude 1H	0.000 V

Physio - Signal1

1st Signal/Mode	ECG/Trigger
Average cycle	787 ± 129 ms
Average cycle	No Signal ms
Captured cycle	787 ± 129 ms
Acquisition window	800 ms
Trigger pulse	1
Trigger delay	400 ms
TR	288.36 ms
Concatenations	9
Segments	96
Phases	1
Adaptive Triggering	Off

Physio - Cardiac

Tagging	None
Magn. preparation	None
Fat suppr.	None
Dark blood	Off
FoV read	400 mm
FoV phase	100.0 %
Phase resolution	66 %
Cine	Off
Trajectory	Cartesian
Dummy heartbeats	0

Physio - PACE

Resp. control	Off
Concatenations	9

Inline - Common

Subtract	Off
Measurements	1
StdDev	Off
Save original images	On

Inline - Cardiac

Inline Evaluation	Off
Magn. preparation	None
Contrasts	1
TE	1.14 ms
TR	288.36 ms
Save original images	On

Inline - MIP

MIP-Sag	Off
MIP-Cor	Off
MIP-Tra	Off
MIP-Time	Off
Save original images	On

Inline - Composing

Inline Composing	Off
Distortion Corr.	On
Mode	2D
Unfiltered images	Off

Sequence - Part 1

Introduction	Off
Dimension	2D
Reordering	Linear
Asymmetric echo	Weak
Contrasts	1
Optimization	Min. TE

Sequence - Part 1

Multi-slice mode	Sequential
Echo spacing	2.6 ms
Sequence type	Trufi
Bandwidth	1149 Hz/Px

Sequence - Part 2

Define	Shots
Shots per slice	1
Segments	96
Trufi delta freq.	0 Hz
RF pulse type	Fast
Gradient mode	Fast
Excitation	Slice-sel.
Flip angle mode	Constant
Cine	Off

Sequence - Assistant

Mode	Min flip angle
Min flip angle	50 deg
Allowed delay	0 s

\\USER\Cardiac Research Protocols\Rapi-STRESS T1\Rapi-STRESS T1 (V2)\trufi_2-chamber_iPAT
TA: 1.6 s PM: REF Voxel size: 1.5×1.5×8.0 mmPAT: 2 Rel. SNR: 1.00 : tfi

Properties

Prio recon	Off
Load images to viewer	On
Inline movie	Off
Auto store images	On
Load images to stamp segments	On
Load images to graphic segments	On
Auto open inline display	Off
Auto close inline display	Off
Start measurement without further preparation	Off
Wait for user to start	On
Start measurements	Single measurement

Routine

Slice group	1
Slices	1
Dist. factor	20 %
Position	R3.5 A30.0 H5.9 mm
Orientation	S > C-34.5
Phase enc. dir.	A >> P
AutoAlign	---
Phase oversampling	0 %
FoV read	380 mm
FoV phase	87.5 %
Slice thickness	8.0 mm
TR	260.22 ms
TE	1.16 ms
Averages	1
Concatenations	1
Filter	Distortion Corr.(2D), Prescan Normalize
Coil elements	BO1-3;SP1-3

Contrast - Common

TR	260.22 ms
TE	1.16 ms
Magn. preparation	None
Flip angle	80 deg
Fat suppr.	None
Wrap-up Magn.	Restore

Contrast - Dynamic

Averages	1
Averaging mode	Short term
Reconstruction	Magnitude
Measurements	1
Multiple series	Each measurement

Resolution - Common

FoV read	380 mm
FoV phase	87.5 %
Slice thickness	8.0 mm
Base resolution	256
Phase resolution	64 %
Phase partial Fourier	Off
Trajectory	Cartesian
Interpolation	Off

Resolution - iPAT

PAT mode	GRAPPA
Accel. factor PE	2
Ref. lines PE	24
Reference scan mode	Integrated

Resolution - Filter Image

Image Filter	Off
Distortion Corr.	On
Mode	2D
Unfiltered images	Off
Prescan Normalize	On
Unfiltered images	Off
Normalize	Off
B1 filter	Off

Resolution - Filter Rawdata

Raw filter	Off
Elliptical filter	Off
POCS	Off

Geometry - Common

Slice group	1
Slices	1
Dist. factor	20 %
Position	R3.5 A30.0 H5.9 mm
Orientation	S > C-34.5
Phase enc. dir.	A >> P
FoV read	380 mm
FoV phase	87.5 %
Slice thickness	8.0 mm
TR	260.22 ms
Multi-slice mode	Sequential
Series	Interleaved
Concatenations	1

Geometry - AutoAlign

Slice group	1
Position	R3.5 A30.0 H5.9 mm
Orientation	S > C-34.5
Phase enc. dir.	A >> P
AutoAlign	---
Initial Position	R3.5 A30.0 H5.9
Phase	-22.7 mm
Read	5.9 mm
Shift	-19.9 mm
Initial Rotation	0.00 deg
Initial Orientation	S > C
S > C	-34.5
> T	0.0

Geometry - Saturation

Fat suppr.	None
Wrap-up Magn.	Restore
Special sat.	None

Geometry - Navigator

System - Miscellaneous

Positioning mode	REF
Table position	H
Table position	0 mm

System - Miscellaneous

MSMA	S - C - T
Sagittal	R >> L
Coronal	A >> P
Transversal	F >> H
Coil Combine Mode	Adaptive Combine
Save uncombined	Off
Matrix Optimization	Off
Coil Focus	Flat
AutoAlign	---
Coil Select Mode	Default

System - Adjustments

B0 Shim mode	Tune up
Adjust with body coil	Off
Confirm freq. adjustment	Off
Assume Dominant Fat	Off
Assume Silicone	Off
Adjustment Tolerance	Auto

System - Adjust Volume

Position	Isocenter
Orientation	Transversal
Rotation	0.00 deg
A >> P	263 mm
R >> L	350 mm
F >> H	350 mm
Reset	Off

System - Tx/Rx

Frequency 1H	63.683250 MHz
Correction factor	1
Gain	High
Img. Scale Cor.	1.000
Reset	Off
? Ref. amplitude 1H	0.000 V

Physio - Signal1

1st Signal/Mode	ECG/Trigger
Average cycle	787 ± 129 ms
Average cycle	No Signal ms
Captured cycle	787 ± 129 ms
Acquisition window	800 ms
Trigger pulse	1
Trigger delay	400 ms
TR	260.22 ms
Concatenations	1
Segments	84
Phases	1
Adaptive Triggering	Off

Physio - Cardiac

Tagging	None
Magn. preparation	None
Fat suppr.	None
Dark blood	Off
FoV read	380 mm
FoV phase	87.5 %
Phase resolution	64 %
Cine	Off
Trajectory	Cartesian
Dummy heartbeats	0

Physio - PACE

Resp. control	Off
Concatenations	1

Inline - Common

Subtract	Off
Measurements	1
StdDev	Off
Save original images	On

Inline - Cardiac

Inline Evaluation	Off
Magn. preparation	None
Contrasts	1
TE	1.16 ms
TR	260.22 ms
Save original images	On

Inline - MIP

MIP-Sag	Off
MIP-Cor	Off
MIP-Tra	Off
MIP-Time	Off
Save original images	On

Inline - Composing

Inline Composing	Off
Distortion Corr.	On
Mode	2D
Unfiltered images	Off

Sequence - Part 1

Introduction	Off
Dimension	2D
Reordering	Linear
Asymmetric echo	Weak
Contrasts	1
Optimization	Min. TE
Multi-slice mode	Sequential
Echo spacing	2.7 ms
Sequence type	Trufi
Bandwidth	1149 Hz/Px

Sequence - Part 2

Define	Shots
Shots per slice	1
Segments	84
Trufi delta freq.	0 Hz
RF pulse type	Fast
Gradient mode	Fast
Excitation	Slice-sel.
Flip angle mode	Constant
Cine	Off

Sequence - Assistant

Mode	Min flip angle
Min flip angle	50 deg
Allowed delay	0 s

\\USER\Cardiac Research Protocols\Rapi-STRESS T1\Rapi-STRESS T1 (V2)\trufi_4-chamber_iPAT

TA: 1.6 s PM: REF Voxel size: 1.5×1.5×8.0 mmPAT: 2 Rel. SNR: 1.00 : tfi

Properties

Prio recon	Off
Load images to viewer	On
Inline movie	Off
Auto store images	On
Load images to stamp segments	On
Load images to graphic segments	On
Auto open inline display	Off
Auto close inline display	Off
Start measurement without further preparation	Off
Wait for user to start	Off
Start measurements	Single measurement

Routine

Slice group	1
Slices	1
Dist. factor	20 %
Position	L3.9 A36.0 H9.9 mm
Orientation	T > C35.8 > S-21.9
Phase enc. dir.	A >> P
AutoAlign	---
Phase oversampling	0 %
FoV read	380 mm
FoV phase	93.8 %
Slice thickness	8.0 mm
TR	275.34 ms
TE	1.16 ms
Averages	1
Concatenations	1
Filter	Distortion Corr.(2D), Prescan Normalize
Coil elements	BO1-3;SP1-3

Contrast - Common

TR	275.34 ms
TE	1.16 ms
Magn. preparation	None
Flip angle	80 deg
Fat suppr.	None
Wrap-up Magn.	Restore

Contrast - Dynamic

Averages	1
Averaging mode	Short term
Reconstruction	Magnitude
Measurements	1
Multiple series	Each measurement

Resolution - Common

FoV read	380 mm
FoV phase	93.8 %
Slice thickness	8.0 mm
Base resolution	256
Phase resolution	60 %
Phase partial Fourier	Off
Trajectory	Cartesian
Interpolation	Off

Resolution - iPAT

PAT mode	GRAPPA

Resolution - iPAT

Accel. factor PE	2
Ref. lines PE	24
Reference scan mode	Integrated

Resolution - Filter Image

Image Filter	Off
Distortion Corr.	On
Mode	2D
Unfiltered images	Off
Prescan Normalize	On
Unfiltered images	Off
Normalize	Off
B1 filter	Off

Resolution - Filter Rawdata

Raw filter	Off
Elliptical filter	Off
POCS	Off

Geometry - Common

Slice group	1
Slices	1
Dist. factor	20 %
Position	L3.9 A36.0 H9.9 mm
Orientation	T > C35.8 > S-21.9
Phase enc. dir.	A >> P
FoV read	380 mm
FoV phase	93.8 %
Slice thickness	8.0 mm
TR	275.34 ms
Multi-slice mode	Sequential
Series	Interleaved
Concatenations	1

Geometry - AutoAlign

Slice group	1
Position	L3.9 A36.0 H9.9 mm
Orientation	T > C35.8 > S-21.9
Phase enc. dir.	A >> P
AutoAlign	---
Initial Position	L3.9 A36.0 H9.9
Phase	-24.5 mm
Read	0.9 mm
Shift	28.5 mm
Initial Rotation	15.07 deg
Initial Orientation	T > C
T > C	35.8
> S	-21.9

Geometry - Saturation

Fat suppr.	None
Wrap-up Magn.	Restore
Special sat.	None

Geometry - Navigator

System - Miscellaneous

Positioning mode	REF
Table position	H
Table position	0 mm

System - Miscellaneous

MSMA	S - C - T
Sagittal	R >> L
Coronal	A >> P
Transversal	F >> H
Coil Combine Mode	Adaptive Combine
Save uncombined	Off
Matrix Optimization	Off
Coil Focus	Flat
AutoAlign	---
Coil Select Mode	Default

System - Adjustments

B0 Shim mode	Tune up
Adjust with body coil	Off
Confirm freq. adjustment	Off
Assume Dominant Fat	Off
Assume Silicone	Off
Adjustment Tolerance	Auto

System - Adjust Volume

Position	Isocenter
Orientation	Transversal
Rotation	0.00 deg
A >> P	263 mm
R >> L	350 mm
F >> H	350 mm
Reset	Off

System - Tx/Rx

Frequency 1H	63.683250 MHz
Correction factor	1
Gain	High
Img. Scale Cor.	1.000
Reset	Off
? Ref. amplitude 1H	0.000 V

Physio - Signal1

1st Signal/Mode	ECG/Trigger
Average cycle	787 ± 129 ms
Average cycle	No Signal ms
Captured cycle	787 ± 129 ms
Acquisition window	800 ms
Trigger pulse	1
Trigger delay	400 ms
TR	275.34 ms
Concatenations	1
Segments	84
Phases	1
Adaptive Triggering	Off

Physio - Cardiac

Tagging	None
Magn. preparation	None
Fat suppr.	None
Dark blood	Off
FoV read	380 mm
FoV phase	93.8 %
Phase resolution	60 %
Cine	Off
Trajectory	Cartesian
Dummy heartbeats	0

Physio - PACE

Resp. control	Off
Concatenations	1

Inline - Common

Subtract	Off
Measurements	1
StdDev	Off
Save original images	On

Inline - Cardiac

Inline Evaluation	Off
Magn. preparation	None
Contrasts	1
TE	1.16 ms
TR	275.34 ms
Save original images	On

Inline - MIP

MIP-Sag	Off
MIP-Cor	Off
MIP-Tra	Off
MIP-Time	Off
Save original images	On

Inline - Composing

Inline Composing	Off
Distortion Corr.	On
Mode	2D
Unfiltered images	Off

Sequence - Part 1

Introduction	Off
Dimension	2D
Reordering	Linear
Asymmetric echo	Weak
Contrasts	1
Optimization	Min. TE
Multi-slice mode	Sequential
Echo spacing	2.7 ms
Sequence type	Trufi
Bandwidth	1149 Hz/Px

Sequence - Part 2

Define	Shots
Shots per slice	1
Segments	84
Trufi delta freq.	0 Hz
RF pulse type	Fast
Gradient mode	Fast
Excitation	Slice-sel.
Flip angle mode	Constant
Cine	Off

Sequence - Assistant

Mode	Min flip angle
Min flip angle	50 deg
Allowed delay	0 s

\\USER\Cardiac Research Protocols\Rapi-STRESS T1\Rapi-STRESS T1 (V2)\trufi_shortaxis_iPAT

TA: 0:11 PM: REF Voxel size: 1.5×1.5×8.0 mmPAT: 2 Rel. SNR: 1.00 : tfi

Properties

Prio recon	Off
Load images to viewer	On
Inline movie	Off
Auto store images	On
Load images to stamp segments	On
Load images to graphic segments	On
Auto open inline display	Off
Auto close inline display	Off
Start measurement without further preparation	Off
Wait for user to start	Off
Start measurements	Single measurement

Routine

Slice group	1
Slices	7
Dist. factor	100 %
Position	L38.7 A33.2 F40.4 mm
Orientation	T > S43.5 > C-28.0
Phase enc. dir.	A >> P
AutoAlign	---
Phase oversampling	0 %
FoV read	380 mm
FoV phase	87.5 %
Slice thickness	8.0 mm
TR	275.34 ms
TE	1.16 ms
Averages	1
Concatenations	7
Filter	Distortion Corr.(2D), Prescan Normalize
Coil elements	BO1-3;SP1-3

Contrast - Common

TR	275.34 ms
TE	1.16 ms
TD	0 ms
Magn. preparation	None
Flip angle	80 deg
Fat suppr.	None
Wrap-up Magn.	Restore

Contrast - Dynamic

Averages	1
Averaging mode	Short term
Reconstruction	Magnitude
Measurements	1
Multiple series	Each measurement

Resolution - Common

FoV read	380 mm
FoV phase	87.5 %
Slice thickness	8.0 mm
Base resolution	256
Phase resolution	64 %
Phase partial Fourier	Off
Trajectory	Cartesian
Interpolation	Off

Resolution - iPAT

PAT mode	GRAPPA
Accel. factor PE	2
Ref. lines PE	24
Reference scan mode	Integrated

Resolution - Filter Image

Image Filter	Off
Distortion Corr.	On
Mode	2D
Unfiltered images	Off
Prescan Normalize	On
Unfiltered images	Off
Normalize	Off
B1 filter	Off

Resolution - Filter Rawdata

Raw filter	Off
Elliptical filter	Off
POCS	Off

Geometry - Common

Slice group	1
Slices	7
Dist. factor	100 %
Position	L38.7 A33.2 F40.4 mm
Orientation	T > S43.5 > C-28.0
Phase enc. dir.	A >> P
FoV read	380 mm
FoV phase	87.5 %
Slice thickness	8.0 mm
TR	275.34 ms
Multi-slice mode	Sequential
Series	Descending
Concatenations	7

Geometry - AutoAlign

Slice group	1
Position	L38.7 A33.2 F40.4 mm
Orientation	T > S43.5 > C-28.0
Phase enc. dir.	A >> P
AutoAlign	---
Initial Position	L38.7 A33.2 F40.4
Phase	-3.1 mm
Read	-0.3 mm
Shift	-65.0 mm
Initial Rotation	24.03 deg
Initial Orientation	T > S
T > S	43.5
> C	-28.0

Geometry - Saturation

Fat suppr.	None
Wrap-up Magn.	Restore
Special sat.	None

Geometry - Navigator

System - Miscellaneous

Positioning mode	REF
Table position	H

System - Miscellaneous

Table position	0 mm
MSMA	S - C - T
Sagittal	R >> L
Coronal	A >> P
Transversal	F >> H
Coil Combine Mode	Adaptive Combine
Save uncombined	Off
Matrix Optimization	Off
Coil Focus	Flat
AutoAlign	---
Coil Select Mode	Default

System - Adjustments

B0 Shim mode	Tune up
Adjust with body coil	Off
Confirm freq. adjustment	Off
Assume Dominant Fat	Off
Assume Silicone	Off
Adjustment Tolerance	Auto

System - Adjust Volume

Position	Isocenter
Orientation	Transversal
Rotation	0.00 deg
A >> P	263 mm
R >> L	350 mm
F >> H	350 mm
Reset	Off

System - Tx/Rx

Frequency 1H	63.683250 MHz
Correction factor	1
Gain	High
Img. Scale Cor.	1.000
Reset	Off
? Ref. amplitude 1H	0.000 V

Physio - Signal1

1st Signal/Mode	ECG/Trigger
Average cycle	787 ± 129 ms
Average cycle	No Signal ms
Captured cycle	787 ± 129 ms
Acquisition window	800 ms
Trigger pulse	1
Trigger delay	400 ms
TR	275.34 ms
Concatenations	7
Segments	84
Phases	1
Adaptive Triggering	Off

Physio - Cardiac

Tagging	None
Magn. preparation	None
Fat suppr.	None
Dark blood	Off
FoV read	380 mm
FoV phase	87.5 %
Phase resolution	64 %
Cine	Off
Trajectory	Cartesian
Dummy heartbeats	0

Physio - PACE

Resp. control	Off
Concatenations	7

Inline - Common

Subtract	Off
Measurements	1
StdDev	Off
Save original images	On

Inline - Cardiac

Inline Evaluation	Off
Magn. preparation	None
Contrasts	1
TE	1.16 ms
TR	275.34 ms
Save original images	On

Inline - MIP

MIP-Sag	Off
MIP-Cor	Off
MIP-Tra	Off
MIP-Time	Off
Save original images	On

Inline - Composing

Inline Composing	Off
Distortion Corr.	On
Mode	2D
Unfiltered images	Off

Sequence - Part 1

Introduction	Off
Dimension	2D
Reordering	Linear
Asymmetric echo	Weak
Contrasts	1
Optimization	Min. TE
Multi-slice mode	Sequential
Echo spacing	2.7 ms
Sequence type	Trufi
Bandwidth	1149 Hz/Px

Sequence - Part 2

Define	Shots
Shots per slice	1
Segments	84
Trufi delta freq.	0 Hz
RF pulse type	Fast
Gradient mode	Fast
Excitation	Slice-sel.
Flip angle mode	Constant
Cine	Off

Sequence - Assistant

Mode	Min flip angle
Min flip angle	50 deg
Allowed delay	0 s

\\USER\Cardiac Research Protocols\Rapi-STRESS T1\Rapi-STRESS T1 (V2)\HLA tf2d15_retro_iPAT3
TA: 4.7 s PM: REF Voxel size: 2.0×2.0×7.0 mmPAT: 3 Rel. SNR: 1.00 : tfi

Properties

Prio recon	Off
Load images to viewer	On
Inline movie	On
Auto store images	On
Load images to stamp segments	On
Load images to graphic segments	On
Auto open inline display	Off
Auto close inline display	Off
Start measurement without further preparation	Off
Wait for user to start	Off
Start measurements	Single measurement

Routine

Slice group	1
Slices	1
Dist. factor	43 %
Position	L12.2 A31.4 H10.6 mm
Orientation	T > C36.0 > S-5.8
Phase enc. dir.	A >> P
AutoAlign	---
Phase oversampling	0 %
FoV read	380 mm
FoV phase	100.0 %
Slice thickness	7.0 mm
TR	40.50 ms
TE	1.14 ms
Averages	1
Concatenations	1
Filter	Distortion Corr.(2D), Prescan Normalize, Image Filter
Coil elements	BO1-3;SP1-4

Contrast - Common

TR	40.50 ms
TE	1.14 ms
Magn. preparation	None
Flip angle	55 deg
Fat suppr.	None
Wrap-up Magn.	Restore

Contrast - Dynamic

Averages	1
Averaging mode	Short term
Reconstruction	Magnitude
Measurements	1
Multiple series	Each slice

Resolution - Common

FoV read	380 mm
FoV phase	100.0 %
Slice thickness	7.0 mm
Base resolution	192
Phase resolution	100 %
Phase partial Fourier	Off
Trajectory	Cartesian
View sharing	Off
Interpolation	Off

Resolution - iPAT

PAT mode	GRAPPA
Accel. factor PE	3
Ref. lines PE	24
Reference scan mode	GRE/separate

Resolution - Filter Image

Image Filter	On
! Intensity	Medium
Edge Enhancement	2
Smoothing	2
Unfiltered images	Off
Distortion Corr.	On
Mode	2D
Unfiltered images	Off
Prescan Normalize	On
Unfiltered images	Off
Normalize	Off
B1 filter	Off

Resolution - Filter Rawdata

Raw filter	Off
Elliptical filter	Off
POCS	Off

Geometry - Common

Slice group	1
Slices	1
Dist. factor	43 %
Position	L12.2 A31.4 H10.6 mm
Orientation	T > C36.0 > S-5.8
Phase enc. dir.	A >> P
FoV read	380 mm
FoV phase	100.0 %
Slice thickness	7.0 mm
TR	40.50 ms
Multi-slice mode	Sequential
Series	Interleaved
Concatenations	1

Geometry - AutoAlign

Slice group	1
Position	L12.2 A31.4 H10.6 mm
Orientation	T > C36.0 > S-5.8
Phase enc. dir.	A >> P
AutoAlign	---
Initial Position	L12.2 A31.4 H10.6
Phase	-18.4 mm
Read	-10.8 mm
Shift	28.1 mm
Initial Rotation	4.22 deg
Initial Orientation	T > C
T > C	36.0
> S	-5.8

Geometry - Saturation

Fat suppr.	None
Wrap-up Magn.	Restore
Special sat.	None

Geometry - Navigator

System - Miscellaneous

Positioning mode	REF
Table position	H
Table position	0 mm
MSMA	S - C - T
Sagittal	R >> L
Coronal	A >> P
Transversal	F >> H
Coil Combine Mode	Adaptive Combine
Save uncombined	Off
Matrix Optimization	Off
Coil Focus	Flat
AutoAlign	---
Coil Select Mode	Default

System - Adjustments

B0 Shim mode	Tune up
Adjust with body coil	On
Confirm freq. adjustment	Off
Assume Dominant Fat	Off
Assume Silicone	Off
Adjustment Tolerance	Auto

System - Adjust Volume

Position	Isocenter
Orientation	Transversal
Rotation	0.00 deg
A >> P	263 mm
R >> L	350 mm
F >> H	350 mm
Reset	Off

System - Tx/Rx

Frequency 1H	63.683250 MHz
Correction factor	1
Gain	High
Img. Scale Cor.	1.000
Reset	Off
? Ref. amplitude 1H	0.000 V

Physio - Signal1

1st Signal/Mode	ECG/Retro
Average cycle	787 ± 129 ms
Average cycle	No Signal ms
Calculated phases	25
TR	40.50 ms
Concatenations	1
Segments	15
Arrhythmia detection	None

Physio - Cardiac

Tagging	None
Magn. preparation	None
Fat suppr.	None
Dark blood	Off
FoV read	380 mm
FoV phase	100.0 %
Phase resolution	100 %
Cine	On
Trajectory	Cartesian
View sharing	Off
Dummy heartbeats	1

Physio - PACE

Resp. control	Off

Physio - PACE

Concatenations	1

Inline - Common

Subtract	Off
Measurements	1
StdDev	Off
Save original images	On

Inline - Cardiac

Inline Evaluation	Off
Magn. preparation	None
Contrasts	1
TE	1.14 ms
TR	40.50 ms
Save original images	On

Inline - MIP

MIP-Sag	Off
MIP-Cor	Off
MIP-Tra	Off
MIP-Time	Off
Save original images	On

Inline - Composing

Inline Composing	Off
Distortion Corr.	On
Mode	2D
Unfiltered images	Off

Sequence - Part 1

Introduction	Off
Dimension	2D
Reordering	Linear
Asymmetric echo	Weak
Contrasts	1
Optimization	Min. TE TR
Multi-slice mode	Sequential
Echo spacing	2.7 ms
Sequence type	Trufi
Bandwidth	930 Hz/Px

Sequence - Part 2

Define	Segments
Segments	15
Trufi delta freq.	0 Hz
RF pulse type	Fast
Gradient mode	Fast*
Excitation	Slice-sel.
Flip angle mode	Constant
Cine	On

Sequence - Assistant

Mode	Off
Allowed delay	0 s

\\USER\Cardiac Research Protocols\Rapi-STRESS T1\Rapi-STRESS T1 (V2)\VLA tf2d15_retro_iPAT3

TA: 4.7 s PM: REF Voxel size: 2.0×2.0×7.0 mmPAT: 3 Rel. SNR: 1.00 : tfi

Properties

Prio recon	Off
Load images to viewer	On
Inline movie	On
Auto store images	On
Load images to stamp segments	On
Load images to graphic segments	On
Auto open inline display	Off
Auto close inline display	Off
Start measurement without further preparation	Off
Wait for user to start	Off
Start measurements	Single measurement

Routine

Slice group	1
Slices	1
Dist. factor	43 %
Position	L12.2 A31.4 H10.6 mm
Orientation	T > C36.0 > S-5.8
Phase enc. dir.	A >> P
AutoAlign	---
Phase oversampling	0 %
FoV read	380 mm
FoV phase	100.0 %
Slice thickness	7.0 mm
TR	40.50 ms
TE	1.14 ms
Averages	1
Concatenations	1
Filter	Distortion Corr.(2D), Prescan Normalize, Image Filter
Coil elements	BO1-3;SP1-4

Contrast - Common

TR	40.50 ms
TE	1.14 ms
Magn. preparation	None
Flip angle	55 deg
Fat suppr.	None
Wrap-up Magn.	Restore

Contrast - Dynamic

Averages	1
Averaging mode	Short term
Reconstruction	Magnitude
Measurements	1
Multiple series	Each slice

Resolution - Common

FoV read	380 mm
FoV phase	100.0 %
Slice thickness	7.0 mm
Base resolution	192
Phase resolution	100 %
Phase partial Fourier	Off
Trajectory	Cartesian
View sharing	Off
Interpolation	Off

Resolution - iPAT

PAT mode	GRAPPA
Accel. factor PE	3
Ref. lines PE	24
Reference scan mode	GRE/separate

Resolution - Filter Image

Image Filter	On
! Intensity	Medium
Edge Enhancement	2
Smoothing	2
Unfiltered images	Off
Distortion Corr.	On
Mode	2D
Unfiltered images	Off
Prescan Normalize	On
Unfiltered images	Off
Normalize	Off
B1 filter	Off

Resolution - Filter Rawdata

Raw filter	Off
Elliptical filter	Off
POCS	Off

Geometry - Common

Slice group	1
Slices	1
Dist. factor	43 %
Position	L12.2 A31.4 H10.6 mm
Orientation	T > C36.0 > S-5.8
Phase enc. dir.	A >> P
FoV read	380 mm
FoV phase	100.0 %
Slice thickness	7.0 mm
TR	40.50 ms
Multi-slice mode	Sequential
Series	Interleaved
Concatenations	1

Geometry - AutoAlign

Slice group	1
Position	L12.2 A31.4 H10.6 mm
Orientation	T > C36.0 > S-5.8
Phase enc. dir.	A >> P
AutoAlign	---
Initial Position	L12.2 A31.4 H10.6
Phase	-18.4 mm
Read	-10.8 mm
Shift	28.1 mm
Initial Rotation	4.22 deg
Initial Orientation	T > C
T > C	36.0
> S	-5.8

Geometry - Saturation

Fat suppr.	None
Wrap-up Magn.	Restore
Special sat.	None

Geometry - Navigator

System - Miscellaneous

Positioning mode	REF
Table position	H
Table position	0 mm
MSMA	S - C - T
Sagittal	R >> L
Coronal	A >> P
Transversal	F >> H
Coil Combine Mode	Adaptive Combine
Save uncombined	Off
Matrix Optimization	Off
Coil Focus	Flat
AutoAlign	---
Coil Select Mode	Default

System - Adjustments

B0 Shim mode	Tune up
Adjust with body coil	On
Confirm freq. adjustment	Off
Assume Dominant Fat	Off
Assume Silicone	Off
Adjustment Tolerance	Auto

System - Adjust Volume

Position	Isocenter
Orientation	Transversal
Rotation	0.00 deg
A >> P	263 mm
R >> L	350 mm
F >> H	350 mm
Reset	Off

System - Tx/Rx

Frequency 1H	63.683250 MHz
Correction factor	1
Gain	High
Img. Scale Cor.	1.000
Reset	Off
? Ref. amplitude 1H	0.000 V

Physio - Signal1

1st Signal/Mode	ECG/Retro
Average cycle	787 ± 129 ms
Average cycle	No Signal ms
Calculated phases	25
TR	40.50 ms
Concatenations	1
Segments	15
Arrhythmia detection	None

Physio - Cardiac

Tagging	None
Magn. preparation	None
Fat suppr.	None
Dark blood	Off
FoV read	380 mm
FoV phase	100.0 %
Phase resolution	100 %
Cine	On
Trajectory	Cartesian
View sharing	Off
Dummy heartbeats	1

Physio - PACE

Resp. control	Off

Physio - PACE

Concatenations	1

Inline - Common

Subtract	Off
Measurements	1
StdDev	Off
Save original images	On

Inline - Cardiac

Inline Evaluation	Off
Magn. preparation	None
Contrasts	1
TE	1.14 ms
TR	40.50 ms
Save original images	On

Inline - MIP

MIP-Sag	Off
MIP-Cor	Off
MIP-Tra	Off
MIP-Time	Off
Save original images	On

Inline - Composing

Inline Composing	Off
Distortion Corr.	On
Mode	2D
Unfiltered images	Off

Sequence - Part 1

Introduction	Off
Dimension	2D
Reordering	Linear
Asymmetric echo	Weak
Contrasts	1
Optimization	Min. TE TR
Multi-slice mode	Sequential
Echo spacing	2.7 ms
Sequence type	Trufi
Bandwidth	930 Hz/Px

Sequence - Part 2

Define	Segments
Segments	15
Trufi delta freq.	0 Hz
RF pulse type	Fast
Gradient mode	Fast*
Excitation	Slice-sel.
Flip angle mode	Constant
Cine	On

Sequence - Assistant

Mode	Off
Allowed delay	0 s

\\USER\Cardiac Research Protocols\Rapi-STRESS T1\Rapi-STRESS T1 (V2)\LVOT tf2d15_retro_iPAT 3

TA: 4.7 s PM: REF Voxel size: 2.0×2.0×7.0 mmPAT: 3 Rel. SNR: 1.00 : tfi

Properties

Prio recon	Off
Load images to viewer	On
Inline movie	On
Auto store images	On
Load images to stamp segments	On
Load images to graphic segments	On
Auto open inline display	Off
Auto close inline display	Off
Start measurement without further preparation	Off
Wait for user to start	Off
Start measurements	Single measurement

Routine

Slice group	1
Slices	1
Dist. factor	43 %
Position	L12.2 A31.4 H10.6 mm
Orientation	T > C36.0 > S-5.8
Phase enc. dir.	A >> P
AutoAlign	---
Phase oversampling	0 %
FoV read	380 mm
FoV phase	100.0 %
Slice thickness	7.0 mm
TR	40.50 ms
TE	1.14 ms
Averages	1
Concatenations	1
Filter	Distortion Corr.(2D), Prescan Normalize, Image Filter
Coil elements	BO1-3;SP1-4

Contrast - Common

TR	40.50 ms
TE	1.14 ms
Magn. preparation	None
Flip angle	55 deg
Fat suppr.	None
Wrap-up Magn.	Restore

Contrast - Dynamic

Averages	1
Averaging mode	Short term
Reconstruction	Magnitude
Measurements	1
Multiple series	Each slice

Resolution - Common

FoV read	380 mm
FoV phase	100.0 %
Slice thickness	7.0 mm
Base resolution	192
Phase resolution	100 %
Phase partial Fourier	Off
Trajectory	Cartesian
View sharing	Off
Interpolation	Off

Resolution - iPAT

PAT mode	GRAPPA
Accel. factor PE	3
Ref. lines PE	24
Reference scan mode	GRE/separate

Resolution - Filter Image

Image Filter	On
! Intensity	Medium
Edge Enhancement	2
Smoothing	2
Unfiltered images	Off
Distortion Corr.	On
Mode	2D
Unfiltered images	Off
Prescan Normalize	On
Unfiltered images	Off
Normalize	Off
B1 filter	Off

Resolution - Filter Rawdata

Raw filter	Off
Elliptical filter	Off
POCS	Off

Geometry - Common

Slice group	1
Slices	1
Dist. factor	43 %
Position	L12.2 A31.4 H10.6 mm
Orientation	T > C36.0 > S-5.8
Phase enc. dir.	A >> P
FoV read	380 mm
FoV phase	100.0 %
Slice thickness	7.0 mm
TR	40.50 ms
Multi-slice mode	Sequential
Series	Interleaved
Concatenations	1

Geometry - AutoAlign

Slice group	1
Position	L12.2 A31.4 H10.6 mm
Orientation	T > C36.0 > S-5.8
Phase enc. dir.	A >> P
AutoAlign	---
Initial Position	L12.2 A31.4 H10.6
Phase	-18.4 mm
Read	-10.8 mm
Shift	28.1 mm
Initial Rotation	4.22 deg
Initial Orientation	T > C
T > C	36.0
> S	-5.8

Geometry - Saturation

Fat suppr.	None
Wrap-up Magn.	Restore
Special sat.	None

Geometry - Navigator

System - Miscellaneous

Positioning mode	REF
Table position	H
Table position	0 mm
MSMA	S - C - T
Sagittal	R >> L
Coronal	A >> P
Transversal	F >> H
Coil Combine Mode	Adaptive Combine
Save uncombined	Off
Matrix Optimization	Off
Coil Focus	Flat
AutoAlign	---
Coil Select Mode	Default

System - Adjustments

B0 Shim mode	Tune up
Adjust with body coil	On
Confirm freq. adjustment	Off
Assume Dominant Fat	Off
Assume Silicone	Off
Adjustment Tolerance	Auto

System - Adjust Volume

Position	Isocenter
Orientation	Transversal
Rotation	0.00 deg
A >> P	263 mm
R >> L	350 mm
F >> H	350 mm
Reset	Off

System - Tx/Rx

Frequency 1H	63.683250 MHz
Correction factor	1
Gain	High
Img. Scale Cor.	1.000
Reset	Off
? Ref. amplitude 1H	0.000 V

Physio - Signal1

1st Signal/Mode	ECG/Retro
Average cycle	787 ± 129 ms
Average cycle	No Signal ms
Calculated phases	25
TR	40.50 ms
Concatenations	1
Segments	15
Arrhythmia detection	None

Physio - Cardiac

Tagging	None
Magn. preparation	None
Fat suppr.	None
Dark blood	Off
FoV read	380 mm
FoV phase	100.0 %
Phase resolution	100 %
Cine	On
Trajectory	Cartesian
View sharing	Off
Dummy heartbeats	1

Physio - PACE

Resp. control	Off
Concatenations	1

Inline - Common

Subtract	Off
Measurements	1
StdDev	Off
Save original images	On

Inline - Cardiac

Inline Evaluation	Off
Magn. preparation	None
Contrasts	1
TE	1.14 ms
TR	40.50 ms
Save original images	On

Inline - MIP

MIP-Sag	Off
MIP-Cor	Off
MIP-Tra	Off
MIP-Time	Off
Save original images	On

Inline - Composing

Inline Composing	Off
Distortion Corr.	On
Mode	2D
Unfiltered images	Off

Sequence - Part 1

Introduction	Off
Dimension	2D
Reordering	Linear
Asymmetric echo	Weak
Contrasts	1
Optimization	Min. TE TR
Multi-slice mode	Sequential
Echo spacing	2.7 ms
Sequence type	Trufi
Bandwidth	930 Hz/Px

Sequence - Part 2

Define	Segments
Segments	15
Trufi delta freq.	0 Hz
RF pulse type	Fast
Gradient mode	Fast*
Excitation	Slice-sel.
Flip angle mode	Constant
Cine	On

Sequence - Assistant

Mode	Off
Allowed delay	0 s

\\USER\Cardiac Research Protocols\Rapi-STRESS T1\Rapi-STRESS T1 (V2)\SA MID ONLY tf2d15_retro_iPAT3

TA: 4.7 s PM: REF Voxel size: 2.0×2.0×7.0 mmPAT: 3 Rel. SNR: 1.00 : tfi

Properties

Prio recon	Off
Load images to viewer	On
Inline movie	On
Auto store images	On
Load images to stamp segments	On
Load images to graphic segments	On
Auto open inline display	Off
Auto close inline display	Off
Start measurement without further preparation	Off
Wait for user to start	Off
Start measurements	Single measurement

Routine

Slice group	1
Slices	1
Dist. factor	43 %
Position	L12.2 A31.4 H10.6 mm
Orientation	T > C36.0 > S-5.8
Phase enc. dir.	A >> P
AutoAlign	---
Phase oversampling	0 %
FoV read	380 mm
FoV phase	100.0 %
Slice thickness	7.0 mm
TR	40.50 ms
TE	1.14 ms
Averages	1
Concatenations	1
Filter	Distortion Corr.(2D), Prescan Normalize, Image Filter
Coil elements	BO1-3;SP1-4

Contrast - Common

TR	40.50 ms
TE	1.14 ms
Magn. preparation	None
Flip angle	55 deg
Fat suppr.	None
Wrap-up Magn.	Restore

Contrast - Dynamic

Averages	1
Averaging mode	Short term
Reconstruction	Magnitude
Measurements	1
Multiple series	Each slice

Resolution - Common

FoV read	380 mm
FoV phase	100.0 %
Slice thickness	7.0 mm
Base resolution	192
Phase resolution	100 %
Phase partial Fourier	Off
Trajectory	Cartesian
View sharing	Off
Interpolation	Off

Resolution - iPAT

PAT mode	GRAPPA
Accel. factor PE	3
Ref. lines PE	24
Reference scan mode	GRE/separate

Resolution - Filter Image

Image Filter	On
! Intensity	Medium
Edge Enhancement	2
Smoothing	2
Unfiltered images	Off
Distortion Corr.	On
Mode	2D
Unfiltered images	Off
Prescan Normalize	On
Unfiltered images	Off
Normalize	Off
B1 filter	Off

Resolution - Filter Rawdata

Raw filter	Off
Elliptical filter	Off
POCS	Off

Geometry - Common

Slice group	1
Slices	1
Dist. factor	43 %
Position	L12.2 A31.4 H10.6 mm
Orientation	T > C36.0 > S-5.8
Phase enc. dir.	A >> P
FoV read	380 mm
FoV phase	100.0 %
Slice thickness	7.0 mm
TR	40.50 ms
Multi-slice mode	Sequential
Series	Interleaved
Concatenations	1

Geometry - AutoAlign

Slice group	1
Position	L12.2 A31.4 H10.6 mm
Orientation	T > C36.0 > S-5.8
Phase enc. dir.	A >> P
AutoAlign	---
Initial Position	L12.2 A31.4 H10.6
Phase	-18.4 mm
Read	-10.8 mm
Shift	28.1 mm
Initial Rotation	4.22 deg
Initial Orientation	T > C
T > C	36.0
> S	-5.8

Geometry - Saturation

Fat suppr.	None
Wrap-up Magn.	Restore
Special sat.	None

Geometry - Navigator

System - Miscellaneous

Positioning mode	REF
Table position	H
Table position	0 mm
MSMA	S - C - T
Sagittal	R >> L
Coronal	A >> P
Transversal	F >> H
Coil Combine Mode	Adaptive Combine
Save uncombined	Off
Matrix Optimization	Off
Coil Focus	Flat
AutoAlign	---
Coil Select Mode	Default

System - Adjustments

B0 Shim mode	Tune up
Adjust with body coil	On
Confirm freq. adjustment	Off
Assume Dominant Fat	Off
Assume Silicone	Off
Adjustment Tolerance	Auto

System - Adjust Volume

Position	Isocenter
Orientation	Transversal
Rotation	0.00 deg
A >> P	263 mm
R >> L	350 mm
F >> H	350 mm
Reset	Off

System - Tx/Rx

Frequency 1H	63.683250 MHz
Correction factor	1
Gain	High
Img. Scale Cor.	1.000
Reset	Off
? Ref. amplitude 1H	0.000 V

Physio - Signal1

1st Signal/Mode	ECG/Retro
Average cycle	787 ± 129 ms
Average cycle	No Signal ms
Calculated phases	25
TR	40.50 ms
Concatenations	1
Segments	15
Arrhythmia detection	None

Physio - Cardiac

Tagging	None
Magn. preparation	None
Fat suppr.	None
Dark blood	Off
FoV read	380 mm
FoV phase	100.0 %
Phase resolution	100 %
Cine	On
Trajectory	Cartesian
View sharing	Off
Dummy heartbeats	1

Physio - PACE

Resp. control	Off
Concatenations	1

Inline - Common

Subtract	Off
Measurements	1
StdDev	Off
Save original images	On

Inline - Cardiac

Inline Evaluation	Off
Magn. preparation	None
Contrasts	1
TE	1.14 ms
TR	40.50 ms
Save original images	On

Inline - MIP

MIP-Sag	Off
MIP-Cor	Off
MIP-Tra	Off
MIP-Time	Off
Save original images	On

Inline - Composing

Inline Composing	Off
Distortion Corr.	On
Mode	2D
Unfiltered images	Off

Sequence - Part 1

Introduction	Off
Dimension	2D
Reordering	Linear
Asymmetric echo	Weak
Contrasts	1
Optimization	Min. TE TR
Multi-slice mode	Sequential
Echo spacing	2.7 ms
Sequence type	Trufi
Bandwidth	930 Hz/Px

Sequence - Part 2

Define	Segments
Segments	15
Trufi delta freq.	0 Hz
RF pulse type	Fast
Gradient mode	Fast*
Excitation	Slice-sel.
Flip angle mode	Constant
Cine	On

Sequence - Assistant

Mode	Off
Allowed delay	0 s

\\USER\Cardiac Research Protocols\Rapi-STRESS T1\Rapi-STRESS T1 (V2)\T2Map_TrueFISP
TA: 0:14 PM: REF Voxel size: 1.9×1.9×8.0 mmPAT: 2 Rel. SNR: 1.00 : tfi

Properties

Prio recon	Off
Load images to viewer	On
Inline movie	Off
Auto store images	On
Load images to stamp segments	On
Load images to graphic segments	On
Auto open inline display	Off
Auto close inline display	Off
Start measurement without further preparation	Off
Wait for user to start	Off
Start measurements	Single measurement

Routine

Slice group	1
Slices	1
Dist. factor	25 %
Position	L52.9 A23.8 H36.1 mm
Orientation	S > C30.1 > T20.5
Phase enc. dir.	A >> P
AutoAlign	---
Phase oversampling	0 %
FoV read	360 mm
FoV phase	80.2 %
Slice thickness	8.0 mm
TR	193.27 ms
TE	1.07 ms
Averages	1
Concatenations	1
Filter	Distortion Corr.(2D)
Coil elements	BO1-3;SP1-3

Contrast - Common

TR	193.27 ms
TE	1.07 ms
Magn. preparation	T2 prep. adiab.
T2 prep. duration 1	0 ms
T2 prep. duration 2	25 ms
T2 prep. duration 3	55 ms
Flip angle	70 deg
Fat suppr.	None
Wrap-up Magn.	None

Contrast - Dynamic

Averages	1
Averaging mode	Short term
Reconstruction	Magnitude
Measurements	1
Multiple series	Off

Resolution - Common

FoV read	360 mm
FoV phase	80.2 %
Slice thickness	8.0 mm
Base resolution	192
Phase resolution	75 %
Phase partial Fourier	6/8
Trajectory	Cartesian
Interpolation	Off

Resolution - iPAT

PAT mode	GRAPPA
Accel. factor PE	2
Ref. lines PE	36
Reference scan mode	GRE/separate

Resolution - Filter Image

Image Filter	Off
Distortion Corr.	On
Mode	2D
Unfiltered images	Off
Prescan Normalize	Off
Normalize	Off
B1 filter	Off

Resolution - Filter Rawdata

Raw filter	Off
Elliptical filter	Off
POCS	Off

Geometry - Common

Slice group	1
Slices	1
Dist. factor	25 %
Position	L52.9 A23.8 H36.1 mm
Orientation	S > C30.1 > T20.5
Phase enc. dir.	A >> P
FoV read	360 mm
FoV phase	80.2 %
Slice thickness	8.0 mm
TR	193.27 ms
Multi-slice mode	Sequential
Series	Base To Apex
Concatenations	1

Geometry - AutoAlign

Slice group	1
Position	L52.9 A23.8 H36.1 mm
Orientation	S > C30.1 > T20.5
Phase enc. dir.	A >> P
AutoAlign	---
Initial Position	L52.9 A23.8 F15.9
Phase	4.8 mm
Read	6.4 mm
Shift	59.6 mm
Initial Rotation	11.49 deg
Initial Orientation	S > C
S > C	30.1
> T	20.5

Geometry - Saturation

Fat suppr.	None
Wrap-up Magn.	None
Special sat.	None

Geometry - Navigator

System - Miscellaneous

Positioning mode	REF
Table position	H
Table position	52 mm

System - Miscellaneous

MSMA	S - C - T
Sagittal	R >> L
Coronal	A >> P
Transversal	F >> H
Coil Combine Mode	Adaptive Combine
Save uncombined	Off
Matrix Optimization	Off
Coil Focus	Flat
AutoAlign	---
Coil Select Mode	Default

System - Adjustments

B0 Shim mode	Tune up
Adjust with body coil	Off
Confirm freq. adjustment	Off
Assume Dominant Fat	Off
Assume Silicone	Off
Adjustment Tolerance	Auto

System - Adjust Volume

Position	Isocenter
Orientation	Transversal
Rotation	0.00 deg
A >> P	263 mm
R >> L	350 mm
F >> H	350 mm
Reset	Off

System - Tx/Rx

Frequency 1H	63.683250 MHz
Correction factor	1
Gain	High
Img. Scale Cor.	1.000
Reset	Off
? Ref. amplitude 1H	0.000 V

Physio - Signal1

1st Signal/Mode	ECG/Trigger
Average cycle	787 ± 129 ms
Average cycle	No Signal ms
Captured cycle	787 ± 129 ms
Acquisition window	800 ms
Trigger pulse	1
Trigger delay	605 ms
TR	193.27 ms
Concatenations	1
Segments	58
Phases	1
Adaptive Triggering	Off

Physio - Cardiac

Tagging	None
Magn. preparation	T2 prep. adiab.
T2 prep. duration 1	0 ms
T2 prep. duration 2	25 ms
T2 prep. duration 3	55 ms
Fat suppr.	None
Dark blood	Off
FoV read	360 mm
FoV phase	80.2 %
Phase resolution	75 %
Cine	Off
Trajectory	Cartesian
Dummy heartbeats	0

Physio - Cardiac

Motion Correction	Standard

Physio - PACE

Resp. control	Breath-hold
Concatenations	1

Sequence - Part 1

Introduction	Off
Dimension	2D
Reordering	Linear
Asymmetric echo	Weak
Contrasts	1
Optimization	Min. TE TR
Multi-slice mode	Sequential
Sequence type	Trufi
Bandwidth	1184 Hz/Px

Sequence - Part 2

Define	Shots
Shots per slice	1
Segments	58
Trufi delta freq.	0 Hz
RF pulse type	Fast
Gradient mode	Fast
Excitation	Slice-sel.
Flip angle mode	Constant
Cine	Off

Sequence - Assistant

Mode	Off
Allowed delay	0 s

\\USER\Cardiac Research Protocols\Rapi-STRESS T1\Rapi-STRESS T1 (V2)\TEST SSFP_Perf_MBF_MBV_aif_r3 (KELLMAN)

TA: 9.4 s PM: REF Voxel size: 1.9×1.9×8.0 mmPAT: 3 Rel. SNR: 1.00 : tfi

Properties

Prio recon	Off
Load images to viewer	Off
Inline movie	Off
Auto store images	On
Load images to stamp segments	Off
Load images to graphic segments	On
Auto open inline display	Off
Auto close inline display	Off
Start measurement without further preparation	Off
Wait for user to start	On
Start measurements	Single measurement

Routine

Slice group	1
Slices	3
Dist. factor	100 %
Position	Isocenter
Orientation	Transversal
Phase enc. dir.	A >> P
AutoAlign	---
Phase oversampling	0 %
FoV read	360 mm
FoV phase	75.0 %
Slice thickness	8.0 mm
TR	142.00 ms
TE	1.04 ms
Averages	1
Concatenations	1
Filter	Distortion Corr.(2D)
Coil elements	BO1-3;SP3,4

Contrast - Common

TR	142.00 ms
TE	1.04 ms
Magn. preparation	Non-sel. SR perf
TI	105 ms
Flip angle	50 deg
Fat suppr.	Fat sat.
Wrap-up Magn.	None

Contrast - Dynamic

Averages	1
Averaging mode	Long term
Reconstruction	Magnitude
Measurements	12
Pause after meas. 1	0.0 s
Pause after meas. 2	0.0 s
Pause after meas. 3	0.0 s
Pause after meas. 4	0.0 s
Pause after meas. 5	0.0 s
Pause after meas. 6	0.0 s
Pause after meas. 7	0.0 s
Pause after meas. 8	0.0 s
Pause after meas. 9	0.0 s
Pause after meas. 10	0.0 s
Pause after meas. 11	0.0 s
Proton Dens. Maps	3
Multiple series	Off

Resolution - Common

FoV read	360 mm
FoV phase	75.0 %
Slice thickness	8.0 mm
Base resolution	192
Phase resolution	77 %
Phase partial Fourier	6/8
Trajectory	Cartesian
Interpolation	Off

Resolution - iPAT

PAT mode	GRAPPA
Accel. factor PE	3
Reference scan mode	T-PAT

Resolution - Filter Image

Image Filter	Off
Distortion Corr.	On
Mode	2D
Unfiltered images	Off
Prescan Normalize	Off
Normalize	Off
B1 filter	Off

Resolution - Filter Rawdata

Raw filter	Off
Elliptical filter	Off
POCS	Off

Geometry - Common

Slice group	1
Slices	3
Dist. factor	100 %
Position	Isocenter
Orientation	Transversal
Phase enc. dir.	A >> P
FoV read	360 mm
FoV phase	75.0 %
Slice thickness	8.0 mm
TR	142.00 ms
Multi-slice mode	Single shot
Series	Ascending
Concatenations	1

Geometry - AutoAlign

Slice group	1
Position	Isocenter
Orientation	Transversal
Phase enc. dir.	A >> P
AutoAlign	---
Initial Position	Isocenter
Phase	0.0 mm
Read	0.0 mm
Shift	0.0 mm
Initial Rotation	0.00 deg
Initial Orientation	Transversal

Geometry - Saturation

Fat suppr.	Fat sat.
Wrap-up Magn.	None

Geometry - Saturation

Special sat.	None

Geometry - Navigator

System - Miscellaneous

Positioning mode	REF
Table position	H
Table position	0 mm
MSMA	S - C - T
Sagittal	R >> L
Coronal	A >> P
Transversal	F >> H
Coil Combine Mode	Sum of Squares
Save uncombined	Off
Matrix Optimization	Off
Coil Focus	Flat
AutoAlign	---
Coil Select Mode	Default

System - Adjustments

B0 Shim mode	Cardiac
Adjust with body coil	On
Confirm freq. adjustment	Off
Assume Dominant Fat	Off
Assume Silicone	Off
Adjustment Tolerance	Auto

System - Adjust Volume

! Position	Isocenter
! Orientation	Transversal
! Rotation	0.00 deg
! A >> P	150 mm
! R >> L	150 mm
! F >> H	150 mm
Reset	Off

System - Tx/Rx

Frequency 1H	63.683250 MHz
Correction factor	1
Gain	High
Img. Scale Cor.	1.000
Reset	Off
? Ref. amplitude 1H	0.000 V

Physio - Signal1

1st Signal/Mode	ECG/Trigger
Average cycle	787 ± 129 ms
Average cycle	No Signal ms
Captured cycle	787 ± 129 ms
Acquisition window	493 ms
Trigger pulse	1
Trigger delay	0 ms
TR	142.00 ms
Concatenations	1
Segments	37
Phases	1
Adaptive Triggering	Off

Physio - Cardiac

Tagging	None
Magn. preparation	Non-sel. SR perf
TI	105 ms
Fat suppr.	Fat sat.

Physio - Cardiac

Dark blood	Off
FoV read	360 mm
FoV phase	75.0 %
Phase resolution	77 %
Cine	Off
Trajectory	Cartesian
Dummy heartbeats	0
Motion Correction	None

Physio - PACE

Resp. control	Off
Concatenations	1

Inline - Common

Subtract	Off
Measurements	12
StdDev	Off
Motion Correction	None
Save original images	On

Inline - Cardiac

Inline Evaluation	Off
Magn. preparation	Non-sel. SR perf
TE	1.04 ms
TR	142.00 ms
Motion Correction	None
Save original images	On

Inline - MIP

MIP-Sag	Off
MIP-Cor	Off
MIP-Tra	Off
MIP-Time	Off
Save original images	On

Inline - Composing

Inline Composing	Off
Distortion Corr.	On
Mode	2D
Unfiltered images	Off

Sequence - Part 1

Introduction	Off
Dimension	2D
Reordering	Linear
Asymmetric echo	Allowed
Optimization	Min. TE
Multi-slice mode	Single shot
Echo spacing	2.5 ms
Sequence type	Trufi
Bandwidth	1085 Hz/Px

Sequence - Part 2

Define	Shots
Shots per slice	1
EPI factor	1
Segments	37
Trufi delta freq.	0 Hz
RF pulse type	Fast
Gradient mode	Fast
Excitation	Slice-sel.
Flip angle mode	Constant
Cine	Off

Sequence - Special

AIF Images	On
AIF SR Preparation	SR_PERF
Scan	Test
Gadgetron IPR	PERF2
Temporal Filter	On
Filter Method	Gaussian
Filter Strength	Medium

Sequence - Assistant

Mode	Min flip angle
Min flip angle	45 deg
Allowed delay	100 s

\\USER\Cardiac Research Protocols\Rapi-STRESS T1\Rapi-STRESS T1 (V2)\MidShMOLLI_192i_d11_nFilt (TD=0)

TA: 7.1 s PM: REF Voxel size: 0.9×0.9×8.0 mmPAT: 2 Rel. SNR: 1.00 : tfi

Properties

Prio recon	Off
Load images to viewer	On
Inline movie	Off
Auto store images	On
Load images to stamp segments	Off
Load images to graphic segments	On
Auto open inline display	Off
Auto close inline display	Off
Start measurement without further preparation	Off
Wait for user to start	Off
Start measurements	Single measurement

Routine

Slice group	1
Slices	1
Dist. factor	25 %
Position	Isocenter
Orientation	Transversal
Phase enc. dir.	A >> P
AutoAlign	---
Phase oversampling	0 %
FoV read	360 mm
FoV phase	75.0 %
Slice thickness	8.0 mm
TR	378.98 ms
TE	1.07 ms
Averages	1
Concatenations	1
Filter	Raw filter, Distortion Corr.(2D)
Coil elements	BO2;SP2,3

Contrast - Common

TR	378.98 ms
TE	1.07 ms
Magn. preparation	Non-sel. IR
TI	260 ms
Flip angle	35 deg
Fat suppr.	None
Wrap-up Magn.	None

Contrast - Dynamic

Averages	1
Averaging mode	Short term
Reconstruction	Magn./Phase
Measurements	1
Multiple series	Off

Resolution - Common

FoV read	360 mm
FoV phase	75.0 %
Slice thickness	8.0 mm
Base resolution	192
Phase resolution	100 %
Phase partial Fourier	6/8
Trajectory	Cartesian
Interpolation	On

Resolution - iPAT

PAT mode	GRAPPA
Accel. factor PE	2
Ref. lines PE	24
Reference scan mode	Integrated

Resolution - Filter Image

Image Filter	Off
Distortion Corr.	On
Mode	2D
Unfiltered images	Off
Prescan Normalize	Off
Normalize	Off
B1 filter	Off

Resolution - Filter Rawdata

Raw filter	On
Elliptical filter	Off
POCS	Off

Geometry - Common

Slice group	1
Slices	1
Dist. factor	25 %
Position	Isocenter
Orientation	Transversal
Phase enc. dir.	A >> P
FoV read	360 mm
FoV phase	75.0 %
Slice thickness	8.0 mm
TR	378.98 ms
Multi-slice mode	Sequential
Series	Interleaved
Concatenations	1

Geometry - AutoAlign

Slice group	1
Position	Isocenter
Orientation	Transversal
Phase enc. dir.	A >> P
AutoAlign	---
Initial Position	Isocenter
Phase	0.0 mm
Read	0.0 mm
Shift	0.0 mm
Initial Rotation	0.00 deg
Initial Orientation	Transversal

Geometry - Saturation

Fat suppr.	None
Wrap-up Magn.	None
Special sat.	None

Geometry - Navigator

System - Miscellaneous

Positioning mode	REF
Table position	H
Table position	0 mm

System - Miscellaneous

MSMA	S - C - T
Sagittal	R >> L
Coronal	A >> P
Transversal	F >> H
Coil Combine Mode	Adaptive Combine
Save uncombined	Off
Matrix Optimization	Off
Coil Focus	Flat
AutoAlign	---
Coil Select Mode	Default

System - Adjustments

B0 Shim mode	Cardiac
Adjust with body coil	On
Confirm freq. adjustment	Off
Assume Dominant Fat	Off
Assume Silicone	Off
Adjustment Tolerance	Auto

System - Adjust Volume

! Position	Isocenter
! Orientation	Transversal
! Rotation	0.00 deg
! A >> P	150 mm
! R >> L	150 mm
! F >> H	150 mm
Reset	Off

System - Tx/Rx

Frequency 1H	63.683250 MHz
Correction factor	1
Gain	High
Img. Scale Cor.	1.000
Reset	Off
? Ref. amplitude 1H	0.000 V

Physio - Signal1

1st Signal/Mode	ECG/Trigger
Average cycle	787 ± 129 ms
Average cycle	No Signal ms
Captured cycle	787 ± 129 ms
Acquisition window	379 ms
Trigger pulse	1
Trigger delay	0 ms
TR	378.98 ms
Concatenations	1
Segments	84
Phases	1
Adaptive Triggering	Off

Physio - Cardiac

Tagging	None
Magn. preparation	Non-sel. IR
TI	260 ms
Fat suppr.	None
Dark blood	Off
FoV read	360 mm
FoV phase	75.0 %
Phase resolution	100 %
Cine	Off
Trajectory	Cartesian
Dummy heartbeats	0
Motion Correction	None

Physio - PACE

Resp. control	Off
Concatenations	1

Sequence - Part 1

Introduction	Off
Dimension	2D
Reordering	Linear
Asymmetric echo	Weak
Contrasts	1
Optimization	Min. TE TR
Multi-slice mode	Sequential
Sequence type	Trufi
Bandwidth	898 Hz/Px

Sequence - Part 2

Define	Shots
Shots per slice	1
Segments	84
Trufi delta freq.	0 Hz
RF pulse type	Fast
Gradient mode	Fast
Excitation	Slice-sel.
Flip angle mode	Constant
Cine	Off

Sequence - Assistant

Mode	Off
Allowed delay	0 s

\\USER\Cardiac Research Protocols\Rapi-STRESS T1\Rapi-STRESS T1 (V2)\T1Map_LongT1 (TD=0)LowHR

TA: 8.7 s PM: FIX Voxel size: 1.4×1.4×8.0 mmPAT: 2 Rel. SNR: 1.00 : tfi

Properties

Prio recon	Off
Load images to viewer	On
Inline movie	Off
Auto store images	On
Load images to stamp segments	On
Load images to graphic segments	On
Auto open inline display	Off
Auto close inline display	Off
Start measurement without further preparation	On
Wait for user to start	Off
Start measurements	Single measurement

Routine

Slice group	1
Slices	1
Dist. factor	25 %
Position	Isocenter
Orientation	Transversal
Phase enc. dir.	A >> P
AutoAlign	---
Phase oversampling	0 %
FoV read	360 mm
FoV phase	85.2 %
Slice thickness	8.0 mm
TR	279.84 ms
TE	1.13 ms
Averages	1
Concatenations	1
Filter	Distortion Corr.(2D)
Coil elements	BO2;SP2,3

Contrast - Common

TR	279.84 ms
TE	1.13 ms
Magn. preparation	Non-sel. IR T1map
TI	180 ms
Flip angle	35 deg
Fat suppr.	None
Wrap-up Magn.	None

Contrast - Dynamic

Averages	1
Averaging mode	Short term
Reconstruction	Magnitude
Measurements	1
Multiple series	Off

Resolution - Common

FoV read	360 mm
FoV phase	85.2 %
Slice thickness	8.0 mm
Base resolution	256
Phase resolution	66 %
Phase partial Fourier	7/8
Trajectory	Cartesian
Interpolation	Off

Resolution - iPAT

PAT mode	GRAPPA
Accel. factor PE	2
Ref. lines PE	36
Reference scan mode	GRE/separate

Resolution - Filter Image

Image Filter	Off
Distortion Corr.	On
Mode	2D
Unfiltered images	Off
Prescan Normalize	Off
Normalize	Off
B1 filter	Off

Resolution - Filter Rawdata

Raw filter	Off
Elliptical filter	Off
POCS	Off

Geometry - Common

Slice group	1
Slices	1
Dist. factor	25 %
Position	Isocenter
Orientation	Transversal
Phase enc. dir.	A >> P
FoV read	360 mm
FoV phase	85.2 %
Slice thickness	8.0 mm
TR	279.84 ms
Multi-slice mode	Sequential
Series	Base To Apex
Concatenations	1

Geometry - AutoAlign

Slice group	1
Position	Isocenter
Orientation	Transversal
Phase enc. dir.	A >> P
AutoAlign	---
Initial Position	Isocenter
Phase	0.0 mm
Read	0.0 mm
Shift	0.0 mm
Initial Rotation	0.00 deg
Initial Orientation	Transversal

Geometry - Saturation

Fat suppr.	None
Wrap-up Magn.	None
Special sat.	None

Geometry - Navigator

System - Miscellaneous

Positioning mode	FIX
Table position	H
Table position	0 mm

System - Miscellaneous

MSMA	S - C - T
Sagittal	R >> L
Coronal	A >> P
Transversal	F >> H
Coil Combine Mode	Adaptive Combine
Save uncombined	Off
Matrix Optimization	Off
Coil Focus	Flat
AutoAlign	---
Coil Select Mode	Default

System - Adjustments

B0 Shim mode	Cardiac
Adjust with body coil	Off
Confirm freq. adjustment	Off
Assume Dominant Fat	Off
Assume Silicone	Off
Adjustment Tolerance	Auto

System - Adjust Volume

! Position	Isocenter
! Orientation	Transversal
! Rotation	0.00 deg
! A >> P	150 mm
! R >> L	150 mm
! F >> H	150 mm
Reset	Off

System - Tx/Rx

Frequency 1H	63.683250 MHz
Correction factor	1
Gain	High
Img. Scale Cor.	1.000
Reset	Off
? Ref. amplitude 1H	0.000 V

Physio - Signal1

1st Signal/Mode	ECG/Trigger
Average cycle	787 ± 129 ms
Average cycle	No Signal ms
Captured cycle	787 ± 129 ms
Acquisition window	280 ms
Trigger pulse	1
Trigger delay	0 ms
TR	279.84 ms
Concatenations	1
Segments	72
Phases	1
Adaptive Triggering	Off

Physio - Cardiac

Tagging	None
Magn. preparation	Non-sel. IR T1map
TI	180 ms
Fat suppr.	None
Dark blood	Off
FoV read	360 mm
FoV phase	85.2 %
Phase resolution	66 %
Cine	Off
Trajectory	Cartesian
Dummy heartbeats	0
Motion Correction	Standard

Physio - PACE

Resp. control	Breath-hold
Concatenations	1

Sequence - Part 1

Introduction	Off
Dimension	2D
Reordering	Linear
Asymmetric echo	Weak
Contrasts	1
Optimization	Min. TE TR
Multi-slice mode	Sequential
Sequence type	Trufi
Bandwidth	1085 Hz/Px

Sequence - Part 2

Define	Shots
Shots per slice	1
Segments	72
Trufi delta freq.	0 Hz
RF pulse type	Fast
Gradient mode	Fast
Excitation	Slice-sel.
Flip angle mode	Constant
Cine	Off

Sequence - Assistant

Mode	Off
Allowed delay	0 s

\\USER\Cardiac Research Protocols\Rapi-STRESS T1\Rapi-STRESS T1 (V2)\ShMOLLI_192i_d11_nFilt (TD=0)

TA: 7.1 s PM: FIX Voxel size: 0.9×0.9×8.0 mmPAT: 2 Rel. SNR: 1.00 : tfi

Properties

Prio recon	Off
Load images to viewer	On
Inline movie	Off
Auto store images	On
Load images to stamp segments	Off
Load images to graphic segments	On
Auto open inline display	Off
Auto close inline display	Off
Start measurement without further preparation	On
Wait for user to start	Off
Start measurements	Single measurement

Routine

Slice group	1
Slices	1
Dist. factor	25 %
Position	Isocenter
Orientation	Transversal
Phase enc. dir.	A >> P
AutoAlign	---
Phase oversampling	0 %
FoV read	360 mm
FoV phase	75.0 %
Slice thickness	8.0 mm
TR	378.98 ms
TE	1.07 ms
Averages	1
Concatenations	1
Filter	Raw filter, Distortion Corr.(2D)
Coil elements	BO2;SP2,3

Contrast - Common

TR	378.98 ms
TE	1.07 ms
Magn. preparation	Non-sel. IR
TI	260 ms
Flip angle	35 deg
Fat suppr.	None
Wrap-up Magn.	None

Contrast - Dynamic

Averages	1
Averaging mode	Short term
Reconstruction	Magn./Phase
Measurements	1
Multiple series	Off

Resolution - Common

FoV read	360 mm
FoV phase	75.0 %
Slice thickness	8.0 mm
Base resolution	192
Phase resolution	100 %
Phase partial Fourier	6/8
Trajectory	Cartesian
Interpolation	On

Resolution - iPAT

PAT mode	GRAPPA
Accel. factor PE	2
Ref. lines PE	24
Reference scan mode	Integrated

Resolution - Filter Image

Image Filter	Off
Distortion Corr.	On
Mode	2D
Unfiltered images	Off
Prescan Normalize	Off
Normalize	Off
B1 filter	Off

Resolution - Filter Rawdata

Raw filter	On
Elliptical filter	Off
POCS	Off

Geometry - Common

Slice group	1
Slices	1
Dist. factor	25 %
Position	Isocenter
Orientation	Transversal
Phase enc. dir.	A >> P
FoV read	360 mm
FoV phase	75.0 %
Slice thickness	8.0 mm
TR	378.98 ms
Multi-slice mode	Sequential
Series	Interleaved
Concatenations	1

Geometry - AutoAlign

Slice group	1
Position	Isocenter
Orientation	Transversal
Phase enc. dir.	A >> P
AutoAlign	---
Initial Position	Isocenter
Phase	0.0 mm
Read	0.0 mm
Shift	0.0 mm
Initial Rotation	0.00 deg
Initial Orientation	Transversal

Geometry - Saturation

Fat suppr.	None
Wrap-up Magn.	None
Special sat.	None

Geometry - Navigator

System - Miscellaneous

Positioning mode	FIX
Table position	H
Table position	0 mm

System - Miscellaneous

MSMA	S - C - T
Sagittal	R >> L
Coronal	A >> P
Transversal	F >> H
Coil Combine Mode	Adaptive Combine
Save uncombined	Off
Matrix Optimization	Off
Coil Focus	Flat
AutoAlign	---
Coil Select Mode	Default

System - Adjustments

B0 Shim mode	Cardiac
Adjust with body coil	On
Confirm freq. adjustment	Off
Assume Dominant Fat	Off
Assume Silicone	Off
Adjustment Tolerance	Auto

System - Adjust Volume

! Position	Isocenter
! Orientation	Transversal
! Rotation	0.00 deg
! A >> P	150 mm
! R >> L	150 mm
! F >> H	150 mm
Reset	Off

System - Tx/Rx

Frequency 1H	63.683250 MHz
Correction factor	1
Gain	High
Img. Scale Cor.	1.000
Reset	Off
? Ref. amplitude 1H	0.000 V

Physio - Signal1

1st Signal/Mode	ECG/Trigger
Average cycle	787 ± 129 ms
Average cycle	No Signal ms
Captured cycle	787 ± 129 ms
Acquisition window	379 ms
Trigger pulse	1
Trigger delay	0 ms
TR	378.98 ms
Concatenations	1
Segments	84
Phases	1
Adaptive Triggering	Off

Physio - Cardiac

Tagging	None
Magn. preparation	Non-sel. IR
TI	260 ms
Fat suppr.	None
Dark blood	Off
FoV read	360 mm
FoV phase	75.0 %
Phase resolution	100 %
Cine	Off
Trajectory	Cartesian
Dummy heartbeats	0
Motion Correction	None

Physio - PACE

Resp. control	Off
Concatenations	1

Sequence - Part 1

Introduction	Off
Dimension	2D
Reordering	Linear
Asymmetric echo	Weak
Contrasts	1
Optimization	Min. TE TR
Multi-slice mode	Sequential
Sequence type	Trufi
Bandwidth	898 Hz/Px

Sequence - Part 2

Define	Shots
Shots per slice	1
Segments	84
Trufi delta freq.	0 Hz
RF pulse type	Fast
Gradient mode	Fast
Excitation	Slice-sel.
Flip angle mode	Constant
Cine	Off

Sequence - Assistant

Mode	Off
Allowed delay	0 s

\\USER\Cardiac Research Protocols\Rapi-STRESS T1\Rapi-STRESS T1 (V2)\T1Map_LongT1_TD0_HighHR

TA: 8.7 s PM: FIX Voxel size: 1.9×1.9×8.0 mmPAT: 2 Rel. SNR: 1.00 : tfi

Properties

Prio recon	Off
Load images to viewer	On
Inline movie	Off
Auto store images	On
Load images to stamp segments	On
Load images to graphic segments	On
Auto open inline display	Off
Auto close inline display	Off
Start measurement without further preparation	On
Wait for user to start	Off
Start measurements	Single measurement

Routine

Slice group	1
Slices	1
Dist. factor	20 %
Position	Isocenter
Orientation	Transversal
Phase enc. dir.	A >> P
AutoAlign	---
Phase oversampling	0 %
FoV read	360 mm
FoV phase	85.4 %
Slice thickness	8.0 mm
TR	263.55 ms
TE	1.01 ms
Averages	1
Concatenations	1
Filter	Distortion Corr.(2D)
Coil elements	BO2;SP2,3

Contrast - Common

TR	263.55 ms
TE	1.01 ms
Magn. preparation	Non-sel. IR T1map
TI	180 ms
Flip angle	35 deg
Fat suppr.	None
Wrap-up Magn.	None

Contrast - Dynamic

Averages	1
Averaging mode	Short term
Reconstruction	Magnitude
Measurements	1
Multiple series	Off

Resolution - Common

FoV read	360 mm
FoV phase	85.4 %
Slice thickness	8.0 mm
Base resolution	192
Phase resolution	80 %
Phase partial Fourier	7/8
Trajectory	Cartesian
Interpolation	Off

Resolution - iPAT

PAT mode	GRAPPA
Accel. factor PE	2
Ref. lines PE	36
Reference scan mode	GRE/separate

Resolution - Filter Image

Image Filter	Off
Distortion Corr.	On
Mode	2D
Unfiltered images	Off
Prescan Normalize	Off
Normalize	Off
B1 filter	Off

Resolution - Filter Rawdata

Raw filter	Off
Elliptical filter	Off
POCS	Off

Geometry - Common

Slice group	1
Slices	1
Dist. factor	20 %
Position	Isocenter
Orientation	Transversal
Phase enc. dir.	A >> P
FoV read	360 mm
FoV phase	85.4 %
Slice thickness	8.0 mm
TR	263.55 ms
Multi-slice mode	Sequential
Series	Base To Apex
Concatenations	1

Geometry - AutoAlign

Slice group	1
Position	Isocenter
Orientation	Transversal
Phase enc. dir.	A >> P
AutoAlign	---
Initial Position	Isocenter
Phase	0.0 mm
Read	0.0 mm
Shift	0.0 mm
Initial Rotation	0.00 deg
Initial Orientation	Transversal

Geometry - Saturation

Fat suppr.	None
Wrap-up Magn.	None
Special sat.	None

Geometry - Navigator

System - Miscellaneous

Positioning mode	FIX
Table position	H
Table position	0 mm

System - Miscellaneous

MSMA	S - C - T
Sagittal	R >> L
Coronal	A >> P
Transversal	F >> H
Coil Combine Mode	Adaptive Combine
Save uncombined	Off
Matrix Optimization	Off
Coil Focus	Flat
AutoAlign	---
Coil Select Mode	Default

System - Adjustments

B0 Shim mode	Cardiac
Adjust with body coil	Off
Confirm freq. adjustment	Off
Assume Dominant Fat	Off
Assume Silicone	Off
Adjustment Tolerance	Auto

System - Adjust Volume

! Position	Isocenter
! Orientation	Transversal
! Rotation	0.00 deg
! A >> P	150 mm
! R >> L	150 mm
! F >> H	150 mm
Reset	Off

System - Tx/Rx

Frequency 1H	63.683250 MHz
Correction factor	1
Gain	High
Img. Scale Cor.	1.000
Reset	Off
? Ref. amplitude 1H	0.000 V

Physio - Signal1

1st Signal/Mode	ECG/Trigger
Average cycle	787 ± 129 ms
Average cycle	No Signal ms
Captured cycle	787 ± 129 ms
Acquisition window	700 ms
Trigger pulse	1
Trigger delay	0 ms
TR	263.55 ms
Concatenations	1
Segments	66
Phases	1
Adaptive Triggering	Off

Physio - Cardiac

Tagging	None
Magn. preparation	Non-sel. IR T1map
TI	180 ms
Fat suppr.	None
Dark blood	Off
FoV read	360 mm
FoV phase	85.4 %
Phase resolution	80 %
Cine	Off
Trajectory	Cartesian
Dummy heartbeats	0
Motion Correction	Standard

Physio - PACE

Resp. control	Breath-hold
Concatenations	1

Sequence - Part 1

Introduction	Off
Dimension	2D
Reordering	Linear
Asymmetric echo	Weak
Contrasts	1
Optimization	Min. TE TR
Multi-slice mode	Sequential
Sequence type	Trufi
Bandwidth	1085 Hz/Px

Sequence - Part 2

Define	Shots
Shots per slice	1
Segments	66
Trufi delta freq.	0 Hz
RF pulse type	Fast
Gradient mode	Fast
Excitation	Slice-sel.
Flip angle mode	Constant
Cine	Off

Sequence - Assistant

Mode	Off
Allowed delay	0 s

\\USER\Cardiac Research Protocols\Rapi-STRESS T1\Rapi-STRESS T1 (V2)\ShMOLLI_192i_d11_nFilt (TD=0)

TA: 7.1 s PM: FIX Voxel size: 0.9×0.9×8.0 mmPAT: 2 Rel. SNR: 1.00 : tfi

Properties

Prio recon	Off
Load images to viewer	On
Inline movie	Off
Auto store images	On
Load images to stamp segments	Off
Load images to graphic segments	On
Auto open inline display	Off
Auto close inline display	Off
Start measurement without further preparation	On
Wait for user to start	Off
Start measurements	Single measurement

Routine

Slice group	1
Slices	1
Dist. factor	25 %
Position	Isocenter
Orientation	Transversal
Phase enc. dir.	A >> P
AutoAlign	---
Phase oversampling	0 %
FoV read	360 mm
FoV phase	75.0 %
Slice thickness	8.0 mm
TR	378.98 ms
TE	1.07 ms
Averages	1
Concatenations	1
Filter	Raw filter, Distortion Corr.(2D)
Coil elements	BO2;SP2,3

Contrast - Common

TR	378.98 ms
TE	1.07 ms
Magn. preparation	Non-sel. IR
TI	260 ms
Flip angle	35 deg
Fat suppr.	None
Wrap-up Magn.	None

Contrast - Dynamic

Averages	1
Averaging mode	Short term
Reconstruction	Magn./Phase
Measurements	1
Multiple series	Off

Resolution - Common

FoV read	360 mm
FoV phase	75.0 %
Slice thickness	8.0 mm
Base resolution	192
Phase resolution	100 %
Phase partial Fourier	6/8
Trajectory	Cartesian
Interpolation	On

Resolution - iPAT

PAT mode	GRAPPA
Accel. factor PE	2
Ref. lines PE	24
Reference scan mode	Integrated

Resolution - Filter Image

Image Filter	Off
Distortion Corr.	On
Mode	2D
Unfiltered images	Off
Prescan Normalize	Off
Normalize	Off
B1 filter	Off

Resolution - Filter Rawdata

Raw filter	On
Elliptical filter	Off
POCS	Off

Geometry - Common

Slice group	1
Slices	1
Dist. factor	25 %
Position	Isocenter
Orientation	Transversal
Phase enc. dir.	A >> P
FoV read	360 mm
FoV phase	75.0 %
Slice thickness	8.0 mm
TR	378.98 ms
Multi-slice mode	Sequential
Series	Interleaved
Concatenations	1

Geometry - AutoAlign

Slice group	1
Position	Isocenter
Orientation	Transversal
Phase enc. dir.	A >> P
AutoAlign	---
Initial Position	Isocenter
Phase	0.0 mm
Read	0.0 mm
Shift	0.0 mm
Initial Rotation	0.00 deg
Initial Orientation	Transversal

Geometry - Saturation

Fat suppr.	None
Wrap-up Magn.	None
Special sat.	None

Geometry - Navigator

System - Miscellaneous

Positioning mode	FIX
Table position	H
Table position	0 mm

System - Miscellaneous

MSMA	S - C - T
Sagittal	R >> L
Coronal	A >> P
Transversal	F >> H
Coil Combine Mode	Adaptive Combine
Save uncombined	Off
Matrix Optimization	Off
Coil Focus	Flat
AutoAlign	---
Coil Select Mode	Default

System - Adjustments

B0 Shim mode	Cardiac
Adjust with body coil	On
Confirm freq. adjustment	Off
Assume Dominant Fat	Off
Assume Silicone	Off
Adjustment Tolerance	Auto

System - Adjust Volume

! Position	Isocenter
! Orientation	Transversal
! Rotation	0.00 deg
! A >> P	150 mm
! R >> L	150 mm
! F >> H	150 mm
Reset	Off

System - Tx/Rx

Frequency 1H	63.683250 MHz
Correction factor	1
Gain	High
Img. Scale Cor.	1.000
Reset	Off
? Ref. amplitude 1H	0.000 V

Physio - Signal1

1st Signal/Mode	ECG/Trigger
Average cycle	787 ± 129 ms
Average cycle	No Signal ms
Captured cycle	787 ± 129 ms
Acquisition window	379 ms
Trigger pulse	1
Trigger delay	0 ms
TR	378.98 ms
Concatenations	1
Segments	84
Phases	1
Adaptive Triggering	Off

Physio - Cardiac

Tagging	None
Magn. preparation	Non-sel. IR
TI	260 ms
Fat suppr.	None
Dark blood	Off
FoV read	360 mm
FoV phase	75.0 %
Phase resolution	100 %
Cine	Off
Trajectory	Cartesian
Dummy heartbeats	0
Motion Correction	None

Physio - PACE

Resp. control	Off
Concatenations	1

Sequence - Part 1

Introduction	Off
Dimension	2D
Reordering	Linear
Asymmetric echo	Weak
Contrasts	1
Optimization	Min. TE TR
Multi-slice mode	Sequential
Sequence type	Trufi
Bandwidth	898 Hz/Px

Sequence - Part 2

Define	Shots
Shots per slice	1
Segments	84
Trufi delta freq.	0 Hz
RF pulse type	Fast
Gradient mode	Fast
Excitation	Slice-sel.
Flip angle mode	Constant
Cine	Off

Sequence - Assistant

Mode	Off
Allowed delay	0 s

\\USER\Cardiac Research Protocols\Rapi-STRESS T1\Rapi-STRESS T1 (V2)\MidShMOLLI_192i_d11_nFilt (TD=0)

TA: 7.1 s PM: REF Voxel size: 0.9×0.9×8.0 mmPAT: 2 Rel. SNR: 1.00 : tfi

Properties

Prio recon	Off
Load images to viewer	On
Inline movie	Off
Auto store images	On
Load images to stamp segments	Off
Load images to graphic segments	On
Auto open inline display	Off
Auto close inline display	Off
Start measurement without further preparation	Off
Wait for user to start	Off
Start measurements	Single measurement

Routine

Slice group	1
Slices	1
Dist. factor	25 %
Position	Isocenter
Orientation	Transversal
Phase enc. dir.	A >> P
AutoAlign	---
Phase oversampling	0 %
FoV read	360 mm
FoV phase	75.0 %
Slice thickness	8.0 mm
TR	378.98 ms
TE	1.07 ms
Averages	1
Concatenations	1
Filter	Raw filter, Distortion Corr.(2D)
Coil elements	BO2;SP2,3

Contrast - Common

TR	378.98 ms
TE	1.07 ms
Magn. preparation	Non-sel. IR
TI	260 ms
Flip angle	35 deg
Fat suppr.	None
Wrap-up Magn.	None

Contrast - Dynamic

Averages	1
Averaging mode	Short term
Reconstruction	Magn./Phase
Measurements	1
Multiple series	Off

Resolution - Common

FoV read	360 mm
FoV phase	75.0 %
Slice thickness	8.0 mm
Base resolution	192
Phase resolution	100 %
Phase partial Fourier	6/8
Trajectory	Cartesian
Interpolation	On

Resolution - iPAT

PAT mode	GRAPPA
Accel. factor PE	2
Ref. lines PE	24
Reference scan mode	Integrated

Resolution - Filter Image

Image Filter	Off
Distortion Corr.	On
Mode	2D
Unfiltered images	Off
Prescan Normalize	Off
Normalize	Off
B1 filter	Off

Resolution - Filter Rawdata

Raw filter	On
Elliptical filter	Off
POCS	Off

Geometry - Common

Slice group	1
Slices	1
Dist. factor	25 %
Position	Isocenter
Orientation	Transversal
Phase enc. dir.	A >> P
FoV read	360 mm
FoV phase	75.0 %
Slice thickness	8.0 mm
TR	378.98 ms
Multi-slice mode	Sequential
Series	Interleaved
Concatenations	1

Geometry - AutoAlign

Slice group	1
Position	Isocenter
Orientation	Transversal
Phase enc. dir.	A >> P
AutoAlign	---
Initial Position	Isocenter
Phase	0.0 mm
Read	0.0 mm
Shift	0.0 mm
Initial Rotation	0.00 deg
Initial Orientation	Transversal

Geometry - Saturation

Fat suppr.	None
Wrap-up Magn.	None
Special sat.	None

Geometry - Navigator

System - Miscellaneous

Positioning mode	REF
Table position	H
Table position	0 mm

System - Miscellaneous

MSMA	S - C - T
Sagittal	R >> L
Coronal	A >> P
Transversal	F >> H
Coil Combine Mode	Adaptive Combine
Save uncombined	Off
Matrix Optimization	Off
Coil Focus	Flat
AutoAlign	---
Coil Select Mode	Default

System - Adjustments

B0 Shim mode	Cardiac
Adjust with body coil	On
Confirm freq. adjustment	Off
Assume Dominant Fat	Off
Assume Silicone	Off
Adjustment Tolerance	Auto

System - Adjust Volume

! Position	Isocenter
! Orientation	Transversal
! Rotation	0.00 deg
! A >> P	150 mm
! R >> L	150 mm
! F >> H	150 mm
Reset	Off

System - Tx/Rx

Frequency 1H	63.683250 MHz
Correction factor	1
Gain	High
Img. Scale Cor.	1.000
Reset	Off
? Ref. amplitude 1H	0.000 V

Physio - Signal1

1st Signal/Mode	ECG/Trigger
Average cycle	787 ± 129 ms
Average cycle	No Signal ms
Captured cycle	787 ± 129 ms
Acquisition window	379 ms
Trigger pulse	1
Trigger delay	0 ms
TR	378.98 ms
Concatenations	1
Segments	84
Phases	1
Adaptive Triggering	Off

Physio - Cardiac

Tagging	None
Magn. preparation	Non-sel. IR
TI	260 ms
Fat suppr.	None
Dark blood	Off
FoV read	360 mm
FoV phase	75.0 %
Phase resolution	100 %
Cine	Off
Trajectory	Cartesian
Dummy heartbeats	0
Motion Correction	None

Physio - PACE

Resp. control	Off
Concatenations	1

Sequence - Part 1

Introduction	Off
Dimension	2D
Reordering	Linear
Asymmetric echo	Weak
Contrasts	1
Optimization	Min. TE TR
Multi-slice mode	Sequential
Sequence type	Trufi
Bandwidth	898 Hz/Px

Sequence - Part 2

Define	Shots
Shots per slice	1
Segments	84
Trufi delta freq.	0 Hz
RF pulse type	Fast
Gradient mode	Fast
Excitation	Slice-sel.
Flip angle mode	Constant
Cine	Off

Sequence - Assistant

Mode	Off
Allowed delay	0 s

\\USER\Cardiac Research Protocols\Rapi-STRESS T1\Rapi-STRESS T1 (V2)\ShMOLLI_192i_d11_nFilt (TD=0)

TA: 7.1 s PM: FIX Voxel size: 0.9×0.9×8.0 mmPAT: 2 Rel. SNR: 1.00 : tfi

Properties

Prio recon	Off
Load images to viewer	On
Inline movie	Off
Auto store images	On
Load images to stamp segments	Off
Load images to graphic segments	On
Auto open inline display	Off
Auto close inline display	Off
Start measurement without further preparation	On
Wait for user to start	Off
Start measurements	Single measurement

Routine

Slice group	1
Slices	1
Dist. factor	25 %
Position	Isocenter
Orientation	Transversal
Phase enc. dir.	A >> P
AutoAlign	---
Phase oversampling	0 %
FoV read	360 mm
FoV phase	75.0 %
Slice thickness	8.0 mm
TR	378.98 ms
TE	1.07 ms
Averages	1
Concatenations	1
Filter	Raw filter, Distortion Corr.(2D)
Coil elements	BO2;SP2,3

Contrast - Common

TR	378.98 ms
TE	1.07 ms
Magn. preparation	Non-sel. IR
TI	260 ms
Flip angle	35 deg
Fat suppr.	None
Wrap-up Magn.	None

Contrast - Dynamic

Averages	1
Averaging mode	Short term
Reconstruction	Magn./Phase
Measurements	1
Multiple series	Off

Resolution - Common

FoV read	360 mm
FoV phase	75.0 %
Slice thickness	8.0 mm
Base resolution	192
Phase resolution	100 %
Phase partial Fourier	6/8
Trajectory	Cartesian
Interpolation	On

Resolution - iPAT

PAT mode	GRAPPA
Accel. factor PE	2
Ref. lines PE	24
Reference scan mode	Integrated

Resolution - Filter Image

Image Filter	Off
Distortion Corr.	On
Mode	2D
Unfiltered images	Off
Prescan Normalize	Off
Normalize	Off
B1 filter	Off

Resolution - Filter Rawdata

Raw filter	On
Elliptical filter	Off
POCS	Off

Geometry - Common

Slice group	1
Slices	1
Dist. factor	25 %
Position	Isocenter
Orientation	Transversal
Phase enc. dir.	A >> P
FoV read	360 mm
FoV phase	75.0 %
Slice thickness	8.0 mm
TR	378.98 ms
Multi-slice mode	Sequential
Series	Interleaved
Concatenations	1

Geometry - AutoAlign

Slice group	1
Position	Isocenter
Orientation	Transversal
Phase enc. dir.	A >> P
AutoAlign	---
Initial Position	Isocenter
Phase	0.0 mm
Read	0.0 mm
Shift	0.0 mm
Initial Rotation	0.00 deg
Initial Orientation	Transversal

Geometry - Saturation

Fat suppr.	None
Wrap-up Magn.	None
Special sat.	None

Geometry - Navigator

System - Miscellaneous

Positioning mode	FIX
Table position	H
Table position	0 mm

System - Miscellaneous

MSMA	S - C - T
Sagittal	R >> L
Coronal	A >> P
Transversal	F >> H
Coil Combine Mode	Adaptive Combine
Save uncombined	Off
Matrix Optimization	Off
Coil Focus	Flat
AutoAlign	---
Coil Select Mode	Default

System - Adjustments

B0 Shim mode	Cardiac
Adjust with body coil	On
Confirm freq. adjustment	Off
Assume Dominant Fat	Off
Assume Silicone	Off
Adjustment Tolerance	Auto

System - Adjust Volume

! Position	Isocenter
! Orientation	Transversal
! Rotation	0.00 deg
! A >> P	150 mm
! R >> L	150 mm
! F >> H	150 mm
Reset	Off

System - Tx/Rx

Frequency 1H	63.683250 MHz
Correction factor	1
Gain	High
Img. Scale Cor.	1.000
Reset	Off
? Ref. amplitude 1H	0.000 V

Physio - Signal1

1st Signal/Mode	ECG/Trigger
Average cycle	787 ± 129 ms
Average cycle	No Signal ms
Captured cycle	787 ± 129 ms
Acquisition window	379 ms
Trigger pulse	1
Trigger delay	0 ms
TR	378.98 ms
Concatenations	1
Segments	84
Phases	1
Adaptive Triggering	Off

Physio - Cardiac

Tagging	None
Magn. preparation	Non-sel. IR
TI	260 ms
Fat suppr.	None
Dark blood	Off
FoV read	360 mm
FoV phase	75.0 %
Phase resolution	100 %
Cine	Off
Trajectory	Cartesian
Dummy heartbeats	0
Motion Correction	None

Physio - PACE

Resp. control	Off
Concatenations	1

Sequence - Part 1

Introduction	Off
Dimension	2D
Reordering	Linear
Asymmetric echo	Weak
Contrasts	1
Optimization	Min. TE TR
Multi-slice mode	Sequential
Sequence type	Trufi
Bandwidth	898 Hz/Px

Sequence - Part 2

Define	Shots
Shots per slice	1
Segments	84
Trufi delta freq.	0 Hz
RF pulse type	Fast
Gradient mode	Fast
Excitation	Slice-sel.
Flip angle mode	Constant
Cine	Off

Sequence - Assistant

Mode	Off
Allowed delay	0 s

\\USER\Cardiac Research Protocols\Rapi-STRESS T1\Rapi-STRESS T1 (V2)\ShMOLLI_192i_d11_nFilt (TD=0)

TA: 7.1 s PM: FIX Voxel size: 0.9×0.9×8.0 mmPAT: 2 Rel. SNR: 1.00 : tfi

Properties

Prio recon	Off
Load images to viewer	On
Inline movie	Off
Auto store images	On
Load images to stamp segments	Off
Load images to graphic segments	On
Auto open inline display	Off
Auto close inline display	Off
Start measurement without further preparation	On
Wait for user to start	Off
Start measurements	Single measurement

Routine

Slice group	1
Slices	1
Dist. factor	25 %
Position	Isocenter
Orientation	Transversal
Phase enc. dir.	A >> P
AutoAlign	---
Phase oversampling	0 %
FoV read	360 mm
FoV phase	75.0 %
Slice thickness	8.0 mm
TR	378.98 ms
TE	1.07 ms
Averages	1
Concatenations	1
Filter	Raw filter, Distortion Corr.(2D)
Coil elements	BO2;SP2,3

Contrast - Common

TR	378.98 ms
TE	1.07 ms
Magn. preparation	Non-sel. IR
TI	260 ms
Flip angle	35 deg
Fat suppr.	None
Wrap-up Magn.	None

Contrast - Dynamic

Averages	1
Averaging mode	Short term
Reconstruction	Magn./Phase
Measurements	1
Multiple series	Off

Resolution - Common

FoV read	360 mm
FoV phase	75.0 %
Slice thickness	8.0 mm
Base resolution	192
Phase resolution	100 %
Phase partial Fourier	6/8
Trajectory	Cartesian
Interpolation	On

Resolution - iPAT

PAT mode	GRAPPA
Accel. factor PE	2
Ref. lines PE	24
Reference scan mode	Integrated

Resolution - Filter Image

Image Filter	Off
Distortion Corr.	On
Mode	2D
Unfiltered images	Off
Prescan Normalize	Off
Normalize	Off
B1 filter	Off

Resolution - Filter Rawdata

Raw filter	On
Elliptical filter	Off
POCS	Off

Geometry - Common

Slice group	1
Slices	1
Dist. factor	25 %
Position	Isocenter
Orientation	Transversal
Phase enc. dir.	A >> P
FoV read	360 mm
FoV phase	75.0 %
Slice thickness	8.0 mm
TR	378.98 ms
Multi-slice mode	Sequential
Series	Interleaved
Concatenations	1

Geometry - AutoAlign

Slice group	1
Position	Isocenter
Orientation	Transversal
Phase enc. dir.	A >> P
AutoAlign	---
Initial Position	Isocenter
Phase	0.0 mm
Read	0.0 mm
Shift	0.0 mm
Initial Rotation	0.00 deg
Initial Orientation	Transversal

Geometry - Saturation

Fat suppr.	None
Wrap-up Magn.	None
Special sat.	None

Geometry - Navigator

System - Miscellaneous

Positioning mode	FIX
Table position	H
Table position	0 mm

System - Miscellaneous

MSMA	S - C - T
Sagittal	R >> L
Coronal	A >> P
Transversal	F >> H
Coil Combine Mode	Adaptive Combine
Save uncombined	Off
Matrix Optimization	Off
Coil Focus	Flat
AutoAlign	---
Coil Select Mode	Default

System - Adjustments

B0 Shim mode	Cardiac
Adjust with body coil	On
Confirm freq. adjustment	Off
Assume Dominant Fat	Off
Assume Silicone	Off
Adjustment Tolerance	Auto

System - Adjust Volume

! Position	Isocenter
! Orientation	Transversal
! Rotation	0.00 deg
! A >> P	150 mm
! R >> L	150 mm
! F >> H	150 mm
Reset	Off

System - Tx/Rx

Frequency 1H	63.683250 MHz
Correction factor	1
Gain	High
Img. Scale Cor.	1.000
Reset	Off
? Ref. amplitude 1H	0.000 V

Physio - Signal1

1st Signal/Mode	ECG/Trigger
Average cycle	787 ± 129 ms
Average cycle	No Signal ms
Captured cycle	787 ± 129 ms
Acquisition window	379 ms
Trigger pulse	1
Trigger delay	0 ms
TR	378.98 ms
Concatenations	1
Segments	84
Phases	1
Adaptive Triggering	Off

Physio - Cardiac

Tagging	None
Magn. preparation	Non-sel. IR
TI	260 ms
Fat suppr.	None
Dark blood	Off
FoV read	360 mm
FoV phase	75.0 %
Phase resolution	100 %
Cine	Off
Trajectory	Cartesian
Dummy heartbeats	0
Motion Correction	None

Physio - PACE

Resp. control	Off
Concatenations	1

Sequence - Part 1

Introduction	Off
Dimension	2D
Reordering	Linear
Asymmetric echo	Weak
Contrasts	1
Optimization	Min. TE TR
Multi-slice mode	Sequential
Sequence type	Trufi
Bandwidth	898 Hz/Px

Sequence - Part 2

Define	Shots
Shots per slice	1
Segments	84
Trufi delta freq.	0 Hz
RF pulse type	Fast
Gradient mode	Fast
Excitation	Slice-sel.
Flip angle mode	Constant
Cine	Off

Sequence - Assistant

Mode	Off
Allowed delay	0 s

\\USER\Cardiac Research Protocols\Rapi-STRESS T1\Rapi-STRESS T1 (V2)\ShMOLLI_192i_d11_nFilt (TD=0)

TA: 7.1 s PM: FIX Voxel size: 0.9×0.9×8.0 mmPAT: 2 Rel. SNR: 1.00 : tfi

Properties

Prio recon	Off
Load images to viewer	On
Inline movie	Off
Auto store images	On
Load images to stamp segments	Off
Load images to graphic segments	On
Auto open inline display	Off
Auto close inline display	Off
Start measurement without further preparation	On
Wait for user to start	Off
Start measurements	Single measurement

Routine

Slice group	1
Slices	1
Dist. factor	25 %
Position	Isocenter
Orientation	Transversal
Phase enc. dir.	A >> P
AutoAlign	---
Phase oversampling	0 %
FoV read	360 mm
FoV phase	75.0 %
Slice thickness	8.0 mm
TR	378.98 ms
TE	1.07 ms
Averages	1
Concatenations	1
Filter	Raw filter, Distortion Corr.(2D)
Coil elements	BO2;SP2,3

Contrast - Common

TR	378.98 ms
TE	1.07 ms
Magn. preparation	Non-sel. IR
TI	260 ms
Flip angle	35 deg
Fat suppr.	None
Wrap-up Magn.	None

Contrast - Dynamic

Averages	1
Averaging mode	Short term
Reconstruction	Magn./Phase
Measurements	1
Multiple series	Off

Resolution - Common

FoV read	360 mm
FoV phase	75.0 %
Slice thickness	8.0 mm
Base resolution	192
Phase resolution	100 %
Phase partial Fourier	6/8
Trajectory	Cartesian
Interpolation	On

Resolution - iPAT

PAT mode	GRAPPA
Accel. factor PE	2
Ref. lines PE	24
Reference scan mode	Integrated

Resolution - Filter Image

Image Filter	Off
Distortion Corr.	On
Mode	2D
Unfiltered images	Off
Prescan Normalize	Off
Normalize	Off
B1 filter	Off

Resolution - Filter Rawdata

Raw filter	On
Elliptical filter	Off
POCS	Off

Geometry - Common

Slice group	1
Slices	1
Dist. factor	25 %
Position	Isocenter
Orientation	Transversal
Phase enc. dir.	A >> P
FoV read	360 mm
FoV phase	75.0 %
Slice thickness	8.0 mm
TR	378.98 ms
Multi-slice mode	Sequential
Series	Interleaved
Concatenations	1

Geometry - AutoAlign

Slice group	1
Position	Isocenter
Orientation	Transversal
Phase enc. dir.	A >> P
AutoAlign	---
Initial Position	Isocenter
Phase	0.0 mm
Read	0.0 mm
Shift	0.0 mm
Initial Rotation	0.00 deg
Initial Orientation	Transversal

Geometry - Saturation

Fat suppr.	None
Wrap-up Magn.	None
Special sat.	None

Geometry - Navigator

System - Miscellaneous

Positioning mode	FIX
Table position	H
Table position	0 mm

System - Miscellaneous

MSMA	S - C - T
Sagittal	R >> L
Coronal	A >> P
Transversal	F >> H
Coil Combine Mode	Adaptive Combine
Save uncombined	Off
Matrix Optimization	Off
Coil Focus	Flat
AutoAlign	---
Coil Select Mode	Default

System - Adjustments

B0 Shim mode	Cardiac
Adjust with body coil	On
Confirm freq. adjustment	Off
Assume Dominant Fat	Off
Assume Silicone	Off
Adjustment Tolerance	Auto

System - Adjust Volume

! Position	Isocenter
! Orientation	Transversal
! Rotation	0.00 deg
! A >> P	150 mm
! R >> L	150 mm
! F >> H	150 mm
Reset	Off

System - Tx/Rx

Frequency 1H	63.683250 MHz
Correction factor	1
Gain	High
Img. Scale Cor.	1.000
Reset	Off
? Ref. amplitude 1H	0.000 V

Physio - Signal1

1st Signal/Mode	ECG/Trigger
Average cycle	787 ± 129 ms
Average cycle	No Signal ms
Captured cycle	787 ± 129 ms
Acquisition window	379 ms
Trigger pulse	1
Trigger delay	0 ms
TR	378.98 ms
Concatenations	1
Segments	84
Phases	1
Adaptive Triggering	Off

Physio - Cardiac

Tagging	None
Magn. preparation	Non-sel. IR
TI	260 ms
Fat suppr.	None
Dark blood	Off
FoV read	360 mm
FoV phase	75.0 %
Phase resolution	100 %
Cine	Off
Trajectory	Cartesian
Dummy heartbeats	0
Motion Correction	None

Physio - PACE

Resp. control	Off
Concatenations	1

Sequence - Part 1

Introduction	Off
Dimension	2D
Reordering	Linear
Asymmetric echo	Weak
Contrasts	1
Optimization	Min. TE TR
Multi-slice mode	Sequential
Sequence type	Trufi
Bandwidth	898 Hz/Px

Sequence - Part 2

Define	Shots
Shots per slice	1
Segments	84
Trufi delta freq.	0 Hz
RF pulse type	Fast
Gradient mode	Fast
Excitation	Slice-sel.
Flip angle mode	Constant
Cine	Off

Sequence - Assistant

Mode	Off
Allowed delay	0 s

\\USER\Cardiac Research Protocols\Rapi-STRESS T1\Rapi-STRESS T1 (V2)\MidShMOLLI_192i_d11_nFilt (TD=0)

TA: 7.1 s PM: REF Voxel size: 0.9×0.9×8.0 mmPAT: 2 Rel. SNR: 1.00 : tfi

Properties

Prio recon	Off
Load images to viewer	On
Inline movie	Off
Auto store images	On
Load images to stamp segments	Off
Load images to graphic segments	On
Auto open inline display	Off
Auto close inline display	Off
Start measurement without further preparation	Off
Wait for user to start	Off
Start measurements	Single measurement

Routine

Slice group	1
Slices	1
Dist. factor	25 %
Position	Isocenter
Orientation	Transversal
Phase enc. dir.	A >> P
AutoAlign	---
Phase oversampling	0 %
FoV read	360 mm
FoV phase	75.0 %
Slice thickness	8.0 mm
TR	378.98 ms
TE	1.07 ms
Averages	1
Concatenations	1
Filter	Raw filter, Distortion Corr.(2D)
Coil elements	BO2;SP2,3

Contrast - Common

TR	378.98 ms
TE	1.07 ms
Magn. preparation	Non-sel. IR
TI	260 ms
Flip angle	35 deg
Fat suppr.	None
Wrap-up Magn.	None

Contrast - Dynamic

Averages	1
Averaging mode	Short term
Reconstruction	Magn./Phase
Measurements	1
Multiple series	Off

Resolution - Common

FoV read	360 mm
FoV phase	75.0 %
Slice thickness	8.0 mm
Base resolution	192
Phase resolution	100 %
Phase partial Fourier	6/8
Trajectory	Cartesian
Interpolation	On

Resolution - iPAT

PAT mode	GRAPPA
Accel. factor PE	2
Ref. lines PE	24
Reference scan mode	Integrated

Resolution - Filter Image

Image Filter	Off
Distortion Corr.	On
Mode	2D
Unfiltered images	Off
Prescan Normalize	Off
Normalize	Off
B1 filter	Off

Resolution - Filter Rawdata

Raw filter	On
Elliptical filter	Off
POCS	Off

Geometry - Common

Slice group	1
Slices	1
Dist. factor	25 %
Position	Isocenter
Orientation	Transversal
Phase enc. dir.	A >> P
FoV read	360 mm
FoV phase	75.0 %
Slice thickness	8.0 mm
TR	378.98 ms
Multi-slice mode	Sequential
Series	Interleaved
Concatenations	1

Geometry - AutoAlign

Slice group	1
Position	Isocenter
Orientation	Transversal
Phase enc. dir.	A >> P
AutoAlign	---
Initial Position	Isocenter
Phase	0.0 mm
Read	0.0 mm
Shift	0.0 mm
Initial Rotation	0.00 deg
Initial Orientation	Transversal

Geometry - Saturation

Fat suppr.	None
Wrap-up Magn.	None
Special sat.	None

Geometry - Navigator

System - Miscellaneous

Positioning mode	REF
Table position	H
Table position	0 mm

System - Miscellaneous

MSMA	S - C - T
Sagittal	R >> L
Coronal	A >> P
Transversal	F >> H
Coil Combine Mode	Adaptive Combine
Save uncombined	Off
Matrix Optimization	Off
Coil Focus	Flat
AutoAlign	---
Coil Select Mode	Default

System - Adjustments

B0 Shim mode	Cardiac
Adjust with body coil	On
Confirm freq. adjustment	Off
Assume Dominant Fat	Off
Assume Silicone	Off
Adjustment Tolerance	Auto

System - Adjust Volume

! Position	Isocenter
! Orientation	Transversal
! Rotation	0.00 deg
! A >> P	150 mm
! R >> L	150 mm
! F >> H	150 mm
Reset	Off

System - Tx/Rx

Frequency 1H	63.683250 MHz
Correction factor	1
Gain	High
Img. Scale Cor.	1.000
Reset	Off
? Ref. amplitude 1H	0.000 V

Physio - Signal1

1st Signal/Mode	ECG/Trigger
Average cycle	787 ± 129 ms
Average cycle	No Signal ms
Captured cycle	787 ± 129 ms
Acquisition window	379 ms
Trigger pulse	1
Trigger delay	0 ms
TR	378.98 ms
Concatenations	1
Segments	84
Phases	1
Adaptive Triggering	Off

Physio - Cardiac

Tagging	None
Magn. preparation	Non-sel. IR
TI	260 ms
Fat suppr.	None
Dark blood	Off
FoV read	360 mm
FoV phase	75.0 %
Phase resolution	100 %
Cine	Off
Trajectory	Cartesian
Dummy heartbeats	0
Motion Correction	None

Physio - PACE

Resp. control	Off
Concatenations	1

Sequence - Part 1

Introduction	Off
Dimension	2D
Reordering	Linear
Asymmetric echo	Weak
Contrasts	1
Optimization	Min. TE TR
Multi-slice mode	Sequential
Sequence type	Trufi
Bandwidth	898 Hz/Px

Sequence - Part 2

Define	Shots
Shots per slice	1
Segments	84
Trufi delta freq.	0 Hz
RF pulse type	Fast
Gradient mode	Fast
Excitation	Slice-sel.
Flip angle mode	Constant
Cine	Off

Sequence - Assistant

Mode	Off
Allowed delay	0 s

\\USER\Cardiac Research Protocols\Rapi-STRESS T1\Rapi-STRESS T1 (V2)\T1Map_LongT1 (TD=0)LowHR

TA: 8.7 s PM: FIX Voxel size: 1.4×1.4×8.0 mmPAT: 2 Rel. SNR: 1.00 : tfi

Properties

Prio recon	Off
Load images to viewer	On
Inline movie	Off
Auto store images	On
Load images to stamp segments	On
Load images to graphic segments	On
Auto open inline display	Off
Auto close inline display	Off
Start measurement without further preparation	On
Wait for user to start	Off
Start measurements	Single measurement

Routine

Slice group	1
Slices	1
Dist. factor	25 %
Position	Isocenter
Orientation	Transversal
Phase enc. dir.	A >> P
AutoAlign	---
Phase oversampling	0 %
FoV read	360 mm
FoV phase	85.2 %
Slice thickness	8.0 mm
TR	279.84 ms
TE	1.13 ms
Averages	1
Concatenations	1
Filter	Distortion Corr.(2D)
Coil elements	BO2;SP2,3

Contrast - Common

TR	279.84 ms
TE	1.13 ms
Magn. preparation	Non-sel. IR T1map
TI	180 ms
Flip angle	35 deg
Fat suppr.	None
Wrap-up Magn.	None

Contrast - Dynamic

Averages	1
Averaging mode	Short term
Reconstruction	Magnitude
Measurements	1
Multiple series	Off

Resolution - Common

FoV read	360 mm
FoV phase	85.2 %
Slice thickness	8.0 mm
Base resolution	256
Phase resolution	66 %
Phase partial Fourier	7/8
Trajectory	Cartesian
Interpolation	Off

Resolution - iPAT

PAT mode	GRAPPA
Accel. factor PE	2
Ref. lines PE	36
Reference scan mode	GRE/separate

Resolution - Filter Image

Image Filter	Off
Distortion Corr.	On
Mode	2D
Unfiltered images	Off
Prescan Normalize	Off
Normalize	Off
B1 filter	Off

Resolution - Filter Rawdata

Raw filter	Off
Elliptical filter	Off
POCS	Off

Geometry - Common

Slice group	1
Slices	1
Dist. factor	25 %
Position	Isocenter
Orientation	Transversal
Phase enc. dir.	A >> P
FoV read	360 mm
FoV phase	85.2 %
Slice thickness	8.0 mm
TR	279.84 ms
Multi-slice mode	Sequential
Series	Base To Apex
Concatenations	1

Geometry - AutoAlign

Slice group	1
Position	Isocenter
Orientation	Transversal
Phase enc. dir.	A >> P
AutoAlign	---
Initial Position	Isocenter
Phase	0.0 mm
Read	0.0 mm
Shift	0.0 mm
Initial Rotation	0.00 deg
Initial Orientation	Transversal

Geometry - Saturation

Fat suppr.	None
Wrap-up Magn.	None
Special sat.	None

Geometry - Navigator

System - Miscellaneous

Positioning mode	FIX
Table position	H
Table position	0 mm

System - Miscellaneous

MSMA	S - C - T
Sagittal	R >> L
Coronal	A >> P
Transversal	F >> H
Coil Combine Mode	Adaptive Combine
Save uncombined	Off
Matrix Optimization	Off
Coil Focus	Flat
AutoAlign	---
Coil Select Mode	Default

System - Adjustments

B0 Shim mode	Cardiac
Adjust with body coil	Off
Confirm freq. adjustment	Off
Assume Dominant Fat	Off
Assume Silicone	Off
Adjustment Tolerance	Auto

System - Adjust Volume

! Position	Isocenter
! Orientation	Transversal
! Rotation	0.00 deg
! A >> P	150 mm
! R >> L	150 mm
! F >> H	150 mm
Reset	Off

System - Tx/Rx

Frequency 1H	63.683250 MHz
Correction factor	1
Gain	High
Img. Scale Cor.	1.000
Reset	Off
? Ref. amplitude 1H	0.000 V

Physio - Signal1

1st Signal/Mode	ECG/Trigger
Average cycle	787 ± 129 ms
Average cycle	No Signal ms
Captured cycle	787 ± 129 ms
Acquisition window	280 ms
Trigger pulse	1
Trigger delay	0 ms
TR	279.84 ms
Concatenations	1
Segments	72
Phases	1
Adaptive Triggering	Off

Physio - Cardiac

Tagging	None
Magn. preparation	Non-sel. IR T1map
TI	180 ms
Fat suppr.	None
Dark blood	Off
FoV read	360 mm
FoV phase	85.2 %
Phase resolution	66 %
Cine	Off
Trajectory	Cartesian
Dummy heartbeats	0
Motion Correction	Standard

Physio - PACE

Resp. control	Breath-hold
Concatenations	1

Sequence - Part 1

Introduction	Off
Dimension	2D
Reordering	Linear
Asymmetric echo	Weak
Contrasts	1
Optimization	Min. TE TR
Multi-slice mode	Sequential
Sequence type	Trufi
Bandwidth	1085 Hz/Px

Sequence - Part 2

Define	Shots
Shots per slice	1
Segments	72
Trufi delta freq.	0 Hz
RF pulse type	Fast
Gradient mode	Fast
Excitation	Slice-sel.
Flip angle mode	Constant
Cine	Off

Sequence - Assistant

Mode	Off
Allowed delay	0 s

\\USER\Cardiac Research Protocols\Rapi-STRESS T1\Rapi-STRESS T1 (V2)\ShMOLLI_192i_d11_nFilt (TD=0)

TA: 7.1 s PM: FIX Voxel size: 0.9×0.9×8.0 mmPAT: 2 Rel. SNR: 1.00 : tfi

Properties

Prio recon	Off
Load images to viewer	On
Inline movie	Off
Auto store images	On
Load images to stamp segments	Off
Load images to graphic segments	On
Auto open inline display	Off
Auto close inline display	Off
Start measurement without further preparation	On
Wait for user to start	Off
Start measurements	Single measurement

Routine

Slice group	1
Slices	1
Dist. factor	25 %
Position	Isocenter
Orientation	Transversal
Phase enc. dir.	A >> P
AutoAlign	---
Phase oversampling	0 %
FoV read	360 mm
FoV phase	75.0 %
Slice thickness	8.0 mm
TR	378.98 ms
TE	1.07 ms
Averages	1
Concatenations	1
Filter	Raw filter, Distortion Corr.(2D)
Coil elements	BO2;SP2,3

Contrast - Common

TR	378.98 ms
TE	1.07 ms
Magn. preparation	Non-sel. IR
TI	260 ms
Flip angle	35 deg
Fat suppr.	None
Wrap-up Magn.	None

Contrast - Dynamic

Averages	1
Averaging mode	Short term
Reconstruction	Magn./Phase
Measurements	1
Multiple series	Off

Resolution - Common

FoV read	360 mm
FoV phase	75.0 %
Slice thickness	8.0 mm
Base resolution	192
Phase resolution	100 %
Phase partial Fourier	6/8
Trajectory	Cartesian
Interpolation	On

Resolution - iPAT

PAT mode	GRAPPA
Accel. factor PE	2
Ref. lines PE	24
Reference scan mode	Integrated

Resolution - Filter Image

Image Filter	Off
Distortion Corr.	On
Mode	2D
Unfiltered images	Off
Prescan Normalize	Off
Normalize	Off
B1 filter	Off

Resolution - Filter Rawdata

Raw filter	On
Elliptical filter	Off
POCS	Off

Geometry - Common

Slice group	1
Slices	1
Dist. factor	25 %
Position	Isocenter
Orientation	Transversal
Phase enc. dir.	A >> P
FoV read	360 mm
FoV phase	75.0 %
Slice thickness	8.0 mm
TR	378.98 ms
Multi-slice mode	Sequential
Series	Interleaved
Concatenations	1

Geometry - AutoAlign

Slice group	1
Position	Isocenter
Orientation	Transversal
Phase enc. dir.	A >> P
AutoAlign	---
Initial Position	Isocenter
Phase	0.0 mm
Read	0.0 mm
Shift	0.0 mm
Initial Rotation	0.00 deg
Initial Orientation	Transversal

Geometry - Saturation

Fat suppr.	None
Wrap-up Magn.	None
Special sat.	None

Geometry - Navigator

System - Miscellaneous

Positioning mode	FIX
Table position	H
Table position	0 mm

System - Miscellaneous

MSMA	S - C - T
Sagittal	R >> L
Coronal	A >> P
Transversal	F >> H
Coil Combine Mode	Adaptive Combine
Save uncombined	Off
Matrix Optimization	Off
Coil Focus	Flat
AutoAlign	---
Coil Select Mode	Default

System - Adjustments

B0 Shim mode	Cardiac
Adjust with body coil	On
Confirm freq. adjustment	Off
Assume Dominant Fat	Off
Assume Silicone	Off
Adjustment Tolerance	Auto

System - Adjust Volume

! Position	Isocenter
! Orientation	Transversal
! Rotation	0.00 deg
! A >> P	150 mm
! R >> L	150 mm
! F >> H	150 mm
Reset	Off

System - Tx/Rx

Frequency 1H	63.683250 MHz
Correction factor	1
Gain	High
Img. Scale Cor.	1.000
Reset	Off
? Ref. amplitude 1H	0.000 V

Physio - Signal1

1st Signal/Mode	ECG/Trigger
Average cycle	787 ± 129 ms
Average cycle	No Signal ms
Captured cycle	787 ± 129 ms
Acquisition window	379 ms
Trigger pulse	1
Trigger delay	0 ms
TR	378.98 ms
Concatenations	1
Segments	84
Phases	1
Adaptive Triggering	Off

Physio - Cardiac

Tagging	None
Magn. preparation	Non-sel. IR
TI	260 ms
Fat suppr.	None
Dark blood	Off
FoV read	360 mm
FoV phase	75.0 %
Phase resolution	100 %
Cine	Off
Trajectory	Cartesian
Dummy heartbeats	0
Motion Correction	None

Physio - PACE

Resp. control	Off
Concatenations	1

Sequence - Part 1

Introduction	Off
Dimension	2D
Reordering	Linear
Asymmetric echo	Weak
Contrasts	1
Optimization	Min. TE TR
Multi-slice mode	Sequential
Sequence type	Trufi
Bandwidth	898 Hz/Px

Sequence - Part 2

Define	Shots
Shots per slice	1
Segments	84
Trufi delta freq.	0 Hz
RF pulse type	Fast
Gradient mode	Fast
Excitation	Slice-sel.
Flip angle mode	Constant
Cine	Off

Sequence - Assistant

Mode	Off
Allowed delay	0 s

\\USER\Cardiac Research Protocols\Rapi-STRESS T1\Rapi-STRESS T1 (V2)\T1Map_LongT1_TD0_HighHR

TA: 8.7 s PM: FIX Voxel size: 1.9×1.9×8.0 mmPAT: 2 Rel. SNR: 1.00 : tfi

Properties

Prio recon	Off
Load images to viewer	On
Inline movie	Off
Auto store images	On
Load images to stamp segments	On
Load images to graphic segments	On
Auto open inline display	Off
Auto close inline display	Off
Start measurement without further preparation	On
Wait for user to start	Off
Start measurements	Single measurement

Routine

Slice group	1
Slices	1
Dist. factor	20 %
Position	Isocenter
Orientation	Transversal
Phase enc. dir.	A >> P
AutoAlign	---
Phase oversampling	0 %
FoV read	360 mm
FoV phase	85.4 %
Slice thickness	8.0 mm
TR	263.55 ms
TE	1.01 ms
Averages	1
Concatenations	1
Filter	Distortion Corr.(2D)
Coil elements	BO2;SP2,3

Contrast - Common

TR	263.55 ms
TE	1.01 ms
Magn. preparation	Non-sel. IR T1map
TI	180 ms
Flip angle	35 deg
Fat suppr.	None
Wrap-up Magn.	None

Contrast - Dynamic

Averages	1
Averaging mode	Short term
Reconstruction	Magnitude
Measurements	1
Multiple series	Off

Resolution - Common

FoV read	360 mm
FoV phase	85.4 %
Slice thickness	8.0 mm
Base resolution	192
Phase resolution	80 %
Phase partial Fourier	7/8
Trajectory	Cartesian
Interpolation	Off

Resolution - iPAT

PAT mode	GRAPPA
Accel. factor PE	2
Ref. lines PE	36
Reference scan mode	GRE/separate

Resolution - Filter Image

Image Filter	Off
Distortion Corr.	On
Mode	2D
Unfiltered images	Off
Prescan Normalize	Off
Normalize	Off
B1 filter	Off

Resolution - Filter Rawdata

Raw filter	Off
Elliptical filter	Off
POCS	Off

Geometry - Common

Slice group	1
Slices	1
Dist. factor	20 %
Position	Isocenter
Orientation	Transversal
Phase enc. dir.	A >> P
FoV read	360 mm
FoV phase	85.4 %
Slice thickness	8.0 mm
TR	263.55 ms
Multi-slice mode	Sequential
Series	Base To Apex
Concatenations	1

Geometry - AutoAlign

Slice group	1
Position	Isocenter
Orientation	Transversal
Phase enc. dir.	A >> P
AutoAlign	---
Initial Position	Isocenter
Phase	0.0 mm
Read	0.0 mm
Shift	0.0 mm
Initial Rotation	0.00 deg
Initial Orientation	Transversal

Geometry - Saturation

Fat suppr.	None
Wrap-up Magn.	None
Special sat.	None

Geometry - Navigator

System - Miscellaneous

Positioning mode	FIX
Table position	H
Table position	0 mm

System - Miscellaneous

MSMA	S - C - T
Sagittal	R >> L
Coronal	A >> P
Transversal	F >> H
Coil Combine Mode	Adaptive Combine
Save uncombined	Off
Matrix Optimization	Off
Coil Focus	Flat
AutoAlign	---
Coil Select Mode	Default

System - Adjustments

B0 Shim mode	Cardiac
Adjust with body coil	Off
Confirm freq. adjustment	Off
Assume Dominant Fat	Off
Assume Silicone	Off
Adjustment Tolerance	Auto

System - Adjust Volume

! Position	Isocenter
! Orientation	Transversal
! Rotation	0.00 deg
! A >> P	150 mm
! R >> L	150 mm
! F >> H	150 mm
Reset	Off

System - Tx/Rx

Frequency 1H	63.683250 MHz
Correction factor	1
Gain	High
Img. Scale Cor.	1.000
Reset	Off
? Ref. amplitude 1H	0.000 V

Physio - Signal1

1st Signal/Mode	ECG/Trigger
Average cycle	787 ± 129 ms
Average cycle	No Signal ms
Captured cycle	787 ± 129 ms
Acquisition window	700 ms
Trigger pulse	1
Trigger delay	0 ms
TR	263.55 ms
Concatenations	1
Segments	66
Phases	1
Adaptive Triggering	Off

Physio - Cardiac

Tagging	None
Magn. preparation	Non-sel. IR T1map
TI	180 ms
Fat suppr.	None
Dark blood	Off
FoV read	360 mm
FoV phase	85.4 %
Phase resolution	80 %
Cine	Off
Trajectory	Cartesian
Dummy heartbeats	0
Motion Correction	Standard

Physio - PACE

Resp. control	Breath-hold
Concatenations	1

Sequence - Part 1

Introduction	Off
Dimension	2D
Reordering	Linear
Asymmetric echo	Weak
Contrasts	1
Optimization	Min. TE TR
Multi-slice mode	Sequential
Sequence type	Trufi
Bandwidth	1085 Hz/Px

Sequence - Part 2

Define	Shots
Shots per slice	1
Segments	66
Trufi delta freq.	0 Hz
RF pulse type	Fast
Gradient mode	Fast
Excitation	Slice-sel.
Flip angle mode	Constant
Cine	Off

Sequence - Assistant

Mode	Off
Allowed delay	0 s

\\USER\Cardiac Research Protocols\Rapi-STRESS T1\Rapi-STRESS T1 (V2)\ShMOLLI_192i_d11_nFilt (TD=0)

TA: 7.1 s PM: FIX Voxel size: 0.9×0.9×8.0 mmPAT: 2 Rel. SNR: 1.00 : tfi

Properties

Prio recon	Off
Load images to viewer	On
Inline movie	Off
Auto store images	On
Load images to stamp segments	Off
Load images to graphic segments	On
Auto open inline display	Off
Auto close inline display	Off
Start measurement without further preparation	On
Wait for user to start	Off
Start measurements	Single measurement

Routine

Slice group	1
Slices	1
Dist. factor	25 %
Position	Isocenter
Orientation	Transversal
Phase enc. dir.	A >> P
AutoAlign	---
Phase oversampling	0 %
FoV read	360 mm
FoV phase	75.0 %
Slice thickness	8.0 mm
TR	378.98 ms
TE	1.07 ms
Averages	1
Concatenations	1
Filter	Raw filter, Distortion Corr.(2D)
Coil elements	BO2;SP2,3

Contrast - Common

TR	378.98 ms
TE	1.07 ms
Magn. preparation	Non-sel. IR
TI	260 ms
Flip angle	35 deg
Fat suppr.	None
Wrap-up Magn.	None

Contrast - Dynamic

Averages	1
Averaging mode	Short term
Reconstruction	Magn./Phase
Measurements	1
Multiple series	Off

Resolution - Common

FoV read	360 mm
FoV phase	75.0 %
Slice thickness	8.0 mm
Base resolution	192
Phase resolution	100 %
Phase partial Fourier	6/8
Trajectory	Cartesian
Interpolation	On

Resolution - iPAT

PAT mode	GRAPPA
Accel. factor PE	2
Ref. lines PE	24
Reference scan mode	Integrated

Resolution - Filter Image

Image Filter	Off
Distortion Corr.	On
Mode	2D
Unfiltered images	Off
Prescan Normalize	Off
Normalize	Off
B1 filter	Off

Resolution - Filter Rawdata

Raw filter	On
Elliptical filter	Off
POCS	Off

Geometry - Common

Slice group	1
Slices	1
Dist. factor	25 %
Position	Isocenter
Orientation	Transversal
Phase enc. dir.	A >> P
FoV read	360 mm
FoV phase	75.0 %
Slice thickness	8.0 mm
TR	378.98 ms
Multi-slice mode	Sequential
Series	Interleaved
Concatenations	1

Geometry - AutoAlign

Slice group	1
Position	Isocenter
Orientation	Transversal
Phase enc. dir.	A >> P
AutoAlign	---
Initial Position	Isocenter
Phase	0.0 mm
Read	0.0 mm
Shift	0.0 mm
Initial Rotation	0.00 deg
Initial Orientation	Transversal

Geometry - Saturation

Fat suppr.	None
Wrap-up Magn.	None
Special sat.	None

Geometry - Navigator

System - Miscellaneous

Positioning mode	FIX
Table position	H
Table position	0 mm

System - Miscellaneous

MSMA	S - C - T
Sagittal	R >> L
Coronal	A >> P
Transversal	F >> H
Coil Combine Mode	Adaptive Combine
Save uncombined	Off
Matrix Optimization	Off
Coil Focus	Flat
AutoAlign	---
Coil Select Mode	Default

System - Adjustments

B0 Shim mode	Cardiac
Adjust with body coil	On
Confirm freq. adjustment	Off
Assume Dominant Fat	Off
Assume Silicone	Off
Adjustment Tolerance	Auto

System - Adjust Volume

! Position	Isocenter
! Orientation	Transversal
! Rotation	0.00 deg
! A >> P	150 mm
! R >> L	150 mm
! F >> H	150 mm
Reset	Off

System - Tx/Rx

Frequency 1H	63.683250 MHz
Correction factor	1
Gain	High
Img. Scale Cor.	1.000
Reset	Off
? Ref. amplitude 1H	0.000 V

Physio - Signal1

1st Signal/Mode	ECG/Trigger
Average cycle	787 ± 129 ms
Average cycle	No Signal ms
Captured cycle	787 ± 129 ms
Acquisition window	379 ms
Trigger pulse	1
Trigger delay	0 ms
TR	378.98 ms
Concatenations	1
Segments	84
Phases	1
Adaptive Triggering	Off

Physio - Cardiac

Tagging	None
Magn. preparation	Non-sel. IR
TI	260 ms
Fat suppr.	None
Dark blood	Off
FoV read	360 mm
FoV phase	75.0 %
Phase resolution	100 %
Cine	Off
Trajectory	Cartesian
Dummy heartbeats	0
Motion Correction	None

Physio - PACE

Resp. control	Off
Concatenations	1

Sequence - Part 1

Introduction	Off
Dimension	2D
Reordering	Linear
Asymmetric echo	Weak
Contrasts	1
Optimization	Min. TE TR
Multi-slice mode	Sequential
Sequence type	Trufi
Bandwidth	898 Hz/Px

Sequence - Part 2

Define	Shots
Shots per slice	1
Segments	84
Trufi delta freq.	0 Hz
RF pulse type	Fast
Gradient mode	Fast
Excitation	Slice-sel.
Flip angle mode	Constant
Cine	Off

Sequence - Assistant

Mode	Off
Allowed delay	0 s

\\USER\Cardiac Research Protocols\Rapi-STRESS T1\Rapi-STRESS T1 (V2)\MidShMOLLI_192i_d11_nFilt (TD=0)

TA: 7.1 s PM: REF Voxel size: 0.9×0.9×8.0 mmPAT: 2 Rel. SNR: 1.00 : tfi

Properties

Prio recon	Off
Load images to viewer	On
Inline movie	Off
Auto store images	On
Load images to stamp segments	Off
Load images to graphic segments	On
Auto open inline display	Off
Auto close inline display	Off
Start measurement without further preparation	Off
Wait for user to start	Off
Start measurements	Single measurement

Routine

Slice group	1
Slices	1
Dist. factor	25 %
Position	Isocenter
Orientation	Transversal
Phase enc. dir.	A >> P
AutoAlign	---
Phase oversampling	0 %
FoV read	360 mm
FoV phase	75.0 %
Slice thickness	8.0 mm
TR	378.98 ms
TE	1.07 ms
Averages	1
Concatenations	1
Filter	Raw filter, Distortion Corr.(2D)
Coil elements	BO2;SP2,3

Contrast - Common

TR	378.98 ms
TE	1.07 ms
Magn. preparation	Non-sel. IR
TI	260 ms
Flip angle	35 deg
Fat suppr.	None
Wrap-up Magn.	None

Contrast - Dynamic

Averages	1
Averaging mode	Short term
Reconstruction	Magn./Phase
Measurements	1
Multiple series	Off

Resolution - Common

FoV read	360 mm
FoV phase	75.0 %
Slice thickness	8.0 mm
Base resolution	192
Phase resolution	100 %
Phase partial Fourier	6/8
Trajectory	Cartesian
Interpolation	On

Resolution - iPAT

PAT mode	GRAPPA
Accel. factor PE	2
Ref. lines PE	24
Reference scan mode	Integrated

Resolution - Filter Image

Image Filter	Off
Distortion Corr.	On
Mode	2D
Unfiltered images	Off
Prescan Normalize	Off
Normalize	Off
B1 filter	Off

Resolution - Filter Rawdata

Raw filter	On
Elliptical filter	Off
POCS	Off

Geometry - Common

Slice group	1
Slices	1
Dist. factor	25 %
Position	Isocenter
Orientation	Transversal
Phase enc. dir.	A >> P
FoV read	360 mm
FoV phase	75.0 %
Slice thickness	8.0 mm
TR	378.98 ms
Multi-slice mode	Sequential
Series	Interleaved
Concatenations	1

Geometry - AutoAlign

Slice group	1
Position	Isocenter
Orientation	Transversal
Phase enc. dir.	A >> P
AutoAlign	---
Initial Position	Isocenter
Phase	0.0 mm
Read	0.0 mm
Shift	0.0 mm
Initial Rotation	0.00 deg
Initial Orientation	Transversal

Geometry - Saturation

Fat suppr.	None
Wrap-up Magn.	None
Special sat.	None

Geometry - Navigator

System - Miscellaneous

Positioning mode	REF
Table position	H
Table position	0 mm

System - Miscellaneous

MSMA	S - C - T
Sagittal	R >> L
Coronal	A >> P
Transversal	F >> H
Coil Combine Mode	Adaptive Combine
Save uncombined	Off
Matrix Optimization	Off
Coil Focus	Flat
AutoAlign	---
Coil Select Mode	Default

System - Adjustments

B0 Shim mode	Cardiac
Adjust with body coil	On
Confirm freq. adjustment	Off
Assume Dominant Fat	Off
Assume Silicone	Off
Adjustment Tolerance	Auto

System - Adjust Volume

! Position	Isocenter
! Orientation	Transversal
! Rotation	0.00 deg
! A >> P	150 mm
! R >> L	150 mm
! F >> H	150 mm
Reset	Off

System - Tx/Rx

Frequency 1H	63.683250 MHz
Correction factor	1
Gain	High
Img. Scale Cor.	1.000
Reset	Off
? Ref. amplitude 1H	0.000 V

Physio - Signal1

1st Signal/Mode	ECG/Trigger
Average cycle	787 ± 129 ms
Average cycle	No Signal ms
Captured cycle	787 ± 129 ms
Acquisition window	379 ms
Trigger pulse	1
Trigger delay	0 ms
TR	378.98 ms
Concatenations	1
Segments	84
Phases	1
Adaptive Triggering	Off

Physio - Cardiac

Tagging	None
Magn. preparation	Non-sel. IR
TI	260 ms
Fat suppr.	None
Dark blood	Off
FoV read	360 mm
FoV phase	75.0 %
Phase resolution	100 %
Cine	Off
Trajectory	Cartesian
Dummy heartbeats	0
Motion Correction	None

Physio - PACE

Resp. control	Off
Concatenations	1

Sequence - Part 1

Introduction	Off
Dimension	2D
Reordering	Linear
Asymmetric echo	Weak
Contrasts	1
Optimization	Min. TE TR
Multi-slice mode	Sequential
Sequence type	Trufi
Bandwidth	898 Hz/Px

Sequence - Part 2

Define	Shots
Shots per slice	1
Segments	84
Trufi delta freq.	0 Hz
RF pulse type	Fast
Gradient mode	Fast
Excitation	Slice-sel.
Flip angle mode	Constant
Cine	Off

Sequence - Assistant

Mode	Off
Allowed delay	0 s

\\USER\Cardiac Research Protocols\Rapi-STRESS T1\Rapi-STRESS T1 (V2)\T1Map_LongT1 (TD=0)LowHR

TA: 8.7 s PM: FIX Voxel size: 1.4×1.4×8.0 mmPAT: 2 Rel. SNR: 1.00 : tfi

Properties

Prio recon	Off
Load images to viewer	On
Inline movie	Off
Auto store images	On
Load images to stamp segments	On
Load images to graphic segments	On
Auto open inline display	Off
Auto close inline display	Off
Start measurement without further preparation	On
Wait for user to start	Off
Start measurements	Single measurement

Routine

Slice group	1
Slices	1
Dist. factor	25 %
Position	Isocenter
Orientation	Transversal
Phase enc. dir.	A >> P
AutoAlign	---
Phase oversampling	0 %
FoV read	360 mm
FoV phase	85.2 %
Slice thickness	8.0 mm
TR	279.84 ms
TE	1.13 ms
Averages	1
Concatenations	1
Filter	Distortion Corr.(2D)
Coil elements	BO2;SP2,3

Contrast - Common

TR	279.84 ms
TE	1.13 ms
Magn. preparation	Non-sel. IR T1map
TI	180 ms
Flip angle	35 deg
Fat suppr.	None
Wrap-up Magn.	None

Contrast - Dynamic

Averages	1
Averaging mode	Short term
Reconstruction	Magnitude
Measurements	1
Multiple series	Off

Resolution - Common

FoV read	360 mm
FoV phase	85.2 %
Slice thickness	8.0 mm
Base resolution	256
Phase resolution	66 %
Phase partial Fourier	7/8
Trajectory	Cartesian
Interpolation	Off

Resolution - iPAT

PAT mode	GRAPPA
Accel. factor PE	2
Ref. lines PE	36
Reference scan mode	GRE/separate

Resolution - Filter Image

Image Filter	Off
Distortion Corr.	On
Mode	2D
Unfiltered images	Off
Prescan Normalize	Off
Normalize	Off
B1 filter	Off

Resolution - Filter Rawdata

Raw filter	Off
Elliptical filter	Off
POCS	Off

Geometry - Common

Slice group	1
Slices	1
Dist. factor	25 %
Position	Isocenter
Orientation	Transversal
Phase enc. dir.	A >> P
FoV read	360 mm
FoV phase	85.2 %
Slice thickness	8.0 mm
TR	279.84 ms
Multi-slice mode	Sequential
Series	Base To Apex
Concatenations	1

Geometry - AutoAlign

Slice group	1
Position	Isocenter
Orientation	Transversal
Phase enc. dir.	A >> P
AutoAlign	---
Initial Position	Isocenter
Phase	0.0 mm
Read	0.0 mm
Shift	0.0 mm
Initial Rotation	0.00 deg
Initial Orientation	Transversal

Geometry - Saturation

Fat suppr.	None
Wrap-up Magn.	None
Special sat.	None

Geometry - Navigator

System - Miscellaneous

Positioning mode	FIX
Table position	H
Table position	0 mm

System - Miscellaneous

MSMA	S - C - T
Sagittal	R >> L
Coronal	A >> P
Transversal	F >> H
Coil Combine Mode	Adaptive Combine
Save uncombined	Off
Matrix Optimization	Off
Coil Focus	Flat
AutoAlign	---
Coil Select Mode	Default

System - Adjustments

B0 Shim mode	Cardiac
Adjust with body coil	Off
Confirm freq. adjustment	Off
Assume Dominant Fat	Off
Assume Silicone	Off
Adjustment Tolerance	Auto

System - Adjust Volume

! Position	Isocenter
! Orientation	Transversal
! Rotation	0.00 deg
! A >> P	150 mm
! R >> L	150 mm
! F >> H	150 mm
Reset	Off

System - Tx/Rx

Frequency 1H	63.683250 MHz
Correction factor	1
Gain	High
Img. Scale Cor.	1.000
Reset	Off
? Ref. amplitude 1H	0.000 V

Physio - Signal1

1st Signal/Mode	ECG/Trigger
Average cycle	787 ± 129 ms
Average cycle	No Signal ms
Captured cycle	787 ± 129 ms
Acquisition window	280 ms
Trigger pulse	1
Trigger delay	0 ms
TR	279.84 ms
Concatenations	1
Segments	72
Phases	1
Adaptive Triggering	Off

Physio - Cardiac

Tagging	None
Magn. preparation	Non-sel. IR T1map
TI	180 ms
Fat suppr.	None
Dark blood	Off
FoV read	360 mm
FoV phase	85.2 %
Phase resolution	66 %
Cine	Off
Trajectory	Cartesian
Dummy heartbeats	0
Motion Correction	Standard

Physio - PACE

Resp. control	Breath-hold
Concatenations	1

Sequence - Part 1

Introduction	Off
Dimension	2D
Reordering	Linear
Asymmetric echo	Weak
Contrasts	1
Optimization	Min. TE TR
Multi-slice mode	Sequential
Sequence type	Trufi
Bandwidth	1085 Hz/Px

Sequence - Part 2

Define	Shots
Shots per slice	1
Segments	72
Trufi delta freq.	0 Hz
RF pulse type	Fast
Gradient mode	Fast
Excitation	Slice-sel.
Flip angle mode	Constant
Cine	Off

Sequence - Assistant

Mode	Off
Allowed delay	0 s

\\USER\Cardiac Research Protocols\Rapi-STRESS T1\Rapi-STRESS T1 (V2)\ShMOLLI_192i_d11_nFilt (TD=0)

TA: 7.1 s PM: FIX Voxel size: 0.9×0.9×8.0 mmPAT: 2 Rel. SNR: 1.00 : tfi

Properties

Prio recon	Off
Load images to viewer	On
Inline movie	Off
Auto store images	On
Load images to stamp segments	Off
Load images to graphic segments	On
Auto open inline display	Off
Auto close inline display	Off
Start measurement without further preparation	On
Wait for user to start	Off
Start measurements	Single measurement

Routine

Slice group	1
Slices	1
Dist. factor	25 %
Position	Isocenter
Orientation	Transversal
Phase enc. dir.	A >> P
AutoAlign	---
Phase oversampling	0 %
FoV read	360 mm
FoV phase	75.0 %
Slice thickness	8.0 mm
TR	378.98 ms
TE	1.07 ms
Averages	1
Concatenations	1
Filter	Raw filter, Distortion Corr.(2D)
Coil elements	BO2;SP2,3

Contrast - Common

TR	378.98 ms
TE	1.07 ms
Magn. preparation	Non-sel. IR
TI	260 ms
Flip angle	35 deg
Fat suppr.	None
Wrap-up Magn.	None

Contrast - Dynamic

Averages	1
Averaging mode	Short term
Reconstruction	Magn./Phase
Measurements	1
Multiple series	Off

Resolution - Common

FoV read	360 mm
FoV phase	75.0 %
Slice thickness	8.0 mm
Base resolution	192
Phase resolution	100 %
Phase partial Fourier	6/8
Trajectory	Cartesian
Interpolation	On

Resolution - iPAT

PAT mode	GRAPPA
Accel. factor PE	2
Ref. lines PE	24
Reference scan mode	Integrated

Resolution - Filter Image

Image Filter	Off
Distortion Corr.	On
Mode	2D
Unfiltered images	Off
Prescan Normalize	Off
Normalize	Off
B1 filter	Off

Resolution - Filter Rawdata

Raw filter	On
Elliptical filter	Off
POCS	Off

Geometry - Common

Slice group	1
Slices	1
Dist. factor	25 %
Position	Isocenter
Orientation	Transversal
Phase enc. dir.	A >> P
FoV read	360 mm
FoV phase	75.0 %
Slice thickness	8.0 mm
TR	378.98 ms
Multi-slice mode	Sequential
Series	Interleaved
Concatenations	1

Geometry - AutoAlign

Slice group	1
Position	Isocenter
Orientation	Transversal
Phase enc. dir.	A >> P
AutoAlign	---
Initial Position	Isocenter
Phase	0.0 mm
Read	0.0 mm
Shift	0.0 mm
Initial Rotation	0.00 deg
Initial Orientation	Transversal

Geometry - Saturation

Fat suppr.	None
Wrap-up Magn.	None
Special sat.	None

Geometry - Navigator

System - Miscellaneous

Positioning mode	FIX
Table position	H
Table position	0 mm

System - Miscellaneous

MSMA	S - C - T
Sagittal	R >> L
Coronal	A >> P
Transversal	F >> H
Coil Combine Mode	Adaptive Combine
Save uncombined	Off
Matrix Optimization	Off
Coil Focus	Flat
AutoAlign	---
Coil Select Mode	Default

System - Adjustments

B0 Shim mode	Cardiac
Adjust with body coil	On
Confirm freq. adjustment	Off
Assume Dominant Fat	Off
Assume Silicone	Off
Adjustment Tolerance	Auto

System - Adjust Volume

! Position	Isocenter
! Orientation	Transversal
! Rotation	0.00 deg
! A >> P	150 mm
! R >> L	150 mm
! F >> H	150 mm
Reset	Off

System - Tx/Rx

Frequency 1H	63.683250 MHz
Correction factor	1
Gain	High
Img. Scale Cor.	1.000
Reset	Off
? Ref. amplitude 1H	0.000 V

Physio - Signal1

1st Signal/Mode	ECG/Trigger
Average cycle	787 ± 129 ms
Average cycle	No Signal ms
Captured cycle	787 ± 129 ms
Acquisition window	379 ms
Trigger pulse	1
Trigger delay	0 ms
TR	378.98 ms
Concatenations	1
Segments	84
Phases	1
Adaptive Triggering	Off

Physio - Cardiac

Tagging	None
Magn. preparation	Non-sel. IR
TI	260 ms
Fat suppr.	None
Dark blood	Off
FoV read	360 mm
FoV phase	75.0 %
Phase resolution	100 %
Cine	Off
Trajectory	Cartesian
Dummy heartbeats	0
Motion Correction	None

Physio - PACE

Resp. control	Off
Concatenations	1

Sequence - Part 1

Introduction	Off
Dimension	2D
Reordering	Linear
Asymmetric echo	Weak
Contrasts	1
Optimization	Min. TE TR
Multi-slice mode	Sequential
Sequence type	Trufi
Bandwidth	898 Hz/Px

Sequence - Part 2

Define	Shots
Shots per slice	1
Segments	84
Trufi delta freq.	0 Hz
RF pulse type	Fast
Gradient mode	Fast
Excitation	Slice-sel.
Flip angle mode	Constant
Cine	Off

Sequence - Assistant

Mode	Off
Allowed delay	0 s

\\USER\Cardiac Research Protocols\Rapi-STRESS T1\Rapi-STRESS T1 (V2)\T1Map_LongT1_TD0_HighHR

TA: 8.7 s PM: FIX Voxel size: 1.9×1.9×8.0 mmPAT: 2 Rel. SNR: 1.00 : tfi

Properties

Prio recon	Off
Load images to viewer	On
Inline movie	Off
Auto store images	On
Load images to stamp segments	On
Load images to graphic segments	On
Auto open inline display	Off
Auto close inline display	Off
Start measurement without further preparation	On
Wait for user to start	Off
Start measurements	Single measurement

Routine

Slice group	1
Slices	1
Dist. factor	20 %
Position	Isocenter
Orientation	Transversal
Phase enc. dir.	A >> P
AutoAlign	---
Phase oversampling	0 %
FoV read	360 mm
FoV phase	85.4 %
Slice thickness	8.0 mm
TR	263.55 ms
TE	1.01 ms
Averages	1
Concatenations	1
Filter	Distortion Corr.(2D)
Coil elements	BO2;SP2,3

Contrast - Common

TR	263.55 ms
TE	1.01 ms
Magn. preparation	Non-sel. IR T1map
TI	180 ms
Flip angle	35 deg
Fat suppr.	None
Wrap-up Magn.	None

Contrast - Dynamic

Averages	1
Averaging mode	Short term
Reconstruction	Magnitude
Measurements	1
Multiple series	Off

Resolution - Common

FoV read	360 mm
FoV phase	85.4 %
Slice thickness	8.0 mm
Base resolution	192
Phase resolution	80 %
Phase partial Fourier	7/8
Trajectory	Cartesian
Interpolation	Off

Resolution - iPAT

PAT mode	GRAPPA
Accel. factor PE	2
Ref. lines PE	36
Reference scan mode	GRE/separate

Resolution - Filter Image

Image Filter	Off
Distortion Corr.	On
Mode	2D
Unfiltered images	Off
Prescan Normalize	Off
Normalize	Off
B1 filter	Off

Resolution - Filter Rawdata

Raw filter	Off
Elliptical filter	Off
POCS	Off

Geometry - Common

Slice group	1
Slices	1
Dist. factor	20 %
Position	Isocenter
Orientation	Transversal
Phase enc. dir.	A >> P
FoV read	360 mm
FoV phase	85.4 %
Slice thickness	8.0 mm
TR	263.55 ms
Multi-slice mode	Sequential
Series	Base To Apex
Concatenations	1

Geometry - AutoAlign

Slice group	1
Position	Isocenter
Orientation	Transversal
Phase enc. dir.	A >> P
AutoAlign	---
Initial Position	Isocenter
Phase	0.0 mm
Read	0.0 mm
Shift	0.0 mm
Initial Rotation	0.00 deg
Initial Orientation	Transversal

Geometry - Saturation

Fat suppr.	None
Wrap-up Magn.	None
Special sat.	None

Geometry - Navigator

System - Miscellaneous

Positioning mode	FIX
Table position	H
Table position	0 mm

System - Miscellaneous

MSMA	S - C - T
Sagittal	R >> L
Coronal	A >> P
Transversal	F >> H
Coil Combine Mode	Adaptive Combine
Save uncombined	Off
Matrix Optimization	Off
Coil Focus	Flat
AutoAlign	---
Coil Select Mode	Default

System - Adjustments

B0 Shim mode	Cardiac
Adjust with body coil	Off
Confirm freq. adjustment	Off
Assume Dominant Fat	Off
Assume Silicone	Off
Adjustment Tolerance	Auto

System - Adjust Volume

! Position	Isocenter
! Orientation	Transversal
! Rotation	0.00 deg
! A >> P	150 mm
! R >> L	150 mm
! F >> H	150 mm
Reset	Off

System - Tx/Rx

Frequency 1H	63.683250 MHz
Correction factor	1
Gain	High
Img. Scale Cor.	1.000
Reset	Off
? Ref. amplitude 1H	0.000 V

Physio - Signal1

1st Signal/Mode	ECG/Trigger
Average cycle	787 ± 129 ms
Average cycle	No Signal ms
Captured cycle	787 ± 129 ms
Acquisition window	700 ms
Trigger pulse	1
Trigger delay	0 ms
TR	263.55 ms
Concatenations	1
Segments	66
Phases	1
Adaptive Triggering	Off

Physio - Cardiac

Tagging	None
Magn. preparation	Non-sel. IR T1map
TI	180 ms
Fat suppr.	None
Dark blood	Off
FoV read	360 mm
FoV phase	85.4 %
Phase resolution	80 %
Cine	Off
Trajectory	Cartesian
Dummy heartbeats	0
Motion Correction	Standard

Physio - PACE

Resp. control	Breath-hold
Concatenations	1

Sequence - Part 1

Introduction	Off
Dimension	2D
Reordering	Linear
Asymmetric echo	Weak
Contrasts	1
Optimization	Min. TE TR
Multi-slice mode	Sequential
Sequence type	Trufi
Bandwidth	1085 Hz/Px

Sequence - Part 2

Define	Shots
Shots per slice	1
Segments	66
Trufi delta freq.	0 Hz
RF pulse type	Fast
Gradient mode	Fast
Excitation	Slice-sel.
Flip angle mode	Constant
Cine	Off

Sequence - Assistant

Mode	Off
Allowed delay	0 s

\\USER\Cardiac Research Protocols\Rapi-STRESS T1\Rapi-STRESS T1 (V2)\ShMOLLI_192i_d11_nFilt (TD=0)

TA: 7.1 s PM: FIX Voxel size: 0.9×0.9×8.0 mmPAT: 2 Rel. SNR: 1.00 : tfi

Properties

Prio recon	Off
Load images to viewer	On
Inline movie	Off
Auto store images	On
Load images to stamp segments	Off
Load images to graphic segments	On
Auto open inline display	Off
Auto close inline display	Off
Start measurement without further preparation	On
Wait for user to start	Off
Start measurements	Single measurement

Routine

Slice group	1
Slices	1
Dist. factor	25 %
Position	Isocenter
Orientation	Transversal
Phase enc. dir.	A >> P
AutoAlign	---
Phase oversampling	0 %
FoV read	360 mm
FoV phase	75.0 %
Slice thickness	8.0 mm
TR	378.98 ms
TE	1.07 ms
Averages	1
Concatenations	1
Filter	Raw filter, Distortion Corr.(2D)
Coil elements	BO2;SP2,3

Contrast - Common

TR	378.98 ms
TE	1.07 ms
Magn. preparation	Non-sel. IR
TI	260 ms
Flip angle	35 deg
Fat suppr.	None
Wrap-up Magn.	None

Contrast - Dynamic

Averages	1
Averaging mode	Short term
Reconstruction	Magn./Phase
Measurements	1
Multiple series	Off

Resolution - Common

FoV read	360 mm
FoV phase	75.0 %
Slice thickness	8.0 mm
Base resolution	192
Phase resolution	100 %
Phase partial Fourier	6/8
Trajectory	Cartesian
Interpolation	On

Resolution - iPAT

PAT mode	GRAPPA
Accel. factor PE	2
Ref. lines PE	24
Reference scan mode	Integrated

Resolution - Filter Image

Image Filter	Off
Distortion Corr.	On
Mode	2D
Unfiltered images	Off
Prescan Normalize	Off
Normalize	Off
B1 filter	Off

Resolution - Filter Rawdata

Raw filter	On
Elliptical filter	Off
POCS	Off

Geometry - Common

Slice group	1
Slices	1
Dist. factor	25 %
Position	Isocenter
Orientation	Transversal
Phase enc. dir.	A >> P
FoV read	360 mm
FoV phase	75.0 %
Slice thickness	8.0 mm
TR	378.98 ms
Multi-slice mode	Sequential
Series	Interleaved
Concatenations	1

Geometry - AutoAlign

Slice group	1
Position	Isocenter
Orientation	Transversal
Phase enc. dir.	A >> P
AutoAlign	---
Initial Position	Isocenter
Phase	0.0 mm
Read	0.0 mm
Shift	0.0 mm
Initial Rotation	0.00 deg
Initial Orientation	Transversal

Geometry - Saturation

Fat suppr.	None
Wrap-up Magn.	None
Special sat.	None

Geometry - Navigator

System - Miscellaneous

Positioning mode	FIX
Table position	H
Table position	0 mm

System - Miscellaneous

MSMA	S - C - T
Sagittal	R >> L
Coronal	A >> P
Transversal	F >> H
Coil Combine Mode	Adaptive Combine
Save uncombined	Off
Matrix Optimization	Off
Coil Focus	Flat
AutoAlign	---
Coil Select Mode	Default

System - Adjustments

B0 Shim mode	Cardiac
Adjust with body coil	On
Confirm freq. adjustment	Off
Assume Dominant Fat	Off
Assume Silicone	Off
Adjustment Tolerance	Auto

System - Adjust Volume

! Position	Isocenter
! Orientation	Transversal
! Rotation	0.00 deg
! A >> P	150 mm
! R >> L	150 mm
! F >> H	150 mm
Reset	Off

System - Tx/Rx

Frequency 1H	63.683250 MHz
Correction factor	1
Gain	High
Img. Scale Cor.	1.000
Reset	Off
? Ref. amplitude 1H	0.000 V

Physio - Signal1

1st Signal/Mode	ECG/Trigger
Average cycle	787 ± 129 ms
Average cycle	No Signal ms
Captured cycle	787 ± 129 ms
Acquisition window	379 ms
Trigger pulse	1
Trigger delay	0 ms
TR	378.98 ms
Concatenations	1
Segments	84
Phases	1
Adaptive Triggering	Off

Physio - Cardiac

Tagging	None
Magn. preparation	Non-sel. IR
TI	260 ms
Fat suppr.	None
Dark blood	Off
FoV read	360 mm
FoV phase	75.0 %
Phase resolution	100 %
Cine	Off
Trajectory	Cartesian
Dummy heartbeats	0
Motion Correction	None

Physio - PACE

Resp. control	Off
Concatenations	1

Sequence - Part 1

Introduction	Off
Dimension	2D
Reordering	Linear
Asymmetric echo	Weak
Contrasts	1
Optimization	Min. TE TR
Multi-slice mode	Sequential
Sequence type	Trufi
Bandwidth	898 Hz/Px

Sequence - Part 2

Define	Shots
Shots per slice	1
Segments	84
Trufi delta freq.	0 Hz
RF pulse type	Fast
Gradient mode	Fast
Excitation	Slice-sel.
Flip angle mode	Constant
Cine	Off

Sequence - Assistant

Mode	Off
Allowed delay	0 s

\\USER\Cardiac Research Protocols\Rapi-STRESS T1\Rapi-STRESS T1 (V2)\MidShMOLLI_192i_d11_nFilt (TD=0)

TA: 7.1 s PM: REF Voxel size: 0.9×0.9×8.0 mmPAT: 2 Rel. SNR: 1.00 : tfi

Properties

Prio recon	Off
Load images to viewer	On
Inline movie	Off
Auto store images	On
Load images to stamp segments	Off
Load images to graphic segments	On
Auto open inline display	Off
Auto close inline display	Off
Start measurement without further preparation	Off
Wait for user to start	Off
Start measurements	Single measurement

Routine

Slice group	1
Slices	1
Dist. factor	25 %
Position	Isocenter
Orientation	Transversal
Phase enc. dir.	A >> P
AutoAlign	---
Phase oversampling	0 %
FoV read	360 mm
FoV phase	75.0 %
Slice thickness	8.0 mm
TR	378.98 ms
TE	1.07 ms
Averages	1
Concatenations	1
Filter	Raw filter, Distortion Corr.(2D)
Coil elements	BO2;SP2,3

Contrast - Common

TR	378.98 ms
TE	1.07 ms
Magn. preparation	Non-sel. IR
TI	260 ms
Flip angle	35 deg
Fat suppr.	None
Wrap-up Magn.	None

Contrast - Dynamic

Averages	1
Averaging mode	Short term
Reconstruction	Magn./Phase
Measurements	1
Multiple series	Off

Resolution - Common

FoV read	360 mm
FoV phase	75.0 %
Slice thickness	8.0 mm
Base resolution	192
Phase resolution	100 %
Phase partial Fourier	6/8
Trajectory	Cartesian
Interpolation	On

Resolution - iPAT

PAT mode	GRAPPA
Accel. factor PE	2
Ref. lines PE	24
Reference scan mode	Integrated

Resolution - Filter Image

Image Filter	Off
Distortion Corr.	On
Mode	2D
Unfiltered images	Off
Prescan Normalize	Off
Normalize	Off
B1 filter	Off

Resolution - Filter Rawdata

Raw filter	On
Elliptical filter	Off
POCS	Off

Geometry - Common

Slice group	1
Slices	1
Dist. factor	25 %
Position	Isocenter
Orientation	Transversal
Phase enc. dir.	A >> P
FoV read	360 mm
FoV phase	75.0 %
Slice thickness	8.0 mm
TR	378.98 ms
Multi-slice mode	Sequential
Series	Interleaved
Concatenations	1

Geometry - AutoAlign

Slice group	1
Position	Isocenter
Orientation	Transversal
Phase enc. dir.	A >> P
AutoAlign	---
Initial Position	Isocenter
Phase	0.0 mm
Read	0.0 mm
Shift	0.0 mm
Initial Rotation	0.00 deg
Initial Orientation	Transversal

Geometry - Saturation

Fat suppr.	None
Wrap-up Magn.	None
Special sat.	None

Geometry - Navigator

System - Miscellaneous

Positioning mode	REF
Table position	H
Table position	0 mm

System - Miscellaneous

MSMA	S - C - T
Sagittal	R >> L
Coronal	A >> P
Transversal	F >> H
Coil Combine Mode	Adaptive Combine
Save uncombined	Off
Matrix Optimization	Off
Coil Focus	Flat
AutoAlign	---
Coil Select Mode	Default

System - Adjustments

B0 Shim mode	Cardiac
Adjust with body coil	On
Confirm freq. adjustment	Off
Assume Dominant Fat	Off
Assume Silicone	Off
Adjustment Tolerance	Auto

System - Adjust Volume

! Position	Isocenter
! Orientation	Transversal
! Rotation	0.00 deg
! A >> P	150 mm
! R >> L	150 mm
! F >> H	150 mm
Reset	Off

System - Tx/Rx

Frequency 1H	63.683250 MHz
Correction factor	1
Gain	High
Img. Scale Cor.	1.000
Reset	Off
? Ref. amplitude 1H	0.000 V

Physio - Signal1

1st Signal/Mode	ECG/Trigger
Average cycle	787 ± 129 ms
Average cycle	No Signal ms
Captured cycle	787 ± 129 ms
Acquisition window	379 ms
Trigger pulse	1
Trigger delay	0 ms
TR	378.98 ms
Concatenations	1
Segments	84
Phases	1
Adaptive Triggering	Off

Physio - Cardiac

Tagging	None
Magn. preparation	Non-sel. IR
TI	260 ms
Fat suppr.	None
Dark blood	Off
FoV read	360 mm
FoV phase	75.0 %
Phase resolution	100 %
Cine	Off
Trajectory	Cartesian
Dummy heartbeats	0
Motion Correction	None

Physio - PACE

Resp. control	Off
Concatenations	1

Sequence - Part 1

Introduction	Off
Dimension	2D
Reordering	Linear
Asymmetric echo	Weak
Contrasts	1
Optimization	Min. TE TR
Multi-slice mode	Sequential
Sequence type	Trufi
Bandwidth	898 Hz/Px

Sequence - Part 2

Define	Shots
Shots per slice	1
Segments	84
Trufi delta freq.	0 Hz
RF pulse type	Fast
Gradient mode	Fast
Excitation	Slice-sel.
Flip angle mode	Constant
Cine	Off

Sequence - Assistant

Mode	Off
Allowed delay	0 s

\\USER\Cardiac Research Protocols\Rapi-STRESS T1\Rapi-STRESS T1 (V2)\T1Map_LongT1 (TD=0)LowHR

TA: 8.7 s PM: FIX Voxel size: 1.4×1.4×8.0 mmPAT: 2 Rel. SNR: 1.00 : tfi

Properties

Prio recon	Off
Load images to viewer	On
Inline movie	Off
Auto store images	On
Load images to stamp segments	On
Load images to graphic segments	On
Auto open inline display	Off
Auto close inline display	Off
Start measurement without further preparation	On
Wait for user to start	Off
Start measurements	Single measurement

Routine

Slice group	1
Slices	1
Dist. factor	25 %
Position	Isocenter
Orientation	Transversal
Phase enc. dir.	A >> P
AutoAlign	---
Phase oversampling	0 %
FoV read	360 mm
FoV phase	85.2 %
Slice thickness	8.0 mm
TR	279.84 ms
TE	1.13 ms
Averages	1
Concatenations	1
Filter	Distortion Corr.(2D)
Coil elements	BO2;SP2,3

Contrast - Common

TR	279.84 ms
TE	1.13 ms
Magn. preparation	Non-sel. IR T1map
TI	180 ms
Flip angle	35 deg
Fat suppr.	None
Wrap-up Magn.	None

Contrast - Dynamic

Averages	1
Averaging mode	Short term
Reconstruction	Magnitude
Measurements	1
Multiple series	Off

Resolution - Common

FoV read	360 mm
FoV phase	85.2 %
Slice thickness	8.0 mm
Base resolution	256
Phase resolution	66 %
Phase partial Fourier	7/8
Trajectory	Cartesian
Interpolation	Off

Resolution - iPAT

PAT mode	GRAPPA
Accel. factor PE	2
Ref. lines PE	36
Reference scan mode	GRE/separate

Resolution - Filter Image

Image Filter	Off
Distortion Corr.	On
Mode	2D
Unfiltered images	Off
Prescan Normalize	Off
Normalize	Off
B1 filter	Off

Resolution - Filter Rawdata

Raw filter	Off
Elliptical filter	Off
POCS	Off

Geometry - Common

Slice group	1
Slices	1
Dist. factor	25 %
Position	Isocenter
Orientation	Transversal
Phase enc. dir.	A >> P
FoV read	360 mm
FoV phase	85.2 %
Slice thickness	8.0 mm
TR	279.84 ms
Multi-slice mode	Sequential
Series	Base To Apex
Concatenations	1

Geometry - AutoAlign

Slice group	1
Position	Isocenter
Orientation	Transversal
Phase enc. dir.	A >> P
AutoAlign	---
Initial Position	Isocenter
Phase	0.0 mm
Read	0.0 mm
Shift	0.0 mm
Initial Rotation	0.00 deg
Initial Orientation	Transversal

Geometry - Saturation

Fat suppr.	None
Wrap-up Magn.	None
Special sat.	None

Geometry - Navigator

System - Miscellaneous

Positioning mode	FIX
Table position	H
Table position	0 mm

System - Miscellaneous

MSMA	S - C - T
Sagittal	R >> L
Coronal	A >> P
Transversal	F >> H
Coil Combine Mode	Adaptive Combine
Save uncombined	Off
Matrix Optimization	Off
Coil Focus	Flat
AutoAlign	---
Coil Select Mode	Default

System - Adjustments

B0 Shim mode	Cardiac
Adjust with body coil	Off
Confirm freq. adjustment	Off
Assume Dominant Fat	Off
Assume Silicone	Off
Adjustment Tolerance	Auto

System - Adjust Volume

! Position	Isocenter
! Orientation	Transversal
! Rotation	0.00 deg
! A >> P	150 mm
! R >> L	150 mm
! F >> H	150 mm
Reset	Off

System - Tx/Rx

Frequency 1H	63.683250 MHz
Correction factor	1
Gain	High
Img. Scale Cor.	1.000
Reset	Off
? Ref. amplitude 1H	0.000 V

Physio - Signal1

1st Signal/Mode	ECG/Trigger
Average cycle	787 ± 129 ms
Average cycle	No Signal ms
Captured cycle	787 ± 129 ms
Acquisition window	280 ms
Trigger pulse	1
Trigger delay	0 ms
TR	279.84 ms
Concatenations	1
Segments	72
Phases	1
Adaptive Triggering	Off

Physio - Cardiac

Tagging	None
Magn. preparation	Non-sel. IR T1map
TI	180 ms
Fat suppr.	None
Dark blood	Off
FoV read	360 mm
FoV phase	85.2 %
Phase resolution	66 %
Cine	Off
Trajectory	Cartesian
Dummy heartbeats	0
Motion Correction	Standard

Physio - PACE

Resp. control	Breath-hold
Concatenations	1

Sequence - Part 1

Introduction	Off
Dimension	2D
Reordering	Linear
Asymmetric echo	Weak
Contrasts	1
Optimization	Min. TE TR
Multi-slice mode	Sequential
Sequence type	Trufi
Bandwidth	1085 Hz/Px

Sequence - Part 2

Define	Shots
Shots per slice	1
Segments	72
Trufi delta freq.	0 Hz
RF pulse type	Fast
Gradient mode	Fast
Excitation	Slice-sel.
Flip angle mode	Constant
Cine	Off

Sequence - Assistant

Mode	Off
Allowed delay	0 s

\\USER\Cardiac Research Protocols\Rapi-STRESS T1\Rapi-STRESS T1 (V2)\ShMOLLI_192i_d11_nFilt (TD=0)

TA: 7.1 s PM: FIX Voxel size: 0.9×0.9×8.0 mmPAT: 2 Rel. SNR: 1.00 : tfi

Properties

Prio recon	Off
Load images to viewer	On
Inline movie	Off
Auto store images	On
Load images to stamp segments	Off
Load images to graphic segments	On
Auto open inline display	Off
Auto close inline display	Off
Start measurement without further preparation	On
Wait for user to start	Off
Start measurements	Single measurement

Routine

Slice group	1
Slices	1
Dist. factor	25 %
Position	Isocenter
Orientation	Transversal
Phase enc. dir.	A >> P
AutoAlign	---
Phase oversampling	0 %
FoV read	360 mm
FoV phase	75.0 %
Slice thickness	8.0 mm
TR	378.98 ms
TE	1.07 ms
Averages	1
Concatenations	1
Filter	Raw filter, Distortion Corr.(2D)
Coil elements	BO2;SP2,3

Contrast - Common

TR	378.98 ms
TE	1.07 ms
Magn. preparation	Non-sel. IR
TI	260 ms
Flip angle	35 deg
Fat suppr.	None
Wrap-up Magn.	None

Contrast - Dynamic

Averages	1
Averaging mode	Short term
Reconstruction	Magn./Phase
Measurements	1
Multiple series	Off

Resolution - Common

FoV read	360 mm
FoV phase	75.0 %
Slice thickness	8.0 mm
Base resolution	192
Phase resolution	100 %
Phase partial Fourier	6/8
Trajectory	Cartesian
Interpolation	On

Resolution - iPAT

PAT mode	GRAPPA
Accel. factor PE	2
Ref. lines PE	24
Reference scan mode	Integrated

Resolution - Filter Image

Image Filter	Off
Distortion Corr.	On
Mode	2D
Unfiltered images	Off
Prescan Normalize	Off
Normalize	Off
B1 filter	Off

Resolution - Filter Rawdata

Raw filter	On
Elliptical filter	Off
POCS	Off

Geometry - Common

Slice group	1
Slices	1
Dist. factor	25 %
Position	Isocenter
Orientation	Transversal
Phase enc. dir.	A >> P
FoV read	360 mm
FoV phase	75.0 %
Slice thickness	8.0 mm
TR	378.98 ms
Multi-slice mode	Sequential
Series	Interleaved
Concatenations	1

Geometry - AutoAlign

Slice group	1
Position	Isocenter
Orientation	Transversal
Phase enc. dir.	A >> P
AutoAlign	---
Initial Position	Isocenter
Phase	0.0 mm
Read	0.0 mm
Shift	0.0 mm
Initial Rotation	0.00 deg
Initial Orientation	Transversal

Geometry - Saturation

Fat suppr.	None
Wrap-up Magn.	None
Special sat.	None

Geometry - Navigator

System - Miscellaneous

Positioning mode	FIX
Table position	H
Table position	0 mm

System - Miscellaneous

MSMA	S - C - T
Sagittal	R >> L
Coronal	A >> P
Transversal	F >> H
Coil Combine Mode	Adaptive Combine
Save uncombined	Off
Matrix Optimization	Off
Coil Focus	Flat
AutoAlign	---
Coil Select Mode	Default

System - Adjustments

B0 Shim mode	Cardiac
Adjust with body coil	On
Confirm freq. adjustment	Off
Assume Dominant Fat	Off
Assume Silicone	Off
Adjustment Tolerance	Auto

System - Adjust Volume

! Position	Isocenter
! Orientation	Transversal
! Rotation	0.00 deg
! A >> P	150 mm
! R >> L	150 mm
! F >> H	150 mm
Reset	Off

System - Tx/Rx

Frequency 1H	63.683250 MHz
Correction factor	1
Gain	High
Img. Scale Cor.	1.000
Reset	Off
? Ref. amplitude 1H	0.000 V

Physio - Signal1

1st Signal/Mode	ECG/Trigger
Average cycle	787 ± 129 ms
Average cycle	No Signal ms
Captured cycle	787 ± 129 ms
Acquisition window	379 ms
Trigger pulse	1
Trigger delay	0 ms
TR	378.98 ms
Concatenations	1
Segments	84
Phases	1
Adaptive Triggering	Off

Physio - Cardiac

Tagging	None
Magn. preparation	Non-sel. IR
TI	260 ms
Fat suppr.	None
Dark blood	Off
FoV read	360 mm
FoV phase	75.0 %
Phase resolution	100 %
Cine	Off
Trajectory	Cartesian
Dummy heartbeats	0
Motion Correction	None

Physio - PACE

Resp. control	Off
Concatenations	1

Sequence - Part 1

Introduction	Off
Dimension	2D
Reordering	Linear
Asymmetric echo	Weak
Contrasts	1
Optimization	Min. TE TR
Multi-slice mode	Sequential
Sequence type	Trufi
Bandwidth	898 Hz/Px

Sequence - Part 2

Define	Shots
Shots per slice	1
Segments	84
Trufi delta freq.	0 Hz
RF pulse type	Fast
Gradient mode	Fast
Excitation	Slice-sel.
Flip angle mode	Constant
Cine	Off

Sequence - Assistant

Mode	Off
Allowed delay	0 s

\\USER\Cardiac Research Protocols\Rapi-STRESS T1\Rapi-STRESS T1 (V2)\T1Map_LongT1_TD0_HighHR

TA: 8.7 s PM: FIX Voxel size: 1.9×1.9×8.0 mmPAT: 2 Rel. SNR: 1.00 : tfi

Properties

Prio recon	Off
Load images to viewer	On
Inline movie	Off
Auto store images	On
Load images to stamp segments	On
Load images to graphic segments	On
Auto open inline display	Off
Auto close inline display	Off
Start measurement without further preparation	On
Wait for user to start	Off
Start measurements	Single measurement

Routine

Slice group	1
Slices	1
Dist. factor	20 %
Position	Isocenter
Orientation	Transversal
Phase enc. dir.	A >> P
AutoAlign	---
Phase oversampling	0 %
FoV read	360 mm
FoV phase	85.4 %
Slice thickness	8.0 mm
TR	263.55 ms
TE	1.01 ms
Averages	1
Concatenations	1
Filter	Distortion Corr.(2D)
Coil elements	BO2;SP2,3

Contrast - Common

TR	263.55 ms
TE	1.01 ms
Magn. preparation	Non-sel. IR T1map
TI	180 ms
Flip angle	35 deg
Fat suppr.	None
Wrap-up Magn.	None

Contrast - Dynamic

Averages	1
Averaging mode	Short term
Reconstruction	Magnitude
Measurements	1
Multiple series	Off

Resolution - Common

FoV read	360 mm
FoV phase	85.4 %
Slice thickness	8.0 mm
Base resolution	192
Phase resolution	80 %
Phase partial Fourier	7/8
Trajectory	Cartesian
Interpolation	Off

Resolution - iPAT

PAT mode	GRAPPA
Accel. factor PE	2
Ref. lines PE	36
Reference scan mode	GRE/separate

Resolution - Filter Image

Image Filter	Off
Distortion Corr.	On
Mode	2D
Unfiltered images	Off
Prescan Normalize	Off
Normalize	Off
B1 filter	Off

Resolution - Filter Rawdata

Raw filter	Off
Elliptical filter	Off
POCS	Off

Geometry - Common

Slice group	1
Slices	1
Dist. factor	20 %
Position	Isocenter
Orientation	Transversal
Phase enc. dir.	A >> P
FoV read	360 mm
FoV phase	85.4 %
Slice thickness	8.0 mm
TR	263.55 ms
Multi-slice mode	Sequential
Series	Base To Apex
Concatenations	1

Geometry - AutoAlign

Slice group	1
Position	Isocenter
Orientation	Transversal
Phase enc. dir.	A >> P
AutoAlign	---
Initial Position	Isocenter
Phase	0.0 mm
Read	0.0 mm
Shift	0.0 mm
Initial Rotation	0.00 deg
Initial Orientation	Transversal

Geometry - Saturation

Fat suppr.	None
Wrap-up Magn.	None
Special sat.	None

Geometry - Navigator

System - Miscellaneous

Positioning mode	FIX
Table position	H
Table position	0 mm

System - Miscellaneous

MSMA	S - C - T
Sagittal	R >> L
Coronal	A >> P
Transversal	F >> H
Coil Combine Mode	Adaptive Combine
Save uncombined	Off
Matrix Optimization	Off
Coil Focus	Flat
AutoAlign	---
Coil Select Mode	Default

System - Adjustments

B0 Shim mode	Cardiac
Adjust with body coil	Off
Confirm freq. adjustment	Off
Assume Dominant Fat	Off
Assume Silicone	Off
Adjustment Tolerance	Auto

System - Adjust Volume

! Position	Isocenter
! Orientation	Transversal
! Rotation	0.00 deg
! A >> P	150 mm
! R >> L	150 mm
! F >> H	150 mm
Reset	Off

System - Tx/Rx

Frequency 1H	63.683250 MHz
Correction factor	1
Gain	High
Img. Scale Cor.	1.000
Reset	Off
? Ref. amplitude 1H	0.000 V

Physio - Signal1

1st Signal/Mode	ECG/Trigger
Average cycle	787 ± 129 ms
Average cycle	No Signal ms
Captured cycle	787 ± 129 ms
Acquisition window	700 ms
Trigger pulse	1
Trigger delay	0 ms
TR	263.55 ms
Concatenations	1
Segments	66
Phases	1
Adaptive Triggering	Off

Physio - Cardiac

Tagging	None
Magn. preparation	Non-sel. IR T1map
TI	180 ms
Fat suppr.	None
Dark blood	Off
FoV read	360 mm
FoV phase	85.4 %
Phase resolution	80 %
Cine	Off
Trajectory	Cartesian
Dummy heartbeats	0
Motion Correction	Standard

Physio - PACE

Resp. control	Breath-hold
Concatenations	1

Sequence - Part 1

Introduction	Off
Dimension	2D
Reordering	Linear
Asymmetric echo	Weak
Contrasts	1
Optimization	Min. TE TR
Multi-slice mode	Sequential
Sequence type	Trufi
Bandwidth	1085 Hz/Px

Sequence - Part 2

Define	Shots
Shots per slice	1
Segments	66
Trufi delta freq.	0 Hz
RF pulse type	Fast
Gradient mode	Fast
Excitation	Slice-sel.
Flip angle mode	Constant
Cine	Off

Sequence - Assistant

Mode	Off
Allowed delay	0 s

\\USER\Cardiac Research Protocols\Rapi-STRESS T1\Rapi-STRESS T1 (V2)\ShMOLLI_192i_d11_nFilt (TD=0)

TA: 7.1 s PM: FIX Voxel size: 0.9×0.9×8.0 mmPAT: 2 Rel. SNR: 1.00 : tfi

Properties

Prio recon	Off
Load images to viewer	On
Inline movie	Off
Auto store images	On
Load images to stamp segments	Off
Load images to graphic segments	On
Auto open inline display	Off
Auto close inline display	Off
Start measurement without further preparation	On
Wait for user to start	Off
Start measurements	Single measurement

Routine

Slice group	1
Slices	1
Dist. factor	25 %
Position	Isocenter
Orientation	Transversal
Phase enc. dir.	A >> P
AutoAlign	---
Phase oversampling	0 %
FoV read	360 mm
FoV phase	75.0 %
Slice thickness	8.0 mm
TR	378.98 ms
TE	1.07 ms
Averages	1
Concatenations	1
Filter	Raw filter, Distortion Corr.(2D)
Coil elements	BO2;SP2,3

Contrast - Common

TR	378.98 ms
TE	1.07 ms
Magn. preparation	Non-sel. IR
TI	260 ms
Flip angle	35 deg
Fat suppr.	None
Wrap-up Magn.	None

Contrast - Dynamic

Averages	1
Averaging mode	Short term
Reconstruction	Magn./Phase
Measurements	1
Multiple series	Off

Resolution - Common

FoV read	360 mm
FoV phase	75.0 %
Slice thickness	8.0 mm
Base resolution	192
Phase resolution	100 %
Phase partial Fourier	6/8
Trajectory	Cartesian
Interpolation	On

Resolution - iPAT

PAT mode	GRAPPA
Accel. factor PE	2
Ref. lines PE	24
Reference scan mode	Integrated

Resolution - Filter Image

Image Filter	Off
Distortion Corr.	On
Mode	2D
Unfiltered images	Off
Prescan Normalize	Off
Normalize	Off
B1 filter	Off

Resolution - Filter Rawdata

Raw filter	On
Elliptical filter	Off
POCS	Off

Geometry - Common

Slice group	1
Slices	1
Dist. factor	25 %
Position	Isocenter
Orientation	Transversal
Phase enc. dir.	A >> P
FoV read	360 mm
FoV phase	75.0 %
Slice thickness	8.0 mm
TR	378.98 ms
Multi-slice mode	Sequential
Series	Interleaved
Concatenations	1

Geometry - AutoAlign

Slice group	1
Position	Isocenter
Orientation	Transversal
Phase enc. dir.	A >> P
AutoAlign	---
Initial Position	Isocenter
Phase	0.0 mm
Read	0.0 mm
Shift	0.0 mm
Initial Rotation	0.00 deg
Initial Orientation	Transversal

Geometry - Saturation

Fat suppr.	None
Wrap-up Magn.	None
Special sat.	None

Geometry - Navigator

System - Miscellaneous

Positioning mode	FIX
Table position	H
Table position	0 mm

System - Miscellaneous

MSMA	S - C - T
Sagittal	R >> L
Coronal	A >> P
Transversal	F >> H
Coil Combine Mode	Adaptive Combine
Save uncombined	Off
Matrix Optimization	Off
Coil Focus	Flat
AutoAlign	---
Coil Select Mode	Default

System - Adjustments

B0 Shim mode	Cardiac
Adjust with body coil	On
Confirm freq. adjustment	Off
Assume Dominant Fat	Off
Assume Silicone	Off
Adjustment Tolerance	Auto

System - Adjust Volume

! Position	Isocenter
! Orientation	Transversal
! Rotation	0.00 deg
! A >> P	150 mm
! R >> L	150 mm
! F >> H	150 mm
Reset	Off

System - Tx/Rx

Frequency 1H	63.683250 MHz
Correction factor	1
Gain	High
Img. Scale Cor.	1.000
Reset	Off
? Ref. amplitude 1H	0.000 V

Physio - Signal1

1st Signal/Mode	ECG/Trigger
Average cycle	787 ± 129 ms
Average cycle	No Signal ms
Captured cycle	787 ± 129 ms
Acquisition window	379 ms
Trigger pulse	1
Trigger delay	0 ms
TR	378.98 ms
Concatenations	1
Segments	84
Phases	1
Adaptive Triggering	Off

Physio - Cardiac

Tagging	None
Magn. preparation	Non-sel. IR
TI	260 ms
Fat suppr.	None
Dark blood	Off
FoV read	360 mm
FoV phase	75.0 %
Phase resolution	100 %
Cine	Off
Trajectory	Cartesian
Dummy heartbeats	0
Motion Correction	None

Physio - PACE

Resp. control	Off
Concatenations	1

Sequence - Part 1

Introduction	Off
Dimension	2D
Reordering	Linear
Asymmetric echo	Weak
Contrasts	1
Optimization	Min. TE TR
Multi-slice mode	Sequential
Sequence type	Trufi
Bandwidth	898 Hz/Px

Sequence - Part 2

Define	Shots
Shots per slice	1
Segments	84
Trufi delta freq.	0 Hz
RF pulse type	Fast
Gradient mode	Fast
Excitation	Slice-sel.
Flip angle mode	Constant
Cine	Off

Sequence - Assistant

Mode	Off
Allowed delay	0 s

\\USER\Cardiac Research Protocols\Rapi-STRESS T1\Rapi-STRESS T1 (V2)\MidShMOLLI_192i_d11_nFilt (TD=0)

TA: 7.1 s PM: REF Voxel size: 0.9×0.9×8.0 mmPAT: 2 Rel. SNR: 1.00 : tfi

Properties

Prio recon	Off
Load images to viewer	On
Inline movie	Off
Auto store images	On
Load images to stamp segments	Off
Load images to graphic segments	On
Auto open inline display	Off
Auto close inline display	Off
Start measurement without further preparation	Off
Wait for user to start	Off
Start measurements	Single measurement

Routine

Slice group	1
Slices	1
Dist. factor	25 %
Position	Isocenter
Orientation	Transversal
Phase enc. dir.	A >> P
AutoAlign	---
Phase oversampling	0 %
FoV read	360 mm
FoV phase	75.0 %
Slice thickness	8.0 mm
TR	378.98 ms
TE	1.07 ms
Averages	1
Concatenations	1
Filter	Raw filter, Distortion Corr.(2D)
Coil elements	BO2;SP2,3

Contrast - Common

TR	378.98 ms
TE	1.07 ms
Magn. preparation	Non-sel. IR
TI	260 ms
Flip angle	35 deg
Fat suppr.	None
Wrap-up Magn.	None

Contrast - Dynamic

Averages	1
Averaging mode	Short term
Reconstruction	Magn./Phase
Measurements	1
Multiple series	Off

Resolution - Common

FoV read	360 mm
FoV phase	75.0 %
Slice thickness	8.0 mm
Base resolution	192
Phase resolution	100 %
Phase partial Fourier	6/8
Trajectory	Cartesian
Interpolation	On

Resolution - iPAT

PAT mode	GRAPPA
Accel. factor PE	2
Ref. lines PE	24
Reference scan mode	Integrated

Resolution - Filter Image

Image Filter	Off
Distortion Corr.	On
Mode	2D
Unfiltered images	Off
Prescan Normalize	Off
Normalize	Off
B1 filter	Off

Resolution - Filter Rawdata

Raw filter	On
Elliptical filter	Off
POCS	Off

Geometry - Common

Slice group	1
Slices	1
Dist. factor	25 %
Position	Isocenter
Orientation	Transversal
Phase enc. dir.	A >> P
FoV read	360 mm
FoV phase	75.0 %
Slice thickness	8.0 mm
TR	378.98 ms
Multi-slice mode	Sequential
Series	Interleaved
Concatenations	1

Geometry - AutoAlign

Slice group	1
Position	Isocenter
Orientation	Transversal
Phase enc. dir.	A >> P
AutoAlign	---
Initial Position	Isocenter
Phase	0.0 mm
Read	0.0 mm
Shift	0.0 mm
Initial Rotation	0.00 deg
Initial Orientation	Transversal

Geometry - Saturation

Fat suppr.	None
Wrap-up Magn.	None
Special sat.	None

Geometry - Navigator

System - Miscellaneous

Positioning mode	REF
Table position	H
Table position	0 mm

System - Miscellaneous

MSMA	S - C - T
Sagittal	R >> L
Coronal	A >> P
Transversal	F >> H
Coil Combine Mode	Adaptive Combine
Save uncombined	Off
Matrix Optimization	Off
Coil Focus	Flat
AutoAlign	---
Coil Select Mode	Default

System - Adjustments

B0 Shim mode	Cardiac
Adjust with body coil	On
Confirm freq. adjustment	Off
Assume Dominant Fat	Off
Assume Silicone	Off
Adjustment Tolerance	Auto

System - Adjust Volume

! Position	Isocenter
! Orientation	Transversal
! Rotation	0.00 deg
! A >> P	150 mm
! R >> L	150 mm
! F >> H	150 mm
Reset	Off

System - Tx/Rx

Frequency 1H	63.683250 MHz
Correction factor	1
Gain	High
Img. Scale Cor.	1.000
Reset	Off
? Ref. amplitude 1H	0.000 V

Physio - Signal1

1st Signal/Mode	ECG/Trigger
Average cycle	787 ± 129 ms
Average cycle	No Signal ms
Captured cycle	787 ± 129 ms
Acquisition window	379 ms
Trigger pulse	1
Trigger delay	0 ms
TR	378.98 ms
Concatenations	1
Segments	84
Phases	1
Adaptive Triggering	Off

Physio - Cardiac

Tagging	None
Magn. preparation	Non-sel. IR
TI	260 ms
Fat suppr.	None
Dark blood	Off
FoV read	360 mm
FoV phase	75.0 %
Phase resolution	100 %
Cine	Off
Trajectory	Cartesian
Dummy heartbeats	0
Motion Correction	None

Physio - PACE

Resp. control	Off
Concatenations	1

Sequence - Part 1

Introduction	Off
Dimension	2D
Reordering	Linear
Asymmetric echo	Weak
Contrasts	1
Optimization	Min. TE TR
Multi-slice mode	Sequential
Sequence type	Trufi
Bandwidth	898 Hz/Px

Sequence - Part 2

Define	Shots
Shots per slice	1
Segments	84
Trufi delta freq.	0 Hz
RF pulse type	Fast
Gradient mode	Fast
Excitation	Slice-sel.
Flip angle mode	Constant
Cine	Off

Sequence - Assistant

Mode	Off
Allowed delay	0 s

\\USER\Cardiac Research Protocols\Rapi-STRESS T1\Rapi-STRESS T1 (V2)\T1Map_LongT1 (TD=0)LowHR

TA: 8.7 s PM: FIX Voxel size: 1.4×1.4×8.0 mmPAT: 2 Rel. SNR: 1.00 : tfi

Properties

Prio recon	Off
Load images to viewer	On
Inline movie	Off
Auto store images	On
Load images to stamp segments	On
Load images to graphic segments	On
Auto open inline display	Off
Auto close inline display	Off
Start measurement without further preparation	On
Wait for user to start	Off
Start measurements	Single measurement

Routine

Slice group	1
Slices	1
Dist. factor	25 %
Position	Isocenter
Orientation	Transversal
Phase enc. dir.	A >> P
AutoAlign	---
Phase oversampling	0 %
FoV read	360 mm
FoV phase	85.2 %
Slice thickness	8.0 mm
TR	279.84 ms
TE	1.13 ms
Averages	1
Concatenations	1
Filter	Distortion Corr.(2D)
Coil elements	BO2;SP2,3

Contrast - Common

TR	279.84 ms
TE	1.13 ms
Magn. preparation	Non-sel. IR T1map
TI	180 ms
Flip angle	35 deg
Fat suppr.	None
Wrap-up Magn.	None

Contrast - Dynamic

Averages	1
Averaging mode	Short term
Reconstruction	Magnitude
Measurements	1
Multiple series	Off

Resolution - Common

FoV read	360 mm
FoV phase	85.2 %
Slice thickness	8.0 mm
Base resolution	256
Phase resolution	66 %
Phase partial Fourier	7/8
Trajectory	Cartesian
Interpolation	Off

Resolution - iPAT

PAT mode	GRAPPA
Accel. factor PE	2
Ref. lines PE	36
Reference scan mode	GRE/separate

Resolution - Filter Image

Image Filter	Off
Distortion Corr.	On
Mode	2D
Unfiltered images	Off
Prescan Normalize	Off
Normalize	Off
B1 filter	Off

Resolution - Filter Rawdata

Raw filter	Off
Elliptical filter	Off
POCS	Off

Geometry - Common

Slice group	1
Slices	1
Dist. factor	25 %
Position	Isocenter
Orientation	Transversal
Phase enc. dir.	A >> P
FoV read	360 mm
FoV phase	85.2 %
Slice thickness	8.0 mm
TR	279.84 ms
Multi-slice mode	Sequential
Series	Base To Apex
Concatenations	1

Geometry - AutoAlign

Slice group	1
Position	Isocenter
Orientation	Transversal
Phase enc. dir.	A >> P
AutoAlign	---
Initial Position	Isocenter
Phase	0.0 mm
Read	0.0 mm
Shift	0.0 mm
Initial Rotation	0.00 deg
Initial Orientation	Transversal

Geometry - Saturation

Fat suppr.	None
Wrap-up Magn.	None
Special sat.	None

Geometry - Navigator

System - Miscellaneous

Positioning mode	FIX
Table position	H
Table position	0 mm

System - Miscellaneous

MSMA	S - C - T
Sagittal	R >> L
Coronal	A >> P
Transversal	F >> H
Coil Combine Mode	Adaptive Combine
Save uncombined	Off
Matrix Optimization	Off
Coil Focus	Flat
AutoAlign	---
Coil Select Mode	Default

System - Adjustments

B0 Shim mode	Cardiac
Adjust with body coil	Off
Confirm freq. adjustment	Off
Assume Dominant Fat	Off
Assume Silicone	Off
Adjustment Tolerance	Auto

System - Adjust Volume

! Position	Isocenter
! Orientation	Transversal
! Rotation	0.00 deg
! A >> P	150 mm
! R >> L	150 mm
! F >> H	150 mm
Reset	Off

System - Tx/Rx

Frequency 1H	63.683250 MHz
Correction factor	1
Gain	High
Img. Scale Cor.	1.000
Reset	Off
? Ref. amplitude 1H	0.000 V

Physio - Signal1

1st Signal/Mode	ECG/Trigger
Average cycle	787 ± 129 ms
Average cycle	No Signal ms
Captured cycle	787 ± 129 ms
Acquisition window	280 ms
Trigger pulse	1
Trigger delay	0 ms
TR	279.84 ms
Concatenations	1
Segments	72
Phases	1
Adaptive Triggering	Off

Physio - Cardiac

Tagging	None
Magn. preparation	Non-sel. IR T1map
TI	180 ms
Fat suppr.	None
Dark blood	Off
FoV read	360 mm
FoV phase	85.2 %
Phase resolution	66 %
Cine	Off
Trajectory	Cartesian
Dummy heartbeats	0
Motion Correction	Standard

Physio - PACE

Resp. control	Breath-hold
Concatenations	1

Sequence - Part 1

Introduction	Off
Dimension	2D
Reordering	Linear
Asymmetric echo	Weak
Contrasts	1
Optimization	Min. TE TR
Multi-slice mode	Sequential
Sequence type	Trufi
Bandwidth	1085 Hz/Px

Sequence - Part 2

Define	Shots
Shots per slice	1
Segments	72
Trufi delta freq.	0 Hz
RF pulse type	Fast
Gradient mode	Fast
Excitation	Slice-sel.
Flip angle mode	Constant
Cine	Off

Sequence - Assistant

Mode	Off
Allowed delay	0 s

\\USER\Cardiac Research Protocols\Rapi-STRESS T1\Rapi-STRESS T1 (V2)\ShMOLLI_192i_d11_nFilt (TD=0)

TA: 7.1 s PM: FIX Voxel size: 0.9×0.9×8.0 mmPAT: 2 Rel. SNR: 1.00 : tfi

Properties

Prio recon	Off
Load images to viewer	On
Inline movie	Off
Auto store images	On
Load images to stamp segments	Off
Load images to graphic segments	On
Auto open inline display	Off
Auto close inline display	Off
Start measurement without further preparation	On
Wait for user to start	Off
Start measurements	Single measurement

Routine

Slice group	1
Slices	1
Dist. factor	25 %
Position	Isocenter
Orientation	Transversal
Phase enc. dir.	A >> P
AutoAlign	---
Phase oversampling	0 %
FoV read	360 mm
FoV phase	75.0 %
Slice thickness	8.0 mm
TR	378.98 ms
TE	1.07 ms
Averages	1
Concatenations	1
Filter	Raw filter, Distortion Corr.(2D)
Coil elements	BO2;SP2,3

Contrast - Common

TR	378.98 ms
TE	1.07 ms
Magn. preparation	Non-sel. IR
TI	260 ms
Flip angle	35 deg
Fat suppr.	None
Wrap-up Magn.	None

Contrast - Dynamic

Averages	1
Averaging mode	Short term
Reconstruction	Magn./Phase
Measurements	1
Multiple series	Off

Resolution - Common

FoV read	360 mm
FoV phase	75.0 %
Slice thickness	8.0 mm
Base resolution	192
Phase resolution	100 %
Phase partial Fourier	6/8
Trajectory	Cartesian
Interpolation	On

Resolution - iPAT

PAT mode	GRAPPA
Accel. factor PE	2
Ref. lines PE	24
Reference scan mode	Integrated

Resolution - Filter Image

Image Filter	Off
Distortion Corr.	On
Mode	2D
Unfiltered images	Off
Prescan Normalize	Off
Normalize	Off
B1 filter	Off

Resolution - Filter Rawdata

Raw filter	On
Elliptical filter	Off
POCS	Off

Geometry - Common

Slice group	1
Slices	1
Dist. factor	25 %
Position	Isocenter
Orientation	Transversal
Phase enc. dir.	A >> P
FoV read	360 mm
FoV phase	75.0 %
Slice thickness	8.0 mm
TR	378.98 ms
Multi-slice mode	Sequential
Series	Interleaved
Concatenations	1

Geometry - AutoAlign

Slice group	1
Position	Isocenter
Orientation	Transversal
Phase enc. dir.	A >> P
AutoAlign	---
Initial Position	Isocenter
Phase	0.0 mm
Read	0.0 mm
Shift	0.0 mm
Initial Rotation	0.00 deg
Initial Orientation	Transversal

Geometry - Saturation

Fat suppr.	None
Wrap-up Magn.	None
Special sat.	None

Geometry - Navigator

System - Miscellaneous

Positioning mode	FIX
Table position	H
Table position	0 mm

System - Miscellaneous

MSMA	S - C - T
Sagittal	R >> L
Coronal	A >> P
Transversal	F >> H
Coil Combine Mode	Adaptive Combine
Save uncombined	Off
Matrix Optimization	Off
Coil Focus	Flat
AutoAlign	---
Coil Select Mode	Default

System - Adjustments

B0 Shim mode	Cardiac
Adjust with body coil	On
Confirm freq. adjustment	Off
Assume Dominant Fat	Off
Assume Silicone	Off
Adjustment Tolerance	Auto

System - Adjust Volume

! Position	Isocenter
! Orientation	Transversal
! Rotation	0.00 deg
! A >> P	150 mm
! R >> L	150 mm
! F >> H	150 mm
Reset	Off

System - Tx/Rx

Frequency 1H	63.683250 MHz
Correction factor	1
Gain	High
Img. Scale Cor.	1.000
Reset	Off
? Ref. amplitude 1H	0.000 V

Physio - Signal1

1st Signal/Mode	ECG/Trigger
Average cycle	787 ± 129 ms
Average cycle	No Signal ms
Captured cycle	787 ± 129 ms
Acquisition window	379 ms
Trigger pulse	1
Trigger delay	0 ms
TR	378.98 ms
Concatenations	1
Segments	84
Phases	1
Adaptive Triggering	Off

Physio - Cardiac

Tagging	None
Magn. preparation	Non-sel. IR
TI	260 ms
Fat suppr.	None
Dark blood	Off
FoV read	360 mm
FoV phase	75.0 %
Phase resolution	100 %
Cine	Off
Trajectory	Cartesian
Dummy heartbeats	0
Motion Correction	None

Physio - PACE

Resp. control	Off
Concatenations	1

Sequence - Part 1

Introduction	Off
Dimension	2D
Reordering	Linear
Asymmetric echo	Weak
Contrasts	1
Optimization	Min. TE TR
Multi-slice mode	Sequential
Sequence type	Trufi
Bandwidth	898 Hz/Px

Sequence - Part 2

Define	Shots
Shots per slice	1
Segments	84
Trufi delta freq.	0 Hz
RF pulse type	Fast
Gradient mode	Fast
Excitation	Slice-sel.
Flip angle mode	Constant
Cine	Off

Sequence - Assistant

Mode	Off
Allowed delay	0 s

\\USER\Cardiac Research Protocols\Rapi-STRESS T1\Rapi-STRESS T1 (V2)\T1Map_LongT1_TD0_HighHR

TA: 8.7 s PM: FIX Voxel size: 1.9×1.9×8.0 mmPAT: 2 Rel. SNR: 1.00 : tfi

Properties

Prio recon	Off
Load images to viewer	On
Inline movie	Off
Auto store images	On
Load images to stamp segments	On
Load images to graphic segments	On
Auto open inline display	Off
Auto close inline display	Off
Start measurement without further preparation	On
Wait for user to start	Off
Start measurements	Single measurement

Routine

Slice group	1
Slices	1
Dist. factor	20 %
Position	Isocenter
Orientation	Transversal
Phase enc. dir.	A >> P
AutoAlign	---
Phase oversampling	0 %
FoV read	360 mm
FoV phase	85.4 %
Slice thickness	8.0 mm
TR	263.55 ms
TE	1.01 ms
Averages	1
Concatenations	1
Filter	Distortion Corr.(2D)
Coil elements	BO2;SP2,3

Contrast - Common

TR	263.55 ms
TE	1.01 ms
Magn. preparation	Non-sel. IR T1map
TI	180 ms
Flip angle	35 deg
Fat suppr.	None
Wrap-up Magn.	None

Contrast - Dynamic

Averages	1
Averaging mode	Short term
Reconstruction	Magnitude
Measurements	1
Multiple series	Off

Resolution - Common

FoV read	360 mm
FoV phase	85.4 %
Slice thickness	8.0 mm
Base resolution	192
Phase resolution	80 %
Phase partial Fourier	7/8
Trajectory	Cartesian
Interpolation	Off

Resolution - iPAT

PAT mode	GRAPPA
Accel. factor PE	2
Ref. lines PE	36
Reference scan mode	GRE/separate

Resolution - Filter Image

Image Filter	Off
Distortion Corr.	On
Mode	2D
Unfiltered images	Off
Prescan Normalize	Off
Normalize	Off
B1 filter	Off

Resolution - Filter Rawdata

Raw filter	Off
Elliptical filter	Off
POCS	Off

Geometry - Common

Slice group	1
Slices	1
Dist. factor	20 %
Position	Isocenter
Orientation	Transversal
Phase enc. dir.	A >> P
FoV read	360 mm
FoV phase	85.4 %
Slice thickness	8.0 mm
TR	263.55 ms
Multi-slice mode	Sequential
Series	Base To Apex
Concatenations	1

Geometry - AutoAlign

Slice group	1
Position	Isocenter
Orientation	Transversal
Phase enc. dir.	A >> P
AutoAlign	---
Initial Position	Isocenter
Phase	0.0 mm
Read	0.0 mm
Shift	0.0 mm
Initial Rotation	0.00 deg
Initial Orientation	Transversal

Geometry - Saturation

Fat suppr.	None
Wrap-up Magn.	None
Special sat.	None

Geometry - Navigator

System - Miscellaneous

Positioning mode	FIX
Table position	H
Table position	0 mm

System - Miscellaneous

MSMA	S - C - T
Sagittal	R >> L
Coronal	A >> P
Transversal	F >> H
Coil Combine Mode	Adaptive Combine
Save uncombined	Off
Matrix Optimization	Off
Coil Focus	Flat
AutoAlign	---
Coil Select Mode	Default

System - Adjustments

B0 Shim mode	Cardiac
Adjust with body coil	Off
Confirm freq. adjustment	Off
Assume Dominant Fat	Off
Assume Silicone	Off
Adjustment Tolerance	Auto

System - Adjust Volume

! Position	Isocenter
! Orientation	Transversal
! Rotation	0.00 deg
! A >> P	150 mm
! R >> L	150 mm
! F >> H	150 mm
Reset	Off

System - Tx/Rx

Frequency 1H	63.683250 MHz
Correction factor	1
Gain	High
Img. Scale Cor.	1.000
Reset	Off
? Ref. amplitude 1H	0.000 V

Physio - Signal1

1st Signal/Mode	ECG/Trigger
Average cycle	787 ± 129 ms
Average cycle	No Signal ms
Captured cycle	787 ± 129 ms
Acquisition window	700 ms
Trigger pulse	1
Trigger delay	0 ms
TR	263.55 ms
Concatenations	1
Segments	66
Phases	1
Adaptive Triggering	Off

Physio - Cardiac

Tagging	None
Magn. preparation	Non-sel. IR T1map
TI	180 ms
Fat suppr.	None
Dark blood	Off
FoV read	360 mm
FoV phase	85.4 %
Phase resolution	80 %
Cine	Off
Trajectory	Cartesian
Dummy heartbeats	0
Motion Correction	Standard

Physio - PACE

Resp. control	Breath-hold
Concatenations	1

Sequence - Part 1

Introduction	Off
Dimension	2D
Reordering	Linear
Asymmetric echo	Weak
Contrasts	1
Optimization	Min. TE TR
Multi-slice mode	Sequential
Sequence type	Trufi
Bandwidth	1085 Hz/Px

Sequence - Part 2

Define	Shots
Shots per slice	1
Segments	66
Trufi delta freq.	0 Hz
RF pulse type	Fast
Gradient mode	Fast
Excitation	Slice-sel.
Flip angle mode	Constant
Cine	Off

Sequence - Assistant

Mode	Off
Allowed delay	0 s

\\USER\Cardiac Research Protocols\Rapi-STRESS T1\Rapi-STRESS T1 (V2)\ShMOLLI_192i_d11_nFilt (TD=0)

TA: 7.1 s PM: FIX Voxel size: 0.9×0.9×8.0 mmPAT: 2 Rel. SNR: 1.00 : tfi

Properties

Prio recon	Off
Load images to viewer	On
Inline movie	Off
Auto store images	On
Load images to stamp segments	Off
Load images to graphic segments	On
Auto open inline display	Off
Auto close inline display	Off
Start measurement without further preparation	On
Wait for user to start	Off
Start measurements	Single measurement

Routine

Slice group	1
Slices	1
Dist. factor	25 %
Position	Isocenter
Orientation	Transversal
Phase enc. dir.	A >> P
AutoAlign	---
Phase oversampling	0 %
FoV read	360 mm
FoV phase	75.0 %
Slice thickness	8.0 mm
TR	378.98 ms
TE	1.07 ms
Averages	1
Concatenations	1
Filter	Raw filter, Distortion Corr.(2D)
Coil elements	BO2;SP2,3

Contrast - Common

TR	378.98 ms
TE	1.07 ms
Magn. preparation	Non-sel. IR
TI	260 ms
Flip angle	35 deg
Fat suppr.	None
Wrap-up Magn.	None

Contrast - Dynamic

Averages	1
Averaging mode	Short term
Reconstruction	Magn./Phase
Measurements	1
Multiple series	Off

Resolution - Common

FoV read	360 mm
FoV phase	75.0 %
Slice thickness	8.0 mm
Base resolution	192
Phase resolution	100 %
Phase partial Fourier	6/8
Trajectory	Cartesian
Interpolation	On

Resolution - iPAT

PAT mode	GRAPPA
Accel. factor PE	2
Ref. lines PE	24
Reference scan mode	Integrated

Resolution - Filter Image

Image Filter	Off
Distortion Corr.	On
Mode	2D
Unfiltered images	Off
Prescan Normalize	Off
Normalize	Off
B1 filter	Off

Resolution - Filter Rawdata

Raw filter	On
Elliptical filter	Off
POCS	Off

Geometry - Common

Slice group	1
Slices	1
Dist. factor	25 %
Position	Isocenter
Orientation	Transversal
Phase enc. dir.	A >> P
FoV read	360 mm
FoV phase	75.0 %
Slice thickness	8.0 mm
TR	378.98 ms
Multi-slice mode	Sequential
Series	Interleaved
Concatenations	1

Geometry - AutoAlign

Slice group	1
Position	Isocenter
Orientation	Transversal
Phase enc. dir.	A >> P
AutoAlign	---
Initial Position	Isocenter
Phase	0.0 mm
Read	0.0 mm
Shift	0.0 mm
Initial Rotation	0.00 deg
Initial Orientation	Transversal

Geometry - Saturation

Fat suppr.	None
Wrap-up Magn.	None
Special sat.	None

Geometry - Navigator

System - Miscellaneous

Positioning mode	FIX
Table position	H
Table position	0 mm

System - Miscellaneous

MSMA	S - C - T
Sagittal	R >> L
Coronal	A >> P
Transversal	F >> H
Coil Combine Mode	Adaptive Combine
Save uncombined	Off
Matrix Optimization	Off
Coil Focus	Flat
AutoAlign	---
Coil Select Mode	Default

System - Adjustments

B0 Shim mode	Cardiac
Adjust with body coil	On
Confirm freq. adjustment	Off
Assume Dominant Fat	Off
Assume Silicone	Off
Adjustment Tolerance	Auto

System - Adjust Volume

! Position	Isocenter
! Orientation	Transversal
! Rotation	0.00 deg
! A >> P	150 mm
! R >> L	150 mm
! F >> H	150 mm
Reset	Off

System - Tx/Rx

Frequency 1H	63.683250 MHz
Correction factor	1
Gain	High
Img. Scale Cor.	1.000
Reset	Off
? Ref. amplitude 1H	0.000 V

Physio - Signal1

1st Signal/Mode	ECG/Trigger
Average cycle	787 ± 129 ms
Average cycle	No Signal ms
Captured cycle	787 ± 129 ms
Acquisition window	379 ms
Trigger pulse	1
Trigger delay	0 ms
TR	378.98 ms
Concatenations	1
Segments	84
Phases	1
Adaptive Triggering	Off

Physio - Cardiac

Tagging	None
Magn. preparation	Non-sel. IR
TI	260 ms
Fat suppr.	None
Dark blood	Off
FoV read	360 mm
FoV phase	75.0 %
Phase resolution	100 %
Cine	Off
Trajectory	Cartesian
Dummy heartbeats	0
Motion Correction	None

Physio - PACE

Resp. control	Off
Concatenations	1

Sequence - Part 1

Introduction	Off
Dimension	2D
Reordering	Linear
Asymmetric echo	Weak
Contrasts	1
Optimization	Min. TE TR
Multi-slice mode	Sequential
Sequence type	Trufi
Bandwidth	898 Hz/Px

Sequence - Part 2

Define	Shots
Shots per slice	1
Segments	84
Trufi delta freq.	0 Hz
RF pulse type	Fast
Gradient mode	Fast
Excitation	Slice-sel.
Flip angle mode	Constant
Cine	Off

Sequence - Assistant

Mode	Off
Allowed delay	0 s

\\USER\Cardiac Research Protocols\Rapi-STRESS T1\Rapi-STRESS T1 (V2)\MidShMOLLI_192i_d11_nFilt (TD=0)

TA: 7.1 s PM: REF Voxel size: 0.9×0.9×8.0 mmPAT: 2 Rel. SNR: 1.00 : tfi

Properties

Prio recon	Off
Load images to viewer	On
Inline movie	Off
Auto store images	On
Load images to stamp segments	Off
Load images to graphic segments	On
Auto open inline display	Off
Auto close inline display	Off
Start measurement without further preparation	Off
Wait for user to start	Off
Start measurements	Single measurement

Routine

Slice group	1
Slices	1
Dist. factor	25 %
Position	Isocenter
Orientation	Transversal
Phase enc. dir.	A >> P
AutoAlign	---
Phase oversampling	0 %
FoV read	360 mm
FoV phase	75.0 %
Slice thickness	8.0 mm
TR	378.98 ms
TE	1.07 ms
Averages	1
Concatenations	1
Filter	Raw filter, Distortion Corr.(2D)
Coil elements	BO2;SP2,3

Contrast - Common

TR	378.98 ms
TE	1.07 ms
Magn. preparation	Non-sel. IR
TI	260 ms
Flip angle	35 deg
Fat suppr.	None
Wrap-up Magn.	None

Contrast - Dynamic

Averages	1
Averaging mode	Short term
Reconstruction	Magn./Phase
Measurements	1
Multiple series	Off

Resolution - Common

FoV read	360 mm
FoV phase	75.0 %
Slice thickness	8.0 mm
Base resolution	192
Phase resolution	100 %
Phase partial Fourier	6/8
Trajectory	Cartesian
Interpolation	On

Resolution - iPAT

PAT mode	GRAPPA
Accel. factor PE	2
Ref. lines PE	24
Reference scan mode	Integrated

Resolution - Filter Image

Image Filter	Off
Distortion Corr.	On
Mode	2D
Unfiltered images	Off
Prescan Normalize	Off
Normalize	Off
B1 filter	Off

Resolution - Filter Rawdata

Raw filter	On
Elliptical filter	Off
POCS	Off

Geometry - Common

Slice group	1
Slices	1
Dist. factor	25 %
Position	Isocenter
Orientation	Transversal
Phase enc. dir.	A >> P
FoV read	360 mm
FoV phase	75.0 %
Slice thickness	8.0 mm
TR	378.98 ms
Multi-slice mode	Sequential
Series	Interleaved
Concatenations	1

Geometry - AutoAlign

Slice group	1
Position	Isocenter
Orientation	Transversal
Phase enc. dir.	A >> P
AutoAlign	---
Initial Position	Isocenter
Phase	0.0 mm
Read	0.0 mm
Shift	0.0 mm
Initial Rotation	0.00 deg
Initial Orientation	Transversal

Geometry - Saturation

Fat suppr.	None
Wrap-up Magn.	None
Special sat.	None

Geometry - Navigator

System - Miscellaneous

Positioning mode	REF
Table position	H
Table position	0 mm

System - Miscellaneous

MSMA	S - C - T
Sagittal	R >> L
Coronal	A >> P
Transversal	F >> H
Coil Combine Mode	Adaptive Combine
Save uncombined	Off
Matrix Optimization	Off
Coil Focus	Flat
AutoAlign	---
Coil Select Mode	Default

System - Adjustments

B0 Shim mode	Cardiac
Adjust with body coil	On
Confirm freq. adjustment	Off
Assume Dominant Fat	Off
Assume Silicone	Off
Adjustment Tolerance	Auto

System - Adjust Volume

! Position	Isocenter
! Orientation	Transversal
! Rotation	0.00 deg
! A >> P	150 mm
! R >> L	150 mm
! F >> H	150 mm
Reset	Off

System - Tx/Rx

Frequency 1H	63.683250 MHz
Correction factor	1
Gain	High
Img. Scale Cor.	1.000
Reset	Off
? Ref. amplitude 1H	0.000 V

Physio - Signal1

1st Signal/Mode	ECG/Trigger
Average cycle	787 ± 129 ms
Average cycle	No Signal ms
Captured cycle	787 ± 129 ms
Acquisition window	379 ms
Trigger pulse	1
Trigger delay	0 ms
TR	378.98 ms
Concatenations	1
Segments	84
Phases	1
Adaptive Triggering	Off

Physio - Cardiac

Tagging	None
Magn. preparation	Non-sel. IR
TI	260 ms
Fat suppr.	None
Dark blood	Off
FoV read	360 mm
FoV phase	75.0 %
Phase resolution	100 %
Cine	Off
Trajectory	Cartesian
Dummy heartbeats	0
Motion Correction	None

Physio - PACE

Resp. control	Off
Concatenations	1

Sequence - Part 1

Introduction	Off
Dimension	2D
Reordering	Linear
Asymmetric echo	Weak
Contrasts	1
Optimization	Min. TE TR
Multi-slice mode	Sequential
Sequence type	Trufi
Bandwidth	898 Hz/Px

Sequence - Part 2

Define	Shots
Shots per slice	1
Segments	84
Trufi delta freq.	0 Hz
RF pulse type	Fast
Gradient mode	Fast
Excitation	Slice-sel.
Flip angle mode	Constant
Cine	Off

Sequence - Assistant

Mode	Off
Allowed delay	0 s

\\USER\Cardiac Research Protocols\Rapi-STRESS T1\Rapi-STRESS T1 (V2)\T1Map_LongT1 (TD=0)LowHR

TA: 8.7 s PM: FIX Voxel size: 1.4×1.4×8.0 mmPAT: 2 Rel. SNR: 1.00 : tfi

Properties

Prio recon	Off
Load images to viewer	On
Inline movie	Off
Auto store images	On
Load images to stamp segments	On
Load images to graphic segments	On
Auto open inline display	Off
Auto close inline display	Off
Start measurement without further preparation	On
Wait for user to start	Off
Start measurements	Single measurement

Routine

Slice group	1
Slices	1
Dist. factor	25 %
Position	Isocenter
Orientation	Transversal
Phase enc. dir.	A >> P
AutoAlign	---
Phase oversampling	0 %
FoV read	360 mm
FoV phase	85.2 %
Slice thickness	8.0 mm
TR	279.84 ms
TE	1.13 ms
Averages	1
Concatenations	1
Filter	Distortion Corr.(2D)
Coil elements	BO2;SP2,3

Contrast - Common

TR	279.84 ms
TE	1.13 ms
Magn. preparation	Non-sel. IR T1map
TI	180 ms
Flip angle	35 deg
Fat suppr.	None
Wrap-up Magn.	None

Contrast - Dynamic

Averages	1
Averaging mode	Short term
Reconstruction	Magnitude
Measurements	1
Multiple series	Off

Resolution - Common

FoV read	360 mm
FoV phase	85.2 %
Slice thickness	8.0 mm
Base resolution	256
Phase resolution	66 %
Phase partial Fourier	7/8
Trajectory	Cartesian
Interpolation	Off

Resolution - iPAT

PAT mode	GRAPPA
Accel. factor PE	2
Ref. lines PE	36
Reference scan mode	GRE/separate

Resolution - Filter Image

Image Filter	Off
Distortion Corr.	On
Mode	2D
Unfiltered images	Off
Prescan Normalize	Off
Normalize	Off
B1 filter	Off

Resolution - Filter Rawdata

Raw filter	Off
Elliptical filter	Off
POCS	Off

Geometry - Common

Slice group	1
Slices	1
Dist. factor	25 %
Position	Isocenter
Orientation	Transversal
Phase enc. dir.	A >> P
FoV read	360 mm
FoV phase	85.2 %
Slice thickness	8.0 mm
TR	279.84 ms
Multi-slice mode	Sequential
Series	Base To Apex
Concatenations	1

Geometry - AutoAlign

Slice group	1
Position	Isocenter
Orientation	Transversal
Phase enc. dir.	A >> P
AutoAlign	---
Initial Position	Isocenter
Phase	0.0 mm
Read	0.0 mm
Shift	0.0 mm
Initial Rotation	0.00 deg
Initial Orientation	Transversal

Geometry - Saturation

Fat suppr.	None
Wrap-up Magn.	None
Special sat.	None

Geometry - Navigator

System - Miscellaneous

Positioning mode	FIX
Table position	H
Table position	0 mm

System - Miscellaneous

MSMA	S - C - T
Sagittal	R >> L
Coronal	A >> P
Transversal	F >> H
Coil Combine Mode	Adaptive Combine
Save uncombined	Off
Matrix Optimization	Off
Coil Focus	Flat
AutoAlign	---
Coil Select Mode	Default

System - Adjustments

B0 Shim mode	Cardiac
Adjust with body coil	Off
Confirm freq. adjustment	Off
Assume Dominant Fat	Off
Assume Silicone	Off
Adjustment Tolerance	Auto

System - Adjust Volume

! Position	Isocenter
! Orientation	Transversal
! Rotation	0.00 deg
! A >> P	150 mm
! R >> L	150 mm
! F >> H	150 mm
Reset	Off

System - Tx/Rx

Frequency 1H	63.683250 MHz
Correction factor	1
Gain	High
Img. Scale Cor.	1.000
Reset	Off
? Ref. amplitude 1H	0.000 V

Physio - Signal1

1st Signal/Mode	ECG/Trigger
Average cycle	787 ± 129 ms
Average cycle	No Signal ms
Captured cycle	787 ± 129 ms
Acquisition window	280 ms
Trigger pulse	1
Trigger delay	0 ms
TR	279.84 ms
Concatenations	1
Segments	72
Phases	1
Adaptive Triggering	Off

Physio - Cardiac

Tagging	None
Magn. preparation	Non-sel. IR T1map
TI	180 ms
Fat suppr.	None
Dark blood	Off
FoV read	360 mm
FoV phase	85.2 %
Phase resolution	66 %
Cine	Off
Trajectory	Cartesian
Dummy heartbeats	0
Motion Correction	Standard

Physio - PACE

Resp. control	Breath-hold
Concatenations	1

Sequence - Part 1

Introduction	Off
Dimension	2D
Reordering	Linear
Asymmetric echo	Weak
Contrasts	1
Optimization	Min. TE TR
Multi-slice mode	Sequential
Sequence type	Trufi
Bandwidth	1085 Hz/Px

Sequence - Part 2

Define	Shots
Shots per slice	1
Segments	72
Trufi delta freq.	0 Hz
RF pulse type	Fast
Gradient mode	Fast
Excitation	Slice-sel.
Flip angle mode	Constant
Cine	Off

Sequence - Assistant

Mode	Off
Allowed delay	0 s

\\USER\Cardiac Research Protocols\Rapi-STRESS T1\Rapi-STRESS T1 (V2)\ShMOLLI_192i_d11_nFilt (TD=0)

TA: 7.1 s PM: FIX Voxel size: 0.9×0.9×8.0 mmPAT: 2 Rel. SNR: 1.00 : tfi

Properties

Prio recon	Off
Load images to viewer	On
Inline movie	Off
Auto store images	On
Load images to stamp segments	Off
Load images to graphic segments	On
Auto open inline display	Off
Auto close inline display	Off
Start measurement without further preparation	On
Wait for user to start	Off
Start measurements	Single measurement

Routine

Slice group	1
Slices	1
Dist. factor	25 %
Position	Isocenter
Orientation	Transversal
Phase enc. dir.	A >> P
AutoAlign	---
Phase oversampling	0 %
FoV read	360 mm
FoV phase	75.0 %
Slice thickness	8.0 mm
TR	378.98 ms
TE	1.07 ms
Averages	1
Concatenations	1
Filter	Raw filter, Distortion Corr.(2D)
Coil elements	BO2;SP2,3

Contrast - Common

TR	378.98 ms
TE	1.07 ms
Magn. preparation	Non-sel. IR
TI	260 ms
Flip angle	35 deg
Fat suppr.	None
Wrap-up Magn.	None

Contrast - Dynamic

Averages	1
Averaging mode	Short term
Reconstruction	Magn./Phase
Measurements	1
Multiple series	Off

Resolution - Common

FoV read	360 mm
FoV phase	75.0 %
Slice thickness	8.0 mm
Base resolution	192
Phase resolution	100 %
Phase partial Fourier	6/8
Trajectory	Cartesian
Interpolation	On

Resolution - iPAT

PAT mode	GRAPPA
Accel. factor PE	2
Ref. lines PE	24
Reference scan mode	Integrated

Resolution - Filter Image

Image Filter	Off
Distortion Corr.	On
Mode	2D
Unfiltered images	Off
Prescan Normalize	Off
Normalize	Off
B1 filter	Off

Resolution - Filter Rawdata

Raw filter	On
Elliptical filter	Off
POCS	Off

Geometry - Common

Slice group	1
Slices	1
Dist. factor	25 %
Position	Isocenter
Orientation	Transversal
Phase enc. dir.	A >> P
FoV read	360 mm
FoV phase	75.0 %
Slice thickness	8.0 mm
TR	378.98 ms
Multi-slice mode	Sequential
Series	Interleaved
Concatenations	1

Geometry - AutoAlign

Slice group	1
Position	Isocenter
Orientation	Transversal
Phase enc. dir.	A >> P
AutoAlign	---
Initial Position	Isocenter
Phase	0.0 mm
Read	0.0 mm
Shift	0.0 mm
Initial Rotation	0.00 deg
Initial Orientation	Transversal

Geometry - Saturation

Fat suppr.	None
Wrap-up Magn.	None
Special sat.	None

Geometry - Navigator

System - Miscellaneous

Positioning mode	FIX
Table position	H
Table position	0 mm

System - Miscellaneous

MSMA	S - C - T
Sagittal	R >> L
Coronal	A >> P
Transversal	F >> H
Coil Combine Mode	Adaptive Combine
Save uncombined	Off
Matrix Optimization	Off
Coil Focus	Flat
AutoAlign	---
Coil Select Mode	Default

System - Adjustments

B0 Shim mode	Cardiac
Adjust with body coil	On
Confirm freq. adjustment	Off
Assume Dominant Fat	Off
Assume Silicone	Off
Adjustment Tolerance	Auto

System - Adjust Volume

! Position	Isocenter
! Orientation	Transversal
! Rotation	0.00 deg
! A >> P	150 mm
! R >> L	150 mm
! F >> H	150 mm
Reset	Off

System - Tx/Rx

Frequency 1H	63.683250 MHz
Correction factor	1
Gain	High
Img. Scale Cor.	1.000
Reset	Off
? Ref. amplitude 1H	0.000 V

Physio - Signal1

1st Signal/Mode	ECG/Trigger
Average cycle	787 ± 129 ms
Average cycle	No Signal ms
Captured cycle	787 ± 129 ms
Acquisition window	379 ms
Trigger pulse	1
Trigger delay	0 ms
TR	378.98 ms
Concatenations	1
Segments	84
Phases	1
Adaptive Triggering	Off

Physio - Cardiac

Tagging	None
Magn. preparation	Non-sel. IR
TI	260 ms
Fat suppr.	None
Dark blood	Off
FoV read	360 mm
FoV phase	75.0 %
Phase resolution	100 %
Cine	Off
Trajectory	Cartesian
Dummy heartbeats	0
Motion Correction	None

Physio - PACE

Resp. control	Off
Concatenations	1

Sequence - Part 1

Introduction	Off
Dimension	2D
Reordering	Linear
Asymmetric echo	Weak
Contrasts	1
Optimization	Min. TE TR
Multi-slice mode	Sequential
Sequence type	Trufi
Bandwidth	898 Hz/Px

Sequence - Part 2

Define	Shots
Shots per slice	1
Segments	84
Trufi delta freq.	0 Hz
RF pulse type	Fast
Gradient mode	Fast
Excitation	Slice-sel.
Flip angle mode	Constant
Cine	Off

Sequence - Assistant

Mode	Off
Allowed delay	0 s

\\USER\Cardiac Research Protocols\Rapi-STRESS T1\Rapi-STRESS T1 (V2)\T1Map_LongT1_TD0_HighHR

TA: 8.7 s PM: FIX Voxel size: 1.9×1.9×8.0 mmPAT: 2 Rel. SNR: 1.00 : tfi

Properties

Prio recon	Off
Load images to viewer	On
Inline movie	Off
Auto store images	On
Load images to stamp segments	On
Load images to graphic segments	On
Auto open inline display	Off
Auto close inline display	Off
Start measurement without further preparation	On
Wait for user to start	Off
Start measurements	Single measurement

Routine

Slice group	1
Slices	1
Dist. factor	20 %
Position	Isocenter
Orientation	Transversal
Phase enc. dir.	A >> P
AutoAlign	---
Phase oversampling	0 %
FoV read	360 mm
FoV phase	85.4 %
Slice thickness	8.0 mm
TR	263.55 ms
TE	1.01 ms
Averages	1
Concatenations	1
Filter	Distortion Corr.(2D)
Coil elements	BO2;SP2,3

Contrast - Common

TR	263.55 ms
TE	1.01 ms
Magn. preparation	Non-sel. IR T1map
TI	180 ms
Flip angle	35 deg
Fat suppr.	None
Wrap-up Magn.	None

Contrast - Dynamic

Averages	1
Averaging mode	Short term
Reconstruction	Magnitude
Measurements	1
Multiple series	Off

Resolution - Common

FoV read	360 mm
FoV phase	85.4 %
Slice thickness	8.0 mm
Base resolution	192
Phase resolution	80 %
Phase partial Fourier	7/8
Trajectory	Cartesian
Interpolation	Off

Resolution - iPAT

PAT mode	GRAPPA
Accel. factor PE	2
Ref. lines PE	36
Reference scan mode	GRE/separate

Resolution - Filter Image

Image Filter	Off
Distortion Corr.	On
Mode	2D
Unfiltered images	Off
Prescan Normalize	Off
Normalize	Off
B1 filter	Off

Resolution - Filter Rawdata

Raw filter	Off
Elliptical filter	Off
POCS	Off

Geometry - Common

Slice group	1
Slices	1
Dist. factor	20 %
Position	Isocenter
Orientation	Transversal
Phase enc. dir.	A >> P
FoV read	360 mm
FoV phase	85.4 %
Slice thickness	8.0 mm
TR	263.55 ms
Multi-slice mode	Sequential
Series	Base To Apex
Concatenations	1

Geometry - AutoAlign

Slice group	1
Position	Isocenter
Orientation	Transversal
Phase enc. dir.	A >> P
AutoAlign	---
Initial Position	Isocenter
Phase	0.0 mm
Read	0.0 mm
Shift	0.0 mm
Initial Rotation	0.00 deg
Initial Orientation	Transversal

Geometry - Saturation

Fat suppr.	None
Wrap-up Magn.	None
Special sat.	None

Geometry - Navigator

System - Miscellaneous

Positioning mode	FIX
Table position	H
Table position	0 mm

System - Miscellaneous

MSMA	S - C - T
Sagittal	R >> L
Coronal	A >> P
Transversal	F >> H
Coil Combine Mode	Adaptive Combine
Save uncombined	Off
Matrix Optimization	Off
Coil Focus	Flat
AutoAlign	---
Coil Select Mode	Default

System - Adjustments

B0 Shim mode	Cardiac
Adjust with body coil	Off
Confirm freq. adjustment	Off
Assume Dominant Fat	Off
Assume Silicone	Off
Adjustment Tolerance	Auto

System - Adjust Volume

! Position	Isocenter
! Orientation	Transversal
! Rotation	0.00 deg
! A >> P	150 mm
! R >> L	150 mm
! F >> H	150 mm
Reset	Off

System - Tx/Rx

Frequency 1H	63.683250 MHz
Correction factor	1
Gain	High
Img. Scale Cor.	1.000
Reset	Off
? Ref. amplitude 1H	0.000 V

Physio - Signal1

1st Signal/Mode	ECG/Trigger
Average cycle	787 ± 129 ms
Average cycle	No Signal ms
Captured cycle	787 ± 129 ms
Acquisition window	700 ms
Trigger pulse	1
Trigger delay	0 ms
TR	263.55 ms
Concatenations	1
Segments	66
Phases	1
Adaptive Triggering	Off

Physio - Cardiac

Tagging	None
Magn. preparation	Non-sel. IR T1map
TI	180 ms
Fat suppr.	None
Dark blood	Off
FoV read	360 mm
FoV phase	85.4 %
Phase resolution	80 %
Cine	Off
Trajectory	Cartesian
Dummy heartbeats	0
Motion Correction	Standard

Physio - PACE

Resp. control	Breath-hold
Concatenations	1

Sequence - Part 1

Introduction	Off
Dimension	2D
Reordering	Linear
Asymmetric echo	Weak
Contrasts	1
Optimization	Min. TE TR
Multi-slice mode	Sequential
Sequence type	Trufi
Bandwidth	1085 Hz/Px

Sequence - Part 2

Define	Shots
Shots per slice	1
Segments	66
Trufi delta freq.	0 Hz
RF pulse type	Fast
Gradient mode	Fast
Excitation	Slice-sel.
Flip angle mode	Constant
Cine	Off

Sequence - Assistant

Mode	Off
Allowed delay	0 s

\\USER\Cardiac Research Protocols\Rapi-STRESS T1\Rapi-STRESS T1 (V2)\ShMOLLI_192i_d11_nFilt (TD=0)

TA: 7.1 s PM: FIX Voxel size: 0.9×0.9×8.0 mmPAT: 2 Rel. SNR: 1.00 : tfi

Properties

Prio recon	Off
Load images to viewer	On
Inline movie	Off
Auto store images	On
Load images to stamp segments	Off
Load images to graphic segments	On
Auto open inline display	Off
Auto close inline display	Off
Start measurement without further preparation	On
Wait for user to start	Off
Start measurements	Single measurement

Routine

Slice group	1
Slices	1
Dist. factor	25 %
Position	Isocenter
Orientation	Transversal
Phase enc. dir.	A >> P
AutoAlign	---
Phase oversampling	0 %
FoV read	360 mm
FoV phase	75.0 %
Slice thickness	8.0 mm
TR	378.98 ms
TE	1.07 ms
Averages	1
Concatenations	1
Filter	Raw filter, Distortion Corr.(2D)
Coil elements	BO2;SP2,3

Contrast - Common

TR	378.98 ms
TE	1.07 ms
Magn. preparation	Non-sel. IR
TI	260 ms
Flip angle	35 deg
Fat suppr.	None
Wrap-up Magn.	None

Contrast - Dynamic

Averages	1
Averaging mode	Short term
Reconstruction	Magn./Phase
Measurements	1
Multiple series	Off

Resolution - Common

FoV read	360 mm
FoV phase	75.0 %
Slice thickness	8.0 mm
Base resolution	192
Phase resolution	100 %
Phase partial Fourier	6/8
Trajectory	Cartesian
Interpolation	On

Resolution - iPAT

PAT mode	GRAPPA
Accel. factor PE	2
Ref. lines PE	24
Reference scan mode	Integrated

Resolution - Filter Image

Image Filter	Off
Distortion Corr.	On
Mode	2D
Unfiltered images	Off
Prescan Normalize	Off
Normalize	Off
B1 filter	Off

Resolution - Filter Rawdata

Raw filter	On
Elliptical filter	Off
POCS	Off

Geometry - Common

Slice group	1
Slices	1
Dist. factor	25 %
Position	Isocenter
Orientation	Transversal
Phase enc. dir.	A >> P
FoV read	360 mm
FoV phase	75.0 %
Slice thickness	8.0 mm
TR	378.98 ms
Multi-slice mode	Sequential
Series	Interleaved
Concatenations	1

Geometry - AutoAlign

Slice group	1
Position	Isocenter
Orientation	Transversal
Phase enc. dir.	A >> P
AutoAlign	---
Initial Position	Isocenter
Phase	0.0 mm
Read	0.0 mm
Shift	0.0 mm
Initial Rotation	0.00 deg
Initial Orientation	Transversal

Geometry - Saturation

Fat suppr.	None
Wrap-up Magn.	None
Special sat.	None

Geometry - Navigator

System - Miscellaneous

Positioning mode	FIX
Table position	H
Table position	0 mm

System - Miscellaneous

MSMA	S - C - T
Sagittal	R >> L
Coronal	A >> P
Transversal	F >> H
Coil Combine Mode	Adaptive Combine
Save uncombined	Off
Matrix Optimization	Off
Coil Focus	Flat
AutoAlign	---
Coil Select Mode	Default

System - Adjustments

B0 Shim mode	Cardiac
Adjust with body coil	On
Confirm freq. adjustment	Off
Assume Dominant Fat	Off
Assume Silicone	Off
Adjustment Tolerance	Auto

System - Adjust Volume

! Position	Isocenter
! Orientation	Transversal
! Rotation	0.00 deg
! A >> P	150 mm
! R >> L	150 mm
! F >> H	150 mm
Reset	Off

System - Tx/Rx

Frequency 1H	63.683250 MHz
Correction factor	1
Gain	High
Img. Scale Cor.	1.000
Reset	Off
? Ref. amplitude 1H	0.000 V

Physio - Signal1

1st Signal/Mode	ECG/Trigger
Average cycle	787 ± 129 ms
Average cycle	No Signal ms
Captured cycle	787 ± 129 ms
Acquisition window	379 ms
Trigger pulse	1
Trigger delay	0 ms
TR	378.98 ms
Concatenations	1
Segments	84
Phases	1
Adaptive Triggering	Off

Physio - Cardiac

Tagging	None
Magn. preparation	Non-sel. IR
TI	260 ms
Fat suppr.	None
Dark blood	Off
FoV read	360 mm
FoV phase	75.0 %
Phase resolution	100 %
Cine	Off
Trajectory	Cartesian
Dummy heartbeats	0
Motion Correction	None

Physio - PACE

Resp. control	Off
Concatenations	1

Sequence - Part 1

Introduction	Off
Dimension	2D
Reordering	Linear
Asymmetric echo	Weak
Contrasts	1
Optimization	Min. TE TR
Multi-slice mode	Sequential
Sequence type	Trufi
Bandwidth	898 Hz/Px

Sequence - Part 2

Define	Shots
Shots per slice	1
Segments	84
Trufi delta freq.	0 Hz
RF pulse type	Fast
Gradient mode	Fast
Excitation	Slice-sel.
Flip angle mode	Constant
Cine	Off

Sequence - Assistant

Mode	Off
Allowed delay	0 s

\\USER\Cardiac Research Protocols\Rapi-STRESS T1\Rapi-STRESS T1 (V2)\STRESS SSFP_Perf_MBF_MBV_aif_r3 (KELLMAN)

TA: 9.4 s PM: REF Voxel size: 1.9×1.9×8.0 mmPAT: 3 Rel. SNR: 1.00 : tfi

Properties

Prio recon	Off
Load images to viewer	Off
Inline movie	Off
Auto store images	On
Load images to stamp segments	Off
Load images to graphic segments	On
Auto open inline display	Off
Auto close inline display	Off
Start measurement without further preparation	Off
Wait for user to start	On
Start measurements	Single measurement

Routine

Slice group	1
Slices	3
Dist. factor	100 %
Position	Isocenter
Orientation	Transversal
Phase enc. dir.	A >> P
AutoAlign	---
Phase oversampling	0 %
FoV read	360 mm
FoV phase	75.0 %
Slice thickness	8.0 mm
TR	142.00 ms
TE	1.04 ms
Averages	1
Concatenations	1
Filter	Distortion Corr.(2D)
Coil elements	BO1-3;SP3,4

Contrast - Common

TR	142.00 ms
TE	1.04 ms
Magn. preparation	Non-sel. SR perf
TI	105 ms
Flip angle	50 deg
Fat suppr.	Fat sat.
Wrap-up Magn.	None

Contrast - Dynamic

Averages	1
Averaging mode	Long term
Reconstruction	Magnitude
Measurements	12
Pause after meas. 1	0.0 s
Pause after meas. 2	0.0 s
Pause after meas. 3	0.0 s
Pause after meas. 4	0.0 s
Pause after meas. 5	0.0 s
Pause after meas. 6	0.0 s
Pause after meas. 7	0.0 s
Pause after meas. 8	0.0 s
Pause after meas. 9	0.0 s
Pause after meas. 10	0.0 s
Pause after meas. 11	0.0 s
Proton Dens. Maps	3
Multiple series	Off

Resolution - Common

FoV read	360 mm
FoV phase	75.0 %
Slice thickness	8.0 mm
Base resolution	192
Phase resolution	77 %
Phase partial Fourier	6/8
Trajectory	Cartesian
Interpolation	Off

Resolution - iPAT

PAT mode	GRAPPA
Accel. factor PE	3
Reference scan mode	T-PAT

Resolution - Filter Image

Image Filter	Off
Distortion Corr.	On
Mode	2D
Unfiltered images	Off
Prescan Normalize	Off
Normalize	Off
B1 filter	Off

Resolution - Filter Rawdata

Raw filter	Off
Elliptical filter	Off
POCS	Off

Geometry - Common

Slice group	1
Slices	3
Dist. factor	100 %
Position	Isocenter
Orientation	Transversal
Phase enc. dir.	A >> P
FoV read	360 mm
FoV phase	75.0 %
Slice thickness	8.0 mm
TR	142.00 ms
Multi-slice mode	Single shot
Series	Ascending
Concatenations	1

Geometry - AutoAlign

Slice group	1
Position	Isocenter
Orientation	Transversal
Phase enc. dir.	A >> P
AutoAlign	---
Initial Position	Isocenter
Phase	0.0 mm
Read	0.0 mm
Shift	0.0 mm
Initial Rotation	0.00 deg
Initial Orientation	Transversal

Geometry - Saturation

Fat suppr.	Fat sat.
Wrap-up Magn.	None

Geometry - Saturation

Special sat.	None

Geometry - Navigator

System - Miscellaneous

Positioning mode	REF
Table position	H
Table position	0 mm
MSMA	S - C - T
Sagittal	R >> L
Coronal	A >> P
Transversal	F >> H
Coil Combine Mode	Sum of Squares
Save uncombined	Off
Matrix Optimization	Off
Coil Focus	Flat
AutoAlign	---
Coil Select Mode	Default

System - Adjustments

B0 Shim mode	Cardiac
Adjust with body coil	On
Confirm freq. adjustment	Off
Assume Dominant Fat	Off
Assume Silicone	Off
Adjustment Tolerance	Auto

System - Adjust Volume

! Position	Isocenter
! Orientation	Transversal
! Rotation	0.00 deg
! A >> P	150 mm
! R >> L	150 mm
! F >> H	150 mm
Reset	Off

System - Tx/Rx

Frequency 1H	63.683250 MHz
Correction factor	1
Gain	High
Img. Scale Cor.	1.000
Reset	Off
? Ref. amplitude 1H	0.000 V

Physio - Signal1

1st Signal/Mode	ECG/Trigger
Average cycle	787 ± 129 ms
Average cycle	No Signal ms
Captured cycle	787 ± 129 ms
Acquisition window	493 ms
Trigger pulse	1
Trigger delay	0 ms
TR	142.00 ms
Concatenations	1
Segments	37
Phases	1
Adaptive Triggering	Off

Physio - Cardiac

Tagging	None
Magn. preparation	Non-sel. SR perf
TI	105 ms
Fat suppr.	Fat sat.

Physio - Cardiac

Dark blood	Off
FoV read	360 mm
FoV phase	75.0 %
Phase resolution	77 %
Cine	Off
Trajectory	Cartesian
Dummy heartbeats	0
Motion Correction	None

Physio - PACE

Resp. control	Off
Concatenations	1

Inline - Common

Subtract	Off
Measurements	12
StdDev	Off
Motion Correction	None
Save original images	On

Inline - Cardiac

Inline Evaluation	Off
Magn. preparation	Non-sel. SR perf
TE	1.04 ms
TR	142.00 ms
Motion Correction	None
Save original images	On

Inline - MIP

MIP-Sag	Off
MIP-Cor	Off
MIP-Tra	Off
MIP-Time	Off
Save original images	On

Inline - Composing

Inline Composing	Off
Distortion Corr.	On
Mode	2D
Unfiltered images	Off

Sequence - Part 1

Introduction	Off
Dimension	2D
Reordering	Linear
Asymmetric echo	Allowed
Optimization	Min. TE
Multi-slice mode	Single shot
Echo spacing	2.5 ms
Sequence type	Trufi
Bandwidth	1085 Hz/Px

Sequence - Part 2

Define	Shots
Shots per slice	1
EPI factor	1
Segments	37
Trufi delta freq.	0 Hz
RF pulse type	Fast
Gradient mode	Fast
Excitation	Slice-sel.
Flip angle mode	Constant
Cine	Off

Sequence - Special

AIF Images	On
AIF SR Preparation	SR_PERF
Scan	Test
Gadgetron IPR	PERF2
Temporal Filter	On
Filter Method	Gaussian
Filter Strength	Medium

Sequence - Assistant

Mode	Min flip angle
Min flip angle	45 deg
Allowed delay	100 s

\\USER\Cardiac Research Protocols\Rapi-STRESS T1\Rapi-STRESS T1 (V2)\MidShMOLLI_192i_d11_nFilt (TD=0)

TA: 7.1 s PM: REF Voxel size: 0.9×0.9×8.0 mmPAT: 2 Rel. SNR: 1.00 : tfi

Properties

Prio recon	Off
Load images to viewer	On
Inline movie	Off
Auto store images	On
Load images to stamp segments	Off
Load images to graphic segments	On
Auto open inline display	Off
Auto close inline display	Off
Start measurement without further preparation	Off
Wait for user to start	Off
Start measurements	Single measurement

Routine

Slice group	1
Slices	1
Dist. factor	25 %
Position	Isocenter
Orientation	Transversal
Phase enc. dir.	A >> P
AutoAlign	---
Phase oversampling	0 %
FoV read	360 mm
FoV phase	75.0 %
Slice thickness	8.0 mm
TR	378.98 ms
TE	1.07 ms
Averages	1
Concatenations	1
Filter	Raw filter, Distortion Corr.(2D)
Coil elements	BO2;SP2,3

Contrast - Common

TR	378.98 ms
TE	1.07 ms
Magn. preparation	Non-sel. IR
TI	260 ms
Flip angle	35 deg
Fat suppr.	None
Wrap-up Magn.	None

Contrast - Dynamic

Averages	1
Averaging mode	Short term
Reconstruction	Magn./Phase
Measurements	1
Multiple series	Off

Resolution - Common

FoV read	360 mm
FoV phase	75.0 %
Slice thickness	8.0 mm
Base resolution	192
Phase resolution	100 %
Phase partial Fourier	6/8
Trajectory	Cartesian
Interpolation	On

Resolution - iPAT

PAT mode	GRAPPA
Accel. factor PE	2
Ref. lines PE	24
Reference scan mode	Integrated

Resolution - Filter Image

Image Filter	Off
Distortion Corr.	On
Mode	2D
Unfiltered images	Off
Prescan Normalize	Off
Normalize	Off
B1 filter	Off

Resolution - Filter Rawdata

Raw filter	On
Elliptical filter	Off
POCS	Off

Geometry - Common

Slice group	1
Slices	1
Dist. factor	25 %
Position	Isocenter
Orientation	Transversal
Phase enc. dir.	A >> P
FoV read	360 mm
FoV phase	75.0 %
Slice thickness	8.0 mm
TR	378.98 ms
Multi-slice mode	Sequential
Series	Interleaved
Concatenations	1

Geometry - AutoAlign

Slice group	1
Position	Isocenter
Orientation	Transversal
Phase enc. dir.	A >> P
AutoAlign	---
Initial Position	Isocenter
Phase	0.0 mm
Read	0.0 mm
Shift	0.0 mm
Initial Rotation	0.00 deg
Initial Orientation	Transversal

Geometry - Saturation

Fat suppr.	None
Wrap-up Magn.	None
Special sat.	None

Geometry - Navigator

System - Miscellaneous

Positioning mode	REF
Table position	H
Table position	0 mm

System - Miscellaneous

MSMA	S - C - T
Sagittal	R >> L
Coronal	A >> P
Transversal	F >> H
Coil Combine Mode	Adaptive Combine
Save uncombined	Off
Matrix Optimization	Off
Coil Focus	Flat
AutoAlign	---
Coil Select Mode	Default

System - Adjustments

B0 Shim mode	Cardiac
Adjust with body coil	On
Confirm freq. adjustment	Off
Assume Dominant Fat	Off
Assume Silicone	Off
Adjustment Tolerance	Auto

System - Adjust Volume

! Position	Isocenter
! Orientation	Transversal
! Rotation	0.00 deg
! A >> P	150 mm
! R >> L	150 mm
! F >> H	150 mm
Reset	Off

System - Tx/Rx

Frequency 1H	63.683250 MHz
Correction factor	1
Gain	High
Img. Scale Cor.	1.000
Reset	Off
? Ref. amplitude 1H	0.000 V

Physio - Signal1

1st Signal/Mode	ECG/Trigger
Average cycle	787 ± 129 ms
Average cycle	No Signal ms
Captured cycle	787 ± 129 ms
Acquisition window	379 ms
Trigger pulse	1
Trigger delay	0 ms
TR	378.98 ms
Concatenations	1
Segments	84
Phases	1
Adaptive Triggering	Off

Physio - Cardiac

Tagging	None
Magn. preparation	Non-sel. IR
TI	260 ms
Fat suppr.	None
Dark blood	Off
FoV read	360 mm
FoV phase	75.0 %
Phase resolution	100 %
Cine	Off
Trajectory	Cartesian
Dummy heartbeats	0
Motion Correction	None

Physio - PACE

Resp. control	Off
Concatenations	1

Sequence - Part 1

Introduction	Off
Dimension	2D
Reordering	Linear
Asymmetric echo	Weak
Contrasts	1
Optimization	Min. TE TR
Multi-slice mode	Sequential
Sequence type	Trufi
Bandwidth	898 Hz/Px

Sequence - Part 2

Define	Shots
Shots per slice	1
Segments	84
Trufi delta freq.	0 Hz
RF pulse type	Fast
Gradient mode	Fast
Excitation	Slice-sel.
Flip angle mode	Constant
Cine	Off

Sequence - Assistant

Mode	Off
Allowed delay	0 s

\\USER\Cardiac Research Protocols\Rapi-STRESS T1\Rapi-STRESS T1 (V2)\ShMOLLI_192i_d11_nFilt (TD=0)

TA: 7.1 s PM: FIX Voxel size: 0.9×0.9×8.0 mmPAT: 2 Rel. SNR: 1.00 : tfi

Properties

Prio recon	Off
Load images to viewer	On
Inline movie	Off
Auto store images	On
Load images to stamp segments	Off
Load images to graphic segments	On
Auto open inline display	Off
Auto close inline display	Off
Start measurement without further preparation	On
Wait for user to start	Off
Start measurements	Single measurement

Routine

Slice group	1
Slices	1
Dist. factor	25 %
Position	Isocenter
Orientation	Transversal
Phase enc. dir.	A >> P
AutoAlign	---
Phase oversampling	0 %
FoV read	360 mm
FoV phase	75.0 %
Slice thickness	8.0 mm
TR	378.98 ms
TE	1.07 ms
Averages	1
Concatenations	1
Filter	Raw filter, Distortion Corr.(2D)
Coil elements	BO2;SP2,3

Contrast - Common

TR	378.98 ms
TE	1.07 ms
Magn. preparation	Non-sel. IR
TI	260 ms
Flip angle	35 deg
Fat suppr.	None
Wrap-up Magn.	None

Contrast - Dynamic

Averages	1
Averaging mode	Short term
Reconstruction	Magn./Phase
Measurements	1
Multiple series	Off

Resolution - Common

FoV read	360 mm
FoV phase	75.0 %
Slice thickness	8.0 mm
Base resolution	192
Phase resolution	100 %
Phase partial Fourier	6/8
Trajectory	Cartesian
Interpolation	On

Resolution - iPAT

PAT mode	GRAPPA
Accel. factor PE	2
Ref. lines PE	24
Reference scan mode	Integrated

Resolution - Filter Image

Image Filter	Off
Distortion Corr.	On
Mode	2D
Unfiltered images	Off
Prescan Normalize	Off
Normalize	Off
B1 filter	Off

Resolution - Filter Rawdata

Raw filter	On
Elliptical filter	Off
POCS	Off

Geometry - Common

Slice group	1
Slices	1
Dist. factor	25 %
Position	Isocenter
Orientation	Transversal
Phase enc. dir.	A >> P
FoV read	360 mm
FoV phase	75.0 %
Slice thickness	8.0 mm
TR	378.98 ms
Multi-slice mode	Sequential
Series	Interleaved
Concatenations	1

Geometry - AutoAlign

Slice group	1
Position	Isocenter
Orientation	Transversal
Phase enc. dir.	A >> P
AutoAlign	---
Initial Position	Isocenter
Phase	0.0 mm
Read	0.0 mm
Shift	0.0 mm
Initial Rotation	0.00 deg
Initial Orientation	Transversal

Geometry - Saturation

Fat suppr.	None
Wrap-up Magn.	None
Special sat.	None

Geometry - Navigator

System - Miscellaneous

Positioning mode	FIX
Table position	H
Table position	0 mm

System - Miscellaneous

MSMA	S - C - T
Sagittal	R >> L
Coronal	A >> P
Transversal	F >> H
Coil Combine Mode	Adaptive Combine
Save uncombined	Off
Matrix Optimization	Off
Coil Focus	Flat
AutoAlign	---
Coil Select Mode	Default

System - Adjustments

B0 Shim mode	Cardiac
Adjust with body coil	On
Confirm freq. adjustment	Off
Assume Dominant Fat	Off
Assume Silicone	Off
Adjustment Tolerance	Auto

System - Adjust Volume

! Position	Isocenter
! Orientation	Transversal
! Rotation	0.00 deg
! A >> P	150 mm
! R >> L	150 mm
! F >> H	150 mm
Reset	Off

System - Tx/Rx

Frequency 1H	63.683250 MHz
Correction factor	1
Gain	High
Img. Scale Cor.	1.000
Reset	Off
? Ref. amplitude 1H	0.000 V

Physio - Signal1

1st Signal/Mode	ECG/Trigger
Average cycle	787 ± 129 ms
Average cycle	No Signal ms
Captured cycle	787 ± 129 ms
Acquisition window	379 ms
Trigger pulse	1
Trigger delay	0 ms
TR	378.98 ms
Concatenations	1
Segments	84
Phases	1
Adaptive Triggering	Off

Physio - Cardiac

Tagging	None
Magn. preparation	Non-sel. IR
TI	260 ms
Fat suppr.	None
Dark blood	Off
FoV read	360 mm
FoV phase	75.0 %
Phase resolution	100 %
Cine	Off
Trajectory	Cartesian
Dummy heartbeats	0
Motion Correction	None

Physio - PACE

Resp. control	Off
Concatenations	1

Sequence - Part 1

Introduction	Off
Dimension	2D
Reordering	Linear
Asymmetric echo	Weak
Contrasts	1
Optimization	Min. TE TR
Multi-slice mode	Sequential
Sequence type	Trufi
Bandwidth	898 Hz/Px

Sequence - Part 2

Define	Shots
Shots per slice	1
Segments	84
Trufi delta freq.	0 Hz
RF pulse type	Fast
Gradient mode	Fast
Excitation	Slice-sel.
Flip angle mode	Constant
Cine	Off

Sequence - Assistant

Mode	Off
Allowed delay	0 s

\\USER\Cardiac Research Protocols\Rapi-STRESS T1\Rapi-STRESS T1 (V2)\ShMOLLI_192i_d11_nFilt (TD=0)

TA: 7.1 s PM: FIX Voxel size: 0.9×0.9×8.0 mmPAT: 2 Rel. SNR: 1.00 : tfi

Properties

Prio recon	Off
Load images to viewer	On
Inline movie	Off
Auto store images	On
Load images to stamp segments	Off
Load images to graphic segments	On
Auto open inline display	Off
Auto close inline display	Off
Start measurement without further preparation	On
Wait for user to start	Off
Start measurements	Single measurement

Routine

Slice group	1
Slices	1
Dist. factor	25 %
Position	Isocenter
Orientation	Transversal
Phase enc. dir.	A >> P
AutoAlign	---
Phase oversampling	0 %
FoV read	360 mm
FoV phase	75.0 %
Slice thickness	8.0 mm
TR	378.98 ms
TE	1.07 ms
Averages	1
Concatenations	1
Filter	Raw filter, Distortion Corr.(2D)
Coil elements	BO2;SP2,3

Contrast - Common

TR	378.98 ms
TE	1.07 ms
Magn. preparation	Non-sel. IR
TI	260 ms
Flip angle	35 deg
Fat suppr.	None
Wrap-up Magn.	None

Contrast - Dynamic

Averages	1
Averaging mode	Short term
Reconstruction	Magn./Phase
Measurements	1
Multiple series	Off

Resolution - Common

FoV read	360 mm
FoV phase	75.0 %
Slice thickness	8.0 mm
Base resolution	192
Phase resolution	100 %
Phase partial Fourier	6/8
Trajectory	Cartesian
Interpolation	On

Resolution - iPAT

PAT mode	GRAPPA
Accel. factor PE	2
Ref. lines PE	24
Reference scan mode	Integrated

Resolution - Filter Image

Image Filter	Off
Distortion Corr.	On
Mode	2D
Unfiltered images	Off
Prescan Normalize	Off
Normalize	Off
B1 filter	Off

Resolution - Filter Rawdata

Raw filter	On
Elliptical filter	Off
POCS	Off

Geometry - Common

Slice group	1
Slices	1
Dist. factor	25 %
Position	Isocenter
Orientation	Transversal
Phase enc. dir.	A >> P
FoV read	360 mm
FoV phase	75.0 %
Slice thickness	8.0 mm
TR	378.98 ms
Multi-slice mode	Sequential
Series	Interleaved
Concatenations	1

Geometry - AutoAlign

Slice group	1
Position	Isocenter
Orientation	Transversal
Phase enc. dir.	A >> P
AutoAlign	---
Initial Position	Isocenter
Phase	0.0 mm
Read	0.0 mm
Shift	0.0 mm
Initial Rotation	0.00 deg
Initial Orientation	Transversal

Geometry - Saturation

Fat suppr.	None
Wrap-up Magn.	None
Special sat.	None

Geometry - Navigator

System - Miscellaneous

Positioning mode	FIX
Table position	H
Table position	0 mm

System - Miscellaneous

MSMA	S - C - T
Sagittal	R >> L
Coronal	A >> P
Transversal	F >> H
Coil Combine Mode	Adaptive Combine
Save uncombined	Off
Matrix Optimization	Off
Coil Focus	Flat
AutoAlign	---
Coil Select Mode	Default

System - Adjustments

B0 Shim mode	Cardiac
Adjust with body coil	On
Confirm freq. adjustment	Off
Assume Dominant Fat	Off
Assume Silicone	Off
Adjustment Tolerance	Auto

System - Adjust Volume

! Position	Isocenter
! Orientation	Transversal
! Rotation	0.00 deg
! A >> P	150 mm
! R >> L	150 mm
! F >> H	150 mm
Reset	Off

System - Tx/Rx

Frequency 1H	63.683250 MHz
Correction factor	1
Gain	High
Img. Scale Cor.	1.000
Reset	Off
? Ref. amplitude 1H	0.000 V

Physio - Signal1

1st Signal/Mode	ECG/Trigger
Average cycle	787 ± 129 ms
Average cycle	No Signal ms
Captured cycle	787 ± 129 ms
Acquisition window	379 ms
Trigger pulse	1
Trigger delay	0 ms
TR	378.98 ms
Concatenations	1
Segments	84
Phases	1
Adaptive Triggering	Off

Physio - Cardiac

Tagging	None
Magn. preparation	Non-sel. IR
TI	260 ms
Fat suppr.	None
Dark blood	Off
FoV read	360 mm
FoV phase	75.0 %
Phase resolution	100 %
Cine	Off
Trajectory	Cartesian
Dummy heartbeats	0
Motion Correction	None

Physio - PACE

Resp. control	Off
Concatenations	1

Sequence - Part 1

Introduction	Off
Dimension	2D
Reordering	Linear
Asymmetric echo	Weak
Contrasts	1
Optimization	Min. TE TR
Multi-slice mode	Sequential
Sequence type	Trufi
Bandwidth	898 Hz/Px

Sequence - Part 2

Define	Shots
Shots per slice	1
Segments	84
Trufi delta freq.	0 Hz
RF pulse type	Fast
Gradient mode	Fast
Excitation	Slice-sel.
Flip angle mode	Constant
Cine	Off

Sequence - Assistant

Mode	Off
Allowed delay	0 s

\\USER\Cardiac Research Protocols\Rapi-STRESS T1\Rapi-STRESS T1 (V2)\ShMOLLI_192i_d11_nFilt (TD=0)

TA: 7.1 s PM: FIX Voxel size: 0.9×0.9×8.0 mmPAT: 2 Rel. SNR: 1.00 : tfi

Properties

Prio recon	Off
Load images to viewer	On
Inline movie	Off
Auto store images	On
Load images to stamp segments	Off
Load images to graphic segments	On
Auto open inline display	Off
Auto close inline display	Off
Start measurement without further preparation	On
Wait for user to start	Off
Start measurements	Single measurement

Routine

Slice group	1
Slices	1
Dist. factor	25 %
Position	Isocenter
Orientation	Transversal
Phase enc. dir.	A >> P
AutoAlign	---
Phase oversampling	0 %
FoV read	360 mm
FoV phase	75.0 %
Slice thickness	8.0 mm
TR	378.98 ms
TE	1.07 ms
Averages	1
Concatenations	1
Filter	Raw filter, Distortion Corr.(2D)
Coil elements	BO2;SP2,3

Contrast - Common

TR	378.98 ms
TE	1.07 ms
Magn. preparation	Non-sel. IR
TI	260 ms
Flip angle	35 deg
Fat suppr.	None
Wrap-up Magn.	None

Contrast - Dynamic

Averages	1
Averaging mode	Short term
Reconstruction	Magn./Phase
Measurements	1
Multiple series	Off

Resolution - Common

FoV read	360 mm
FoV phase	75.0 %
Slice thickness	8.0 mm
Base resolution	192
Phase resolution	100 %
Phase partial Fourier	6/8
Trajectory	Cartesian
Interpolation	On

Resolution - iPAT

PAT mode	GRAPPA
Accel. factor PE	2
Ref. lines PE	24
Reference scan mode	Integrated

Resolution - Filter Image

Image Filter	Off
Distortion Corr.	On
Mode	2D
Unfiltered images	Off
Prescan Normalize	Off
Normalize	Off
B1 filter	Off

Resolution - Filter Rawdata

Raw filter	On
Elliptical filter	Off
POCS	Off

Geometry - Common

Slice group	1
Slices	1
Dist. factor	25 %
Position	Isocenter
Orientation	Transversal
Phase enc. dir.	A >> P
FoV read	360 mm
FoV phase	75.0 %
Slice thickness	8.0 mm
TR	378.98 ms
Multi-slice mode	Sequential
Series	Interleaved
Concatenations	1

Geometry - AutoAlign

Slice group	1
Position	Isocenter
Orientation	Transversal
Phase enc. dir.	A >> P
AutoAlign	---
Initial Position	Isocenter
Phase	0.0 mm
Read	0.0 mm
Shift	0.0 mm
Initial Rotation	0.00 deg
Initial Orientation	Transversal

Geometry - Saturation

Fat suppr.	None
Wrap-up Magn.	None
Special sat.	None

Geometry - Navigator

System - Miscellaneous

Positioning mode	FIX
Table position	H
Table position	0 mm

System - Miscellaneous

MSMA	S - C - T
Sagittal	R >> L
Coronal	A >> P
Transversal	F >> H
Coil Combine Mode	Adaptive Combine
Save uncombined	Off
Matrix Optimization	Off
Coil Focus	Flat
AutoAlign	---
Coil Select Mode	Default

System - Adjustments

B0 Shim mode	Cardiac
Adjust with body coil	On
Confirm freq. adjustment	Off
Assume Dominant Fat	Off
Assume Silicone	Off
Adjustment Tolerance	Auto

System - Adjust Volume

! Position	Isocenter
! Orientation	Transversal
! Rotation	0.00 deg
! A >> P	150 mm
! R >> L	150 mm
! F >> H	150 mm
Reset	Off

System - Tx/Rx

Frequency 1H	63.683250 MHz
Correction factor	1
Gain	High
Img. Scale Cor.	1.000
Reset	Off
? Ref. amplitude 1H	0.000 V

Physio - Signal1

1st Signal/Mode	ECG/Trigger
Average cycle	787 ± 129 ms
Average cycle	No Signal ms
Captured cycle	787 ± 129 ms
Acquisition window	379 ms
Trigger pulse	1
Trigger delay	0 ms
TR	378.98 ms
Concatenations	1
Segments	84
Phases	1
Adaptive Triggering	Off

Physio - Cardiac

Tagging	None
Magn. preparation	Non-sel. IR
TI	260 ms
Fat suppr.	None
Dark blood	Off
FoV read	360 mm
FoV phase	75.0 %
Phase resolution	100 %
Cine	Off
Trajectory	Cartesian
Dummy heartbeats	0
Motion Correction	None

Physio - PACE

Resp. control	Off
Concatenations	1

Sequence - Part 1

Introduction	Off
Dimension	2D
Reordering	Linear
Asymmetric echo	Weak
Contrasts	1
Optimization	Min. TE TR
Multi-slice mode	Sequential
Sequence type	Trufi
Bandwidth	898 Hz/Px

Sequence - Part 2

Define	Shots
Shots per slice	1
Segments	84
Trufi delta freq.	0 Hz
RF pulse type	Fast
Gradient mode	Fast
Excitation	Slice-sel.
Flip angle mode	Constant
Cine	Off

Sequence - Assistant

Mode	Off
Allowed delay	0 s

\\USER\Cardiac Research Protocols\Rapi-STRESS T1\Rapi-STRESS T1 (V2)\MidShMOLLI_192i_d11_nFilt (TD=0)

TA: 7.1 s PM: REF Voxel size: 0.9×0.9×8.0 mmPAT: 2 Rel. SNR: 1.00 : tfi

Properties

Prio recon	Off
Load images to viewer	On
Inline movie	Off
Auto store images	On
Load images to stamp segments	Off
Load images to graphic segments	On
Auto open inline display	Off
Auto close inline display	Off
Start measurement without further preparation	Off
Wait for user to start	Off
Start measurements	Single measurement

Routine

Slice group	1
Slices	1
Dist. factor	25 %
Position	Isocenter
Orientation	Transversal
Phase enc. dir.	A >> P
AutoAlign	---
Phase oversampling	0 %
FoV read	360 mm
FoV phase	75.0 %
Slice thickness	8.0 mm
TR	378.98 ms
TE	1.07 ms
Averages	1
Concatenations	1
Filter	Raw filter, Distortion Corr.(2D)
Coil elements	BO2;SP2,3

Contrast - Common

TR	378.98 ms
TE	1.07 ms
Magn. preparation	Non-sel. IR
TI	260 ms
Flip angle	35 deg
Fat suppr.	None
Wrap-up Magn.	None

Contrast - Dynamic

Averages	1
Averaging mode	Short term
Reconstruction	Magn./Phase
Measurements	1
Multiple series	Off

Resolution - Common

FoV read	360 mm
FoV phase	75.0 %
Slice thickness	8.0 mm
Base resolution	192
Phase resolution	100 %
Phase partial Fourier	6/8
Trajectory	Cartesian
Interpolation	On

Resolution - iPAT

PAT mode	GRAPPA
Accel. factor PE	2
Ref. lines PE	24
Reference scan mode	Integrated

Resolution - Filter Image

Image Filter	Off
Distortion Corr.	On
Mode	2D
Unfiltered images	Off
Prescan Normalize	Off
Normalize	Off
B1 filter	Off

Resolution - Filter Rawdata

Raw filter	On
Elliptical filter	Off
POCS	Off

Geometry - Common

Slice group	1
Slices	1
Dist. factor	25 %
Position	Isocenter
Orientation	Transversal
Phase enc. dir.	A >> P
FoV read	360 mm
FoV phase	75.0 %
Slice thickness	8.0 mm
TR	378.98 ms
Multi-slice mode	Sequential
Series	Interleaved
Concatenations	1

Geometry - AutoAlign

Slice group	1
Position	Isocenter
Orientation	Transversal
Phase enc. dir.	A >> P
AutoAlign	---
Initial Position	Isocenter
Phase	0.0 mm
Read	0.0 mm
Shift	0.0 mm
Initial Rotation	0.00 deg
Initial Orientation	Transversal

Geometry - Saturation

Fat suppr.	None
Wrap-up Magn.	None
Special sat.	None

Geometry - Navigator

System - Miscellaneous

Positioning mode	REF
Table position	H
Table position	0 mm

System - Miscellaneous

MSMA	S - C - T
Sagittal	R >> L
Coronal	A >> P
Transversal	F >> H
Coil Combine Mode	Adaptive Combine
Save uncombined	Off
Matrix Optimization	Off
Coil Focus	Flat
AutoAlign	---
Coil Select Mode	Default

System - Adjustments

B0 Shim mode	Cardiac
Adjust with body coil	On
Confirm freq. adjustment	Off
Assume Dominant Fat	Off
Assume Silicone	Off
Adjustment Tolerance	Auto

System - Adjust Volume

! Position	Isocenter
! Orientation	Transversal
! Rotation	0.00 deg
! A >> P	150 mm
! R >> L	150 mm
! F >> H	150 mm
Reset	Off

System - Tx/Rx

Frequency 1H	63.683250 MHz
Correction factor	1
Gain	High
Img. Scale Cor.	1.000
Reset	Off
? Ref. amplitude 1H	0.000 V

Physio - Signal1

1st Signal/Mode	ECG/Trigger
Average cycle	787 ± 129 ms
Average cycle	No Signal ms
Captured cycle	787 ± 129 ms
Acquisition window	379 ms
Trigger pulse	1
Trigger delay	0 ms
TR	378.98 ms
Concatenations	1
Segments	84
Phases	1
Adaptive Triggering	Off

Physio - Cardiac

Tagging	None
Magn. preparation	Non-sel. IR
TI	260 ms
Fat suppr.	None
Dark blood	Off
FoV read	360 mm
FoV phase	75.0 %
Phase resolution	100 %
Cine	Off
Trajectory	Cartesian
Dummy heartbeats	0
Motion Correction	None

Physio - PACE

Resp. control	Off
Concatenations	1

Sequence - Part 1

Introduction	Off
Dimension	2D
Reordering	Linear
Asymmetric echo	Weak
Contrasts	1
Optimization	Min. TE TR
Multi-slice mode	Sequential
Sequence type	Trufi
Bandwidth	898 Hz/Px

Sequence - Part 2

Define	Shots
Shots per slice	1
Segments	84
Trufi delta freq.	0 Hz
RF pulse type	Fast
Gradient mode	Fast
Excitation	Slice-sel.
Flip angle mode	Constant
Cine	Off

Sequence - Assistant

Mode	Off
Allowed delay	0 s

\\USER\Cardiac Research Protocols\Rapi-STRESS T1\Rapi-STRESS T1 (V2)\T1Map_ShortT1(TD=0)lowHR

TA: 0:17 PM: FIX Voxel size: 1.4×1.4×8.0 mmPAT: 2 Rel. SNR: 1.00 : tfi

Properties

Prio recon	Off
Load images to viewer	On
Inline movie	Off
Auto store images	On
Load images to stamp segments	On
Load images to graphic segments	On
Auto open inline display	Off
Auto close inline display	Off
Start measurement without further preparation	On
Wait for user to start	Off
Start measurements	Single measurement

Routine

Slice group	1
Slices	1
Dist. factor	20 %
Position	Isocenter
Orientation	Transversal
Phase enc. dir.	A >> P
AutoAlign	---
Phase oversampling	0 %
FoV read	360 mm
FoV phase	85.2 %
Slice thickness	8.0 mm
TR	359.84 ms
TE	1.13 ms
Averages	1
Concatenations	1
Filter	Distortion Corr.(2D)
Coil elements	BO2;SP2,3

Contrast - Common

TR	359.84 ms
TE	1.13 ms
Magn. preparation	Non-sel. IR T1map
TI	260 ms
Flip angle	35 deg
Fat suppr.	None
Wrap-up Magn.	None

Contrast - Dynamic

Averages	1
Averaging mode	Short term
Reconstruction	Magnitude
Measurements	1
Multiple series	Off

Resolution - Common

FoV read	360 mm
FoV phase	85.2 %
Slice thickness	8.0 mm
Base resolution	256
Phase resolution	66 %
Phase partial Fourier	7/8
Trajectory	Cartesian
Interpolation	Off

Resolution - iPAT

PAT mode	GRAPPA
Accel. factor PE	2
Ref. lines PE	36
Reference scan mode	GRE/separate

Resolution - Filter Image

Image Filter	Off
Distortion Corr.	On
Mode	2D
Unfiltered images	Off
Prescan Normalize	Off
Normalize	Off
B1 filter	Off

Resolution - Filter Rawdata

Raw filter	Off
Elliptical filter	Off
POCS	Off

Geometry - Common

Slice group	1
Slices	1
Dist. factor	20 %
Position	Isocenter
Orientation	Transversal
Phase enc. dir.	A >> P
FoV read	360 mm
FoV phase	85.2 %
Slice thickness	8.0 mm
TR	359.84 ms
Multi-slice mode	Sequential
Series	Base To Apex
Concatenations	1

Geometry - AutoAlign

Slice group	1
Position	Isocenter
Orientation	Transversal
Phase enc. dir.	A >> P
AutoAlign	---
Initial Position	Isocenter
Phase	0.0 mm
Read	0.0 mm
Shift	0.0 mm
Initial Rotation	0.00 deg
Initial Orientation	Transversal

Geometry - Saturation

Fat suppr.	None
Wrap-up Magn.	None
Special sat.	None

Geometry - Navigator

System - Miscellaneous

Positioning mode	FIX
Table position	H
Table position	0 mm

System - Miscellaneous

MSMA	S - C - T
Sagittal	R >> L
Coronal	A >> P
Transversal	F >> H
Coil Combine Mode	Adaptive Combine
Save uncombined	Off
Matrix Optimization	Off
Coil Focus	Flat
AutoAlign	---
Coil Select Mode	Default

System - Adjustments

B0 Shim mode	Cardiac
Adjust with body coil	Off
Confirm freq. adjustment	Off
Assume Dominant Fat	Off
Assume Silicone	Off
Adjustment Tolerance	Auto

System - Adjust Volume

! Position	Isocenter
! Orientation	Transversal
! Rotation	0.00 deg
! A >> P	150 mm
! R >> L	150 mm
! F >> H	150 mm
Reset	Off

System - Tx/Rx

Frequency 1H	63.683250 MHz
Correction factor	1
Gain	High
Img. Scale Cor.	1.000
Reset	Off
? Ref. amplitude 1H	0.000 V

Physio - Signal1

1st Signal/Mode	ECG/Trigger
Average cycle	787 ± 129 ms
Average cycle	No Signal ms
Captured cycle	787 ± 129 ms
Acquisition window	813 ms
Trigger pulse	1
Trigger delay	0 ms
TR	359.84 ms
Concatenations	1
Segments	72
Phases	1
Adaptive Triggering	Off

Physio - Cardiac

Tagging	None
Magn. preparation	Non-sel. IR T1map
TI	260 ms
Fat suppr.	None
Dark blood	Off
FoV read	360 mm
FoV phase	85.2 %
Phase resolution	66 %
Cine	Off
Trajectory	Cartesian
Dummy heartbeats	0
Motion Correction	Standard

Physio - PACE

Resp. control	Breath-hold
Concatenations	1

Sequence - Part 1

Introduction	Off
Dimension	2D
Reordering	Linear
Asymmetric echo	Weak
Contrasts	1
Optimization	Min. TE TR
Multi-slice mode	Sequential
Sequence type	Trufi
Bandwidth	1085 Hz/Px

Sequence - Part 2

Define	Shots
Shots per slice	1
Segments	72
Trufi delta freq.	0 Hz
RF pulse type	Fast
Gradient mode	Fast
Excitation	Slice-sel.
Flip angle mode	Constant
Cine	Off

Sequence - Assistant

Mode	Off
Allowed delay	0 s

\\USER\Cardiac Research Protocols\Rapi-STRESS T1\Rapi-STRESS T1 (V2)\ShMOLLI_192i_d11_nFilt (TD=0)

TA: 7.1 s PM: FIX Voxel size: 0.9×0.9×8.0 mmPAT: 2 Rel. SNR: 1.00 : tfi

Properties

Prio recon	Off
Load images to viewer	On
Inline movie	Off
Auto store images	On
Load images to stamp segments	Off
Load images to graphic segments	On
Auto open inline display	Off
Auto close inline display	Off
Start measurement without further preparation	On
Wait for user to start	Off
Start measurements	Single measurement

Routine

Slice group	1
Slices	1
Dist. factor	25 %
Position	Isocenter
Orientation	Transversal
Phase enc. dir.	A >> P
AutoAlign	---
Phase oversampling	0 %
FoV read	360 mm
FoV phase	75.0 %
Slice thickness	8.0 mm
TR	378.98 ms
TE	1.07 ms
Averages	1
Concatenations	1
Filter	Raw filter, Distortion Corr.(2D)
Coil elements	BO2;SP2,3

Contrast - Common

TR	378.98 ms
TE	1.07 ms
Magn. preparation	Non-sel. IR
TI	260 ms
Flip angle	35 deg
Fat suppr.	None
Wrap-up Magn.	None

Contrast - Dynamic

Averages	1
Averaging mode	Short term
Reconstruction	Magn./Phase
Measurements	1
Multiple series	Off

Resolution - Common

FoV read	360 mm
FoV phase	75.0 %
Slice thickness	8.0 mm
Base resolution	192
Phase resolution	100 %
Phase partial Fourier	6/8
Trajectory	Cartesian
Interpolation	On

Resolution - iPAT

PAT mode	GRAPPA
Accel. factor PE	2
Ref. lines PE	24
Reference scan mode	Integrated

Resolution - Filter Image

Image Filter	Off
Distortion Corr.	On
Mode	2D
Unfiltered images	Off
Prescan Normalize	Off
Normalize	Off
B1 filter	Off

Resolution - Filter Rawdata

Raw filter	On
Elliptical filter	Off
POCS	Off

Geometry - Common

Slice group	1
Slices	1
Dist. factor	25 %
Position	Isocenter
Orientation	Transversal
Phase enc. dir.	A >> P
FoV read	360 mm
FoV phase	75.0 %
Slice thickness	8.0 mm
TR	378.98 ms
Multi-slice mode	Sequential
Series	Interleaved
Concatenations	1

Geometry - AutoAlign

Slice group	1
Position	Isocenter
Orientation	Transversal
Phase enc. dir.	A >> P
AutoAlign	---
Initial Position	Isocenter
Phase	0.0 mm
Read	0.0 mm
Shift	0.0 mm
Initial Rotation	0.00 deg
Initial Orientation	Transversal

Geometry - Saturation

Fat suppr.	None
Wrap-up Magn.	None
Special sat.	None

Geometry - Navigator

System - Miscellaneous

Positioning mode	FIX
Table position	H
Table position	0 mm

System - Miscellaneous

MSMA	S - C - T
Sagittal	R >> L
Coronal	A >> P
Transversal	F >> H
Coil Combine Mode	Adaptive Combine
Save uncombined	Off
Matrix Optimization	Off
Coil Focus	Flat
AutoAlign	---
Coil Select Mode	Default

System - Adjustments

B0 Shim mode	Cardiac
Adjust with body coil	On
Confirm freq. adjustment	Off
Assume Dominant Fat	Off
Assume Silicone	Off
Adjustment Tolerance	Auto

System - Adjust Volume

! Position	Isocenter
! Orientation	Transversal
! Rotation	0.00 deg
! A >> P	150 mm
! R >> L	150 mm
! F >> H	150 mm
Reset	Off

System - Tx/Rx

Frequency 1H	63.683250 MHz
Correction factor	1
Gain	High
Img. Scale Cor.	1.000
Reset	Off
? Ref. amplitude 1H	0.000 V

Physio - Signal1

1st Signal/Mode	ECG/Trigger
Average cycle	787 ± 129 ms
Average cycle	No Signal ms
Captured cycle	787 ± 129 ms
Acquisition window	379 ms
Trigger pulse	1
Trigger delay	0 ms
TR	378.98 ms
Concatenations	1
Segments	84
Phases	1
Adaptive Triggering	Off

Physio - Cardiac

Tagging	None
Magn. preparation	Non-sel. IR
TI	260 ms
Fat suppr.	None
Dark blood	Off
FoV read	360 mm
FoV phase	75.0 %
Phase resolution	100 %
Cine	Off
Trajectory	Cartesian
Dummy heartbeats	0
Motion Correction	None

Physio - PACE

Resp. control	Off
Concatenations	1

Sequence - Part 1

Introduction	Off
Dimension	2D
Reordering	Linear
Asymmetric echo	Weak
Contrasts	1
Optimization	Min. TE TR
Multi-slice mode	Sequential
Sequence type	Trufi
Bandwidth	898 Hz/Px

Sequence - Part 2

Define	Shots
Shots per slice	1
Segments	84
Trufi delta freq.	0 Hz
RF pulse type	Fast
Gradient mode	Fast
Excitation	Slice-sel.
Flip angle mode	Constant
Cine	Off

Sequence - Assistant

Mode	Off
Allowed delay	0 s

\\USER\Cardiac Research Protocols\Rapi-STRESS T1\Rapi-STRESS T1 (V2)\T1Map_ShortT1(TD=0)highHR

TA: 8.7 s PM: FIX Voxel size: 1.9×1.9×8.0 mmPAT: 2 Rel. SNR: 1.00 : tfi

Properties

Prio recon	Off
Load images to viewer	On
Inline movie	Off
Auto store images	On
Load images to stamp segments	On
Load images to graphic segments	On
Auto open inline display	Off
Auto close inline display	Off
Start measurement without further preparation	On
Wait for user to start	Off
Start measurements	Single measurement

Routine

Slice group	1
Slices	1
Dist. factor	20 %
Position	Isocenter
Orientation	Transversal
Phase enc. dir.	A >> P
AutoAlign	---
Phase oversampling	0 %
FoV read	360 mm
FoV phase	85.4 %
Slice thickness	8.0 mm
TR	341.12 ms
TE	1.01 ms
Averages	1
Concatenations	1
Filter	Distortion Corr.(2D)
Coil elements	BO2;SP2,3

Contrast - Common

TR	341.12 ms
TE	1.01 ms
Magn. preparation	Non-sel. IR T1map
TI	260 ms
Flip angle	35 deg
Fat suppr.	None
Wrap-up Magn.	None

Contrast - Dynamic

Averages	1
Averaging mode	Short term
Reconstruction	Magnitude
Measurements	1
Multiple series	Off

Resolution - Common

FoV read	360 mm
FoV phase	85.4 %
Slice thickness	8.0 mm
Base resolution	192
Phase resolution	78 %
Phase partial Fourier	7/8
Trajectory	Cartesian
Interpolation	Off

Resolution - iPAT

PAT mode	GRAPPA
Accel. factor PE	2
Ref. lines PE	36
Reference scan mode	GRE/separate

Resolution - Filter Image

Image Filter	Off
Distortion Corr.	On
Mode	2D
Unfiltered images	Off
Prescan Normalize	Off
Normalize	Off
B1 filter	Off

Resolution - Filter Rawdata

Raw filter	Off
Elliptical filter	Off
POCS	Off

Geometry - Common

Slice group	1
Slices	1
Dist. factor	20 %
Position	Isocenter
Orientation	Transversal
Phase enc. dir.	A >> P
FoV read	360 mm
FoV phase	85.4 %
Slice thickness	8.0 mm
TR	341.12 ms
Multi-slice mode	Sequential
Series	Base To Apex
Concatenations	1

Geometry - AutoAlign

Slice group	1
Position	Isocenter
Orientation	Transversal
Phase enc. dir.	A >> P
AutoAlign	---
Initial Position	Isocenter
Phase	0.0 mm
Read	0.0 mm
Shift	0.0 mm
Initial Rotation	0.00 deg
Initial Orientation	Transversal

Geometry - Saturation

Fat suppr.	None
Wrap-up Magn.	None
Special sat.	None

Geometry - Navigator

System - Miscellaneous

Positioning mode	FIX
Table position	H
Table position	0 mm

System - Miscellaneous

MSMA	S - C - T
Sagittal	R >> L
Coronal	A >> P
Transversal	F >> H
Coil Combine Mode	Adaptive Combine
Save uncombined	Off
Matrix Optimization	Off
Coil Focus	Flat
AutoAlign	---
Coil Select Mode	Default

System - Adjustments

B0 Shim mode	Cardiac
Adjust with body coil	Off
Confirm freq. adjustment	Off
Assume Dominant Fat	Off
Assume Silicone	Off
Adjustment Tolerance	Auto

System - Adjust Volume

! Position	Isocenter
! Orientation	Transversal
! Rotation	0.00 deg
! A >> P	150 mm
! R >> L	150 mm
! F >> H	150 mm
Reset	Off

System - Tx/Rx

Frequency 1H	63.683250 MHz
Correction factor	1
Gain	High
Img. Scale Cor.	1.000
Reset	Off
? Ref. amplitude 1H	0.000 V

Physio - Signal1

1st Signal/Mode	ECG/Trigger
Average cycle	787 ± 129 ms
Average cycle	No Signal ms
Captured cycle	787 ± 129 ms
Acquisition window	704 ms
Trigger pulse	1
Trigger delay	0 ms
TR	341.12 ms
Concatenations	1
Segments	64
Phases	1
Adaptive Triggering	Off

Physio - Cardiac

Tagging	None
Magn. preparation	Non-sel. IR T1map
TI	260 ms
Fat suppr.	None
Dark blood	Off
FoV read	360 mm
FoV phase	85.4 %
Phase resolution	78 %
Cine	Off
Trajectory	Cartesian
Dummy heartbeats	0
Motion Correction	Standard

Physio - PACE

Resp. control	Breath-hold
Concatenations	1

Sequence - Part 1

Introduction	Off
Dimension	2D
Reordering	Linear
Asymmetric echo	Weak
Contrasts	1
Optimization	Min. TE TR
Multi-slice mode	Sequential
Sequence type	Trufi
Bandwidth	1085 Hz/Px

Sequence - Part 2

Define	Shots
Shots per slice	1
Segments	64
Trufi delta freq.	0 Hz
RF pulse type	Fast
Gradient mode	Fast
Excitation	Slice-sel.
Flip angle mode	Constant
Cine	Off

Sequence - Assistant

Mode	Off
Allowed delay	0 s

\\USER\Cardiac Research Protocols\Rapi-STRESS T1\Rapi-STRESS T1 (V2)\ShMOLLI_192i_d11_nFilt (TD=0)

TA: 7.1 s PM: FIX Voxel size: 0.9×0.9×8.0 mmPAT: 2 Rel. SNR: 1.00 : tfi

Properties

Prio recon	Off
Load images to viewer	On
Inline movie	Off
Auto store images	On
Load images to stamp segments	Off
Load images to graphic segments	On
Auto open inline display	Off
Auto close inline display	Off
Start measurement without further preparation	On
Wait for user to start	Off
Start measurements	Single measurement

Routine

Slice group	1
Slices	1
Dist. factor	25 %
Position	Isocenter
Orientation	Transversal
Phase enc. dir.	A >> P
AutoAlign	---
Phase oversampling	0 %
FoV read	360 mm
FoV phase	75.0 %
Slice thickness	8.0 mm
TR	378.98 ms
TE	1.07 ms
Averages	1
Concatenations	1
Filter	Raw filter, Distortion Corr.(2D)
Coil elements	BO2;SP2,3

Contrast - Common

TR	378.98 ms
TE	1.07 ms
Magn. preparation	Non-sel. IR
TI	260 ms
Flip angle	35 deg
Fat suppr.	None
Wrap-up Magn.	None

Contrast - Dynamic

Averages	1
Averaging mode	Short term
Reconstruction	Magn./Phase
Measurements	1
Multiple series	Off

Resolution - Common

FoV read	360 mm
FoV phase	75.0 %
Slice thickness	8.0 mm
Base resolution	192
Phase resolution	100 %
Phase partial Fourier	6/8
Trajectory	Cartesian
Interpolation	On

Resolution - iPAT

PAT mode	GRAPPA
Accel. factor PE	2
Ref. lines PE	24
Reference scan mode	Integrated

Resolution - Filter Image

Image Filter	Off
Distortion Corr.	On
Mode	2D
Unfiltered images	Off
Prescan Normalize	Off
Normalize	Off
B1 filter	Off

Resolution - Filter Rawdata

Raw filter	On
Elliptical filter	Off
POCS	Off

Geometry - Common

Slice group	1
Slices	1
Dist. factor	25 %
Position	Isocenter
Orientation	Transversal
Phase enc. dir.	A >> P
FoV read	360 mm
FoV phase	75.0 %
Slice thickness	8.0 mm
TR	378.98 ms
Multi-slice mode	Sequential
Series	Interleaved
Concatenations	1

Geometry - AutoAlign

Slice group	1
Position	Isocenter
Orientation	Transversal
Phase enc. dir.	A >> P
AutoAlign	---
Initial Position	Isocenter
Phase	0.0 mm
Read	0.0 mm
Shift	0.0 mm
Initial Rotation	0.00 deg
Initial Orientation	Transversal

Geometry - Saturation

Fat suppr.	None
Wrap-up Magn.	None
Special sat.	None

Geometry - Navigator

System - Miscellaneous

Positioning mode	FIX
Table position	H
Table position	0 mm

System - Miscellaneous

MSMA	S - C - T
Sagittal	R >> L
Coronal	A >> P
Transversal	F >> H
Coil Combine Mode	Adaptive Combine
Save uncombined	Off
Matrix Optimization	Off
Coil Focus	Flat
AutoAlign	---
Coil Select Mode	Default

System - Adjustments

B0 Shim mode	Cardiac
Adjust with body coil	On
Confirm freq. adjustment	Off
Assume Dominant Fat	Off
Assume Silicone	Off
Adjustment Tolerance	Auto

System - Adjust Volume

! Position	Isocenter
! Orientation	Transversal
! Rotation	0.00 deg
! A >> P	150 mm
! R >> L	150 mm
! F >> H	150 mm
Reset	Off

System - Tx/Rx

Frequency 1H	63.683250 MHz
Correction factor	1
Gain	High
Img. Scale Cor.	1.000
Reset	Off
? Ref. amplitude 1H	0.000 V

Physio - Signal1

1st Signal/Mode	ECG/Trigger
Average cycle	787 ± 129 ms
Average cycle	No Signal ms
Captured cycle	787 ± 129 ms
Acquisition window	379 ms
Trigger pulse	1
Trigger delay	0 ms
TR	378.98 ms
Concatenations	1
Segments	84
Phases	1
Adaptive Triggering	Off

Physio - Cardiac

Tagging	None
Magn. preparation	Non-sel. IR
TI	260 ms
Fat suppr.	None
Dark blood	Off
FoV read	360 mm
FoV phase	75.0 %
Phase resolution	100 %
Cine	Off
Trajectory	Cartesian
Dummy heartbeats	0
Motion Correction	None

Physio - PACE

Resp. control	Off
Concatenations	1

Sequence - Part 1

Introduction	Off
Dimension	2D
Reordering	Linear
Asymmetric echo	Weak
Contrasts	1
Optimization	Min. TE TR
Multi-slice mode	Sequential
Sequence type	Trufi
Bandwidth	898 Hz/Px

Sequence - Part 2

Define	Shots
Shots per slice	1
Segments	84
Trufi delta freq.	0 Hz
RF pulse type	Fast
Gradient mode	Fast
Excitation	Slice-sel.
Flip angle mode	Constant
Cine	Off

Sequence - Assistant

Mode	Off
Allowed delay	0 s

\\USER\Cardiac Research Protocols\Rapi-STRESS T1\Rapi-STRESS T1 (V2)\MidShMOLLI_192i_d11_nFilt (TD=0)

TA: 7.1 s PM: REF Voxel size: 0.9×0.9×8.0 mmPAT: 2 Rel. SNR: 1.00 : tfi

Properties

Prio recon	Off
Load images to viewer	On
Inline movie	Off
Auto store images	On
Load images to stamp segments	Off
Load images to graphic segments	On
Auto open inline display	Off
Auto close inline display	Off
Start measurement without further preparation	Off
Wait for user to start	Off
Start measurements	Single measurement

Routine

Slice group	1
Slices	1
Dist. factor	25 %
Position	Isocenter
Orientation	Transversal
Phase enc. dir.	A >> P
AutoAlign	---
Phase oversampling	0 %
FoV read	360 mm
FoV phase	75.0 %
Slice thickness	8.0 mm
TR	378.98 ms
TE	1.07 ms
Averages	1
Concatenations	1
Filter	Raw filter, Distortion Corr.(2D)
Coil elements	BO2;SP2,3

Contrast - Common

TR	378.98 ms
TE	1.07 ms
Magn. preparation	Non-sel. IR
TI	260 ms
Flip angle	35 deg
Fat suppr.	None
Wrap-up Magn.	None

Contrast - Dynamic

Averages	1
Averaging mode	Short term
Reconstruction	Magn./Phase
Measurements	1
Multiple series	Off

Resolution - Common

FoV read	360 mm
FoV phase	75.0 %
Slice thickness	8.0 mm
Base resolution	192
Phase resolution	100 %
Phase partial Fourier	6/8
Trajectory	Cartesian
Interpolation	On

Resolution - iPAT

PAT mode	GRAPPA
Accel. factor PE	2
Ref. lines PE	24
Reference scan mode	Integrated

Resolution - Filter Image

Image Filter	Off
Distortion Corr.	On
Mode	2D
Unfiltered images	Off
Prescan Normalize	Off
Normalize	Off
B1 filter	Off

Resolution - Filter Rawdata

Raw filter	On
Elliptical filter	Off
POCS	Off

Geometry - Common

Slice group	1
Slices	1
Dist. factor	25 %
Position	Isocenter
Orientation	Transversal
Phase enc. dir.	A >> P
FoV read	360 mm
FoV phase	75.0 %
Slice thickness	8.0 mm
TR	378.98 ms
Multi-slice mode	Sequential
Series	Interleaved
Concatenations	1

Geometry - AutoAlign

Slice group	1
Position	Isocenter
Orientation	Transversal
Phase enc. dir.	A >> P
AutoAlign	---
Initial Position	Isocenter
Phase	0.0 mm
Read	0.0 mm
Shift	0.0 mm
Initial Rotation	0.00 deg
Initial Orientation	Transversal

Geometry - Saturation

Fat suppr.	None
Wrap-up Magn.	None
Special sat.	None

Geometry - Navigator

System - Miscellaneous

Positioning mode	REF
Table position	H
Table position	0 mm

System - Miscellaneous

MSMA	S - C - T
Sagittal	R >> L
Coronal	A >> P
Transversal	F >> H
Coil Combine Mode	Adaptive Combine
Save uncombined	Off
Matrix Optimization	Off
Coil Focus	Flat
AutoAlign	---
Coil Select Mode	Default

System - Adjustments

B0 Shim mode	Cardiac
Adjust with body coil	On
Confirm freq. adjustment	Off
Assume Dominant Fat	Off
Assume Silicone	Off
Adjustment Tolerance	Auto

System - Adjust Volume

! Position	Isocenter
! Orientation	Transversal
! Rotation	0.00 deg
! A >> P	150 mm
! R >> L	150 mm
! F >> H	150 mm
Reset	Off

System - Tx/Rx

Frequency 1H	63.683250 MHz
Correction factor	1
Gain	High
Img. Scale Cor.	1.000
Reset	Off
? Ref. amplitude 1H	0.000 V

Physio - Signal1

1st Signal/Mode	ECG/Trigger
Average cycle	787 ± 129 ms
Average cycle	No Signal ms
Captured cycle	787 ± 129 ms
Acquisition window	379 ms
Trigger pulse	1
Trigger delay	0 ms
TR	378.98 ms
Concatenations	1
Segments	84
Phases	1
Adaptive Triggering	Off

Physio - Cardiac

Tagging	None
Magn. preparation	Non-sel. IR
TI	260 ms
Fat suppr.	None
Dark blood	Off
FoV read	360 mm
FoV phase	75.0 %
Phase resolution	100 %
Cine	Off
Trajectory	Cartesian
Dummy heartbeats	0
Motion Correction	None

Physio - PACE

Resp. control	Off
Concatenations	1

Sequence - Part 1

Introduction	Off
Dimension	2D
Reordering	Linear
Asymmetric echo	Weak
Contrasts	1
Optimization	Min. TE TR
Multi-slice mode	Sequential
Sequence type	Trufi
Bandwidth	898 Hz/Px

Sequence - Part 2

Define	Shots
Shots per slice	1
Segments	84
Trufi delta freq.	0 Hz
RF pulse type	Fast
Gradient mode	Fast
Excitation	Slice-sel.
Flip angle mode	Constant
Cine	Off

Sequence - Assistant

Mode	Off
Allowed delay	0 s

\\USER\Cardiac Research Protocols\Rapi-STRESS T1\Rapi-STRESS T1 (V2)\T1Map_ShortT1(TD=0)lowHR

TA: 0:17 PM: FIX Voxel size: 1.4×1.4×8.0 mmPAT: 2 Rel. SNR: 1.00 : tfi

Properties

Prio recon	Off
Load images to viewer	On
Inline movie	Off
Auto store images	On
Load images to stamp segments	On
Load images to graphic segments	On
Auto open inline display	Off
Auto close inline display	Off
Start measurement without further preparation	On
Wait for user to start	Off
Start measurements	Single measurement

Routine

Slice group	1
Slices	1
Dist. factor	20 %
Position	Isocenter
Orientation	Transversal
Phase enc. dir.	A >> P
AutoAlign	---
Phase oversampling	0 %
FoV read	360 mm
FoV phase	85.2 %
Slice thickness	8.0 mm
TR	359.84 ms
TE	1.13 ms
Averages	1
Concatenations	1
Filter	Distortion Corr.(2D)
Coil elements	BO2;SP2,3

Contrast - Common

TR	359.84 ms
TE	1.13 ms
Magn. preparation	Non-sel. IR T1map
TI	260 ms
Flip angle	35 deg
Fat suppr.	None
Wrap-up Magn.	None

Contrast - Dynamic

Averages	1
Averaging mode	Short term
Reconstruction	Magnitude
Measurements	1
Multiple series	Off

Resolution - Common

FoV read	360 mm
FoV phase	85.2 %
Slice thickness	8.0 mm
Base resolution	256
Phase resolution	66 %
Phase partial Fourier	7/8
Trajectory	Cartesian
Interpolation	Off

Resolution - iPAT

PAT mode	GRAPPA
Accel. factor PE	2
Ref. lines PE	36
Reference scan mode	GRE/separate

Resolution - Filter Image

Image Filter	Off
Distortion Corr.	On
Mode	2D
Unfiltered images	Off
Prescan Normalize	Off
Normalize	Off
B1 filter	Off

Resolution - Filter Rawdata

Raw filter	Off
Elliptical filter	Off
POCS	Off

Geometry - Common

Slice group	1
Slices	1
Dist. factor	20 %
Position	Isocenter
Orientation	Transversal
Phase enc. dir.	A >> P
FoV read	360 mm
FoV phase	85.2 %
Slice thickness	8.0 mm
TR	359.84 ms
Multi-slice mode	Sequential
Series	Base To Apex
Concatenations	1

Geometry - AutoAlign

Slice group	1
Position	Isocenter
Orientation	Transversal
Phase enc. dir.	A >> P
AutoAlign	---
Initial Position	Isocenter
Phase	0.0 mm
Read	0.0 mm
Shift	0.0 mm
Initial Rotation	0.00 deg
Initial Orientation	Transversal

Geometry - Saturation

Fat suppr.	None
Wrap-up Magn.	None
Special sat.	None

Geometry - Navigator

System - Miscellaneous

Positioning mode	FIX
Table position	H
Table position	0 mm

System - Miscellaneous

MSMA	S - C - T
Sagittal	R >> L
Coronal	A >> P
Transversal	F >> H
Coil Combine Mode	Adaptive Combine
Save uncombined	Off
Matrix Optimization	Off
Coil Focus	Flat
AutoAlign	---
Coil Select Mode	Default

System - Adjustments

B0 Shim mode	Cardiac
Adjust with body coil	Off
Confirm freq. adjustment	Off
Assume Dominant Fat	Off
Assume Silicone	Off
Adjustment Tolerance	Auto

System - Adjust Volume

! Position	Isocenter
! Orientation	Transversal
! Rotation	0.00 deg
! A >> P	150 mm
! R >> L	150 mm
! F >> H	150 mm
Reset	Off

System - Tx/Rx

Frequency 1H	63.683250 MHz
Correction factor	1
Gain	High
Img. Scale Cor.	1.000
Reset	Off
? Ref. amplitude 1H	0.000 V

Physio - Signal1

1st Signal/Mode	ECG/Trigger
Average cycle	787 ± 129 ms
Average cycle	No Signal ms
Captured cycle	787 ± 129 ms
Acquisition window	813 ms
Trigger pulse	1
Trigger delay	0 ms
TR	359.84 ms
Concatenations	1
Segments	72
Phases	1
Adaptive Triggering	Off

Physio - Cardiac

Tagging	None
Magn. preparation	Non-sel. IR T1map
TI	260 ms
Fat suppr.	None
Dark blood	Off
FoV read	360 mm
FoV phase	85.2 %
Phase resolution	66 %
Cine	Off
Trajectory	Cartesian
Dummy heartbeats	0
Motion Correction	Standard

Physio - PACE

Resp. control	Breath-hold
Concatenations	1

Sequence - Part 1

Introduction	Off
Dimension	2D
Reordering	Linear
Asymmetric echo	Weak
Contrasts	1
Optimization	Min. TE TR
Multi-slice mode	Sequential
Sequence type	Trufi
Bandwidth	1085 Hz/Px

Sequence - Part 2

Define	Shots
Shots per slice	1
Segments	72
Trufi delta freq.	0 Hz
RF pulse type	Fast
Gradient mode	Fast
Excitation	Slice-sel.
Flip angle mode	Constant
Cine	Off

Sequence - Assistant

Mode	Off
Allowed delay	0 s

\\USER\Cardiac Research Protocols\Rapi-STRESS T1\Rapi-STRESS T1 (V2)\ShMOLLI_192i_d11_nFilt (TD=0)

TA: 7.1 s PM: FIX Voxel size: 0.9×0.9×8.0 mmPAT: 2 Rel. SNR: 1.00 : tfi

Properties

Prio recon	Off
Load images to viewer	On
Inline movie	Off
Auto store images	On
Load images to stamp segments	Off
Load images to graphic segments	On
Auto open inline display	Off
Auto close inline display	Off
Start measurement without further preparation	On
Wait for user to start	Off
Start measurements	Single measurement

Routine

Slice group	1
Slices	1
Dist. factor	25 %
Position	Isocenter
Orientation	Transversal
Phase enc. dir.	A >> P
AutoAlign	---
Phase oversampling	0 %
FoV read	360 mm
FoV phase	75.0 %
Slice thickness	8.0 mm
TR	378.98 ms
TE	1.07 ms
Averages	1
Concatenations	1
Filter	Raw filter, Distortion Corr.(2D)
Coil elements	BO2;SP2,3

Contrast - Common

TR	378.98 ms
TE	1.07 ms
Magn. preparation	Non-sel. IR
TI	260 ms
Flip angle	35 deg
Fat suppr.	None
Wrap-up Magn.	None

Contrast - Dynamic

Averages	1
Averaging mode	Short term
Reconstruction	Magn./Phase
Measurements	1
Multiple series	Off

Resolution - Common

FoV read	360 mm
FoV phase	75.0 %
Slice thickness	8.0 mm
Base resolution	192
Phase resolution	100 %
Phase partial Fourier	6/8
Trajectory	Cartesian
Interpolation	On

Resolution - iPAT

PAT mode	GRAPPA
Accel. factor PE	2
Ref. lines PE	24
Reference scan mode	Integrated

Resolution - Filter Image

Image Filter	Off
Distortion Corr.	On
Mode	2D
Unfiltered images	Off
Prescan Normalize	Off
Normalize	Off
B1 filter	Off

Resolution - Filter Rawdata

Raw filter	On
Elliptical filter	Off
POCS	Off

Geometry - Common

Slice group	1
Slices	1
Dist. factor	25 %
Position	Isocenter
Orientation	Transversal
Phase enc. dir.	A >> P
FoV read	360 mm
FoV phase	75.0 %
Slice thickness	8.0 mm
TR	378.98 ms
Multi-slice mode	Sequential
Series	Interleaved
Concatenations	1

Geometry - AutoAlign

Slice group	1
Position	Isocenter
Orientation	Transversal
Phase enc. dir.	A >> P
AutoAlign	---
Initial Position	Isocenter
Phase	0.0 mm
Read	0.0 mm
Shift	0.0 mm
Initial Rotation	0.00 deg
Initial Orientation	Transversal

Geometry - Saturation

Fat suppr.	None
Wrap-up Magn.	None
Special sat.	None

Geometry - Navigator

System - Miscellaneous

Positioning mode	FIX
Table position	H
Table position	0 mm

System - Miscellaneous

MSMA	S - C - T
Sagittal	R >> L
Coronal	A >> P
Transversal	F >> H
Coil Combine Mode	Adaptive Combine
Save uncombined	Off
Matrix Optimization	Off
Coil Focus	Flat
AutoAlign	---
Coil Select Mode	Default

System - Adjustments

B0 Shim mode	Cardiac
Adjust with body coil	On
Confirm freq. adjustment	Off
Assume Dominant Fat	Off
Assume Silicone	Off
Adjustment Tolerance	Auto

System - Adjust Volume

! Position	Isocenter
! Orientation	Transversal
! Rotation	0.00 deg
! A >> P	150 mm
! R >> L	150 mm
! F >> H	150 mm
Reset	Off

System - Tx/Rx

Frequency 1H	63.683250 MHz
Correction factor	1
Gain	High
Img. Scale Cor.	1.000
Reset	Off
? Ref. amplitude 1H	0.000 V

Physio - Signal1

1st Signal/Mode	ECG/Trigger
Average cycle	787 ± 129 ms
Average cycle	No Signal ms
Captured cycle	787 ± 129 ms
Acquisition window	379 ms
Trigger pulse	1
Trigger delay	0 ms
TR	378.98 ms
Concatenations	1
Segments	84
Phases	1
Adaptive Triggering	Off

Physio - Cardiac

Tagging	None
Magn. preparation	Non-sel. IR
TI	260 ms
Fat suppr.	None
Dark blood	Off
FoV read	360 mm
FoV phase	75.0 %
Phase resolution	100 %
Cine	Off
Trajectory	Cartesian
Dummy heartbeats	0
Motion Correction	None

Physio - PACE

Resp. control	Off
Concatenations	1

Sequence - Part 1

Introduction	Off
Dimension	2D
Reordering	Linear
Asymmetric echo	Weak
Contrasts	1
Optimization	Min. TE TR
Multi-slice mode	Sequential
Sequence type	Trufi
Bandwidth	898 Hz/Px

Sequence - Part 2

Define	Shots
Shots per slice	1
Segments	84
Trufi delta freq.	0 Hz
RF pulse type	Fast
Gradient mode	Fast
Excitation	Slice-sel.
Flip angle mode	Constant
Cine	Off

Sequence - Assistant

Mode	Off
Allowed delay	0 s

\\USER\Cardiac Research Protocols\Rapi-STRESS T1\Rapi-STRESS T1 (V2)\T1Map_ShortT1(TD=0)highHR

TA: 8.7 s PM: FIX Voxel size: 1.9×1.9×8.0 mmPAT: 2 Rel. SNR: 1.00 : tfi

Properties

Prio recon	Off
Load images to viewer	On
Inline movie	Off
Auto store images	On
Load images to stamp segments	On
Load images to graphic segments	On
Auto open inline display	Off
Auto close inline display	Off
Start measurement without further preparation	On
Wait for user to start	Off
Start measurements	Single measurement

Routine

Slice group	1
Slices	1
Dist. factor	20 %
Position	Isocenter
Orientation	Transversal
Phase enc. dir.	A >> P
AutoAlign	---
Phase oversampling	0 %
FoV read	360 mm
FoV phase	85.4 %
Slice thickness	8.0 mm
TR	341.12 ms
TE	1.01 ms
Averages	1
Concatenations	1
Filter	Distortion Corr.(2D)
Coil elements	BO2;SP2,3

Contrast - Common

TR	341.12 ms
TE	1.01 ms
Magn. preparation	Non-sel. IR T1map
TI	260 ms
Flip angle	35 deg
Fat suppr.	None
Wrap-up Magn.	None

Contrast - Dynamic

Averages	1
Averaging mode	Short term
Reconstruction	Magnitude
Measurements	1
Multiple series	Off

Resolution - Common

FoV read	360 mm
FoV phase	85.4 %
Slice thickness	8.0 mm
Base resolution	192
Phase resolution	78 %
Phase partial Fourier	7/8
Trajectory	Cartesian
Interpolation	Off

Resolution - iPAT

PAT mode	GRAPPA
Accel. factor PE	2
Ref. lines PE	36
Reference scan mode	GRE/separate

Resolution - Filter Image

Image Filter	Off
Distortion Corr.	On
Mode	2D
Unfiltered images	Off
Prescan Normalize	Off
Normalize	Off
B1 filter	Off

Resolution - Filter Rawdata

Raw filter	Off
Elliptical filter	Off
POCS	Off

Geometry - Common

Slice group	1
Slices	1
Dist. factor	20 %
Position	Isocenter
Orientation	Transversal
Phase enc. dir.	A >> P
FoV read	360 mm
FoV phase	85.4 %
Slice thickness	8.0 mm
TR	341.12 ms
Multi-slice mode	Sequential
Series	Base To Apex
Concatenations	1

Geometry - AutoAlign

Slice group	1
Position	Isocenter
Orientation	Transversal
Phase enc. dir.	A >> P
AutoAlign	---
Initial Position	Isocenter
Phase	0.0 mm
Read	0.0 mm
Shift	0.0 mm
Initial Rotation	0.00 deg
Initial Orientation	Transversal

Geometry - Saturation

Fat suppr.	None
Wrap-up Magn.	None
Special sat.	None

Geometry - Navigator

System - Miscellaneous

Positioning mode	FIX
Table position	H
Table position	0 mm

System - Miscellaneous

MSMA	S - C - T
Sagittal	R >> L
Coronal	A >> P
Transversal	F >> H
Coil Combine Mode	Adaptive Combine
Save uncombined	Off
Matrix Optimization	Off
Coil Focus	Flat
AutoAlign	---
Coil Select Mode	Default

System - Adjustments

B0 Shim mode	Cardiac
Adjust with body coil	Off
Confirm freq. adjustment	Off
Assume Dominant Fat	Off
Assume Silicone	Off
Adjustment Tolerance	Auto

System - Adjust Volume

! Position	Isocenter
! Orientation	Transversal
! Rotation	0.00 deg
! A >> P	150 mm
! R >> L	150 mm
! F >> H	150 mm
Reset	Off

System - Tx/Rx

Frequency 1H	63.683250 MHz
Correction factor	1
Gain	High
Img. Scale Cor.	1.000
Reset	Off
? Ref. amplitude 1H	0.000 V

Physio - Signal1

1st Signal/Mode	ECG/Trigger
Average cycle	787 ± 129 ms
Average cycle	No Signal ms
Captured cycle	787 ± 129 ms
Acquisition window	704 ms
Trigger pulse	1
Trigger delay	0 ms
TR	341.12 ms
Concatenations	1
Segments	64
Phases	1
Adaptive Triggering	Off

Physio - Cardiac

Tagging	None
Magn. preparation	Non-sel. IR T1map
TI	260 ms
Fat suppr.	None
Dark blood	Off
FoV read	360 mm
FoV phase	85.4 %
Phase resolution	78 %
Cine	Off
Trajectory	Cartesian
Dummy heartbeats	0
Motion Correction	Standard

Physio - PACE

Resp. control	Breath-hold
Concatenations	1

Sequence - Part 1

Introduction	Off
Dimension	2D
Reordering	Linear
Asymmetric echo	Weak
Contrasts	1
Optimization	Min. TE TR
Multi-slice mode	Sequential
Sequence type	Trufi
Bandwidth	1085 Hz/Px

Sequence - Part 2

Define	Shots
Shots per slice	1
Segments	64
Trufi delta freq.	0 Hz
RF pulse type	Fast
Gradient mode	Fast
Excitation	Slice-sel.
Flip angle mode	Constant
Cine	Off

Sequence - Assistant

Mode	Off
Allowed delay	0 s

\\USER\Cardiac Research Protocols\Rapi-STRESS T1\Rapi-STRESS T1 (V2)\ShMOLLI_192i_d11_nFilt (TD=0)

TA: 7.1 s PM: FIX Voxel size: 0.9×0.9×8.0 mmPAT: 2 Rel. SNR: 1.00 : tfi

Properties

Prio recon	Off
Load images to viewer	On
Inline movie	Off
Auto store images	On
Load images to stamp segments	Off
Load images to graphic segments	On
Auto open inline display	Off
Auto close inline display	Off
Start measurement without further preparation	On
Wait for user to start	Off
Start measurements	Single measurement

Routine

Slice group	1
Slices	1
Dist. factor	25 %
Position	Isocenter
Orientation	Transversal
Phase enc. dir.	A >> P
AutoAlign	---
Phase oversampling	0 %
FoV read	360 mm
FoV phase	75.0 %
Slice thickness	8.0 mm
TR	378.98 ms
TE	1.07 ms
Averages	1
Concatenations	1
Filter	Raw filter, Distortion Corr.(2D)
Coil elements	BO2;SP2,3

Contrast - Common

TR	378.98 ms
TE	1.07 ms
Magn. preparation	Non-sel. IR
TI	260 ms
Flip angle	35 deg
Fat suppr.	None
Wrap-up Magn.	None

Contrast - Dynamic

Averages	1
Averaging mode	Short term
Reconstruction	Magn./Phase
Measurements	1
Multiple series	Off

Resolution - Common

FoV read	360 mm
FoV phase	75.0 %
Slice thickness	8.0 mm
Base resolution	192
Phase resolution	100 %
Phase partial Fourier	6/8
Trajectory	Cartesian
Interpolation	On

Resolution - iPAT

PAT mode	GRAPPA
Accel. factor PE	2
Ref. lines PE	24
Reference scan mode	Integrated

Resolution - Filter Image

Image Filter	Off
Distortion Corr.	On
Mode	2D
Unfiltered images	Off
Prescan Normalize	Off
Normalize	Off
B1 filter	Off

Resolution - Filter Rawdata

Raw filter	On
Elliptical filter	Off
POCS	Off

Geometry - Common

Slice group	1
Slices	1
Dist. factor	25 %
Position	Isocenter
Orientation	Transversal
Phase enc. dir.	A >> P
FoV read	360 mm
FoV phase	75.0 %
Slice thickness	8.0 mm
TR	378.98 ms
Multi-slice mode	Sequential
Series	Interleaved
Concatenations	1

Geometry - AutoAlign

Slice group	1
Position	Isocenter
Orientation	Transversal
Phase enc. dir.	A >> P
AutoAlign	---
Initial Position	Isocenter
Phase	0.0 mm
Read	0.0 mm
Shift	0.0 mm
Initial Rotation	0.00 deg
Initial Orientation	Transversal

Geometry - Saturation

Fat suppr.	None
Wrap-up Magn.	None
Special sat.	None

Geometry - Navigator

System - Miscellaneous

Positioning mode	FIX
Table position	H
Table position	0 mm

System - Miscellaneous

MSMA	S - C - T
Sagittal	R >> L
Coronal	A >> P
Transversal	F >> H
Coil Combine Mode	Adaptive Combine
Save uncombined	Off
Matrix Optimization	Off
Coil Focus	Flat
AutoAlign	---
Coil Select Mode	Default

System - Adjustments

B0 Shim mode	Cardiac
Adjust with body coil	On
Confirm freq. adjustment	Off
Assume Dominant Fat	Off
Assume Silicone	Off
Adjustment Tolerance	Auto

System - Adjust Volume

! Position	Isocenter
! Orientation	Transversal
! Rotation	0.00 deg
! A >> P	150 mm
! R >> L	150 mm
! F >> H	150 mm
Reset	Off

System - Tx/Rx

Frequency 1H	63.683250 MHz
Correction factor	1
Gain	High
Img. Scale Cor.	1.000
Reset	Off
? Ref. amplitude 1H	0.000 V

Physio - Signal1

1st Signal/Mode	ECG/Trigger
Average cycle	787 ± 129 ms
Average cycle	No Signal ms
Captured cycle	787 ± 129 ms
Acquisition window	379 ms
Trigger pulse	1
Trigger delay	0 ms
TR	378.98 ms
Concatenations	1
Segments	84
Phases	1
Adaptive Triggering	Off

Physio - Cardiac

Tagging	None
Magn. preparation	Non-sel. IR
TI	260 ms
Fat suppr.	None
Dark blood	Off
FoV read	360 mm
FoV phase	75.0 %
Phase resolution	100 %
Cine	Off
Trajectory	Cartesian
Dummy heartbeats	0
Motion Correction	None

Physio - PACE

Resp. control	Off
Concatenations	1

Sequence - Part 1

Introduction	Off
Dimension	2D
Reordering	Linear
Asymmetric echo	Weak
Contrasts	1
Optimization	Min. TE TR
Multi-slice mode	Sequential
Sequence type	Trufi
Bandwidth	898 Hz/Px

Sequence - Part 2

Define	Shots
Shots per slice	1
Segments	84
Trufi delta freq.	0 Hz
RF pulse type	Fast
Gradient mode	Fast
Excitation	Slice-sel.
Flip angle mode	Constant
Cine	Off

Sequence - Assistant

Mode	Off
Allowed delay	0 s

\\USER\Cardiac Research Protocols\Rapi-STRESS T1\Rapi-STRESS T1 (V2)\MidShMOLLI_192i_d11_nFilt (TD=0)

TA: 7.1 s PM: REF Voxel size: 0.9×0.9×8.0 mmPAT: 2 Rel. SNR: 1.00 : tfi

Properties

Prio recon	Off
Load images to viewer	On
Inline movie	Off
Auto store images	On
Load images to stamp segments	Off
Load images to graphic segments	On
Auto open inline display	Off
Auto close inline display	Off
Start measurement without further preparation	Off
Wait for user to start	Off
Start measurements	Single measurement

Routine

Slice group	1
Slices	1
Dist. factor	25 %
Position	Isocenter
Orientation	Transversal
Phase enc. dir.	A >> P
AutoAlign	---
Phase oversampling	0 %
FoV read	360 mm
FoV phase	75.0 %
Slice thickness	8.0 mm
TR	378.98 ms
TE	1.07 ms
Averages	1
Concatenations	1
Filter	Raw filter, Distortion Corr.(2D)
Coil elements	BO2;SP2,3

Contrast - Common

TR	378.98 ms
TE	1.07 ms
Magn. preparation	Non-sel. IR
TI	260 ms
Flip angle	35 deg
Fat suppr.	None
Wrap-up Magn.	None

Contrast - Dynamic

Averages	1
Averaging mode	Short term
Reconstruction	Magn./Phase
Measurements	1
Multiple series	Off

Resolution - Common

FoV read	360 mm
FoV phase	75.0 %
Slice thickness	8.0 mm
Base resolution	192
Phase resolution	100 %
Phase partial Fourier	6/8
Trajectory	Cartesian
Interpolation	On

Resolution - iPAT

PAT mode	GRAPPA
Accel. factor PE	2
Ref. lines PE	24
Reference scan mode	Integrated

Resolution - Filter Image

Image Filter	Off
Distortion Corr.	On
Mode	2D
Unfiltered images	Off
Prescan Normalize	Off
Normalize	Off
B1 filter	Off

Resolution - Filter Rawdata

Raw filter	On
Elliptical filter	Off
POCS	Off

Geometry - Common

Slice group	1
Slices	1
Dist. factor	25 %
Position	Isocenter
Orientation	Transversal
Phase enc. dir.	A >> P
FoV read	360 mm
FoV phase	75.0 %
Slice thickness	8.0 mm
TR	378.98 ms
Multi-slice mode	Sequential
Series	Interleaved
Concatenations	1

Geometry - AutoAlign

Slice group	1
Position	Isocenter
Orientation	Transversal
Phase enc. dir.	A >> P
AutoAlign	---
Initial Position	Isocenter
Phase	0.0 mm
Read	0.0 mm
Shift	0.0 mm
Initial Rotation	0.00 deg
Initial Orientation	Transversal

Geometry - Saturation

Fat suppr.	None
Wrap-up Magn.	None
Special sat.	None

Geometry - Navigator

System - Miscellaneous

Positioning mode	REF
Table position	H
Table position	0 mm

System - Miscellaneous

MSMA	S - C - T
Sagittal	R >> L
Coronal	A >> P
Transversal	F >> H
Coil Combine Mode	Adaptive Combine
Save uncombined	Off
Matrix Optimization	Off
Coil Focus	Flat
AutoAlign	---
Coil Select Mode	Default

System - Adjustments

B0 Shim mode	Cardiac
Adjust with body coil	On
Confirm freq. adjustment	Off
Assume Dominant Fat	Off
Assume Silicone	Off
Adjustment Tolerance	Auto

System - Adjust Volume

! Position	Isocenter
! Orientation	Transversal
! Rotation	0.00 deg
! A >> P	150 mm
! R >> L	150 mm
! F >> H	150 mm
Reset	Off

System - Tx/Rx

Frequency 1H	63.683250 MHz
Correction factor	1
Gain	High
Img. Scale Cor.	1.000
Reset	Off
? Ref. amplitude 1H	0.000 V

Physio - Signal1

1st Signal/Mode	ECG/Trigger
Average cycle	787 ± 129 ms
Average cycle	No Signal ms
Captured cycle	787 ± 129 ms
Acquisition window	379 ms
Trigger pulse	1
Trigger delay	0 ms
TR	378.98 ms
Concatenations	1
Segments	84
Phases	1
Adaptive Triggering	Off

Physio - Cardiac

Tagging	None
Magn. preparation	Non-sel. IR
TI	260 ms
Fat suppr.	None
Dark blood	Off
FoV read	360 mm
FoV phase	75.0 %
Phase resolution	100 %
Cine	Off
Trajectory	Cartesian
Dummy heartbeats	0
Motion Correction	None

Physio - PACE

Resp. control	Off
Concatenations	1

Sequence - Part 1

Introduction	Off
Dimension	2D
Reordering	Linear
Asymmetric echo	Weak
Contrasts	1
Optimization	Min. TE TR
Multi-slice mode	Sequential
Sequence type	Trufi
Bandwidth	898 Hz/Px

Sequence - Part 2

Define	Shots
Shots per slice	1
Segments	84
Trufi delta freq.	0 Hz
RF pulse type	Fast
Gradient mode	Fast
Excitation	Slice-sel.
Flip angle mode	Constant
Cine	Off

Sequence - Assistant

Mode	Off
Allowed delay	0 s

\\USER\Cardiac Research Protocols\Rapi-STRESS T1\Rapi-STRESS T1 (V2)\T1Map_ShortT1(TD=0)lowHR

TA: 0:17 PM: FIX Voxel size: 1.4×1.4×8.0 mmPAT: 2 Rel. SNR: 1.00 : tfi

Properties

Prio recon	Off
Load images to viewer	On
Inline movie	Off
Auto store images	On
Load images to stamp segments	On
Load images to graphic segments	On
Auto open inline display	Off
Auto close inline display	Off
Start measurement without further preparation	On
Wait for user to start	Off
Start measurements	Single measurement

Routine

Slice group	1
Slices	1
Dist. factor	20 %
Position	Isocenter
Orientation	Transversal
Phase enc. dir.	A >> P
AutoAlign	---
Phase oversampling	0 %
FoV read	360 mm
FoV phase	85.2 %
Slice thickness	8.0 mm
TR	359.84 ms
TE	1.13 ms
Averages	1
Concatenations	1
Filter	Distortion Corr.(2D)
Coil elements	BO2;SP2,3

Contrast - Common

TR	359.84 ms
TE	1.13 ms
Magn. preparation	Non-sel. IR T1map
TI	260 ms
Flip angle	35 deg
Fat suppr.	None
Wrap-up Magn.	None

Contrast - Dynamic

Averages	1
Averaging mode	Short term
Reconstruction	Magnitude
Measurements	1
Multiple series	Off

Resolution - Common

FoV read	360 mm
FoV phase	85.2 %
Slice thickness	8.0 mm
Base resolution	256
Phase resolution	66 %
Phase partial Fourier	7/8
Trajectory	Cartesian
Interpolation	Off

Resolution - iPAT

PAT mode	GRAPPA
Accel. factor PE	2
Ref. lines PE	36
Reference scan mode	GRE/separate

Resolution - Filter Image

Image Filter	Off
Distortion Corr.	On
Mode	2D
Unfiltered images	Off
Prescan Normalize	Off
Normalize	Off
B1 filter	Off

Resolution - Filter Rawdata

Raw filter	Off
Elliptical filter	Off
POCS	Off

Geometry - Common

Slice group	1
Slices	1
Dist. factor	20 %
Position	Isocenter
Orientation	Transversal
Phase enc. dir.	A >> P
FoV read	360 mm
FoV phase	85.2 %
Slice thickness	8.0 mm
TR	359.84 ms
Multi-slice mode	Sequential
Series	Base To Apex
Concatenations	1

Geometry - AutoAlign

Slice group	1
Position	Isocenter
Orientation	Transversal
Phase enc. dir.	A >> P
AutoAlign	---
Initial Position	Isocenter
Phase	0.0 mm
Read	0.0 mm
Shift	0.0 mm
Initial Rotation	0.00 deg
Initial Orientation	Transversal

Geometry - Saturation

Fat suppr.	None
Wrap-up Magn.	None
Special sat.	None

Geometry - Navigator

System - Miscellaneous

Positioning mode	FIX
Table position	H
Table position	0 mm

System - Miscellaneous

MSMA	S - C - T
Sagittal	R >> L
Coronal	A >> P
Transversal	F >> H
Coil Combine Mode	Adaptive Combine
Save uncombined	Off
Matrix Optimization	Off
Coil Focus	Flat
AutoAlign	---
Coil Select Mode	Default

System - Adjustments

B0 Shim mode	Cardiac
Adjust with body coil	Off
Confirm freq. adjustment	Off
Assume Dominant Fat	Off
Assume Silicone	Off
Adjustment Tolerance	Auto

System - Adjust Volume

! Position	Isocenter
! Orientation	Transversal
! Rotation	0.00 deg
! A >> P	150 mm
! R >> L	150 mm
! F >> H	150 mm
Reset	Off

System - Tx/Rx

Frequency 1H	63.683250 MHz
Correction factor	1
Gain	High
Img. Scale Cor.	1.000
Reset	Off
? Ref. amplitude 1H	0.000 V

Physio - Signal1

1st Signal/Mode	ECG/Trigger
Average cycle	787 ± 129 ms
Average cycle	No Signal ms
Captured cycle	787 ± 129 ms
Acquisition window	813 ms
Trigger pulse	1
Trigger delay	0 ms
TR	359.84 ms
Concatenations	1
Segments	72
Phases	1
Adaptive Triggering	Off

Physio - Cardiac

Tagging	None
Magn. preparation	Non-sel. IR T1map
TI	260 ms
Fat suppr.	None
Dark blood	Off
FoV read	360 mm
FoV phase	85.2 %
Phase resolution	66 %
Cine	Off
Trajectory	Cartesian
Dummy heartbeats	0
Motion Correction	Standard

Physio - PACE

Resp. control	Breath-hold
Concatenations	1

Sequence - Part 1

Introduction	Off
Dimension	2D
Reordering	Linear
Asymmetric echo	Weak
Contrasts	1
Optimization	Min. TE TR
Multi-slice mode	Sequential
Sequence type	Trufi
Bandwidth	1085 Hz/Px

Sequence - Part 2

Define	Shots
Shots per slice	1
Segments	72
Trufi delta freq.	0 Hz
RF pulse type	Fast
Gradient mode	Fast
Excitation	Slice-sel.
Flip angle mode	Constant
Cine	Off

Sequence - Assistant

Mode	Off
Allowed delay	0 s

\\USER\Cardiac Research Protocols\Rapi-STRESS T1\Rapi-STRESS T1 (V2)\ShMOLLI_192i_d11_nFilt (TD=0)

TA: 7.1 s PM: FIX Voxel size: 0.9×0.9×8.0 mmPAT: 2 Rel. SNR: 1.00 : tfi

Properties

Prio recon	Off
Load images to viewer	On
Inline movie	Off
Auto store images	On
Load images to stamp segments	Off
Load images to graphic segments	On
Auto open inline display	Off
Auto close inline display	Off
Start measurement without further preparation	On
Wait for user to start	Off
Start measurements	Single measurement

Routine

Slice group	1
Slices	1
Dist. factor	25 %
Position	Isocenter
Orientation	Transversal
Phase enc. dir.	A >> P
AutoAlign	---
Phase oversampling	0 %
FoV read	360 mm
FoV phase	75.0 %
Slice thickness	8.0 mm
TR	378.98 ms
TE	1.07 ms
Averages	1
Concatenations	1
Filter	Raw filter, Distortion Corr.(2D)
Coil elements	BO2;SP2,3

Contrast - Common

TR	378.98 ms
TE	1.07 ms
Magn. preparation	Non-sel. IR
TI	260 ms
Flip angle	35 deg
Fat suppr.	None
Wrap-up Magn.	None

Contrast - Dynamic

Averages	1
Averaging mode	Short term
Reconstruction	Magn./Phase
Measurements	1
Multiple series	Off

Resolution - Common

FoV read	360 mm
FoV phase	75.0 %
Slice thickness	8.0 mm
Base resolution	192
Phase resolution	100 %
Phase partial Fourier	6/8
Trajectory	Cartesian
Interpolation	On

Resolution - iPAT

PAT mode	GRAPPA
Accel. factor PE	2
Ref. lines PE	24
Reference scan mode	Integrated

Resolution - Filter Image

Image Filter	Off
Distortion Corr.	On
Mode	2D
Unfiltered images	Off
Prescan Normalize	Off
Normalize	Off
B1 filter	Off

Resolution - Filter Rawdata

Raw filter	On
Elliptical filter	Off
POCS	Off

Geometry - Common

Slice group	1
Slices	1
Dist. factor	25 %
Position	Isocenter
Orientation	Transversal
Phase enc. dir.	A >> P
FoV read	360 mm
FoV phase	75.0 %
Slice thickness	8.0 mm
TR	378.98 ms
Multi-slice mode	Sequential
Series	Interleaved
Concatenations	1

Geometry - AutoAlign

Slice group	1
Position	Isocenter
Orientation	Transversal
Phase enc. dir.	A >> P
AutoAlign	---
Initial Position	Isocenter
Phase	0.0 mm
Read	0.0 mm
Shift	0.0 mm
Initial Rotation	0.00 deg
Initial Orientation	Transversal

Geometry - Saturation

Fat suppr.	None
Wrap-up Magn.	None
Special sat.	None

Geometry - Navigator

System - Miscellaneous

Positioning mode	FIX
Table position	H
Table position	0 mm

System - Miscellaneous

MSMA	S - C - T
Sagittal	R >> L
Coronal	A >> P
Transversal	F >> H
Coil Combine Mode	Adaptive Combine
Save uncombined	Off
Matrix Optimization	Off
Coil Focus	Flat
AutoAlign	---
Coil Select Mode	Default

System - Adjustments

B0 Shim mode	Cardiac
Adjust with body coil	On
Confirm freq. adjustment	Off
Assume Dominant Fat	Off
Assume Silicone	Off
Adjustment Tolerance	Auto

System - Adjust Volume

! Position	Isocenter
! Orientation	Transversal
! Rotation	0.00 deg
! A >> P	150 mm
! R >> L	150 mm
! F >> H	150 mm
Reset	Off

System - Tx/Rx

Frequency 1H	63.683250 MHz
Correction factor	1
Gain	High
Img. Scale Cor.	1.000
Reset	Off
? Ref. amplitude 1H	0.000 V

Physio - Signal1

1st Signal/Mode	ECG/Trigger
Average cycle	787 ± 129 ms
Average cycle	No Signal ms
Captured cycle	787 ± 129 ms
Acquisition window	379 ms
Trigger pulse	1
Trigger delay	0 ms
TR	378.98 ms
Concatenations	1
Segments	84
Phases	1
Adaptive Triggering	Off

Physio - Cardiac

Tagging	None
Magn. preparation	Non-sel. IR
TI	260 ms
Fat suppr.	None
Dark blood	Off
FoV read	360 mm
FoV phase	75.0 %
Phase resolution	100 %
Cine	Off
Trajectory	Cartesian
Dummy heartbeats	0
Motion Correction	None

Physio - PACE

Resp. control	Off
Concatenations	1

Sequence - Part 1

Introduction	Off
Dimension	2D
Reordering	Linear
Asymmetric echo	Weak
Contrasts	1
Optimization	Min. TE TR
Multi-slice mode	Sequential
Sequence type	Trufi
Bandwidth	898 Hz/Px

Sequence - Part 2

Define	Shots
Shots per slice	1
Segments	84
Trufi delta freq.	0 Hz
RF pulse type	Fast
Gradient mode	Fast
Excitation	Slice-sel.
Flip angle mode	Constant
Cine	Off

Sequence - Assistant

Mode	Off
Allowed delay	0 s

\\USER\Cardiac Research Protocols\Rapi-STRESS T1\Rapi-STRESS T1 (V2)\T1Map_ShortT1(TD=0)highHR

TA: 8.7 s PM: FIX Voxel size: 1.9×1.9×8.0 mmPAT: 2 Rel. SNR: 1.00 : tfi

Properties

Prio recon	Off
Load images to viewer	On
Inline movie	Off
Auto store images	On
Load images to stamp segments	On
Load images to graphic segments	On
Auto open inline display	Off
Auto close inline display	Off
Start measurement without further preparation	On
Wait for user to start	Off
Start measurements	Single measurement

Routine

Slice group	1
Slices	1
Dist. factor	20 %
Position	Isocenter
Orientation	Transversal
Phase enc. dir.	A >> P
AutoAlign	---
Phase oversampling	0 %
FoV read	360 mm
FoV phase	85.4 %
Slice thickness	8.0 mm
TR	341.12 ms
TE	1.01 ms
Averages	1
Concatenations	1
Filter	Distortion Corr.(2D)
Coil elements	BO2;SP2,3

Contrast - Common

TR	341.12 ms
TE	1.01 ms
Magn. preparation	Non-sel. IR T1map
TI	260 ms
Flip angle	35 deg
Fat suppr.	None
Wrap-up Magn.	None

Contrast - Dynamic

Averages	1
Averaging mode	Short term
Reconstruction	Magnitude
Measurements	1
Multiple series	Off

Resolution - Common

FoV read	360 mm
FoV phase	85.4 %
Slice thickness	8.0 mm
Base resolution	192
Phase resolution	78 %
Phase partial Fourier	7/8
Trajectory	Cartesian
Interpolation	Off

Resolution - iPAT

PAT mode	GRAPPA
Accel. factor PE	2
Ref. lines PE	36
Reference scan mode	GRE/separate

Resolution - Filter Image

Image Filter	Off
Distortion Corr.	On
Mode	2D
Unfiltered images	Off
Prescan Normalize	Off
Normalize	Off
B1 filter	Off

Resolution - Filter Rawdata

Raw filter	Off
Elliptical filter	Off
POCS	Off

Geometry - Common

Slice group	1
Slices	1
Dist. factor	20 %
Position	Isocenter
Orientation	Transversal
Phase enc. dir.	A >> P
FoV read	360 mm
FoV phase	85.4 %
Slice thickness	8.0 mm
TR	341.12 ms
Multi-slice mode	Sequential
Series	Base To Apex
Concatenations	1

Geometry - AutoAlign

Slice group	1
Position	Isocenter
Orientation	Transversal
Phase enc. dir.	A >> P
AutoAlign	---
Initial Position	Isocenter
Phase	0.0 mm
Read	0.0 mm
Shift	0.0 mm
Initial Rotation	0.00 deg
Initial Orientation	Transversal

Geometry - Saturation

Fat suppr.	None
Wrap-up Magn.	None
Special sat.	None

Geometry - Navigator

System - Miscellaneous

Positioning mode	FIX
Table position	H
Table position	0 mm

System - Miscellaneous

MSMA	S - C - T
Sagittal	R >> L
Coronal	A >> P
Transversal	F >> H
Coil Combine Mode	Adaptive Combine
Save uncombined	Off
Matrix Optimization	Off
Coil Focus	Flat
AutoAlign	---
Coil Select Mode	Default

System - Adjustments

B0 Shim mode	Cardiac
Adjust with body coil	Off
Confirm freq. adjustment	Off
Assume Dominant Fat	Off
Assume Silicone	Off
Adjustment Tolerance	Auto

System - Adjust Volume

! Position	Isocenter
! Orientation	Transversal
! Rotation	0.00 deg
! A >> P	150 mm
! R >> L	150 mm
! F >> H	150 mm
Reset	Off

System - Tx/Rx

Frequency 1H	63.683250 MHz
Correction factor	1
Gain	High
Img. Scale Cor.	1.000
Reset	Off
? Ref. amplitude 1H	0.000 V

Physio - Signal1

1st Signal/Mode	ECG/Trigger
Average cycle	787 ± 129 ms
Average cycle	No Signal ms
Captured cycle	787 ± 129 ms
Acquisition window	704 ms
Trigger pulse	1
Trigger delay	0 ms
TR	341.12 ms
Concatenations	1
Segments	64
Phases	1
Adaptive Triggering	Off

Physio - Cardiac

Tagging	None
Magn. preparation	Non-sel. IR T1map
TI	260 ms
Fat suppr.	None
Dark blood	Off
FoV read	360 mm
FoV phase	85.4 %
Phase resolution	78 %
Cine	Off
Trajectory	Cartesian
Dummy heartbeats	0
Motion Correction	Standard

Physio - PACE

Resp. control	Breath-hold
Concatenations	1

Sequence - Part 1

Introduction	Off
Dimension	2D
Reordering	Linear
Asymmetric echo	Weak
Contrasts	1
Optimization	Min. TE TR
Multi-slice mode	Sequential
Sequence type	Trufi
Bandwidth	1085 Hz/Px

Sequence - Part 2

Define	Shots
Shots per slice	1
Segments	64
Trufi delta freq.	0 Hz
RF pulse type	Fast
Gradient mode	Fast
Excitation	Slice-sel.
Flip angle mode	Constant
Cine	Off

Sequence - Assistant

Mode	Off
Allowed delay	0 s

\\USER\Cardiac Research Protocols\Rapi-STRESS T1\Rapi-STRESS T1 (V2)\ShMOLLI_192i_d11_nFilt (TD=0)

TA: 7.1 s PM: FIX Voxel size: 0.9×0.9×8.0 mmPAT: 2 Rel. SNR: 1.00 : tfi

Properties

Prio recon	Off
Load images to viewer	On
Inline movie	Off
Auto store images	On
Load images to stamp segments	Off
Load images to graphic segments	On
Auto open inline display	Off
Auto close inline display	Off
Start measurement without further preparation	On
Wait for user to start	Off
Start measurements	Single measurement

Routine

Slice group	1
Slices	1
Dist. factor	25 %
Position	Isocenter
Orientation	Transversal
Phase enc. dir.	A >> P
AutoAlign	---
Phase oversampling	0 %
FoV read	360 mm
FoV phase	75.0 %
Slice thickness	8.0 mm
TR	378.98 ms
TE	1.07 ms
Averages	1
Concatenations	1
Filter	Raw filter, Distortion Corr.(2D)
Coil elements	BO2;SP2,3

Contrast - Common

TR	378.98 ms
TE	1.07 ms
Magn. preparation	Non-sel. IR
TI	260 ms
Flip angle	35 deg
Fat suppr.	None
Wrap-up Magn.	None

Contrast - Dynamic

Averages	1
Averaging mode	Short term
Reconstruction	Magn./Phase
Measurements	1
Multiple series	Off

Resolution - Common

FoV read	360 mm
FoV phase	75.0 %
Slice thickness	8.0 mm
Base resolution	192
Phase resolution	100 %
Phase partial Fourier	6/8
Trajectory	Cartesian
Interpolation	On

Resolution - iPAT

PAT mode	GRAPPA
Accel. factor PE	2
Ref. lines PE	24
Reference scan mode	Integrated

Resolution - Filter Image

Image Filter	Off
Distortion Corr.	On
Mode	2D
Unfiltered images	Off
Prescan Normalize	Off
Normalize	Off
B1 filter	Off

Resolution - Filter Rawdata

Raw filter	On
Elliptical filter	Off
POCS	Off

Geometry - Common

Slice group	1
Slices	1
Dist. factor	25 %
Position	Isocenter
Orientation	Transversal
Phase enc. dir.	A >> P
FoV read	360 mm
FoV phase	75.0 %
Slice thickness	8.0 mm
TR	378.98 ms
Multi-slice mode	Sequential
Series	Interleaved
Concatenations	1

Geometry - AutoAlign

Slice group	1
Position	Isocenter
Orientation	Transversal
Phase enc. dir.	A >> P
AutoAlign	---
Initial Position	Isocenter
Phase	0.0 mm
Read	0.0 mm
Shift	0.0 mm
Initial Rotation	0.00 deg
Initial Orientation	Transversal

Geometry - Saturation

Fat suppr.	None
Wrap-up Magn.	None
Special sat.	None

Geometry - Navigator

System - Miscellaneous

Positioning mode	FIX
Table position	H
Table position	0 mm

System - Miscellaneous

MSMA	S - C - T
Sagittal	R >> L
Coronal	A >> P
Transversal	F >> H
Coil Combine Mode	Adaptive Combine
Save uncombined	Off
Matrix Optimization	Off
Coil Focus	Flat
AutoAlign	---
Coil Select Mode	Default

System - Adjustments

B0 Shim mode	Cardiac
Adjust with body coil	On
Confirm freq. adjustment	Off
Assume Dominant Fat	Off
Assume Silicone	Off
Adjustment Tolerance	Auto

System - Adjust Volume

! Position	Isocenter
! Orientation	Transversal
! Rotation	0.00 deg
! A >> P	150 mm
! R >> L	150 mm
! F >> H	150 mm
Reset	Off

System - Tx/Rx

Frequency 1H	63.683250 MHz
Correction factor	1
Gain	High
Img. Scale Cor.	1.000
Reset	Off
? Ref. amplitude 1H	0.000 V

Physio - Signal1

1st Signal/Mode	ECG/Trigger
Average cycle	787 ± 129 ms
Average cycle	No Signal ms
Captured cycle	787 ± 129 ms
Acquisition window	379 ms
Trigger pulse	1
Trigger delay	0 ms
TR	378.98 ms
Concatenations	1
Segments	84
Phases	1
Adaptive Triggering	Off

Physio - Cardiac

Tagging	None
Magn. preparation	Non-sel. IR
TI	260 ms
Fat suppr.	None
Dark blood	Off
FoV read	360 mm
FoV phase	75.0 %
Phase resolution	100 %
Cine	Off
Trajectory	Cartesian
Dummy heartbeats	0
Motion Correction	None

Physio - PACE

Resp. control	Off
Concatenations	1

Sequence - Part 1

Introduction	Off
Dimension	2D
Reordering	Linear
Asymmetric echo	Weak
Contrasts	1
Optimization	Min. TE TR
Multi-slice mode	Sequential
Sequence type	Trufi
Bandwidth	898 Hz/Px

Sequence - Part 2

Define	Shots
Shots per slice	1
Segments	84
Trufi delta freq.	0 Hz
RF pulse type	Fast
Gradient mode	Fast
Excitation	Slice-sel.
Flip angle mode	Constant
Cine	Off

Sequence - Assistant

Mode	Off
Allowed delay	0 s

\\USER\Cardiac Research Protocols\Rapi-STRESS T1\Rapi-STRESS T1 (V2)\MidShMOLLI_192i_d11_nFilt (TD=0)
TA: 7.1 s PM: REF Voxel size: 0.9×0.9×8.0 mmPAT: 2 Rel. SNR: 1.00 : tfi

Properties

Prio recon	Off
Load images to viewer	On
Inline movie	Off
Auto store images	On
Load images to stamp segments	Off
Load images to graphic segments	On
Auto open inline display	Off
Auto close inline display	Off
Start measurement without further preparation	Off
Wait for user to start	Off
Start measurements	Single measurement

Routine

Slice group	1
Slices	1
Dist. factor	25 %
Position	Isocenter
Orientation	Transversal
Phase enc. dir.	A >> P
AutoAlign	---
Phase oversampling	0 %
FoV read	360 mm
FoV phase	75.0 %
Slice thickness	8.0 mm
TR	378.98 ms
TE	1.07 ms
Averages	1
Concatenations	1
Filter	Raw filter, Distortion Corr.(2D)
Coil elements	BO2;SP2,3

Contrast - Common

TR	378.98 ms
TE	1.07 ms
Magn. preparation	Non-sel. IR
TI	260 ms
Flip angle	35 deg
Fat suppr.	None
Wrap-up Magn.	None

Contrast - Dynamic

Averages	1
Averaging mode	Short term
Reconstruction	Magn./Phase
Measurements	1
Multiple series	Off

Resolution - Common

FoV read	360 mm
FoV phase	75.0 %
Slice thickness	8.0 mm
Base resolution	192
Phase resolution	100 %
Phase partial Fourier	6/8
Trajectory	Cartesian
Interpolation	On

Resolution - iPAT

PAT mode	GRAPPA
Accel. factor PE	2
Ref. lines PE	24
Reference scan mode	Integrated

Resolution - Filter Image

Image Filter	Off
Distortion Corr.	On
Mode	2D
Unfiltered images	Off
Prescan Normalize	Off
Normalize	Off
B1 filter	Off

Resolution - Filter Rawdata

Raw filter	On
Elliptical filter	Off
POCS	Off

Geometry - Common

Slice group	1
Slices	1
Dist. factor	25 %
Position	Isocenter
Orientation	Transversal
Phase enc. dir.	A >> P
FoV read	360 mm
FoV phase	75.0 %
Slice thickness	8.0 mm
TR	378.98 ms
Multi-slice mode	Sequential
Series	Interleaved
Concatenations	1

Geometry - AutoAlign

Slice group	1
Position	Isocenter
Orientation	Transversal
Phase enc. dir.	A >> P
AutoAlign	---
Initial Position	Isocenter
Phase	0.0 mm
Read	0.0 mm
Shift	0.0 mm
Initial Rotation	0.00 deg
Initial Orientation	Transversal

Geometry - Saturation

Fat suppr.	None
Wrap-up Magn.	None
Special sat.	None

Geometry - Navigator

System - Miscellaneous

Positioning mode	REF
Table position	H
Table position	0 mm

System - Miscellaneous

MSMA	S - C - T
Sagittal	R >> L
Coronal	A >> P
Transversal	F >> H
Coil Combine Mode	Adaptive Combine
Save uncombined	Off
Matrix Optimization	Off
Coil Focus	Flat
AutoAlign	---
Coil Select Mode	Default

System - Adjustments

B0 Shim mode	Cardiac
Adjust with body coil	On
Confirm freq. adjustment	Off
Assume Dominant Fat	Off
Assume Silicone	Off
Adjustment Tolerance	Auto

System - Adjust Volume

! Position	Isocenter
! Orientation	Transversal
! Rotation	0.00 deg
! A >> P	150 mm
! R >> L	150 mm
! F >> H	150 mm
Reset	Off

System - Tx/Rx

Frequency 1H	63.683250 MHz
Correction factor	1
Gain	High
Img. Scale Cor.	1.000
Reset	Off
? Ref. amplitude 1H	0.000 V

Physio - Signal1

1st Signal/Mode	ECG/Trigger
Average cycle	787 ± 129 ms
Average cycle	No Signal ms
Captured cycle	787 ± 129 ms
Acquisition window	379 ms
Trigger pulse	1
Trigger delay	0 ms
TR	378.98 ms
Concatenations	1
Segments	84
Phases	1
Adaptive Triggering	Off

Physio - Cardiac

Tagging	None
Magn. preparation	Non-sel. IR
TI	260 ms
Fat suppr.	None
Dark blood	Off
FoV read	360 mm
FoV phase	75.0 %
Phase resolution	100 %
Cine	Off
Trajectory	Cartesian
Dummy heartbeats	0
Motion Correction	None

Physio - PACE

Resp. control	Off
Concatenations	1

Sequence - Part 1

Introduction	Off
Dimension	2D
Reordering	Linear
Asymmetric echo	Weak
Contrasts	1
Optimization	Min. TE TR
Multi-slice mode	Sequential
Sequence type	Trufi
Bandwidth	898 Hz/Px

Sequence - Part 2

Define	Shots
Shots per slice	1
Segments	84
Trufi delta freq.	0 Hz
RF pulse type	Fast
Gradient mode	Fast
Excitation	Slice-sel.
Flip angle mode	Constant
Cine	Off

Sequence - Assistant

Mode	Off
Allowed delay	0 s

\\USER\Cardiac Research Protocols\Rapi-STRESS T1\Rapi-STRESS T1 (V2)\T1Map_ShortT1(TD=0)lowHR

TA: 0:17 PM: FIX Voxel size: 1.4×1.4×8.0 mmPAT: 2 Rel. SNR: 1.00 : tfi

Properties

Prio recon	Off
Load images to viewer	On
Inline movie	Off
Auto store images	On
Load images to stamp segments	On
Load images to graphic segments	On
Auto open inline display	Off
Auto close inline display	Off
Start measurement without further preparation	On
Wait for user to start	Off
Start measurements	Single measurement

Routine

Slice group	1
Slices	1
Dist. factor	20 %
Position	Isocenter
Orientation	Transversal
Phase enc. dir.	A >> P
AutoAlign	---
Phase oversampling	0 %
FoV read	360 mm
FoV phase	85.2 %
Slice thickness	8.0 mm
TR	359.84 ms
TE	1.13 ms
Averages	1
Concatenations	1
Filter	Distortion Corr.(2D)
Coil elements	BO2;SP2,3

Contrast - Common

TR	359.84 ms
TE	1.13 ms
Magn. preparation	Non-sel. IR T1map
TI	260 ms
Flip angle	35 deg
Fat suppr.	None
Wrap-up Magn.	None

Contrast - Dynamic

Averages	1
Averaging mode	Short term
Reconstruction	Magnitude
Measurements	1
Multiple series	Off

Resolution - Common

FoV read	360 mm
FoV phase	85.2 %
Slice thickness	8.0 mm
Base resolution	256
Phase resolution	66 %
Phase partial Fourier	7/8
Trajectory	Cartesian
Interpolation	Off

Resolution - iPAT

PAT mode	GRAPPA
Accel. factor PE	2
Ref. lines PE	36
Reference scan mode	GRE/separate

Resolution - Filter Image

Image Filter	Off
Distortion Corr.	On
Mode	2D
Unfiltered images	Off
Prescan Normalize	Off
Normalize	Off
B1 filter	Off

Resolution - Filter Rawdata

Raw filter	Off
Elliptical filter	Off
POCS	Off

Geometry - Common

Slice group	1
Slices	1
Dist. factor	20 %
Position	Isocenter
Orientation	Transversal
Phase enc. dir.	A >> P
FoV read	360 mm
FoV phase	85.2 %
Slice thickness	8.0 mm
TR	359.84 ms
Multi-slice mode	Sequential
Series	Base To Apex
Concatenations	1

Geometry - AutoAlign

Slice group	1
Position	Isocenter
Orientation	Transversal
Phase enc. dir.	A >> P
AutoAlign	---
Initial Position	Isocenter
Phase	0.0 mm
Read	0.0 mm
Shift	0.0 mm
Initial Rotation	0.00 deg
Initial Orientation	Transversal

Geometry - Saturation

Fat suppr.	None
Wrap-up Magn.	None
Special sat.	None

Geometry - Navigator

System - Miscellaneous

Positioning mode	FIX
Table position	H
Table position	0 mm

System - Miscellaneous

MSMA	S - C - T
Sagittal	R >> L
Coronal	A >> P
Transversal	F >> H
Coil Combine Mode	Adaptive Combine
Save uncombined	Off
Matrix Optimization	Off
Coil Focus	Flat
AutoAlign	---
Coil Select Mode	Default

System - Adjustments

B0 Shim mode	Cardiac
Adjust with body coil	Off
Confirm freq. adjustment	Off
Assume Dominant Fat	Off
Assume Silicone	Off
Adjustment Tolerance	Auto

System - Adjust Volume

! Position	Isocenter
! Orientation	Transversal
! Rotation	0.00 deg
! A >> P	150 mm
! R >> L	150 mm
! F >> H	150 mm
Reset	Off

System - Tx/Rx

Frequency 1H	63.683250 MHz
Correction factor	1
Gain	High
Img. Scale Cor.	1.000
Reset	Off
? Ref. amplitude 1H	0.000 V

Physio - Signal1

1st Signal/Mode	ECG/Trigger
Average cycle	787 ± 129 ms
Average cycle	No Signal ms
Captured cycle	787 ± 129 ms
Acquisition window	813 ms
Trigger pulse	1
Trigger delay	0 ms
TR	359.84 ms
Concatenations	1
Segments	72
Phases	1
Adaptive Triggering	Off

Physio - Cardiac

Tagging	None
Magn. preparation	Non-sel. IR T1map
TI	260 ms
Fat suppr.	None
Dark blood	Off
FoV read	360 mm
FoV phase	85.2 %
Phase resolution	66 %
Cine	Off
Trajectory	Cartesian
Dummy heartbeats	0
Motion Correction	Standard

Physio - PACE

Resp. control	Breath-hold
Concatenations	1

Sequence - Part 1

Introduction	Off
Dimension	2D
Reordering	Linear
Asymmetric echo	Weak
Contrasts	1
Optimization	Min. TE TR
Multi-slice mode	Sequential
Sequence type	Trufi
Bandwidth	1085 Hz/Px

Sequence - Part 2

Define	Shots
Shots per slice	1
Segments	72
Trufi delta freq.	0 Hz
RF pulse type	Fast
Gradient mode	Fast
Excitation	Slice-sel.
Flip angle mode	Constant
Cine	Off

Sequence - Assistant

Mode	Off
Allowed delay	0 s

\\USER\Cardiac Research Protocols\Rapi-STRESS T1\Rapi-STRESS T1 (V2)\ShMOLLI_192i_d11_nFilt (TD=0)

TA: 7.1 s PM: FIX Voxel size: 0.9×0.9×8.0 mmPAT: 2 Rel. SNR: 1.00 : tfi

Properties

Prio recon	Off
Load images to viewer	On
Inline movie	Off
Auto store images	On
Load images to stamp segments	Off
Load images to graphic segments	On
Auto open inline display	Off
Auto close inline display	Off
Start measurement without further preparation	On
Wait for user to start	Off
Start measurements	Single measurement

Routine

Slice group	1
Slices	1
Dist. factor	25 %
Position	Isocenter
Orientation	Transversal
Phase enc. dir.	A >> P
AutoAlign	---
Phase oversampling	0 %
FoV read	360 mm
FoV phase	75.0 %
Slice thickness	8.0 mm
TR	378.98 ms
TE	1.07 ms
Averages	1
Concatenations	1
Filter	Raw filter, Distortion Corr.(2D)
Coil elements	BO2;SP2,3

Contrast - Common

TR	378.98 ms
TE	1.07 ms
Magn. preparation	Non-sel. IR
TI	260 ms
Flip angle	35 deg
Fat suppr.	None
Wrap-up Magn.	None

Contrast - Dynamic

Averages	1
Averaging mode	Short term
Reconstruction	Magn./Phase
Measurements	1
Multiple series	Off

Resolution - Common

FoV read	360 mm
FoV phase	75.0 %
Slice thickness	8.0 mm
Base resolution	192
Phase resolution	100 %
Phase partial Fourier	6/8
Trajectory	Cartesian
Interpolation	On

Resolution - iPAT

PAT mode	GRAPPA
Accel. factor PE	2
Ref. lines PE	24
Reference scan mode	Integrated

Resolution - Filter Image

Image Filter	Off
Distortion Corr.	On
Mode	2D
Unfiltered images	Off
Prescan Normalize	Off
Normalize	Off
B1 filter	Off

Resolution - Filter Rawdata

Raw filter	On
Elliptical filter	Off
POCS	Off

Geometry - Common

Slice group	1
Slices	1
Dist. factor	25 %
Position	Isocenter
Orientation	Transversal
Phase enc. dir.	A >> P
FoV read	360 mm
FoV phase	75.0 %
Slice thickness	8.0 mm
TR	378.98 ms
Multi-slice mode	Sequential
Series	Interleaved
Concatenations	1

Geometry - AutoAlign

Slice group	1
Position	Isocenter
Orientation	Transversal
Phase enc. dir.	A >> P
AutoAlign	---
Initial Position	Isocenter
Phase	0.0 mm
Read	0.0 mm
Shift	0.0 mm
Initial Rotation	0.00 deg
Initial Orientation	Transversal

Geometry - Saturation

Fat suppr.	None
Wrap-up Magn.	None
Special sat.	None

Geometry - Navigator

System - Miscellaneous

Positioning mode	FIX
Table position	H
Table position	0 mm

System - Miscellaneous

MSMA	S - C - T
Sagittal	R >> L
Coronal	A >> P
Transversal	F >> H
Coil Combine Mode	Adaptive Combine
Save uncombined	Off
Matrix Optimization	Off
Coil Focus	Flat
AutoAlign	---
Coil Select Mode	Default

System - Adjustments

B0 Shim mode	Cardiac
Adjust with body coil	On
Confirm freq. adjustment	Off
Assume Dominant Fat	Off
Assume Silicone	Off
Adjustment Tolerance	Auto

System - Adjust Volume

! Position	Isocenter
! Orientation	Transversal
! Rotation	0.00 deg
! A >> P	150 mm
! R >> L	150 mm
! F >> H	150 mm
Reset	Off

System - Tx/Rx

Frequency 1H	63.683250 MHz
Correction factor	1
Gain	High
Img. Scale Cor.	1.000
Reset	Off
? Ref. amplitude 1H	0.000 V

Physio - Signal1

1st Signal/Mode	ECG/Trigger
Average cycle	787 ± 129 ms
Average cycle	No Signal ms
Captured cycle	787 ± 129 ms
Acquisition window	379 ms
Trigger pulse	1
Trigger delay	0 ms
TR	378.98 ms
Concatenations	1
Segments	84
Phases	1
Adaptive Triggering	Off

Physio - Cardiac

Tagging	None
Magn. preparation	Non-sel. IR
TI	260 ms
Fat suppr.	None
Dark blood	Off
FoV read	360 mm
FoV phase	75.0 %
Phase resolution	100 %
Cine	Off
Trajectory	Cartesian
Dummy heartbeats	0
Motion Correction	None

Physio - PACE

Resp. control	Off
Concatenations	1

Sequence - Part 1

Introduction	Off
Dimension	2D
Reordering	Linear
Asymmetric echo	Weak
Contrasts	1
Optimization	Min. TE TR
Multi-slice mode	Sequential
Sequence type	Trufi
Bandwidth	898 Hz/Px

Sequence - Part 2

Define	Shots
Shots per slice	1
Segments	84
Trufi delta freq.	0 Hz
RF pulse type	Fast
Gradient mode	Fast
Excitation	Slice-sel.
Flip angle mode	Constant
Cine	Off

Sequence - Assistant

Mode	Off
Allowed delay	0 s

\\USER\Cardiac Research Protocols\Rapi-STRESS T1\Rapi-STRESS T1 (V2)\T1Map_ShortT1(TD=0)highHR

TA: 8.7 s PM: FIX Voxel size: 1.9×1.9×8.0 mmPAT: 2 Rel. SNR: 1.00 : tfi

Properties

Prio recon	Off
Load images to viewer	On
Inline movie	Off
Auto store images	On
Load images to stamp segments	On
Load images to graphic segments	On
Auto open inline display	Off
Auto close inline display	Off
Start measurement without further preparation	On
Wait for user to start	Off
Start measurements	Single measurement

Routine

Slice group	1
Slices	1
Dist. factor	20 %
Position	Isocenter
Orientation	Transversal
Phase enc. dir.	A >> P
AutoAlign	---
Phase oversampling	0 %
FoV read	360 mm
FoV phase	85.4 %
Slice thickness	8.0 mm
TR	341.12 ms
TE	1.01 ms
Averages	1
Concatenations	1
Filter	Distortion Corr.(2D)
Coil elements	BO2;SP2,3

Contrast - Common

TR	341.12 ms
TE	1.01 ms
Magn. preparation	Non-sel. IR T1map
TI	260 ms
Flip angle	35 deg
Fat suppr.	None
Wrap-up Magn.	None

Contrast - Dynamic

Averages	1
Averaging mode	Short term
Reconstruction	Magnitude
Measurements	1
Multiple series	Off

Resolution - Common

FoV read	360 mm
FoV phase	85.4 %
Slice thickness	8.0 mm
Base resolution	192
Phase resolution	78 %
Phase partial Fourier	7/8
Trajectory	Cartesian
Interpolation	Off

Resolution - iPAT

PAT mode	GRAPPA
Accel. factor PE	2
Ref. lines PE	36
Reference scan mode	GRE/separate

Resolution - Filter Image

Image Filter	Off
Distortion Corr.	On
Mode	2D
Unfiltered images	Off
Prescan Normalize	Off
Normalize	Off
B1 filter	Off

Resolution - Filter Rawdata

Raw filter	Off
Elliptical filter	Off
POCS	Off

Geometry - Common

Slice group	1
Slices	1
Dist. factor	20 %
Position	Isocenter
Orientation	Transversal
Phase enc. dir.	A >> P
FoV read	360 mm
FoV phase	85.4 %
Slice thickness	8.0 mm
TR	341.12 ms
Multi-slice mode	Sequential
Series	Base To Apex
Concatenations	1

Geometry - AutoAlign

Slice group	1
Position	Isocenter
Orientation	Transversal
Phase enc. dir.	A >> P
AutoAlign	---
Initial Position	Isocenter
Phase	0.0 mm
Read	0.0 mm
Shift	0.0 mm
Initial Rotation	0.00 deg
Initial Orientation	Transversal

Geometry - Saturation

Fat suppr.	None
Wrap-up Magn.	None
Special sat.	None

Geometry - Navigator

System - Miscellaneous

Positioning mode	FIX
Table position	H
Table position	0 mm

System - Miscellaneous

MSMA	S - C - T
Sagittal	R >> L
Coronal	A >> P
Transversal	F >> H
Coil Combine Mode	Adaptive Combine
Save uncombined	Off
Matrix Optimization	Off
Coil Focus	Flat
AutoAlign	---
Coil Select Mode	Default

System - Adjustments

B0 Shim mode	Cardiac
Adjust with body coil	Off
Confirm freq. adjustment	Off
Assume Dominant Fat	Off
Assume Silicone	Off
Adjustment Tolerance	Auto

System - Adjust Volume

! Position	Isocenter
! Orientation	Transversal
! Rotation	0.00 deg
! A >> P	150 mm
! R >> L	150 mm
! F >> H	150 mm
Reset	Off

System - Tx/Rx

Frequency 1H	63.683250 MHz
Correction factor	1
Gain	High
Img. Scale Cor.	1.000
Reset	Off
? Ref. amplitude 1H	0.000 V

Physio - Signal1

1st Signal/Mode	ECG/Trigger
Average cycle	787 ± 129 ms
Average cycle	No Signal ms
Captured cycle	787 ± 129 ms
Acquisition window	704 ms
Trigger pulse	1
Trigger delay	0 ms
TR	341.12 ms
Concatenations	1
Segments	64
Phases	1
Adaptive Triggering	Off

Physio - Cardiac

Tagging	None
Magn. preparation	Non-sel. IR T1map
TI	260 ms
Fat suppr.	None
Dark blood	Off
FoV read	360 mm
FoV phase	85.4 %
Phase resolution	78 %
Cine	Off
Trajectory	Cartesian
Dummy heartbeats	0
Motion Correction	Standard

Physio - PACE

Resp. control	Breath-hold
Concatenations	1

Sequence - Part 1

Introduction	Off
Dimension	2D
Reordering	Linear
Asymmetric echo	Weak
Contrasts	1
Optimization	Min. TE TR
Multi-slice mode	Sequential
Sequence type	Trufi
Bandwidth	1085 Hz/Px

Sequence - Part 2

Define	Shots
Shots per slice	1
Segments	64
Trufi delta freq.	0 Hz
RF pulse type	Fast
Gradient mode	Fast
Excitation	Slice-sel.
Flip angle mode	Constant
Cine	Off

Sequence - Assistant

Mode	Off
Allowed delay	0 s

\\USER\Cardiac Research Protocols\Rapi-STRESS T1\Rapi-STRESS T1 (V2)\ShMOLLI_192i_d11_nFilt (TD=0)

TA: 7.1 s PM: FIX Voxel size: 0.9×0.9×8.0 mmPAT: 2 Rel. SNR: 1.00 : tfi

Properties

Prio recon	Off
Load images to viewer	On
Inline movie	Off
Auto store images	On
Load images to stamp segments	Off
Load images to graphic segments	On
Auto open inline display	Off
Auto close inline display	Off
Start measurement without further preparation	On
Wait for user to start	Off
Start measurements	Single measurement

Routine

Slice group	1
Slices	1
Dist. factor	25 %
Position	Isocenter
Orientation	Transversal
Phase enc. dir.	A >> P
AutoAlign	---
Phase oversampling	0 %
FoV read	360 mm
FoV phase	75.0 %
Slice thickness	8.0 mm
TR	378.98 ms
TE	1.07 ms
Averages	1
Concatenations	1
Filter	Raw filter, Distortion Corr.(2D)
Coil elements	BO2;SP2,3

Contrast - Common

TR	378.98 ms
TE	1.07 ms
Magn. preparation	Non-sel. IR
TI	260 ms
Flip angle	35 deg
Fat suppr.	None
Wrap-up Magn.	None

Contrast - Dynamic

Averages	1
Averaging mode	Short term
Reconstruction	Magn./Phase
Measurements	1
Multiple series	Off

Resolution - Common

FoV read	360 mm
FoV phase	75.0 %
Slice thickness	8.0 mm
Base resolution	192
Phase resolution	100 %
Phase partial Fourier	6/8
Trajectory	Cartesian
Interpolation	On

Resolution - iPAT

PAT mode	GRAPPA
Accel. factor PE	2
Ref. lines PE	24
Reference scan mode	Integrated

Resolution - Filter Image

Image Filter	Off
Distortion Corr.	On
Mode	2D
Unfiltered images	Off
Prescan Normalize	Off
Normalize	Off
B1 filter	Off

Resolution - Filter Rawdata

Raw filter	On
Elliptical filter	Off
POCS	Off

Geometry - Common

Slice group	1
Slices	1
Dist. factor	25 %
Position	Isocenter
Orientation	Transversal
Phase enc. dir.	A >> P
FoV read	360 mm
FoV phase	75.0 %
Slice thickness	8.0 mm
TR	378.98 ms
Multi-slice mode	Sequential
Series	Interleaved
Concatenations	1

Geometry - AutoAlign

Slice group	1
Position	Isocenter
Orientation	Transversal
Phase enc. dir.	A >> P
AutoAlign	---
Initial Position	Isocenter
Phase	0.0 mm
Read	0.0 mm
Shift	0.0 mm
Initial Rotation	0.00 deg
Initial Orientation	Transversal

Geometry - Saturation

Fat suppr.	None
Wrap-up Magn.	None
Special sat.	None

Geometry - Navigator

System - Miscellaneous

Positioning mode	FIX
Table position	H
Table position	0 mm

System - Miscellaneous

MSMA	S - C - T
Sagittal	R >> L
Coronal	A >> P
Transversal	F >> H
Coil Combine Mode	Adaptive Combine
Save uncombined	Off
Matrix Optimization	Off
Coil Focus	Flat
AutoAlign	---
Coil Select Mode	Default

System - Adjustments

B0 Shim mode	Cardiac
Adjust with body coil	On
Confirm freq. adjustment	Off
Assume Dominant Fat	Off
Assume Silicone	Off
Adjustment Tolerance	Auto

System - Adjust Volume

! Position	Isocenter
! Orientation	Transversal
! Rotation	0.00 deg
! A >> P	150 mm
! R >> L	150 mm
! F >> H	150 mm
Reset	Off

System - Tx/Rx

Frequency 1H	63.683250 MHz
Correction factor	1
Gain	High
Img. Scale Cor.	1.000
Reset	Off
? Ref. amplitude 1H	0.000 V

Physio - Signal1

1st Signal/Mode	ECG/Trigger
Average cycle	787 ± 129 ms
Average cycle	No Signal ms
Captured cycle	787 ± 129 ms
Acquisition window	379 ms
Trigger pulse	1
Trigger delay	0 ms
TR	378.98 ms
Concatenations	1
Segments	84
Phases	1
Adaptive Triggering	Off

Physio - Cardiac

Tagging	None
Magn. preparation	Non-sel. IR
TI	260 ms
Fat suppr.	None
Dark blood	Off
FoV read	360 mm
FoV phase	75.0 %
Phase resolution	100 %
Cine	Off
Trajectory	Cartesian
Dummy heartbeats	0
Motion Correction	None

Physio - PACE

Resp. control	Off
Concatenations	1

Sequence - Part 1

Introduction	Off
Dimension	2D
Reordering	Linear
Asymmetric echo	Weak
Contrasts	1
Optimization	Min. TE TR
Multi-slice mode	Sequential
Sequence type	Trufi
Bandwidth	898 Hz/Px

Sequence - Part 2

Define	Shots
Shots per slice	1
Segments	84
Trufi delta freq.	0 Hz
RF pulse type	Fast
Gradient mode	Fast
Excitation	Slice-sel.
Flip angle mode	Constant
Cine	Off

Sequence - Assistant

Mode	Off
Allowed delay	0 s

\\USER\Cardiac Research Protocols\Rapi-STRESS T1\Rapi-STRESS T1 (V2)\MidShMOLLI_192i_d11_nFilt (TD=0)

TA: 7.1 s PM: REF Voxel size: 0.9×0.9×8.0 mmPAT: 2 Rel. SNR: 1.00 : tfi

Properties

Prio recon	Off
Load images to viewer	On
Inline movie	Off
Auto store images	On
Load images to stamp segments	Off
Load images to graphic segments	On
Auto open inline display	Off
Auto close inline display	Off
Start measurement without further preparation	Off
Wait for user to start	Off
Start measurements	Single measurement

Routine

Slice group	1
Slices	1
Dist. factor	25 %
Position	Isocenter
Orientation	Transversal
Phase enc. dir.	A >> P
AutoAlign	---
Phase oversampling	0 %
FoV read	360 mm
FoV phase	75.0 %
Slice thickness	8.0 mm
TR	378.98 ms
TE	1.07 ms
Averages	1
Concatenations	1
Filter	Raw filter, Distortion Corr.(2D)
Coil elements	BO2;SP2,3

Contrast - Common

TR	378.98 ms
TE	1.07 ms
Magn. preparation	Non-sel. IR
TI	260 ms
Flip angle	35 deg
Fat suppr.	None
Wrap-up Magn.	None

Contrast - Dynamic

Averages	1
Averaging mode	Short term
Reconstruction	Magn./Phase
Measurements	1
Multiple series	Off

Resolution - Common

FoV read	360 mm
FoV phase	75.0 %
Slice thickness	8.0 mm
Base resolution	192
Phase resolution	100 %
Phase partial Fourier	6/8
Trajectory	Cartesian
Interpolation	On

Resolution - iPAT

PAT mode	GRAPPA
Accel. factor PE	2
Ref. lines PE	24
Reference scan mode	Integrated

Resolution - Filter Image

Image Filter	Off
Distortion Corr.	On
Mode	2D
Unfiltered images	Off
Prescan Normalize	Off
Normalize	Off
B1 filter	Off

Resolution - Filter Rawdata

Raw filter	On
Elliptical filter	Off
POCS	Off

Geometry - Common

Slice group	1
Slices	1
Dist. factor	25 %
Position	Isocenter
Orientation	Transversal
Phase enc. dir.	A >> P
FoV read	360 mm
FoV phase	75.0 %
Slice thickness	8.0 mm
TR	378.98 ms
Multi-slice mode	Sequential
Series	Interleaved
Concatenations	1

Geometry - AutoAlign

Slice group	1
Position	Isocenter
Orientation	Transversal
Phase enc. dir.	A >> P
AutoAlign	---
Initial Position	Isocenter
Phase	0.0 mm
Read	0.0 mm
Shift	0.0 mm
Initial Rotation	0.00 deg
Initial Orientation	Transversal

Geometry - Saturation

Fat suppr.	None
Wrap-up Magn.	None
Special sat.	None

Geometry - Navigator

System - Miscellaneous

Positioning mode	REF
Table position	H
Table position	0 mm

System - Miscellaneous

MSMA	S - C - T
Sagittal	R >> L
Coronal	A >> P
Transversal	F >> H
Coil Combine Mode	Adaptive Combine
Save uncombined	Off
Matrix Optimization	Off
Coil Focus	Flat
AutoAlign	---
Coil Select Mode	Default

System - Adjustments

B0 Shim mode	Cardiac
Adjust with body coil	On
Confirm freq. adjustment	Off
Assume Dominant Fat	Off
Assume Silicone	Off
Adjustment Tolerance	Auto

System - Adjust Volume

! Position	Isocenter
! Orientation	Transversal
! Rotation	0.00 deg
! A >> P	150 mm
! R >> L	150 mm
! F >> H	150 mm
Reset	Off

System - Tx/Rx

Frequency 1H	63.683250 MHz
Correction factor	1
Gain	High
Img. Scale Cor.	1.000
Reset	Off
? Ref. amplitude 1H	0.000 V

Physio - Signal1

1st Signal/Mode	ECG/Trigger
Average cycle	787 ± 129 ms
Average cycle	No Signal ms
Captured cycle	787 ± 129 ms
Acquisition window	379 ms
Trigger pulse	1
Trigger delay	0 ms
TR	378.98 ms
Concatenations	1
Segments	84
Phases	1
Adaptive Triggering	Off

Physio - Cardiac

Tagging	None
Magn. preparation	Non-sel. IR
TI	260 ms
Fat suppr.	None
Dark blood	Off
FoV read	360 mm
FoV phase	75.0 %
Phase resolution	100 %
Cine	Off
Trajectory	Cartesian
Dummy heartbeats	0
Motion Correction	None

Physio - PACE

Resp. control	Off
Concatenations	1

Sequence - Part 1

Introduction	Off
Dimension	2D
Reordering	Linear
Asymmetric echo	Weak
Contrasts	1
Optimization	Min. TE TR
Multi-slice mode	Sequential
Sequence type	Trufi
Bandwidth	898 Hz/Px

Sequence - Part 2

Define	Shots
Shots per slice	1
Segments	84
Trufi delta freq.	0 Hz
RF pulse type	Fast
Gradient mode	Fast
Excitation	Slice-sel.
Flip angle mode	Constant
Cine	Off

Sequence - Assistant

Mode	Off
Allowed delay	0 s

\\USER\Cardiac Research Protocols\Rapi-STRESS T1\Rapi-STRESS T1 (V2)\T1Map_ShortT1(TD=0)lowHR

TA: 0:17 PM: FIX Voxel size: 1.4×1.4×8.0 mmPAT: 2 Rel. SNR: 1.00 : tfi

Properties

Prio recon	Off
Load images to viewer	On
Inline movie	Off
Auto store images	On
Load images to stamp segments	On
Load images to graphic segments	On
Auto open inline display	Off
Auto close inline display	Off
Start measurement without further preparation	On
Wait for user to start	Off
Start measurements	Single measurement

Routine

Slice group	1
Slices	1
Dist. factor	20 %
Position	Isocenter
Orientation	Transversal
Phase enc. dir.	A >> P
AutoAlign	---
Phase oversampling	0 %
FoV read	360 mm
FoV phase	85.2 %
Slice thickness	8.0 mm
TR	359.84 ms
TE	1.13 ms
Averages	1
Concatenations	1
Filter	Distortion Corr.(2D)
Coil elements	BO2;SP2,3

Contrast - Common

TR	359.84 ms
TE	1.13 ms
Magn. preparation	Non-sel. IR T1map
TI	260 ms
Flip angle	35 deg
Fat suppr.	None
Wrap-up Magn.	None

Contrast - Dynamic

Averages	1
Averaging mode	Short term
Reconstruction	Magnitude
Measurements	1
Multiple series	Off

Resolution - Common

FoV read	360 mm
FoV phase	85.2 %
Slice thickness	8.0 mm
Base resolution	256
Phase resolution	66 %
Phase partial Fourier	7/8
Trajectory	Cartesian
Interpolation	Off

Resolution - iPAT

PAT mode	GRAPPA
Accel. factor PE	2
Ref. lines PE	36
Reference scan mode	GRE/separate

Resolution - Filter Image

Image Filter	Off
Distortion Corr.	On
Mode	2D
Unfiltered images	Off
Prescan Normalize	Off
Normalize	Off
B1 filter	Off

Resolution - Filter Rawdata

Raw filter	Off
Elliptical filter	Off
POCS	Off

Geometry - Common

Slice group	1
Slices	1
Dist. factor	20 %
Position	Isocenter
Orientation	Transversal
Phase enc. dir.	A >> P
FoV read	360 mm
FoV phase	85.2 %
Slice thickness	8.0 mm
TR	359.84 ms
Multi-slice mode	Sequential
Series	Base To Apex
Concatenations	1

Geometry - AutoAlign

Slice group	1
Position	Isocenter
Orientation	Transversal
Phase enc. dir.	A >> P
AutoAlign	---
Initial Position	Isocenter
Phase	0.0 mm
Read	0.0 mm
Shift	0.0 mm
Initial Rotation	0.00 deg
Initial Orientation	Transversal

Geometry - Saturation

Fat suppr.	None
Wrap-up Magn.	None
Special sat.	None

Geometry - Navigator

System - Miscellaneous

Positioning mode	FIX
Table position	H
Table position	0 mm

System - Miscellaneous

MSMA	S - C - T
Sagittal	R >> L
Coronal	A >> P
Transversal	F >> H
Coil Combine Mode	Adaptive Combine
Save uncombined	Off
Matrix Optimization	Off
Coil Focus	Flat
AutoAlign	---
Coil Select Mode	Default

System - Adjustments

B0 Shim mode	Cardiac
Adjust with body coil	Off
Confirm freq. adjustment	Off
Assume Dominant Fat	Off
Assume Silicone	Off
Adjustment Tolerance	Auto

System - Adjust Volume

! Position	Isocenter
! Orientation	Transversal
! Rotation	0.00 deg
! A >> P	150 mm
! R >> L	150 mm
! F >> H	150 mm
Reset	Off

System - Tx/Rx

Frequency 1H	63.683250 MHz
Correction factor	1
Gain	High
Img. Scale Cor.	1.000
Reset	Off
? Ref. amplitude 1H	0.000 V

Physio - Signal1

1st Signal/Mode	ECG/Trigger
Average cycle	787 ± 129 ms
Average cycle	No Signal ms
Captured cycle	787 ± 129 ms
Acquisition window	813 ms
Trigger pulse	1
Trigger delay	0 ms
TR	359.84 ms
Concatenations	1
Segments	72
Phases	1
Adaptive Triggering	Off

Physio - Cardiac

Tagging	None
Magn. preparation	Non-sel. IR T1map
TI	260 ms
Fat suppr.	None
Dark blood	Off
FoV read	360 mm
FoV phase	85.2 %
Phase resolution	66 %
Cine	Off
Trajectory	Cartesian
Dummy heartbeats	0
Motion Correction	Standard

Physio - PACE

Resp. control	Breath-hold
Concatenations	1

Sequence - Part 1

Introduction	Off
Dimension	2D
Reordering	Linear
Asymmetric echo	Weak
Contrasts	1
Optimization	Min. TE TR
Multi-slice mode	Sequential
Sequence type	Trufi
Bandwidth	1085 Hz/Px

Sequence - Part 2

Define	Shots
Shots per slice	1
Segments	72
Trufi delta freq.	0 Hz
RF pulse type	Fast
Gradient mode	Fast
Excitation	Slice-sel.
Flip angle mode	Constant
Cine	Off

Sequence - Assistant

Mode	Off
Allowed delay	0 s

\\USER\Cardiac Research Protocols\Rapi-STRESS T1\Rapi-STRESS T1 (V2)\ShMOLLI_192i_d11_nFilt (TD=0)

TA: 7.1 s PM: FIX Voxel size: 0.9×0.9×8.0 mmPAT: 2 Rel. SNR: 1.00 : tfi

Properties

Prio recon	Off
Load images to viewer	On
Inline movie	Off
Auto store images	On
Load images to stamp segments	Off
Load images to graphic segments	On
Auto open inline display	Off
Auto close inline display	Off
Start measurement without further preparation	On
Wait for user to start	Off
Start measurements	Single measurement

Routine

Slice group	1
Slices	1
Dist. factor	25 %
Position	Isocenter
Orientation	Transversal
Phase enc. dir.	A >> P
AutoAlign	---
Phase oversampling	0 %
FoV read	360 mm
FoV phase	75.0 %
Slice thickness	8.0 mm
TR	378.98 ms
TE	1.07 ms
Averages	1
Concatenations	1
Filter	Raw filter, Distortion Corr.(2D)
Coil elements	BO2;SP2,3

Contrast - Common

TR	378.98 ms
TE	1.07 ms
Magn. preparation	Non-sel. IR
TI	260 ms
Flip angle	35 deg
Fat suppr.	None
Wrap-up Magn.	None

Contrast - Dynamic

Averages	1
Averaging mode	Short term
Reconstruction	Magn./Phase
Measurements	1
Multiple series	Off

Resolution - Common

FoV read	360 mm
FoV phase	75.0 %
Slice thickness	8.0 mm
Base resolution	192
Phase resolution	100 %
Phase partial Fourier	6/8
Trajectory	Cartesian
Interpolation	On

Resolution - iPAT

PAT mode	GRAPPA
Accel. factor PE	2
Ref. lines PE	24
Reference scan mode	Integrated

Resolution - Filter Image

Image Filter	Off
Distortion Corr.	On
Mode	2D
Unfiltered images	Off
Prescan Normalize	Off
Normalize	Off
B1 filter	Off

Resolution - Filter Rawdata

Raw filter	On
Elliptical filter	Off
POCS	Off

Geometry - Common

Slice group	1
Slices	1
Dist. factor	25 %
Position	Isocenter
Orientation	Transversal
Phase enc. dir.	A >> P
FoV read	360 mm
FoV phase	75.0 %
Slice thickness	8.0 mm
TR	378.98 ms
Multi-slice mode	Sequential
Series	Interleaved
Concatenations	1

Geometry - AutoAlign

Slice group	1
Position	Isocenter
Orientation	Transversal
Phase enc. dir.	A >> P
AutoAlign	---
Initial Position	Isocenter
Phase	0.0 mm
Read	0.0 mm
Shift	0.0 mm
Initial Rotation	0.00 deg
Initial Orientation	Transversal

Geometry - Saturation

Fat suppr.	None
Wrap-up Magn.	None
Special sat.	None

Geometry - Navigator

System - Miscellaneous

Positioning mode	FIX
Table position	H
Table position	0 mm

System - Miscellaneous

MSMA	S - C - T
Sagittal	R >> L
Coronal	A >> P
Transversal	F >> H
Coil Combine Mode	Adaptive Combine
Save uncombined	Off
Matrix Optimization	Off
Coil Focus	Flat
AutoAlign	---
Coil Select Mode	Default

System - Adjustments

B0 Shim mode	Cardiac
Adjust with body coil	On
Confirm freq. adjustment	Off
Assume Dominant Fat	Off
Assume Silicone	Off
Adjustment Tolerance	Auto

System - Adjust Volume

! Position	Isocenter
! Orientation	Transversal
! Rotation	0.00 deg
! A >> P	150 mm
! R >> L	150 mm
! F >> H	150 mm
Reset	Off

System - Tx/Rx

Frequency 1H	63.683250 MHz
Correction factor	1
Gain	High
Img. Scale Cor.	1.000
Reset	Off
? Ref. amplitude 1H	0.000 V

Physio - Signal1

1st Signal/Mode	ECG/Trigger
Average cycle	787 ± 129 ms
Average cycle	No Signal ms
Captured cycle	787 ± 129 ms
Acquisition window	379 ms
Trigger pulse	1
Trigger delay	0 ms
TR	378.98 ms
Concatenations	1
Segments	84
Phases	1
Adaptive Triggering	Off

Physio - Cardiac

Tagging	None
Magn. preparation	Non-sel. IR
TI	260 ms
Fat suppr.	None
Dark blood	Off
FoV read	360 mm
FoV phase	75.0 %
Phase resolution	100 %
Cine	Off
Trajectory	Cartesian
Dummy heartbeats	0
Motion Correction	None

Physio - PACE

Resp. control	Off
Concatenations	1

Sequence - Part 1

Introduction	Off
Dimension	2D
Reordering	Linear
Asymmetric echo	Weak
Contrasts	1
Optimization	Min. TE TR
Multi-slice mode	Sequential
Sequence type	Trufi
Bandwidth	898 Hz/Px

Sequence - Part 2

Define	Shots
Shots per slice	1
Segments	84
Trufi delta freq.	0 Hz
RF pulse type	Fast
Gradient mode	Fast
Excitation	Slice-sel.
Flip angle mode	Constant
Cine	Off

Sequence - Assistant

Mode	Off
Allowed delay	0 s

\\USER\Cardiac Research Protocols\Rapi-STRESS T1\Rapi-STRESS T1 (V2)\T1Map_ShortT1(TD=0)highHR

TA: 8.7 s PM: FIX Voxel size: 1.9×1.9×8.0 mmPAT: 2 Rel. SNR: 1.00 : tfi

Properties

Prio recon	Off
Load images to viewer	On
Inline movie	Off
Auto store images	On
Load images to stamp segments	On
Load images to graphic segments	On
Auto open inline display	Off
Auto close inline display	Off
Start measurement without further preparation	On
Wait for user to start	Off
Start measurements	Single measurement

Routine

Slice group	1
Slices	1
Dist. factor	20 %
Position	Isocenter
Orientation	Transversal
Phase enc. dir.	A >> P
AutoAlign	---
Phase oversampling	0 %
FoV read	360 mm
FoV phase	85.4 %
Slice thickness	8.0 mm
TR	341.12 ms
TE	1.01 ms
Averages	1
Concatenations	1
Filter	Distortion Corr.(2D)
Coil elements	BO2;SP2,3

Contrast - Common

TR	341.12 ms
TE	1.01 ms
Magn. preparation	Non-sel. IR T1map
TI	260 ms
Flip angle	35 deg
Fat suppr.	None
Wrap-up Magn.	None

Contrast - Dynamic

Averages	1
Averaging mode	Short term
Reconstruction	Magnitude
Measurements	1
Multiple series	Off

Resolution - Common

FoV read	360 mm
FoV phase	85.4 %
Slice thickness	8.0 mm
Base resolution	192
Phase resolution	78 %
Phase partial Fourier	7/8
Trajectory	Cartesian
Interpolation	Off

Resolution - iPAT

PAT mode	GRAPPA
Accel. factor PE	2
Ref. lines PE	36
Reference scan mode	GRE/separate

Resolution - Filter Image

Image Filter	Off
Distortion Corr.	On
Mode	2D
Unfiltered images	Off
Prescan Normalize	Off
Normalize	Off
B1 filter	Off

Resolution - Filter Rawdata

Raw filter	Off
Elliptical filter	Off
POCS	Off

Geometry - Common

Slice group	1
Slices	1
Dist. factor	20 %
Position	Isocenter
Orientation	Transversal
Phase enc. dir.	A >> P
FoV read	360 mm
FoV phase	85.4 %
Slice thickness	8.0 mm
TR	341.12 ms
Multi-slice mode	Sequential
Series	Base To Apex
Concatenations	1

Geometry - AutoAlign

Slice group	1
Position	Isocenter
Orientation	Transversal
Phase enc. dir.	A >> P
AutoAlign	---
Initial Position	Isocenter
Phase	0.0 mm
Read	0.0 mm
Shift	0.0 mm
Initial Rotation	0.00 deg
Initial Orientation	Transversal

Geometry - Saturation

Fat suppr.	None
Wrap-up Magn.	None
Special sat.	None

Geometry - Navigator

System - Miscellaneous

Positioning mode	FIX
Table position	H
Table position	0 mm

System - Miscellaneous

MSMA	S - C - T
Sagittal	R >> L
Coronal	A >> P
Transversal	F >> H
Coil Combine Mode	Adaptive Combine
Save uncombined	Off
Matrix Optimization	Off
Coil Focus	Flat
AutoAlign	---
Coil Select Mode	Default

System - Adjustments

B0 Shim mode	Cardiac
Adjust with body coil	Off
Confirm freq. adjustment	Off
Assume Dominant Fat	Off
Assume Silicone	Off
Adjustment Tolerance	Auto

System - Adjust Volume

! Position	Isocenter
! Orientation	Transversal
! Rotation	0.00 deg
! A >> P	150 mm
! R >> L	150 mm
! F >> H	150 mm
Reset	Off

System - Tx/Rx

Frequency 1H	63.683250 MHz
Correction factor	1
Gain	High
Img. Scale Cor.	1.000
Reset	Off
? Ref. amplitude 1H	0.000 V

Physio - Signal1

1st Signal/Mode	ECG/Trigger
Average cycle	787 ± 129 ms
Average cycle	No Signal ms
Captured cycle	787 ± 129 ms
Acquisition window	704 ms
Trigger pulse	1
Trigger delay	0 ms
TR	341.12 ms
Concatenations	1
Segments	64
Phases	1
Adaptive Triggering	Off

Physio - Cardiac

Tagging	None
Magn. preparation	Non-sel. IR T1map
TI	260 ms
Fat suppr.	None
Dark blood	Off
FoV read	360 mm
FoV phase	85.4 %
Phase resolution	78 %
Cine	Off
Trajectory	Cartesian
Dummy heartbeats	0
Motion Correction	Standard

Physio - PACE

Resp. control	Breath-hold
Concatenations	1

Sequence - Part 1

Introduction	Off
Dimension	2D
Reordering	Linear
Asymmetric echo	Weak
Contrasts	1
Optimization	Min. TE TR
Multi-slice mode	Sequential
Sequence type	Trufi
Bandwidth	1085 Hz/Px

Sequence - Part 2

Define	Shots
Shots per slice	1
Segments	64
Trufi delta freq.	0 Hz
RF pulse type	Fast
Gradient mode	Fast
Excitation	Slice-sel.
Flip angle mode	Constant
Cine	Off

Sequence - Assistant

Mode	Off
Allowed delay	0 s

\\USER\Cardiac Research Protocols\Rapi-STRESS T1\Rapi-STRESS T1 (V2)\ShMOLLI_192i_d11_nFilt (TD=0)

TA: 7.1 s PM: FIX Voxel size: 0.9×0.9×8.0 mmPAT: 2 Rel. SNR: 1.00 : tfi

Properties

Prio recon	Off
Load images to viewer	On
Inline movie	Off
Auto store images	On
Load images to stamp segments	Off
Load images to graphic segments	On
Auto open inline display	Off
Auto close inline display	Off
Start measurement without further preparation	On
Wait for user to start	Off
Start measurements	Single measurement

Routine

Slice group	1
Slices	1
Dist. factor	25 %
Position	Isocenter
Orientation	Transversal
Phase enc. dir.	A >> P
AutoAlign	---
Phase oversampling	0 %
FoV read	360 mm
FoV phase	75.0 %
Slice thickness	8.0 mm
TR	378.98 ms
TE	1.07 ms
Averages	1
Concatenations	1
Filter	Raw filter, Distortion Corr.(2D)
Coil elements	BO2;SP2,3

Contrast - Common

TR	378.98 ms
TE	1.07 ms
Magn. preparation	Non-sel. IR
TI	260 ms
Flip angle	35 deg
Fat suppr.	None
Wrap-up Magn.	None

Contrast - Dynamic

Averages	1
Averaging mode	Short term
Reconstruction	Magn./Phase
Measurements	1
Multiple series	Off

Resolution - Common

FoV read	360 mm
FoV phase	75.0 %
Slice thickness	8.0 mm
Base resolution	192
Phase resolution	100 %
Phase partial Fourier	6/8
Trajectory	Cartesian
Interpolation	On

Resolution - iPAT

PAT mode	GRAPPA
Accel. factor PE	2
Ref. lines PE	24
Reference scan mode	Integrated

Resolution - Filter Image

Image Filter	Off
Distortion Corr.	On
Mode	2D
Unfiltered images	Off
Prescan Normalize	Off
Normalize	Off
B1 filter	Off

Resolution - Filter Rawdata

Raw filter	On
Elliptical filter	Off
POCS	Off

Geometry - Common

Slice group	1
Slices	1
Dist. factor	25 %
Position	Isocenter
Orientation	Transversal
Phase enc. dir.	A >> P
FoV read	360 mm
FoV phase	75.0 %
Slice thickness	8.0 mm
TR	378.98 ms
Multi-slice mode	Sequential
Series	Interleaved
Concatenations	1

Geometry - AutoAlign

Slice group	1
Position	Isocenter
Orientation	Transversal
Phase enc. dir.	A >> P
AutoAlign	---
Initial Position	Isocenter
Phase	0.0 mm
Read	0.0 mm
Shift	0.0 mm
Initial Rotation	0.00 deg
Initial Orientation	Transversal

Geometry - Saturation

Fat suppr.	None
Wrap-up Magn.	None
Special sat.	None

Geometry - Navigator

System - Miscellaneous

Positioning mode	FIX
Table position	H
Table position	0 mm

System - Miscellaneous

MSMA	S - C - T
Sagittal	R >> L
Coronal	A >> P
Transversal	F >> H
Coil Combine Mode	Adaptive Combine
Save uncombined	Off
Matrix Optimization	Off
Coil Focus	Flat
AutoAlign	---
Coil Select Mode	Default

System - Adjustments

B0 Shim mode	Cardiac
Adjust with body coil	On
Confirm freq. adjustment	Off
Assume Dominant Fat	Off
Assume Silicone	Off
Adjustment Tolerance	Auto

System - Adjust Volume

! Position	Isocenter
! Orientation	Transversal
! Rotation	0.00 deg
! A >> P	150 mm
! R >> L	150 mm
! F >> H	150 mm
Reset	Off

System - Tx/Rx

Frequency 1H	63.683250 MHz
Correction factor	1
Gain	High
Img. Scale Cor.	1.000
Reset	Off
? Ref. amplitude 1H	0.000 V

Physio - Signal1

1st Signal/Mode	ECG/Trigger
Average cycle	787 ± 129 ms
Average cycle	No Signal ms
Captured cycle	787 ± 129 ms
Acquisition window	379 ms
Trigger pulse	1
Trigger delay	0 ms
TR	378.98 ms
Concatenations	1
Segments	84
Phases	1
Adaptive Triggering	Off

Physio - Cardiac

Tagging	None
Magn. preparation	Non-sel. IR
TI	260 ms
Fat suppr.	None
Dark blood	Off
FoV read	360 mm
FoV phase	75.0 %
Phase resolution	100 %
Cine	Off
Trajectory	Cartesian
Dummy heartbeats	0
Motion Correction	None

Physio - PACE

Resp. control	Off
Concatenations	1

Sequence - Part 1

Introduction	Off
Dimension	2D
Reordering	Linear
Asymmetric echo	Weak
Contrasts	1
Optimization	Min. TE TR
Multi-slice mode	Sequential
Sequence type	Trufi
Bandwidth	898 Hz/Px

Sequence - Part 2

Define	Shots
Shots per slice	1
Segments	84
Trufi delta freq.	0 Hz
RF pulse type	Fast
Gradient mode	Fast
Excitation	Slice-sel.
Flip angle mode	Constant
Cine	Off

Sequence - Assistant

Mode	Off
Allowed delay	0 s

\\USER\Cardiac Research Protocols\Rapi-STRESS T1\Rapi-STRESS T1 (V2)\REST SSFP_Perf_MBF_MBV_aif_r3 (KELLMAN)

TA: 9.4 s PM: REF Voxel size: 1.9×1.9×8.0 mmPAT: 3 Rel. SNR: 1.00 : tfi

Properties

Prio recon	Off
Load images to viewer	Off
Inline movie	Off
Auto store images	On
Load images to stamp segments	Off
Load images to graphic segments	On
Auto open inline display	Off
Auto close inline display	Off
Start measurement without further preparation	Off
Wait for user to start	On
Start measurements	Single measurement

Routine

Slice group	1
Slices	3
Dist. factor	100 %
Position	Isocenter
Orientation	Transversal
Phase enc. dir.	A >> P
AutoAlign	---
Phase oversampling	0 %
FoV read	360 mm
FoV phase	75.0 %
Slice thickness	8.0 mm
TR	142.00 ms
TE	1.04 ms
Averages	1
Concatenations	1
Filter	Distortion Corr.(2D)
Coil elements	BO1-3;SP3,4

Contrast - Common

TR	142.00 ms
TE	1.04 ms
Magn. preparation	Non-sel. SR perf
TI	105 ms
Flip angle	50 deg
Fat suppr.	Fat sat.
Wrap-up Magn.	None

Contrast - Dynamic

Averages	1
Averaging mode	Long term
Reconstruction	Magnitude
Measurements	12
Pause after meas. 1	0.0 s
Pause after meas. 2	0.0 s
Pause after meas. 3	0.0 s
Pause after meas. 4	0.0 s
Pause after meas. 5	0.0 s
Pause after meas. 6	0.0 s
Pause after meas. 7	0.0 s
Pause after meas. 8	0.0 s
Pause after meas. 9	0.0 s
Pause after meas. 10	0.0 s
Pause after meas. 11	0.0 s
Proton Dens. Maps	3
Multiple series	Off

Resolution - Common

FoV read	360 mm
FoV phase	75.0 %
Slice thickness	8.0 mm
Base resolution	192
Phase resolution	77 %
Phase partial Fourier	6/8
Trajectory	Cartesian
Interpolation	Off

Resolution - iPAT

PAT mode	GRAPPA
Accel. factor PE	3
Reference scan mode	T-PAT

Resolution - Filter Image

Image Filter	Off
Distortion Corr.	On
Mode	2D
Unfiltered images	Off
Prescan Normalize	Off
Normalize	Off
B1 filter	Off

Resolution - Filter Rawdata

Raw filter	Off
Elliptical filter	Off
POCS	Off

Geometry - Common

Slice group	1
Slices	3
Dist. factor	100 %
Position	Isocenter
Orientation	Transversal
Phase enc. dir.	A >> P
FoV read	360 mm
FoV phase	75.0 %
Slice thickness	8.0 mm
TR	142.00 ms
Multi-slice mode	Single shot
Series	Ascending
Concatenations	1

Geometry - AutoAlign

Slice group	1
Position	Isocenter
Orientation	Transversal
Phase enc. dir.	A >> P
AutoAlign	---
Initial Position	Isocenter
Phase	0.0 mm
Read	0.0 mm
Shift	0.0 mm
Initial Rotation	0.00 deg
Initial Orientation	Transversal

Geometry - Saturation

Fat suppr.	Fat sat.
Wrap-up Magn.	None

Geometry - Saturation

Special sat.	None

Geometry - Navigator

System - Miscellaneous

Positioning mode	REF
Table position	H
Table position	0 mm
MSMA	S - C - T
Sagittal	R >> L
Coronal	A >> P
Transversal	F >> H
Coil Combine Mode	Sum of Squares
Save uncombined	Off
Matrix Optimization	Off
Coil Focus	Flat
AutoAlign	---
Coil Select Mode	Default

System - Adjustments

B0 Shim mode	Cardiac
Adjust with body coil	On
Confirm freq. adjustment	Off
Assume Dominant Fat	Off
Assume Silicone	Off
Adjustment Tolerance	Auto

System - Adjust Volume

! Position	Isocenter
! Orientation	Transversal
! Rotation	0.00 deg
! A >> P	150 mm
! R >> L	150 mm
! F >> H	150 mm
Reset	Off

System - Tx/Rx

Frequency 1H	63.683250 MHz
Correction factor	1
Gain	High
Img. Scale Cor.	1.000
Reset	Off
? Ref. amplitude 1H	0.000 V

Physio - Signal1

1st Signal/Mode	ECG/Trigger
Average cycle	787 ± 129 ms
Average cycle	No Signal ms
Captured cycle	787 ± 129 ms
Acquisition window	493 ms
Trigger pulse	1
Trigger delay	0 ms
TR	142.00 ms
Concatenations	1
Segments	37
Phases	1
Adaptive Triggering	Off

Physio - Cardiac

Tagging	None
Magn. preparation	Non-sel. SR perf
TI	105 ms
Fat suppr.	Fat sat.

Physio - Cardiac

Dark blood	Off
FoV read	360 mm
FoV phase	75.0 %
Phase resolution	77 %
Cine	Off
Trajectory	Cartesian
Dummy heartbeats	0
Motion Correction	None

Physio - PACE

Resp. control	Off
Concatenations	1

Inline - Common

Subtract	Off
Measurements	12
StdDev	Off
Motion Correction	None
Save original images	On

Inline - Cardiac

Inline Evaluation	Off
Magn. preparation	Non-sel. SR perf
TE	1.04 ms
TR	142.00 ms
Motion Correction	None
Save original images	On

Inline - MIP

MIP-Sag	Off
MIP-Cor	Off
MIP-Tra	Off
MIP-Time	Off
Save original images	On

Inline - Composing

Inline Composing	Off
Distortion Corr.	On
Mode	2D
Unfiltered images	Off

Sequence - Part 1

Introduction	Off
Dimension	2D
Reordering	Linear
Asymmetric echo	Allowed
Optimization	Min. TE
Multi-slice mode	Single shot
Echo spacing	2.5 ms
Sequence type	Trufi
Bandwidth	1085 Hz/Px

Sequence - Part 2

Define	Shots
Shots per slice	1
EPI factor	1
Segments	37
Trufi delta freq.	0 Hz
RF pulse type	Fast
Gradient mode	Fast
Excitation	Slice-sel.
Flip angle mode	Constant
Cine	Off

Sequence - Special

AIF Images	On
AIF SR Preparation	SR_PERF
Scan	Test
Gadgetron IPR	PERF2
Temporal Filter	On
Filter Method	Gaussian
Filter Strength	Medium

Sequence - Assistant

Mode	Min flip angle
Min flip angle	45 deg
Allowed delay	100 s

\\USER\Cardiac Research Protocols\Rapi-STRESS T1\Rapi-STRESS T1 (V2)\MidShMOLLI_192i_d11_nFilt (TD=0)

TA: 7.1 s PM: REF Voxel size: 0.9×0.9×8.0 mmPAT: 2 Rel. SNR: 1.00 : tfi

Properties

Prio recon	Off
Load images to viewer	On
Inline movie	Off
Auto store images	On
Load images to stamp segments	Off
Load images to graphic segments	On
Auto open inline display	Off
Auto close inline display	Off
Start measurement without further preparation	Off
Wait for user to start	Off
Start measurements	Single measurement

Routine

Slice group	1
Slices	1
Dist. factor	25 %
Position	Isocenter
Orientation	Transversal
Phase enc. dir.	A >> P
AutoAlign	---
Phase oversampling	0 %
FoV read	360 mm
FoV phase	75.0 %
Slice thickness	8.0 mm
TR	378.98 ms
TE	1.07 ms
Averages	1
Concatenations	1
Filter	Raw filter, Distortion Corr.(2D)
Coil elements	BO2;SP2,3

Contrast - Common

TR	378.98 ms
TE	1.07 ms
Magn. preparation	Non-sel. IR
TI	260 ms
Flip angle	35 deg
Fat suppr.	None
Wrap-up Magn.	None

Contrast - Dynamic

Averages	1
Averaging mode	Short term
Reconstruction	Magn./Phase
Measurements	1
Multiple series	Off

Resolution - Common

FoV read	360 mm
FoV phase	75.0 %
Slice thickness	8.0 mm
Base resolution	192
Phase resolution	100 %
Phase partial Fourier	6/8
Trajectory	Cartesian
Interpolation	On

Resolution - iPAT

PAT mode	GRAPPA
Accel. factor PE	2
Ref. lines PE	24
Reference scan mode	Integrated

Resolution - Filter Image

Image Filter	Off
Distortion Corr.	On
Mode	2D
Unfiltered images	Off
Prescan Normalize	Off
Normalize	Off
B1 filter	Off

Resolution - Filter Rawdata

Raw filter	On
Elliptical filter	Off
POCS	Off

Geometry - Common

Slice group	1
Slices	1
Dist. factor	25 %
Position	Isocenter
Orientation	Transversal
Phase enc. dir.	A >> P
FoV read	360 mm
FoV phase	75.0 %
Slice thickness	8.0 mm
TR	378.98 ms
Multi-slice mode	Sequential
Series	Interleaved
Concatenations	1

Geometry - AutoAlign

Slice group	1
Position	Isocenter
Orientation	Transversal
Phase enc. dir.	A >> P
AutoAlign	---
Initial Position	Isocenter
Phase	0.0 mm
Read	0.0 mm
Shift	0.0 mm
Initial Rotation	0.00 deg
Initial Orientation	Transversal

Geometry - Saturation

Fat suppr.	None
Wrap-up Magn.	None
Special sat.	None

Geometry - Navigator

System - Miscellaneous

Positioning mode	REF
Table position	H
Table position	0 mm

System - Miscellaneous

MSMA	S - C - T
Sagittal	R >> L
Coronal	A >> P
Transversal	F >> H
Coil Combine Mode	Adaptive Combine
Save uncombined	Off
Matrix Optimization	Off
Coil Focus	Flat
AutoAlign	---
Coil Select Mode	Default

System - Adjustments

B0 Shim mode	Cardiac
Adjust with body coil	On
Confirm freq. adjustment	Off
Assume Dominant Fat	Off
Assume Silicone	Off
Adjustment Tolerance	Auto

System - Adjust Volume

! Position	Isocenter
! Orientation	Transversal
! Rotation	0.00 deg
! A >> P	150 mm
! R >> L	150 mm
! F >> H	150 mm
Reset	Off

System - Tx/Rx

Frequency 1H	63.683250 MHz
Correction factor	1
Gain	High
Img. Scale Cor.	1.000
Reset	Off
? Ref. amplitude 1H	0.000 V

Physio - Signal1

1st Signal/Mode	ECG/Trigger
Average cycle	787 ± 129 ms
Average cycle	No Signal ms
Captured cycle	787 ± 129 ms
Acquisition window	379 ms
Trigger pulse	1
Trigger delay	0 ms
TR	378.98 ms
Concatenations	1
Segments	84
Phases	1
Adaptive Triggering	Off

Physio - Cardiac

Tagging	None
Magn. preparation	Non-sel. IR
TI	260 ms
Fat suppr.	None
Dark blood	Off
FoV read	360 mm
FoV phase	75.0 %
Phase resolution	100 %
Cine	Off
Trajectory	Cartesian
Dummy heartbeats	0
Motion Correction	None

Physio - PACE

Resp. control	Off
Concatenations	1

Sequence - Part 1

Introduction	Off
Dimension	2D
Reordering	Linear
Asymmetric echo	Weak
Contrasts	1
Optimization	Min. TE TR
Multi-slice mode	Sequential
Sequence type	Trufi
Bandwidth	898 Hz/Px

Sequence - Part 2

Define	Shots
Shots per slice	1
Segments	84
Trufi delta freq.	0 Hz
RF pulse type	Fast
Gradient mode	Fast
Excitation	Slice-sel.
Flip angle mode	Constant
Cine	Off

Sequence - Assistant

Mode	Off
Allowed delay	0 s

\\USER\Cardiac Research Protocols\Rapi-STRESS T1\Rapi-STRESS T1 (V2)\ShMOLLI_192i_d11_nFilt (TD=0)

TA: 7.1 s PM: FIX Voxel size: 0.9×0.9×8.0 mmPAT: 2 Rel. SNR: 1.00 : tfi

Properties

Prio recon	Off
Load images to viewer	On
Inline movie	Off
Auto store images	On
Load images to stamp segments	Off
Load images to graphic segments	On
Auto open inline display	Off
Auto close inline display	Off
Start measurement without further preparation	On
Wait for user to start	Off
Start measurements	Single measurement

Routine

Slice group	1
Slices	1
Dist. factor	25 %
Position	Isocenter
Orientation	Transversal
Phase enc. dir.	A >> P
AutoAlign	---
Phase oversampling	0 %
FoV read	360 mm
FoV phase	75.0 %
Slice thickness	8.0 mm
TR	378.98 ms
TE	1.07 ms
Averages	1
Concatenations	1
Filter	Raw filter, Distortion Corr.(2D)
Coil elements	BO2;SP2,3

Contrast - Common

TR	378.98 ms
TE	1.07 ms
Magn. preparation	Non-sel. IR
TI	260 ms
Flip angle	35 deg
Fat suppr.	None
Wrap-up Magn.	None

Contrast - Dynamic

Averages	1
Averaging mode	Short term
Reconstruction	Magn./Phase
Measurements	1
Multiple series	Off

Resolution - Common

FoV read	360 mm
FoV phase	75.0 %
Slice thickness	8.0 mm
Base resolution	192
Phase resolution	100 %
Phase partial Fourier	6/8
Trajectory	Cartesian
Interpolation	On

Resolution - iPAT

PAT mode	GRAPPA
Accel. factor PE	2
Ref. lines PE	24
Reference scan mode	Integrated

Resolution - Filter Image

Image Filter	Off
Distortion Corr.	On
Mode	2D
Unfiltered images	Off
Prescan Normalize	Off
Normalize	Off
B1 filter	Off

Resolution - Filter Rawdata

Raw filter	On
Elliptical filter	Off
POCS	Off

Geometry - Common

Slice group	1
Slices	1
Dist. factor	25 %
Position	Isocenter
Orientation	Transversal
Phase enc. dir.	A >> P
FoV read	360 mm
FoV phase	75.0 %
Slice thickness	8.0 mm
TR	378.98 ms
Multi-slice mode	Sequential
Series	Interleaved
Concatenations	1

Geometry - AutoAlign

Slice group	1
Position	Isocenter
Orientation	Transversal
Phase enc. dir.	A >> P
AutoAlign	---
Initial Position	Isocenter
Phase	0.0 mm
Read	0.0 mm
Shift	0.0 mm
Initial Rotation	0.00 deg
Initial Orientation	Transversal

Geometry - Saturation

Fat suppr.	None
Wrap-up Magn.	None
Special sat.	None

Geometry - Navigator

System - Miscellaneous

Positioning mode	FIX
Table position	H
Table position	0 mm

System - Miscellaneous

MSMA	S - C - T
Sagittal	R >> L
Coronal	A >> P
Transversal	F >> H
Coil Combine Mode	Adaptive Combine
Save uncombined	Off
Matrix Optimization	Off
Coil Focus	Flat
AutoAlign	---
Coil Select Mode	Default

System - Adjustments

B0 Shim mode	Cardiac
Adjust with body coil	On
Confirm freq. adjustment	Off
Assume Dominant Fat	Off
Assume Silicone	Off
Adjustment Tolerance	Auto

System - Adjust Volume

! Position	Isocenter
! Orientation	Transversal
! Rotation	0.00 deg
! A >> P	150 mm
! R >> L	150 mm
! F >> H	150 mm
Reset	Off

System - Tx/Rx

Frequency 1H	63.683250 MHz
Correction factor	1
Gain	High
Img. Scale Cor.	1.000
Reset	Off
? Ref. amplitude 1H	0.000 V

Physio - Signal1

1st Signal/Mode	ECG/Trigger
Average cycle	787 ± 129 ms
Average cycle	No Signal ms
Captured cycle	787 ± 129 ms
Acquisition window	379 ms
Trigger pulse	1
Trigger delay	0 ms
TR	378.98 ms
Concatenations	1
Segments	84
Phases	1
Adaptive Triggering	Off

Physio - Cardiac

Tagging	None
Magn. preparation	Non-sel. IR
TI	260 ms
Fat suppr.	None
Dark blood	Off
FoV read	360 mm
FoV phase	75.0 %
Phase resolution	100 %
Cine	Off
Trajectory	Cartesian
Dummy heartbeats	0
Motion Correction	None

Physio - PACE

Resp. control	Off
Concatenations	1

Sequence - Part 1

Introduction	Off
Dimension	2D
Reordering	Linear
Asymmetric echo	Weak
Contrasts	1
Optimization	Min. TE TR
Multi-slice mode	Sequential
Sequence type	Trufi
Bandwidth	898 Hz/Px

Sequence - Part 2

Define	Shots
Shots per slice	1
Segments	84
Trufi delta freq.	0 Hz
RF pulse type	Fast
Gradient mode	Fast
Excitation	Slice-sel.
Flip angle mode	Constant
Cine	Off

Sequence - Assistant

Mode	Off
Allowed delay	0 s

\\USER\Cardiac Research Protocols\Rapi-STRESS T1\Rapi-STRESS T1 (V2)\ShMOLLI_192i_d11_nFilt (TD=0)

TA: 7.1 s PM: FIX Voxel size: 0.9×0.9×8.0 mmPAT: 2 Rel. SNR: 1.00 : tfi

Properties

Prio recon	Off
Load images to viewer	On
Inline movie	Off
Auto store images	On
Load images to stamp segments	Off
Load images to graphic segments	On
Auto open inline display	Off
Auto close inline display	Off
Start measurement without further preparation	On
Wait for user to start	Off
Start measurements	Single measurement

Routine

Slice group	1
Slices	1
Dist. factor	25 %
Position	Isocenter
Orientation	Transversal
Phase enc. dir.	A >> P
AutoAlign	---
Phase oversampling	0 %
FoV read	360 mm
FoV phase	75.0 %
Slice thickness	8.0 mm
TR	378.98 ms
TE	1.07 ms
Averages	1
Concatenations	1
Filter	Raw filter, Distortion Corr.(2D)
Coil elements	BO2;SP2,3

Contrast - Common

TR	378.98 ms
TE	1.07 ms
Magn. preparation	Non-sel. IR
TI	260 ms
Flip angle	35 deg
Fat suppr.	None
Wrap-up Magn.	None

Contrast - Dynamic

Averages	1
Averaging mode	Short term
Reconstruction	Magn./Phase
Measurements	1
Multiple series	Off

Resolution - Common

FoV read	360 mm
FoV phase	75.0 %
Slice thickness	8.0 mm
Base resolution	192
Phase resolution	100 %
Phase partial Fourier	6/8
Trajectory	Cartesian
Interpolation	On

Resolution - iPAT

PAT mode	GRAPPA
Accel. factor PE	2
Ref. lines PE	24
Reference scan mode	Integrated

Resolution - Filter Image

Image Filter	Off
Distortion Corr.	On
Mode	2D
Unfiltered images	Off
Prescan Normalize	Off
Normalize	Off
B1 filter	Off

Resolution - Filter Rawdata

Raw filter	On
Elliptical filter	Off
POCS	Off

Geometry - Common

Slice group	1
Slices	1
Dist. factor	25 %
Position	Isocenter
Orientation	Transversal
Phase enc. dir.	A >> P
FoV read	360 mm
FoV phase	75.0 %
Slice thickness	8.0 mm
TR	378.98 ms
Multi-slice mode	Sequential
Series	Interleaved
Concatenations	1

Geometry - AutoAlign

Slice group	1
Position	Isocenter
Orientation	Transversal
Phase enc. dir.	A >> P
AutoAlign	---
Initial Position	Isocenter
Phase	0.0 mm
Read	0.0 mm
Shift	0.0 mm
Initial Rotation	0.00 deg
Initial Orientation	Transversal

Geometry - Saturation

Fat suppr.	None
Wrap-up Magn.	None
Special sat.	None

Geometry - Navigator

System - Miscellaneous

Positioning mode	FIX
Table position	H
Table position	0 mm

System - Miscellaneous

MSMA	S - C - T
Sagittal	R >> L
Coronal	A >> P
Transversal	F >> H
Coil Combine Mode	Adaptive Combine
Save uncombined	Off
Matrix Optimization	Off
Coil Focus	Flat
AutoAlign	---
Coil Select Mode	Default

System - Adjustments

B0 Shim mode	Cardiac
Adjust with body coil	On
Confirm freq. adjustment	Off
Assume Dominant Fat	Off
Assume Silicone	Off
Adjustment Tolerance	Auto

System - Adjust Volume

! Position	Isocenter
! Orientation	Transversal
! Rotation	0.00 deg
! A >> P	150 mm
! R >> L	150 mm
! F >> H	150 mm
Reset	Off

System - Tx/Rx

Frequency 1H	63.683250 MHz
Correction factor	1
Gain	High
Img. Scale Cor.	1.000
Reset	Off
? Ref. amplitude 1H	0.000 V

Physio - Signal1

1st Signal/Mode	ECG/Trigger
Average cycle	787 ± 129 ms
Average cycle	No Signal ms
Captured cycle	787 ± 129 ms
Acquisition window	379 ms
Trigger pulse	1
Trigger delay	0 ms
TR	378.98 ms
Concatenations	1
Segments	84
Phases	1
Adaptive Triggering	Off

Physio - Cardiac

Tagging	None
Magn. preparation	Non-sel. IR
TI	260 ms
Fat suppr.	None
Dark blood	Off
FoV read	360 mm
FoV phase	75.0 %
Phase resolution	100 %
Cine	Off
Trajectory	Cartesian
Dummy heartbeats	0
Motion Correction	None

Physio - PACE

Resp. control	Off
Concatenations	1

Sequence - Part 1

Introduction	Off
Dimension	2D
Reordering	Linear
Asymmetric echo	Weak
Contrasts	1
Optimization	Min. TE TR
Multi-slice mode	Sequential
Sequence type	Trufi
Bandwidth	898 Hz/Px

Sequence - Part 2

Define	Shots
Shots per slice	1
Segments	84
Trufi delta freq.	0 Hz
RF pulse type	Fast
Gradient mode	Fast
Excitation	Slice-sel.
Flip angle mode	Constant
Cine	Off

Sequence - Assistant

Mode	Off
Allowed delay	0 s

\\USER\Cardiac Research Protocols\Rapi-STRESS T1\Rapi-STRESS T1 (V2)\ShMOLLI_192i_d11_nFilt (TD=0)

TA: 7.1 s PM: FIX Voxel size: 0.9×0.9×8.0 mmPAT: 2 Rel. SNR: 1.00 : tfi

Properties

Prio recon	Off
Load images to viewer	On
Inline movie	Off
Auto store images	On
Load images to stamp segments	Off
Load images to graphic segments	On
Auto open inline display	Off
Auto close inline display	Off
Start measurement without further preparation	On
Wait for user to start	Off
Start measurements	Single measurement

Routine

Slice group	1
Slices	1
Dist. factor	25 %
Position	Isocenter
Orientation	Transversal
Phase enc. dir.	A >> P
AutoAlign	---
Phase oversampling	0 %
FoV read	360 mm
FoV phase	75.0 %
Slice thickness	8.0 mm
TR	378.98 ms
TE	1.07 ms
Averages	1
Concatenations	1
Filter	Raw filter, Distortion Corr.(2D)
Coil elements	BO2;SP2,3

Contrast - Common

TR	378.98 ms
TE	1.07 ms
Magn. preparation	Non-sel. IR
TI	260 ms
Flip angle	35 deg
Fat suppr.	None
Wrap-up Magn.	None

Contrast - Dynamic

Averages	1
Averaging mode	Short term
Reconstruction	Magn./Phase
Measurements	1
Multiple series	Off

Resolution - Common

FoV read	360 mm
FoV phase	75.0 %
Slice thickness	8.0 mm
Base resolution	192
Phase resolution	100 %
Phase partial Fourier	6/8
Trajectory	Cartesian
Interpolation	On

Resolution - iPAT

PAT mode	GRAPPA
Accel. factor PE	2
Ref. lines PE	24
Reference scan mode	Integrated

Resolution - Filter Image

Image Filter	Off
Distortion Corr.	On
Mode	2D
Unfiltered images	Off
Prescan Normalize	Off
Normalize	Off
B1 filter	Off

Resolution - Filter Rawdata

Raw filter	On
Elliptical filter	Off
POCS	Off

Geometry - Common

Slice group	1
Slices	1
Dist. factor	25 %
Position	Isocenter
Orientation	Transversal
Phase enc. dir.	A >> P
FoV read	360 mm
FoV phase	75.0 %
Slice thickness	8.0 mm
TR	378.98 ms
Multi-slice mode	Sequential
Series	Interleaved
Concatenations	1

Geometry - AutoAlign

Slice group	1
Position	Isocenter
Orientation	Transversal
Phase enc. dir.	A >> P
AutoAlign	---
Initial Position	Isocenter
Phase	0.0 mm
Read	0.0 mm
Shift	0.0 mm
Initial Rotation	0.00 deg
Initial Orientation	Transversal

Geometry - Saturation

Fat suppr.	None
Wrap-up Magn.	None
Special sat.	None

Geometry - Navigator

System - Miscellaneous

Positioning mode	FIX
Table position	H
Table position	0 mm

System - Miscellaneous

MSMA	S - C - T
Sagittal	R >> L
Coronal	A >> P
Transversal	F >> H
Coil Combine Mode	Adaptive Combine
Save uncombined	Off
Matrix Optimization	Off
Coil Focus	Flat
AutoAlign	---
Coil Select Mode	Default

System - Adjustments

B0 Shim mode	Cardiac
Adjust with body coil	On
Confirm freq. adjustment	Off
Assume Dominant Fat	Off
Assume Silicone	Off
Adjustment Tolerance	Auto

System - Adjust Volume

! Position	Isocenter
! Orientation	Transversal
! Rotation	0.00 deg
! A >> P	150 mm
! R >> L	150 mm
! F >> H	150 mm
Reset	Off

System - Tx/Rx

Frequency 1H	63.683250 MHz
Correction factor	1
Gain	High
Img. Scale Cor.	1.000
Reset	Off
? Ref. amplitude 1H	0.000 V

Physio - Signal1

1st Signal/Mode	ECG/Trigger
Average cycle	787 ± 129 ms
Average cycle	No Signal ms
Captured cycle	787 ± 129 ms
Acquisition window	379 ms
Trigger pulse	1
Trigger delay	0 ms
TR	378.98 ms
Concatenations	1
Segments	84
Phases	1
Adaptive Triggering	Off

Physio - Cardiac

Tagging	None
Magn. preparation	Non-sel. IR
TI	260 ms
Fat suppr.	None
Dark blood	Off
FoV read	360 mm
FoV phase	75.0 %
Phase resolution	100 %
Cine	Off
Trajectory	Cartesian
Dummy heartbeats	0
Motion Correction	None

Physio - PACE

Resp. control	Off
Concatenations	1

Sequence - Part 1

Introduction	Off
Dimension	2D
Reordering	Linear
Asymmetric echo	Weak
Contrasts	1
Optimization	Min. TE TR
Multi-slice mode	Sequential
Sequence type	Trufi
Bandwidth	898 Hz/Px

Sequence - Part 2

Define	Shots
Shots per slice	1
Segments	84
Trufi delta freq.	0 Hz
RF pulse type	Fast
Gradient mode	Fast
Excitation	Slice-sel.
Flip angle mode	Constant
Cine	Off

Sequence - Assistant

Mode	Off
Allowed delay	0 s

\\USER\Cardiac Research Protocols\Rapi-STRESS T1\Rapi-STRESS T1 (V2)\MidShMOLLI_192i_d11_nFilt (TD=0)

TA: 7.1 s PM: REF Voxel size: 0.9×0.9×8.0 mmPAT: 2 Rel. SNR: 1.00 : tfi

Properties

Prio recon	Off
Load images to viewer	On
Inline movie	Off
Auto store images	On
Load images to stamp segments	Off
Load images to graphic segments	On
Auto open inline display	Off
Auto close inline display	Off
Start measurement without further preparation	Off
Wait for user to start	Off
Start measurements	Single measurement

Routine

Slice group	1
Slices	1
Dist. factor	25 %
Position	Isocenter
Orientation	Transversal
Phase enc. dir.	A >> P
AutoAlign	---
Phase oversampling	0 %
FoV read	360 mm
FoV phase	75.0 %
Slice thickness	8.0 mm
TR	378.98 ms
TE	1.07 ms
Averages	1
Concatenations	1
Filter	Raw filter, Distortion Corr.(2D)
Coil elements	BO2;SP2,3

Contrast - Common

TR	378.98 ms
TE	1.07 ms
Magn. preparation	Non-sel. IR
TI	260 ms
Flip angle	35 deg
Fat suppr.	None
Wrap-up Magn.	None

Contrast - Dynamic

Averages	1
Averaging mode	Short term
Reconstruction	Magn./Phase
Measurements	1
Multiple series	Off

Resolution - Common

FoV read	360 mm
FoV phase	75.0 %
Slice thickness	8.0 mm
Base resolution	192
Phase resolution	100 %
Phase partial Fourier	6/8
Trajectory	Cartesian
Interpolation	On

Resolution - iPAT

PAT mode	GRAPPA
Accel. factor PE	2
Ref. lines PE	24
Reference scan mode	Integrated

Resolution - Filter Image

Image Filter	Off
Distortion Corr.	On
Mode	2D
Unfiltered images	Off
Prescan Normalize	Off
Normalize	Off
B1 filter	Off

Resolution - Filter Rawdata

Raw filter	On
Elliptical filter	Off
POCS	Off

Geometry - Common

Slice group	1
Slices	1
Dist. factor	25 %
Position	Isocenter
Orientation	Transversal
Phase enc. dir.	A >> P
FoV read	360 mm
FoV phase	75.0 %
Slice thickness	8.0 mm
TR	378.98 ms
Multi-slice mode	Sequential
Series	Interleaved
Concatenations	1

Geometry - AutoAlign

Slice group	1
Position	Isocenter
Orientation	Transversal
Phase enc. dir.	A >> P
AutoAlign	---
Initial Position	Isocenter
Phase	0.0 mm
Read	0.0 mm
Shift	0.0 mm
Initial Rotation	0.00 deg
Initial Orientation	Transversal

Geometry - Saturation

Fat suppr.	None
Wrap-up Magn.	None
Special sat.	None

Geometry - Navigator

System - Miscellaneous

Positioning mode	REF
Table position	H
Table position	0 mm

System - Miscellaneous

MSMA	S - C - T
Sagittal	R >> L
Coronal	A >> P
Transversal	F >> H
Coil Combine Mode	Adaptive Combine
Save uncombined	Off
Matrix Optimization	Off
Coil Focus	Flat
AutoAlign	---
Coil Select Mode	Default

System - Adjustments

B0 Shim mode	Cardiac
Adjust with body coil	On
Confirm freq. adjustment	Off
Assume Dominant Fat	Off
Assume Silicone	Off
Adjustment Tolerance	Auto

System - Adjust Volume

! Position	Isocenter
! Orientation	Transversal
! Rotation	0.00 deg
! A >> P	150 mm
! R >> L	150 mm
! F >> H	150 mm
Reset	Off

System - Tx/Rx

Frequency 1H	63.683250 MHz
Correction factor	1
Gain	High
Img. Scale Cor.	1.000
Reset	Off
? Ref. amplitude 1H	0.000 V

Physio - Signal1

1st Signal/Mode	ECG/Trigger
Average cycle	787 ± 129 ms
Average cycle	No Signal ms
Captured cycle	787 ± 129 ms
Acquisition window	379 ms
Trigger pulse	1
Trigger delay	0 ms
TR	378.98 ms
Concatenations	1
Segments	84
Phases	1
Adaptive Triggering	Off

Physio - Cardiac

Tagging	None
Magn. preparation	Non-sel. IR
TI	260 ms
Fat suppr.	None
Dark blood	Off
FoV read	360 mm
FoV phase	75.0 %
Phase resolution	100 %
Cine	Off
Trajectory	Cartesian
Dummy heartbeats	0
Motion Correction	None

Physio - PACE

Resp. control	Off
Concatenations	1

Sequence - Part 1

Introduction	Off
Dimension	2D
Reordering	Linear
Asymmetric echo	Weak
Contrasts	1
Optimization	Min. TE TR
Multi-slice mode	Sequential
Sequence type	Trufi
Bandwidth	898 Hz/Px

Sequence - Part 2

Define	Shots
Shots per slice	1
Segments	84
Trufi delta freq.	0 Hz
RF pulse type	Fast
Gradient mode	Fast
Excitation	Slice-sel.
Flip angle mode	Constant
Cine	Off

Sequence - Assistant

Mode	Off
Allowed delay	0 s

\\USER\Cardiac Research Protocols\Rapi-STRESS T1\Rapi-STRESS T1 (V2)\T1Map_ShortT1(TD=0)lowHR

TA: 0:17 PM: FIX Voxel size: 1.4×1.4×8.0 mmPAT: 2 Rel. SNR: 1.00 : tfi

Properties

Prio recon	Off
Load images to viewer	On
Inline movie	Off
Auto store images	On
Load images to stamp segments	On
Load images to graphic segments	On
Auto open inline display	Off
Auto close inline display	Off
Start measurement without further preparation	On
Wait for user to start	Off
Start measurements	Single measurement

Routine

Slice group	1
Slices	1
Dist. factor	20 %
Position	Isocenter
Orientation	Transversal
Phase enc. dir.	A >> P
AutoAlign	---
Phase oversampling	0 %
FoV read	360 mm
FoV phase	85.2 %
Slice thickness	8.0 mm
TR	359.84 ms
TE	1.13 ms
Averages	1
Concatenations	1
Filter	Distortion Corr.(2D)
Coil elements	BO2;SP2,3

Contrast - Common

TR	359.84 ms
TE	1.13 ms
Magn. preparation	Non-sel. IR T1map
TI	260 ms
Flip angle	35 deg
Fat suppr.	None
Wrap-up Magn.	None

Contrast - Dynamic

Averages	1
Averaging mode	Short term
Reconstruction	Magnitude
Measurements	1
Multiple series	Off

Resolution - Common

FoV read	360 mm
FoV phase	85.2 %
Slice thickness	8.0 mm
Base resolution	256
Phase resolution	66 %
Phase partial Fourier	7/8
Trajectory	Cartesian
Interpolation	Off

Resolution - iPAT

PAT mode	GRAPPA
Accel. factor PE	2
Ref. lines PE	36
Reference scan mode	GRE/separate

Resolution - Filter Image

Image Filter	Off
Distortion Corr.	On
Mode	2D
Unfiltered images	Off
Prescan Normalize	Off
Normalize	Off
B1 filter	Off

Resolution - Filter Rawdata

Raw filter	Off
Elliptical filter	Off
POCS	Off

Geometry - Common

Slice group	1
Slices	1
Dist. factor	20 %
Position	Isocenter
Orientation	Transversal
Phase enc. dir.	A >> P
FoV read	360 mm
FoV phase	85.2 %
Slice thickness	8.0 mm
TR	359.84 ms
Multi-slice mode	Sequential
Series	Base To Apex
Concatenations	1

Geometry - AutoAlign

Slice group	1
Position	Isocenter
Orientation	Transversal
Phase enc. dir.	A >> P
AutoAlign	---
Initial Position	Isocenter
Phase	0.0 mm
Read	0.0 mm
Shift	0.0 mm
Initial Rotation	0.00 deg
Initial Orientation	Transversal

Geometry - Saturation

Fat suppr.	None
Wrap-up Magn.	None
Special sat.	None

Geometry - Navigator

System - Miscellaneous

Positioning mode	FIX
Table position	H
Table position	0 mm

System - Miscellaneous

MSMA	S - C - T
Sagittal	R >> L
Coronal	A >> P
Transversal	F >> H
Coil Combine Mode	Adaptive Combine
Save uncombined	Off
Matrix Optimization	Off
Coil Focus	Flat
AutoAlign	---
Coil Select Mode	Default

System - Adjustments

B0 Shim mode	Cardiac
Adjust with body coil	Off
Confirm freq. adjustment	Off
Assume Dominant Fat	Off
Assume Silicone	Off
Adjustment Tolerance	Auto

System - Adjust Volume

! Position	Isocenter
! Orientation	Transversal
! Rotation	0.00 deg
! A >> P	150 mm
! R >> L	150 mm
! F >> H	150 mm
Reset	Off

System - Tx/Rx

Frequency 1H	63.683250 MHz
Correction factor	1
Gain	High
Img. Scale Cor.	1.000
Reset	Off
? Ref. amplitude 1H	0.000 V

Physio - Signal1

1st Signal/Mode	ECG/Trigger
Average cycle	787 ± 129 ms
Average cycle	No Signal ms
Captured cycle	787 ± 129 ms
Acquisition window	813 ms
Trigger pulse	1
Trigger delay	0 ms
TR	359.84 ms
Concatenations	1
Segments	72
Phases	1
Adaptive Triggering	Off

Physio - Cardiac

Tagging	None
Magn. preparation	Non-sel. IR T1map
TI	260 ms
Fat suppr.	None
Dark blood	Off
FoV read	360 mm
FoV phase	85.2 %
Phase resolution	66 %
Cine	Off
Trajectory	Cartesian
Dummy heartbeats	0
Motion Correction	Standard

Physio - PACE

Resp. control	Breath-hold
Concatenations	1

Sequence - Part 1

Introduction	Off
Dimension	2D
Reordering	Linear
Asymmetric echo	Weak
Contrasts	1
Optimization	Min. TE TR
Multi-slice mode	Sequential
Sequence type	Trufi
Bandwidth	1085 Hz/Px

Sequence - Part 2

Define	Shots
Shots per slice	1
Segments	72
Trufi delta freq.	0 Hz
RF pulse type	Fast
Gradient mode	Fast
Excitation	Slice-sel.
Flip angle mode	Constant
Cine	Off

Sequence - Assistant

Mode	Off
Allowed delay	0 s

\\USER\Cardiac Research Protocols\Rapi-STRESS T1\Rapi-STRESS T1 (V2)\ShMOLLI_192i_d11_nFilt (TD=0)

TA: 7.1 s PM: FIX Voxel size: 0.9×0.9×8.0 mmPAT: 2 Rel. SNR: 1.00 : tfi

Properties

Prio recon	Off
Load images to viewer	On
Inline movie	Off
Auto store images	On
Load images to stamp segments	Off
Load images to graphic segments	On
Auto open inline display	Off
Auto close inline display	Off
Start measurement without further preparation	On
Wait for user to start	Off
Start measurements	Single measurement

Routine

Slice group	1
Slices	1
Dist. factor	25 %
Position	Isocenter
Orientation	Transversal
Phase enc. dir.	A >> P
AutoAlign	---
Phase oversampling	0 %
FoV read	360 mm
FoV phase	75.0 %
Slice thickness	8.0 mm
TR	378.98 ms
TE	1.07 ms
Averages	1
Concatenations	1
Filter	Raw filter, Distortion Corr.(2D)
Coil elements	BO2;SP2,3

Contrast - Common

TR	378.98 ms
TE	1.07 ms
Magn. preparation	Non-sel. IR
TI	260 ms
Flip angle	35 deg
Fat suppr.	None
Wrap-up Magn.	None

Contrast - Dynamic

Averages	1
Averaging mode	Short term
Reconstruction	Magn./Phase
Measurements	1
Multiple series	Off

Resolution - Common

FoV read	360 mm
FoV phase	75.0 %
Slice thickness	8.0 mm
Base resolution	192
Phase resolution	100 %
Phase partial Fourier	6/8
Trajectory	Cartesian
Interpolation	On

Resolution - iPAT

PAT mode	GRAPPA
Accel. factor PE	2
Ref. lines PE	24
Reference scan mode	Integrated

Resolution - Filter Image

Image Filter	Off
Distortion Corr.	On
Mode	2D
Unfiltered images	Off
Prescan Normalize	Off
Normalize	Off
B1 filter	Off

Resolution - Filter Rawdata

Raw filter	On
Elliptical filter	Off
POCS	Off

Geometry - Common

Slice group	1
Slices	1
Dist. factor	25 %
Position	Isocenter
Orientation	Transversal
Phase enc. dir.	A >> P
FoV read	360 mm
FoV phase	75.0 %
Slice thickness	8.0 mm
TR	378.98 ms
Multi-slice mode	Sequential
Series	Interleaved
Concatenations	1

Geometry - AutoAlign

Slice group	1
Position	Isocenter
Orientation	Transversal
Phase enc. dir.	A >> P
AutoAlign	---
Initial Position	Isocenter
Phase	0.0 mm
Read	0.0 mm
Shift	0.0 mm
Initial Rotation	0.00 deg
Initial Orientation	Transversal

Geometry - Saturation

Fat suppr.	None
Wrap-up Magn.	None
Special sat.	None

Geometry - Navigator

System - Miscellaneous

Positioning mode	FIX
Table position	H
Table position	0 mm

System - Miscellaneous

MSMA	S - C - T
Sagittal	R >> L
Coronal	A >> P
Transversal	F >> H
Coil Combine Mode	Adaptive Combine
Save uncombined	Off
Matrix Optimization	Off
Coil Focus	Flat
AutoAlign	---
Coil Select Mode	Default

System - Adjustments

B0 Shim mode	Cardiac
Adjust with body coil	On
Confirm freq. adjustment	Off
Assume Dominant Fat	Off
Assume Silicone	Off
Adjustment Tolerance	Auto

System - Adjust Volume

! Position	Isocenter
! Orientation	Transversal
! Rotation	0.00 deg
! A >> P	150 mm
! R >> L	150 mm
! F >> H	150 mm
Reset	Off

System - Tx/Rx

Frequency 1H	63.683250 MHz
Correction factor	1
Gain	High
Img. Scale Cor.	1.000
Reset	Off
? Ref. amplitude 1H	0.000 V

Physio - Signal1

1st Signal/Mode	ECG/Trigger
Average cycle	787 ± 129 ms
Average cycle	No Signal ms
Captured cycle	787 ± 129 ms
Acquisition window	379 ms
Trigger pulse	1
Trigger delay	0 ms
TR	378.98 ms
Concatenations	1
Segments	84
Phases	1
Adaptive Triggering	Off

Physio - Cardiac

Tagging	None
Magn. preparation	Non-sel. IR
TI	260 ms
Fat suppr.	None
Dark blood	Off
FoV read	360 mm
FoV phase	75.0 %
Phase resolution	100 %
Cine	Off
Trajectory	Cartesian
Dummy heartbeats	0
Motion Correction	None

Physio - PACE

Resp. control	Off
Concatenations	1

Sequence - Part 1

Introduction	Off
Dimension	2D
Reordering	Linear
Asymmetric echo	Weak
Contrasts	1
Optimization	Min. TE TR
Multi-slice mode	Sequential
Sequence type	Trufi
Bandwidth	898 Hz/Px

Sequence - Part 2

Define	Shots
Shots per slice	1
Segments	84
Trufi delta freq.	0 Hz
RF pulse type	Fast
Gradient mode	Fast
Excitation	Slice-sel.
Flip angle mode	Constant
Cine	Off

Sequence - Assistant

Mode	Off
Allowed delay	0 s

\\USER\Cardiac Research Protocols\Rapi-STRESS T1\Rapi-STRESS T1 (V2)\T1Map_ShortT1(TD=0)highHR

TA: 8.7 s PM: FIX Voxel size: 1.9×1.9×8.0 mmPAT: 2 Rel. SNR: 1.00 : tfi

Properties

Prio recon	Off
Load images to viewer	On
Inline movie	Off
Auto store images	On
Load images to stamp segments	On
Load images to graphic segments	On
Auto open inline display	Off
Auto close inline display	Off
Start measurement without further preparation	On
Wait for user to start	Off
Start measurements	Single measurement

Routine

Slice group	1
Slices	1
Dist. factor	20 %
Position	Isocenter
Orientation	Transversal
Phase enc. dir.	A >> P
AutoAlign	---
Phase oversampling	0 %
FoV read	360 mm
FoV phase	85.4 %
Slice thickness	8.0 mm
TR	341.12 ms
TE	1.01 ms
Averages	1
Concatenations	1
Filter	Distortion Corr.(2D)
Coil elements	BO2;SP2,3

Contrast - Common

TR	341.12 ms
TE	1.01 ms
Magn. preparation	Non-sel. IR T1map
TI	260 ms
Flip angle	35 deg
Fat suppr.	None
Wrap-up Magn.	None

Contrast - Dynamic

Averages	1
Averaging mode	Short term
Reconstruction	Magnitude
Measurements	1
Multiple series	Off

Resolution - Common

FoV read	360 mm
FoV phase	85.4 %
Slice thickness	8.0 mm
Base resolution	192
Phase resolution	78 %
Phase partial Fourier	7/8
Trajectory	Cartesian
Interpolation	Off

Resolution - iPAT

PAT mode	GRAPPA
Accel. factor PE	2
Ref. lines PE	36
Reference scan mode	GRE/separate

Resolution - Filter Image

Image Filter	Off
Distortion Corr.	On
Mode	2D
Unfiltered images	Off
Prescan Normalize	Off
Normalize	Off
B1 filter	Off

Resolution - Filter Rawdata

Raw filter	Off
Elliptical filter	Off
POCS	Off

Geometry - Common

Slice group	1
Slices	1
Dist. factor	20 %
Position	Isocenter
Orientation	Transversal
Phase enc. dir.	A >> P
FoV read	360 mm
FoV phase	85.4 %
Slice thickness	8.0 mm
TR	341.12 ms
Multi-slice mode	Sequential
Series	Base To Apex
Concatenations	1

Geometry - AutoAlign

Slice group	1
Position	Isocenter
Orientation	Transversal
Phase enc. dir.	A >> P
AutoAlign	---
Initial Position	Isocenter
Phase	0.0 mm
Read	0.0 mm
Shift	0.0 mm
Initial Rotation	0.00 deg
Initial Orientation	Transversal

Geometry - Saturation

Fat suppr.	None
Wrap-up Magn.	None
Special sat.	None

Geometry - Navigator

System - Miscellaneous

Positioning mode	FIX
Table position	H
Table position	0 mm

System - Miscellaneous

MSMA	S - C - T
Sagittal	R >> L
Coronal	A >> P
Transversal	F >> H
Coil Combine Mode	Adaptive Combine
Save uncombined	Off
Matrix Optimization	Off
Coil Focus	Flat
AutoAlign	---
Coil Select Mode	Default

System - Adjustments

B0 Shim mode	Cardiac
Adjust with body coil	Off
Confirm freq. adjustment	Off
Assume Dominant Fat	Off
Assume Silicone	Off
Adjustment Tolerance	Auto

System - Adjust Volume

! Position	Isocenter
! Orientation	Transversal
! Rotation	0.00 deg
! A >> P	150 mm
! R >> L	150 mm
! F >> H	150 mm
Reset	Off

System - Tx/Rx

Frequency 1H	63.683250 MHz
Correction factor	1
Gain	High
Img. Scale Cor.	1.000
Reset	Off
? Ref. amplitude 1H	0.000 V

Physio - Signal1

1st Signal/Mode	ECG/Trigger
Average cycle	787 ± 129 ms
Average cycle	No Signal ms
Captured cycle	787 ± 129 ms
Acquisition window	704 ms
Trigger pulse	1
Trigger delay	0 ms
TR	341.12 ms
Concatenations	1
Segments	64
Phases	1
Adaptive Triggering	Off

Physio - Cardiac

Tagging	None
Magn. preparation	Non-sel. IR T1map
TI	260 ms
Fat suppr.	None
Dark blood	Off
FoV read	360 mm
FoV phase	85.4 %
Phase resolution	78 %
Cine	Off
Trajectory	Cartesian
Dummy heartbeats	0
Motion Correction	Standard

Physio - PACE

Resp. control	Breath-hold
Concatenations	1

Sequence - Part 1

Introduction	Off
Dimension	2D
Reordering	Linear
Asymmetric echo	Weak
Contrasts	1
Optimization	Min. TE TR
Multi-slice mode	Sequential
Sequence type	Trufi
Bandwidth	1085 Hz/Px

Sequence - Part 2

Define	Shots
Shots per slice	1
Segments	64
Trufi delta freq.	0 Hz
RF pulse type	Fast
Gradient mode	Fast
Excitation	Slice-sel.
Flip angle mode	Constant
Cine	Off

Sequence - Assistant

Mode	Off
Allowed delay	0 s

\\USER\Cardiac Research Protocols\Rapi-STRESS T1\Rapi-STRESS T1 (V2)\ShMOLLI_192i_d11_nFilt (TD=0)

TA: 7.1 s PM: FIX Voxel size: 0.9×0.9×8.0 mmPAT: 2 Rel. SNR: 1.00 : tfi

Properties

Prio recon	Off
Load images to viewer	On
Inline movie	Off
Auto store images	On
Load images to stamp segments	Off
Load images to graphic segments	On
Auto open inline display	Off
Auto close inline display	Off
Start measurement without further preparation	On
Wait for user to start	Off
Start measurements	Single measurement

Routine

Slice group	1
Slices	1
Dist. factor	25 %
Position	Isocenter
Orientation	Transversal
Phase enc. dir.	A >> P
AutoAlign	---
Phase oversampling	0 %
FoV read	360 mm
FoV phase	75.0 %
Slice thickness	8.0 mm
TR	378.98 ms
TE	1.07 ms
Averages	1
Concatenations	1
Filter	Raw filter, Distortion Corr.(2D)
Coil elements	BO2;SP2,3

Contrast - Common

TR	378.98 ms
TE	1.07 ms
Magn. preparation	Non-sel. IR
TI	260 ms
Flip angle	35 deg
Fat suppr.	None
Wrap-up Magn.	None

Contrast - Dynamic

Averages	1
Averaging mode	Short term
Reconstruction	Magn./Phase
Measurements	1
Multiple series	Off

Resolution - Common

FoV read	360 mm
FoV phase	75.0 %
Slice thickness	8.0 mm
Base resolution	192
Phase resolution	100 %
Phase partial Fourier	6/8
Trajectory	Cartesian
Interpolation	On

Resolution - iPAT

PAT mode	GRAPPA
Accel. factor PE	2
Ref. lines PE	24
Reference scan mode	Integrated

Resolution - Filter Image

Image Filter	Off
Distortion Corr.	On
Mode	2D
Unfiltered images	Off
Prescan Normalize	Off
Normalize	Off
B1 filter	Off

Resolution - Filter Rawdata

Raw filter	On
Elliptical filter	Off
POCS	Off

Geometry - Common

Slice group	1
Slices	1
Dist. factor	25 %
Position	Isocenter
Orientation	Transversal
Phase enc. dir.	A >> P
FoV read	360 mm
FoV phase	75.0 %
Slice thickness	8.0 mm
TR	378.98 ms
Multi-slice mode	Sequential
Series	Interleaved
Concatenations	1

Geometry - AutoAlign

Slice group	1
Position	Isocenter
Orientation	Transversal
Phase enc. dir.	A >> P
AutoAlign	---
Initial Position	Isocenter
Phase	0.0 mm
Read	0.0 mm
Shift	0.0 mm
Initial Rotation	0.00 deg
Initial Orientation	Transversal

Geometry - Saturation

Fat suppr.	None
Wrap-up Magn.	None
Special sat.	None

Geometry - Navigator

System - Miscellaneous

Positioning mode	FIX
Table position	H
Table position	0 mm

System - Miscellaneous

MSMA	S - C - T
Sagittal	R >> L
Coronal	A >> P
Transversal	F >> H
Coil Combine Mode	Adaptive Combine
Save uncombined	Off
Matrix Optimization	Off
Coil Focus	Flat
AutoAlign	---
Coil Select Mode	Default

System - Adjustments

B0 Shim mode	Cardiac
Adjust with body coil	On
Confirm freq. adjustment	Off
Assume Dominant Fat	Off
Assume Silicone	Off
Adjustment Tolerance	Auto

System - Adjust Volume

! Position	Isocenter
! Orientation	Transversal
! Rotation	0.00 deg
! A >> P	150 mm
! R >> L	150 mm
! F >> H	150 mm
Reset	Off

System - Tx/Rx

Frequency 1H	63.683250 MHz
Correction factor	1
Gain	High
Img. Scale Cor.	1.000
Reset	Off
? Ref. amplitude 1H	0.000 V

Physio - Signal1

1st Signal/Mode	ECG/Trigger
Average cycle	787 ± 129 ms
Average cycle	No Signal ms
Captured cycle	787 ± 129 ms
Acquisition window	379 ms
Trigger pulse	1
Trigger delay	0 ms
TR	378.98 ms
Concatenations	1
Segments	84
Phases	1
Adaptive Triggering	Off

Physio - Cardiac

Tagging	None
Magn. preparation	Non-sel. IR
TI	260 ms
Fat suppr.	None
Dark blood	Off
FoV read	360 mm
FoV phase	75.0 %
Phase resolution	100 %
Cine	Off
Trajectory	Cartesian
Dummy heartbeats	0
Motion Correction	None

Physio - PACE

Resp. control	Off
Concatenations	1

Sequence - Part 1

Introduction	Off
Dimension	2D
Reordering	Linear
Asymmetric echo	Weak
Contrasts	1
Optimization	Min. TE TR
Multi-slice mode	Sequential
Sequence type	Trufi
Bandwidth	898 Hz/Px

Sequence - Part 2

Define	Shots
Shots per slice	1
Segments	84
Trufi delta freq.	0 Hz
RF pulse type	Fast
Gradient mode	Fast
Excitation	Slice-sel.
Flip angle mode	Constant
Cine	Off

Sequence - Assistant

Mode	Off
Allowed delay	0 s

\\USER\Cardiac Research Protocols\Rapi-STRESS T1\Rapi-STRESS T1 (V2)\MidShMOLLI_192i_d11_nFilt (TD=0)

TA: 7.1 s PM: REF Voxel size: 0.9×0.9×8.0 mmPAT: 2 Rel. SNR: 1.00 : tfi

Properties

Prio recon	Off
Load images to viewer	On
Inline movie	Off
Auto store images	On
Load images to stamp segments	Off
Load images to graphic segments	On
Auto open inline display	Off
Auto close inline display	Off
Start measurement without further preparation	Off
Wait for user to start	Off
Start measurements	Single measurement

Routine

Slice group	1
Slices	1
Dist. factor	25 %
Position	Isocenter
Orientation	Transversal
Phase enc. dir.	A >> P
AutoAlign	---
Phase oversampling	0 %
FoV read	360 mm
FoV phase	75.0 %
Slice thickness	8.0 mm
TR	378.98 ms
TE	1.07 ms
Averages	1
Concatenations	1
Filter	Raw filter, Distortion Corr.(2D)
Coil elements	BO2;SP2,3

Contrast - Common

TR	378.98 ms
TE	1.07 ms
Magn. preparation	Non-sel. IR
TI	260 ms
Flip angle	35 deg
Fat suppr.	None
Wrap-up Magn.	None

Contrast - Dynamic

Averages	1
Averaging mode	Short term
Reconstruction	Magn./Phase
Measurements	1
Multiple series	Off

Resolution - Common

FoV read	360 mm
FoV phase	75.0 %
Slice thickness	8.0 mm
Base resolution	192
Phase resolution	100 %
Phase partial Fourier	6/8
Trajectory	Cartesian
Interpolation	On

Resolution - iPAT

PAT mode	GRAPPA
Accel. factor PE	2
Ref. lines PE	24
Reference scan mode	Integrated

Resolution - Filter Image

Image Filter	Off
Distortion Corr.	On
Mode	2D
Unfiltered images	Off
Prescan Normalize	Off
Normalize	Off
B1 filter	Off

Resolution - Filter Rawdata

Raw filter	On
Elliptical filter	Off
POCS	Off

Geometry - Common

Slice group	1
Slices	1
Dist. factor	25 %
Position	Isocenter
Orientation	Transversal
Phase enc. dir.	A >> P
FoV read	360 mm
FoV phase	75.0 %
Slice thickness	8.0 mm
TR	378.98 ms
Multi-slice mode	Sequential
Series	Interleaved
Concatenations	1

Geometry - AutoAlign

Slice group	1
Position	Isocenter
Orientation	Transversal
Phase enc. dir.	A >> P
AutoAlign	---
Initial Position	Isocenter
Phase	0.0 mm
Read	0.0 mm
Shift	0.0 mm
Initial Rotation	0.00 deg
Initial Orientation	Transversal

Geometry - Saturation

Fat suppr.	None
Wrap-up Magn.	None
Special sat.	None

Geometry - Navigator

System - Miscellaneous

Positioning mode	REF
Table position	H
Table position	0 mm

System - Miscellaneous

MSMA	S - C - T
Sagittal	R >> L
Coronal	A >> P
Transversal	F >> H
Coil Combine Mode	Adaptive Combine
Save uncombined	Off
Matrix Optimization	Off
Coil Focus	Flat
AutoAlign	---
Coil Select Mode	Default

System - Adjustments

B0 Shim mode	Cardiac
Adjust with body coil	On
Confirm freq. adjustment	Off
Assume Dominant Fat	Off
Assume Silicone	Off
Adjustment Tolerance	Auto

System - Adjust Volume

! Position	Isocenter
! Orientation	Transversal
! Rotation	0.00 deg
! A >> P	150 mm
! R >> L	150 mm
! F >> H	150 mm
Reset	Off

System - Tx/Rx

Frequency 1H	63.683250 MHz
Correction factor	1
Gain	High
Img. Scale Cor.	1.000
Reset	Off
? Ref. amplitude 1H	0.000 V

Physio - Signal1

1st Signal/Mode	ECG/Trigger
Average cycle	787 ± 129 ms
Average cycle	No Signal ms
Captured cycle	787 ± 129 ms
Acquisition window	379 ms
Trigger pulse	1
Trigger delay	0 ms
TR	378.98 ms
Concatenations	1
Segments	84
Phases	1
Adaptive Triggering	Off

Physio - Cardiac

Tagging	None
Magn. preparation	Non-sel. IR
TI	260 ms
Fat suppr.	None
Dark blood	Off
FoV read	360 mm
FoV phase	75.0 %
Phase resolution	100 %
Cine	Off
Trajectory	Cartesian
Dummy heartbeats	0
Motion Correction	None

Physio - PACE

Resp. control	Off
Concatenations	1

Sequence - Part 1

Introduction	Off
Dimension	2D
Reordering	Linear
Asymmetric echo	Weak
Contrasts	1
Optimization	Min. TE TR
Multi-slice mode	Sequential
Sequence type	Trufi
Bandwidth	898 Hz/Px

Sequence - Part 2

Define	Shots
Shots per slice	1
Segments	84
Trufi delta freq.	0 Hz
RF pulse type	Fast
Gradient mode	Fast
Excitation	Slice-sel.
Flip angle mode	Constant
Cine	Off

Sequence - Assistant

Mode	Off
Allowed delay	0 s

\\USER\Cardiac Research Protocols\Rapi-STRESS T1\Rapi-STRESS T1 (V2)\T1Map_ShortT1(TD=0)lowHR

TA: 0:17 PM: FIX Voxel size: 1.4×1.4×8.0 mmPAT: 2 Rel. SNR: 1.00 : tfi

Properties

Prio recon	Off
Load images to viewer	On
Inline movie	Off
Auto store images	On
Load images to stamp segments	On
Load images to graphic segments	On
Auto open inline display	Off
Auto close inline display	Off
Start measurement without further preparation	On
Wait for user to start	Off
Start measurements	Single measurement

Routine

Slice group	1
Slices	1
Dist. factor	20 %
Position	Isocenter
Orientation	Transversal
Phase enc. dir.	A >> P
AutoAlign	---
Phase oversampling	0 %
FoV read	360 mm
FoV phase	85.2 %
Slice thickness	8.0 mm
TR	359.84 ms
TE	1.13 ms
Averages	1
Concatenations	1
Filter	Distortion Corr.(2D)
Coil elements	BO2;SP2,3

Contrast - Common

TR	359.84 ms
TE	1.13 ms
Magn. preparation	Non-sel. IR T1map
TI	260 ms
Flip angle	35 deg
Fat suppr.	None
Wrap-up Magn.	None

Contrast - Dynamic

Averages	1
Averaging mode	Short term
Reconstruction	Magnitude
Measurements	1
Multiple series	Off

Resolution - Common

FoV read	360 mm
FoV phase	85.2 %
Slice thickness	8.0 mm
Base resolution	256
Phase resolution	66 %
Phase partial Fourier	7/8
Trajectory	Cartesian
Interpolation	Off

Resolution - iPAT

PAT mode	GRAPPA
Accel. factor PE	2
Ref. lines PE	36
Reference scan mode	GRE/separate

Resolution - Filter Image

Image Filter	Off
Distortion Corr.	On
Mode	2D
Unfiltered images	Off
Prescan Normalize	Off
Normalize	Off
B1 filter	Off

Resolution - Filter Rawdata

Raw filter	Off
Elliptical filter	Off
POCS	Off

Geometry - Common

Slice group	1
Slices	1
Dist. factor	20 %
Position	Isocenter
Orientation	Transversal
Phase enc. dir.	A >> P
FoV read	360 mm
FoV phase	85.2 %
Slice thickness	8.0 mm
TR	359.84 ms
Multi-slice mode	Sequential
Series	Base To Apex
Concatenations	1

Geometry - AutoAlign

Slice group	1
Position	Isocenter
Orientation	Transversal
Phase enc. dir.	A >> P
AutoAlign	---
Initial Position	Isocenter
Phase	0.0 mm
Read	0.0 mm
Shift	0.0 mm
Initial Rotation	0.00 deg
Initial Orientation	Transversal

Geometry - Saturation

Fat suppr.	None
Wrap-up Magn.	None
Special sat.	None

Geometry - Navigator

System - Miscellaneous

Positioning mode	FIX
Table position	H
Table position	0 mm

System - Miscellaneous

MSMA	S - C - T
Sagittal	R >> L
Coronal	A >> P
Transversal	F >> H
Coil Combine Mode	Adaptive Combine
Save uncombined	Off
Matrix Optimization	Off
Coil Focus	Flat
AutoAlign	---
Coil Select Mode	Default

System - Adjustments

B0 Shim mode	Cardiac
Adjust with body coil	Off
Confirm freq. adjustment	Off
Assume Dominant Fat	Off
Assume Silicone	Off
Adjustment Tolerance	Auto

System - Adjust Volume

! Position	Isocenter
! Orientation	Transversal
! Rotation	0.00 deg
! A >> P	150 mm
! R >> L	150 mm
! F >> H	150 mm
Reset	Off

System - Tx/Rx

Frequency 1H	63.683250 MHz
Correction factor	1
Gain	High
Img. Scale Cor.	1.000
Reset	Off
? Ref. amplitude 1H	0.000 V

Physio - Signal1

1st Signal/Mode	ECG/Trigger
Average cycle	787 ± 129 ms
Average cycle	No Signal ms
Captured cycle	787 ± 129 ms
Acquisition window	813 ms
Trigger pulse	1
Trigger delay	0 ms
TR	359.84 ms
Concatenations	1
Segments	72
Phases	1
Adaptive Triggering	Off

Physio - Cardiac

Tagging	None
Magn. preparation	Non-sel. IR T1map
TI	260 ms
Fat suppr.	None
Dark blood	Off
FoV read	360 mm
FoV phase	85.2 %
Phase resolution	66 %
Cine	Off
Trajectory	Cartesian
Dummy heartbeats	0
Motion Correction	Standard

Physio - PACE

Resp. control	Breath-hold
Concatenations	1

Sequence - Part 1

Introduction	Off
Dimension	2D
Reordering	Linear
Asymmetric echo	Weak
Contrasts	1
Optimization	Min. TE TR
Multi-slice mode	Sequential
Sequence type	Trufi
Bandwidth	1085 Hz/Px

Sequence - Part 2

Define	Shots
Shots per slice	1
Segments	72
Trufi delta freq.	0 Hz
RF pulse type	Fast
Gradient mode	Fast
Excitation	Slice-sel.
Flip angle mode	Constant
Cine	Off

Sequence - Assistant

Mode	Off
Allowed delay	0 s

\\USER\Cardiac Research Protocols\Rapi-STRESS T1\Rapi-STRESS T1 (V2)\ShMOLLI_192i_d11_nFilt (TD=0)

TA: 7.1 s PM: FIX Voxel size: 0.9×0.9×8.0 mmPAT: 2 Rel. SNR: 1.00 : tfi

Properties

Prio recon	Off
Load images to viewer	On
Inline movie	Off
Auto store images	On
Load images to stamp segments	Off
Load images to graphic segments	On
Auto open inline display	Off
Auto close inline display	Off
Start measurement without further preparation	On
Wait for user to start	Off
Start measurements	Single measurement

Routine

Slice group	1
Slices	1
Dist. factor	25 %
Position	Isocenter
Orientation	Transversal
Phase enc. dir.	A >> P
AutoAlign	---
Phase oversampling	0 %
FoV read	360 mm
FoV phase	75.0 %
Slice thickness	8.0 mm
TR	378.98 ms
TE	1.07 ms
Averages	1
Concatenations	1
Filter	Raw filter, Distortion Corr.(2D)
Coil elements	BO2;SP2,3

Contrast - Common

TR	378.98 ms
TE	1.07 ms
Magn. preparation	Non-sel. IR
TI	260 ms
Flip angle	35 deg
Fat suppr.	None
Wrap-up Magn.	None

Contrast - Dynamic

Averages	1
Averaging mode	Short term
Reconstruction	Magn./Phase
Measurements	1
Multiple series	Off

Resolution - Common

FoV read	360 mm
FoV phase	75.0 %
Slice thickness	8.0 mm
Base resolution	192
Phase resolution	100 %
Phase partial Fourier	6/8
Trajectory	Cartesian
Interpolation	On

Resolution - iPAT

PAT mode	GRAPPA
Accel. factor PE	2
Ref. lines PE	24
Reference scan mode	Integrated

Resolution - Filter Image

Image Filter	Off
Distortion Corr.	On
Mode	2D
Unfiltered images	Off
Prescan Normalize	Off
Normalize	Off
B1 filter	Off

Resolution - Filter Rawdata

Raw filter	On
Elliptical filter	Off
POCS	Off

Geometry - Common

Slice group	1
Slices	1
Dist. factor	25 %
Position	Isocenter
Orientation	Transversal
Phase enc. dir.	A >> P
FoV read	360 mm
FoV phase	75.0 %
Slice thickness	8.0 mm
TR	378.98 ms
Multi-slice mode	Sequential
Series	Interleaved
Concatenations	1

Geometry - AutoAlign

Slice group	1
Position	Isocenter
Orientation	Transversal
Phase enc. dir.	A >> P
AutoAlign	---
Initial Position	Isocenter
Phase	0.0 mm
Read	0.0 mm
Shift	0.0 mm
Initial Rotation	0.00 deg
Initial Orientation	Transversal

Geometry - Saturation

Fat suppr.	None
Wrap-up Magn.	None
Special sat.	None

Geometry - Navigator

System - Miscellaneous

Positioning mode	FIX
Table position	H
Table position	0 mm

System - Miscellaneous

MSMA	S - C - T
Sagittal	R >> L
Coronal	A >> P
Transversal	F >> H
Coil Combine Mode	Adaptive Combine
Save uncombined	Off
Matrix Optimization	Off
Coil Focus	Flat
AutoAlign	---
Coil Select Mode	Default

System - Adjustments

B0 Shim mode	Cardiac
Adjust with body coil	On
Confirm freq. adjustment	Off
Assume Dominant Fat	Off
Assume Silicone	Off
Adjustment Tolerance	Auto

System - Adjust Volume

! Position	Isocenter
! Orientation	Transversal
! Rotation	0.00 deg
! A >> P	150 mm
! R >> L	150 mm
! F >> H	150 mm
Reset	Off

System - Tx/Rx

Frequency 1H	63.683250 MHz
Correction factor	1
Gain	High
Img. Scale Cor.	1.000
Reset	Off
? Ref. amplitude 1H	0.000 V

Physio - Signal1

1st Signal/Mode	ECG/Trigger
Average cycle	787 ± 129 ms
Average cycle	No Signal ms
Captured cycle	787 ± 129 ms
Acquisition window	379 ms
Trigger pulse	1
Trigger delay	0 ms
TR	378.98 ms
Concatenations	1
Segments	84
Phases	1
Adaptive Triggering	Off

Physio - Cardiac

Tagging	None
Magn. preparation	Non-sel. IR
TI	260 ms
Fat suppr.	None
Dark blood	Off
FoV read	360 mm
FoV phase	75.0 %
Phase resolution	100 %
Cine	Off
Trajectory	Cartesian
Dummy heartbeats	0
Motion Correction	None

Physio - PACE

Resp. control	Off
Concatenations	1

Sequence - Part 1

Introduction	Off
Dimension	2D
Reordering	Linear
Asymmetric echo	Weak
Contrasts	1
Optimization	Min. TE TR
Multi-slice mode	Sequential
Sequence type	Trufi
Bandwidth	898 Hz/Px

Sequence - Part 2

Define	Shots
Shots per slice	1
Segments	84
Trufi delta freq.	0 Hz
RF pulse type	Fast
Gradient mode	Fast
Excitation	Slice-sel.
Flip angle mode	Constant
Cine	Off

Sequence - Assistant

Mode	Off
Allowed delay	0 s

\\USER\Cardiac Research Protocols\Rapi-STRESS T1\Rapi-STRESS T1 (V2)\T1Map_ShortT1(TD=0)highHR

TA: 8.7 s PM: FIX Voxel size: 1.9×1.9×8.0 mmPAT: 2 Rel. SNR: 1.00 : tfi

Properties

Prio recon	Off
Load images to viewer	On
Inline movie	Off
Auto store images	On
Load images to stamp segments	On
Load images to graphic segments	On
Auto open inline display	Off
Auto close inline display	Off
Start measurement without further preparation	On
Wait for user to start	Off
Start measurements	Single measurement

Routine

Slice group	1
Slices	1
Dist. factor	20 %
Position	Isocenter
Orientation	Transversal
Phase enc. dir.	A >> P
AutoAlign	---
Phase oversampling	0 %
FoV read	360 mm
FoV phase	85.4 %
Slice thickness	8.0 mm
TR	341.12 ms
TE	1.01 ms
Averages	1
Concatenations	1
Filter	Distortion Corr.(2D)
Coil elements	BO2;SP2,3

Contrast - Common

TR	341.12 ms
TE	1.01 ms
Magn. preparation	Non-sel. IR T1map
TI	260 ms
Flip angle	35 deg
Fat suppr.	None
Wrap-up Magn.	None

Contrast - Dynamic

Averages	1
Averaging mode	Short term
Reconstruction	Magnitude
Measurements	1
Multiple series	Off

Resolution - Common

FoV read	360 mm
FoV phase	85.4 %
Slice thickness	8.0 mm
Base resolution	192
Phase resolution	78 %
Phase partial Fourier	7/8
Trajectory	Cartesian
Interpolation	Off

Resolution - iPAT

PAT mode	GRAPPA
Accel. factor PE	2
Ref. lines PE	36
Reference scan mode	GRE/separate

Resolution - Filter Image

Image Filter	Off
Distortion Corr.	On
Mode	2D
Unfiltered images	Off
Prescan Normalize	Off
Normalize	Off
B1 filter	Off

Resolution - Filter Rawdata

Raw filter	Off
Elliptical filter	Off
POCS	Off

Geometry - Common

Slice group	1
Slices	1
Dist. factor	20 %
Position	Isocenter
Orientation	Transversal
Phase enc. dir.	A >> P
FoV read	360 mm
FoV phase	85.4 %
Slice thickness	8.0 mm
TR	341.12 ms
Multi-slice mode	Sequential
Series	Base To Apex
Concatenations	1

Geometry - AutoAlign

Slice group	1
Position	Isocenter
Orientation	Transversal
Phase enc. dir.	A >> P
AutoAlign	---
Initial Position	Isocenter
Phase	0.0 mm
Read	0.0 mm
Shift	0.0 mm
Initial Rotation	0.00 deg
Initial Orientation	Transversal

Geometry - Saturation

Fat suppr.	None
Wrap-up Magn.	None
Special sat.	None

Geometry - Navigator

System - Miscellaneous

Positioning mode	FIX
Table position	H
Table position	0 mm

System - Miscellaneous

MSMA	S - C - T
Sagittal	R >> L
Coronal	A >> P
Transversal	F >> H
Coil Combine Mode	Adaptive Combine
Save uncombined	Off
Matrix Optimization	Off
Coil Focus	Flat
AutoAlign	---
Coil Select Mode	Default

System - Adjustments

B0 Shim mode	Cardiac
Adjust with body coil	Off
Confirm freq. adjustment	Off
Assume Dominant Fat	Off
Assume Silicone	Off
Adjustment Tolerance	Auto

System - Adjust Volume

! Position	Isocenter
! Orientation	Transversal
! Rotation	0.00 deg
! A >> P	150 mm
! R >> L	150 mm
! F >> H	150 mm
Reset	Off

System - Tx/Rx

Frequency 1H	63.683250 MHz
Correction factor	1
Gain	High
Img. Scale Cor.	1.000
Reset	Off
? Ref. amplitude 1H	0.000 V

Physio - Signal1

1st Signal/Mode	ECG/Trigger
Average cycle	787 ± 129 ms
Average cycle	No Signal ms
Captured cycle	787 ± 129 ms
Acquisition window	704 ms
Trigger pulse	1
Trigger delay	0 ms
TR	341.12 ms
Concatenations	1
Segments	64
Phases	1
Adaptive Triggering	Off

Physio - Cardiac

Tagging	None
Magn. preparation	Non-sel. IR T1map
TI	260 ms
Fat suppr.	None
Dark blood	Off
FoV read	360 mm
FoV phase	85.4 %
Phase resolution	78 %
Cine	Off
Trajectory	Cartesian
Dummy heartbeats	0
Motion Correction	Standard

Physio - PACE

Resp. control	Breath-hold
Concatenations	1

Sequence - Part 1

Introduction	Off
Dimension	2D
Reordering	Linear
Asymmetric echo	Weak
Contrasts	1
Optimization	Min. TE TR
Multi-slice mode	Sequential
Sequence type	Trufi
Bandwidth	1085 Hz/Px

Sequence - Part 2

Define	Shots
Shots per slice	1
Segments	64
Trufi delta freq.	0 Hz
RF pulse type	Fast
Gradient mode	Fast
Excitation	Slice-sel.
Flip angle mode	Constant
Cine	Off

Sequence - Assistant

Mode	Off
Allowed delay	0 s

\\USER\Cardiac Research Protocols\Rapi-STRESS T1\Rapi-STRESS T1 (V2)\ShMOLLI_192i_d11_nFilt (TD=0)

TA: 7.1 s PM: FIX Voxel size: 0.9×0.9×8.0 mmPAT: 2 Rel. SNR: 1.00 : tfi

Properties

Prio recon	Off
Load images to viewer	On
Inline movie	Off
Auto store images	On
Load images to stamp segments	Off
Load images to graphic segments	On
Auto open inline display	Off
Auto close inline display	Off
Start measurement without further preparation	On
Wait for user to start	Off
Start measurements	Single measurement

Routine

Slice group	1
Slices	1
Dist. factor	25 %
Position	Isocenter
Orientation	Transversal
Phase enc. dir.	A >> P
AutoAlign	---
Phase oversampling	0 %
FoV read	360 mm
FoV phase	75.0 %
Slice thickness	8.0 mm
TR	378.98 ms
TE	1.07 ms
Averages	1
Concatenations	1
Filter	Raw filter, Distortion Corr.(2D)
Coil elements	BO2;SP2,3

Contrast - Common

TR	378.98 ms
TE	1.07 ms
Magn. preparation	Non-sel. IR
TI	260 ms
Flip angle	35 deg
Fat suppr.	None
Wrap-up Magn.	None

Contrast - Dynamic

Averages	1
Averaging mode	Short term
Reconstruction	Magn./Phase
Measurements	1
Multiple series	Off

Resolution - Common

FoV read	360 mm
FoV phase	75.0 %
Slice thickness	8.0 mm
Base resolution	192
Phase resolution	100 %
Phase partial Fourier	6/8
Trajectory	Cartesian
Interpolation	On

Resolution - iPAT

PAT mode	GRAPPA
Accel. factor PE	2
Ref. lines PE	24
Reference scan mode	Integrated

Resolution - Filter Image

Image Filter	Off
Distortion Corr.	On
Mode	2D
Unfiltered images	Off
Prescan Normalize	Off
Normalize	Off
B1 filter	Off

Resolution - Filter Rawdata

Raw filter	On
Elliptical filter	Off
POCS	Off

Geometry - Common

Slice group	1
Slices	1
Dist. factor	25 %
Position	Isocenter
Orientation	Transversal
Phase enc. dir.	A >> P
FoV read	360 mm
FoV phase	75.0 %
Slice thickness	8.0 mm
TR	378.98 ms
Multi-slice mode	Sequential
Series	Interleaved
Concatenations	1

Geometry - AutoAlign

Slice group	1
Position	Isocenter
Orientation	Transversal
Phase enc. dir.	A >> P
AutoAlign	---
Initial Position	Isocenter
Phase	0.0 mm
Read	0.0 mm
Shift	0.0 mm
Initial Rotation	0.00 deg
Initial Orientation	Transversal

Geometry - Saturation

Fat suppr.	None
Wrap-up Magn.	None
Special sat.	None

Geometry - Navigator

System - Miscellaneous

Positioning mode	FIX
Table position	H
Table position	0 mm

System - Miscellaneous

MSMA	S - C - T
Sagittal	R >> L
Coronal	A >> P
Transversal	F >> H
Coil Combine Mode	Adaptive Combine
Save uncombined	Off
Matrix Optimization	Off
Coil Focus	Flat
AutoAlign	---
Coil Select Mode	Default

System - Adjustments

B0 Shim mode	Cardiac
Adjust with body coil	On
Confirm freq. adjustment	Off
Assume Dominant Fat	Off
Assume Silicone	Off
Adjustment Tolerance	Auto

System - Adjust Volume

! Position	Isocenter
! Orientation	Transversal
! Rotation	0.00 deg
! A >> P	150 mm
! R >> L	150 mm
! F >> H	150 mm
Reset	Off

System - Tx/Rx

Frequency 1H	63.683250 MHz
Correction factor	1
Gain	High
Img. Scale Cor.	1.000
Reset	Off
? Ref. amplitude 1H	0.000 V

Physio - Signal1

1st Signal/Mode	ECG/Trigger
Average cycle	787 ± 129 ms
Average cycle	No Signal ms
Captured cycle	787 ± 129 ms
Acquisition window	379 ms
Trigger pulse	1
Trigger delay	0 ms
TR	378.98 ms
Concatenations	1
Segments	84
Phases	1
Adaptive Triggering	Off

Physio - Cardiac

Tagging	None
Magn. preparation	Non-sel. IR
TI	260 ms
Fat suppr.	None
Dark blood	Off
FoV read	360 mm
FoV phase	75.0 %
Phase resolution	100 %
Cine	Off
Trajectory	Cartesian
Dummy heartbeats	0
Motion Correction	None

Physio - PACE

Resp. control	Off
Concatenations	1

Sequence - Part 1

Introduction	Off
Dimension	2D
Reordering	Linear
Asymmetric echo	Weak
Contrasts	1
Optimization	Min. TE TR
Multi-slice mode	Sequential
Sequence type	Trufi
Bandwidth	898 Hz/Px

Sequence - Part 2

Define	Shots
Shots per slice	1
Segments	84
Trufi delta freq.	0 Hz
RF pulse type	Fast
Gradient mode	Fast
Excitation	Slice-sel.
Flip angle mode	Constant
Cine	Off

Sequence - Assistant

Mode	Off
Allowed delay	0 s

\\USER\Cardiac Research Protocols\Rapi-STRESS T1\Rapi-STRESS T1 (V2)\MidShMOLLI_192i_d11_nFilt (TD=0)

TA: 7.1 s PM: REF Voxel size: 0.9×0.9×8.0 mmPAT: 2 Rel. SNR: 1.00 : tfi

Properties

Prio recon	Off
Load images to viewer	On
Inline movie	Off
Auto store images	On
Load images to stamp segments	Off
Load images to graphic segments	On
Auto open inline display	Off
Auto close inline display	Off
Start measurement without further preparation	Off
Wait for user to start	Off
Start measurements	Single measurement

Routine

Slice group	1
Slices	1
Dist. factor	25 %
Position	Isocenter
Orientation	Transversal
Phase enc. dir.	A >> P
AutoAlign	---
Phase oversampling	0 %
FoV read	360 mm
FoV phase	75.0 %
Slice thickness	8.0 mm
TR	378.98 ms
TE	1.07 ms
Averages	1
Concatenations	1
Filter	Raw filter, Distortion Corr.(2D)
Coil elements	BO2;SP2,3

Contrast - Common

TR	378.98 ms
TE	1.07 ms
Magn. preparation	Non-sel. IR
TI	260 ms
Flip angle	35 deg
Fat suppr.	None
Wrap-up Magn.	None

Contrast - Dynamic

Averages	1
Averaging mode	Short term
Reconstruction	Magn./Phase
Measurements	1
Multiple series	Off

Resolution - Common

FoV read	360 mm
FoV phase	75.0 %
Slice thickness	8.0 mm
Base resolution	192
Phase resolution	100 %
Phase partial Fourier	6/8
Trajectory	Cartesian
Interpolation	On

Resolution - iPAT

PAT mode	GRAPPA
Accel. factor PE	2
Ref. lines PE	24
Reference scan mode	Integrated

Resolution - Filter Image

Image Filter	Off
Distortion Corr.	On
Mode	2D
Unfiltered images	Off
Prescan Normalize	Off
Normalize	Off
B1 filter	Off

Resolution - Filter Rawdata

Raw filter	On
Elliptical filter	Off
POCS	Off

Geometry - Common

Slice group	1
Slices	1
Dist. factor	25 %
Position	Isocenter
Orientation	Transversal
Phase enc. dir.	A >> P
FoV read	360 mm
FoV phase	75.0 %
Slice thickness	8.0 mm
TR	378.98 ms
Multi-slice mode	Sequential
Series	Interleaved
Concatenations	1

Geometry - AutoAlign

Slice group	1
Position	Isocenter
Orientation	Transversal
Phase enc. dir.	A >> P
AutoAlign	---
Initial Position	Isocenter
Phase	0.0 mm
Read	0.0 mm
Shift	0.0 mm
Initial Rotation	0.00 deg
Initial Orientation	Transversal

Geometry - Saturation

Fat suppr.	None
Wrap-up Magn.	None
Special sat.	None

Geometry - Navigator

System - Miscellaneous

Positioning mode	REF
Table position	H
Table position	0 mm

System - Miscellaneous

MSMA	S - C - T
Sagittal	R >> L
Coronal	A >> P
Transversal	F >> H
Coil Combine Mode	Adaptive Combine
Save uncombined	Off
Matrix Optimization	Off
Coil Focus	Flat
AutoAlign	---
Coil Select Mode	Default

System - Adjustments

B0 Shim mode	Cardiac
Adjust with body coil	On
Confirm freq. adjustment	Off
Assume Dominant Fat	Off
Assume Silicone	Off
Adjustment Tolerance	Auto

System - Adjust Volume

! Position	Isocenter
! Orientation	Transversal
! Rotation	0.00 deg
! A >> P	150 mm
! R >> L	150 mm
! F >> H	150 mm
Reset	Off

System - Tx/Rx

Frequency 1H	63.683250 MHz
Correction factor	1
Gain	High
Img. Scale Cor.	1.000
Reset	Off
? Ref. amplitude 1H	0.000 V

Physio - Signal1

1st Signal/Mode	ECG/Trigger
Average cycle	787 ± 129 ms
Average cycle	No Signal ms
Captured cycle	787 ± 129 ms
Acquisition window	379 ms
Trigger pulse	1
Trigger delay	0 ms
TR	378.98 ms
Concatenations	1
Segments	84
Phases	1
Adaptive Triggering	Off

Physio - Cardiac

Tagging	None
Magn. preparation	Non-sel. IR
TI	260 ms
Fat suppr.	None
Dark blood	Off
FoV read	360 mm
FoV phase	75.0 %
Phase resolution	100 %
Cine	Off
Trajectory	Cartesian
Dummy heartbeats	0
Motion Correction	None

Physio - PACE

Resp. control	Off
Concatenations	1

Sequence - Part 1

Introduction	Off
Dimension	2D
Reordering	Linear
Asymmetric echo	Weak
Contrasts	1
Optimization	Min. TE TR
Multi-slice mode	Sequential
Sequence type	Trufi
Bandwidth	898 Hz/Px

Sequence - Part 2

Define	Shots
Shots per slice	1
Segments	84
Trufi delta freq.	0 Hz
RF pulse type	Fast
Gradient mode	Fast
Excitation	Slice-sel.
Flip angle mode	Constant
Cine	Off

Sequence - Assistant

Mode	Off
Allowed delay	0 s

\\USER\Cardiac Research Protocols\Rapi-STRESS T1\Rapi-STRESS T1 (V2)\T1Map_ShortT1(TD=0)lowHR

TA: 0:17 PM: FIX Voxel size: 1.4×1.4×8.0 mmPAT: 2 Rel. SNR: 1.00 : tfi

Properties

Prio recon	Off
Load images to viewer	On
Inline movie	Off
Auto store images	On
Load images to stamp segments	On
Load images to graphic segments	On
Auto open inline display	Off
Auto close inline display	Off
Start measurement without further preparation	On
Wait for user to start	Off
Start measurements	Single measurement

Routine

Slice group	1
Slices	1
Dist. factor	20 %
Position	Isocenter
Orientation	Transversal
Phase enc. dir.	A >> P
AutoAlign	---
Phase oversampling	0 %
FoV read	360 mm
FoV phase	85.2 %
Slice thickness	8.0 mm
TR	359.84 ms
TE	1.13 ms
Averages	1
Concatenations	1
Filter	Distortion Corr.(2D)
Coil elements	BO2;SP2,3

Contrast - Common

TR	359.84 ms
TE	1.13 ms
Magn. preparation	Non-sel. IR T1map
TI	260 ms
Flip angle	35 deg
Fat suppr.	None
Wrap-up Magn.	None

Contrast - Dynamic

Averages	1
Averaging mode	Short term
Reconstruction	Magnitude
Measurements	1
Multiple series	Off

Resolution - Common

FoV read	360 mm
FoV phase	85.2 %
Slice thickness	8.0 mm
Base resolution	256
Phase resolution	66 %
Phase partial Fourier	7/8
Trajectory	Cartesian
Interpolation	Off

Resolution - iPAT

PAT mode	GRAPPA
Accel. factor PE	2
Ref. lines PE	36
Reference scan mode	GRE/separate

Resolution - Filter Image

Image Filter	Off
Distortion Corr.	On
Mode	2D
Unfiltered images	Off
Prescan Normalize	Off
Normalize	Off
B1 filter	Off

Resolution - Filter Rawdata

Raw filter	Off
Elliptical filter	Off
POCS	Off

Geometry - Common

Slice group	1
Slices	1
Dist. factor	20 %
Position	Isocenter
Orientation	Transversal
Phase enc. dir.	A >> P
FoV read	360 mm
FoV phase	85.2 %
Slice thickness	8.0 mm
TR	359.84 ms
Multi-slice mode	Sequential
Series	Base To Apex
Concatenations	1

Geometry - AutoAlign

Slice group	1
Position	Isocenter
Orientation	Transversal
Phase enc. dir.	A >> P
AutoAlign	---
Initial Position	Isocenter
Phase	0.0 mm
Read	0.0 mm
Shift	0.0 mm
Initial Rotation	0.00 deg
Initial Orientation	Transversal

Geometry - Saturation

Fat suppr.	None
Wrap-up Magn.	None
Special sat.	None

Geometry - Navigator

System - Miscellaneous

Positioning mode	FIX
Table position	H
Table position	0 mm

System - Miscellaneous

MSMA	S - C - T
Sagittal	R >> L
Coronal	A >> P
Transversal	F >> H
Coil Combine Mode	Adaptive Combine
Save uncombined	Off
Matrix Optimization	Off
Coil Focus	Flat
AutoAlign	---
Coil Select Mode	Default

System - Adjustments

B0 Shim mode	Cardiac
Adjust with body coil	Off
Confirm freq. adjustment	Off
Assume Dominant Fat	Off
Assume Silicone	Off
Adjustment Tolerance	Auto

System - Adjust Volume

! Position	Isocenter
! Orientation	Transversal
! Rotation	0.00 deg
! A >> P	150 mm
! R >> L	150 mm
! F >> H	150 mm
Reset	Off

System - Tx/Rx

Frequency 1H	63.683250 MHz
Correction factor	1
Gain	High
Img. Scale Cor.	1.000
Reset	Off
? Ref. amplitude 1H	0.000 V

Physio - Signal1

1st Signal/Mode	ECG/Trigger
Average cycle	787 ± 129 ms
Average cycle	No Signal ms
Captured cycle	787 ± 129 ms
Acquisition window	813 ms
Trigger pulse	1
Trigger delay	0 ms
TR	359.84 ms
Concatenations	1
Segments	72
Phases	1
Adaptive Triggering	Off

Physio - Cardiac

Tagging	None
Magn. preparation	Non-sel. IR T1map
TI	260 ms
Fat suppr.	None
Dark blood	Off
FoV read	360 mm
FoV phase	85.2 %
Phase resolution	66 %
Cine	Off
Trajectory	Cartesian
Dummy heartbeats	0
Motion Correction	Standard

Physio - PACE

Resp. control	Breath-hold
Concatenations	1

Sequence - Part 1

Introduction	Off
Dimension	2D
Reordering	Linear
Asymmetric echo	Weak
Contrasts	1
Optimization	Min. TE TR
Multi-slice mode	Sequential
Sequence type	Trufi
Bandwidth	1085 Hz/Px

Sequence - Part 2

Define	Shots
Shots per slice	1
Segments	72
Trufi delta freq.	0 Hz
RF pulse type	Fast
Gradient mode	Fast
Excitation	Slice-sel.
Flip angle mode	Constant
Cine	Off

Sequence - Assistant

Mode	Off
Allowed delay	0 s

\\USER\Cardiac Research Protocols\Rapi-STRESS T1\Rapi-STRESS T1 (V2)\ShMOLLI_192i_d11_nFilt (TD=0)

TA: 7.1 s PM: FIX Voxel size: 0.9×0.9×8.0 mmPAT: 2 Rel. SNR: 1.00 : tfi

Properties

Prio recon	Off
Load images to viewer	On
Inline movie	Off
Auto store images	On
Load images to stamp segments	Off
Load images to graphic segments	On
Auto open inline display	Off
Auto close inline display	Off
Start measurement without further preparation	On
Wait for user to start	Off
Start measurements	Single measurement

Routine

Slice group	1
Slices	1
Dist. factor	25 %
Position	Isocenter
Orientation	Transversal
Phase enc. dir.	A >> P
AutoAlign	---
Phase oversampling	0 %
FoV read	360 mm
FoV phase	75.0 %
Slice thickness	8.0 mm
TR	378.98 ms
TE	1.07 ms
Averages	1
Concatenations	1
Filter	Raw filter, Distortion Corr.(2D)
Coil elements	BO2;SP2,3

Contrast - Common

TR	378.98 ms
TE	1.07 ms
Magn. preparation	Non-sel. IR
TI	260 ms
Flip angle	35 deg
Fat suppr.	None
Wrap-up Magn.	None

Contrast - Dynamic

Averages	1
Averaging mode	Short term
Reconstruction	Magn./Phase
Measurements	1
Multiple series	Off

Resolution - Common

FoV read	360 mm
FoV phase	75.0 %
Slice thickness	8.0 mm
Base resolution	192
Phase resolution	100 %
Phase partial Fourier	6/8
Trajectory	Cartesian
Interpolation	On

Resolution - iPAT

PAT mode	GRAPPA
Accel. factor PE	2
Ref. lines PE	24
Reference scan mode	Integrated

Resolution - Filter Image

Image Filter	Off
Distortion Corr.	On
Mode	2D
Unfiltered images	Off
Prescan Normalize	Off
Normalize	Off
B1 filter	Off

Resolution - Filter Rawdata

Raw filter	On
Elliptical filter	Off
POCS	Off

Geometry - Common

Slice group	1
Slices	1
Dist. factor	25 %
Position	Isocenter
Orientation	Transversal
Phase enc. dir.	A >> P
FoV read	360 mm
FoV phase	75.0 %
Slice thickness	8.0 mm
TR	378.98 ms
Multi-slice mode	Sequential
Series	Interleaved
Concatenations	1

Geometry - AutoAlign

Slice group	1
Position	Isocenter
Orientation	Transversal
Phase enc. dir.	A >> P
AutoAlign	---
Initial Position	Isocenter
Phase	0.0 mm
Read	0.0 mm
Shift	0.0 mm
Initial Rotation	0.00 deg
Initial Orientation	Transversal

Geometry - Saturation

Fat suppr.	None
Wrap-up Magn.	None
Special sat.	None

Geometry - Navigator

System - Miscellaneous

Positioning mode	FIX
Table position	H
Table position	0 mm

System - Miscellaneous

MSMA	S - C - T
Sagittal	R >> L
Coronal	A >> P
Transversal	F >> H
Coil Combine Mode	Adaptive Combine
Save uncombined	Off
Matrix Optimization	Off
Coil Focus	Flat
AutoAlign	---
Coil Select Mode	Default

System - Adjustments

B0 Shim mode	Cardiac
Adjust with body coil	On
Confirm freq. adjustment	Off
Assume Dominant Fat	Off
Assume Silicone	Off
Adjustment Tolerance	Auto

System - Adjust Volume

! Position	Isocenter
! Orientation	Transversal
! Rotation	0.00 deg
! A >> P	150 mm
! R >> L	150 mm
! F >> H	150 mm
Reset	Off

System - Tx/Rx

Frequency 1H	63.683250 MHz
Correction factor	1
Gain	High
Img. Scale Cor.	1.000
Reset	Off
? Ref. amplitude 1H	0.000 V

Physio - Signal1

1st Signal/Mode	ECG/Trigger
Average cycle	787 ± 129 ms
Average cycle	No Signal ms
Captured cycle	787 ± 129 ms
Acquisition window	379 ms
Trigger pulse	1
Trigger delay	0 ms
TR	378.98 ms
Concatenations	1
Segments	84
Phases	1
Adaptive Triggering	Off

Physio - Cardiac

Tagging	None
Magn. preparation	Non-sel. IR
TI	260 ms
Fat suppr.	None
Dark blood	Off
FoV read	360 mm
FoV phase	75.0 %
Phase resolution	100 %
Cine	Off
Trajectory	Cartesian
Dummy heartbeats	0
Motion Correction	None

Physio - PACE

Resp. control	Off
Concatenations	1

Sequence - Part 1

Introduction	Off
Dimension	2D
Reordering	Linear
Asymmetric echo	Weak
Contrasts	1
Optimization	Min. TE TR
Multi-slice mode	Sequential
Sequence type	Trufi
Bandwidth	898 Hz/Px

Sequence - Part 2

Define	Shots
Shots per slice	1
Segments	84
Trufi delta freq.	0 Hz
RF pulse type	Fast
Gradient mode	Fast
Excitation	Slice-sel.
Flip angle mode	Constant
Cine	Off

Sequence - Assistant

Mode	Off
Allowed delay	0 s

\\USER\Cardiac Research Protocols\Rapi-STRESS T1\Rapi-STRESS T1 (V2)\T1Map_ShortT1(TD=0)highHR

TA: 8.7 s PM: FIX Voxel size: 1.9×1.9×8.0 mmPAT: 2 Rel. SNR: 1.00 : tfi

Properties

Prio recon	Off
Load images to viewer	On
Inline movie	Off
Auto store images	On
Load images to stamp segments	On
Load images to graphic segments	On
Auto open inline display	Off
Auto close inline display	Off
Start measurement without further preparation	On
Wait for user to start	Off
Start measurements	Single measurement

Routine

Slice group	1
Slices	1
Dist. factor	20 %
Position	Isocenter
Orientation	Transversal
Phase enc. dir.	A >> P
AutoAlign	---
Phase oversampling	0 %
FoV read	360 mm
FoV phase	85.4 %
Slice thickness	8.0 mm
TR	341.12 ms
TE	1.01 ms
Averages	1
Concatenations	1
Filter	Distortion Corr.(2D)
Coil elements	BO2;SP2,3

Contrast - Common

TR	341.12 ms
TE	1.01 ms
Magn. preparation	Non-sel. IR T1map
TI	260 ms
Flip angle	35 deg
Fat suppr.	None
Wrap-up Magn.	None

Contrast - Dynamic

Averages	1
Averaging mode	Short term
Reconstruction	Magnitude
Measurements	1
Multiple series	Off

Resolution - Common

FoV read	360 mm
FoV phase	85.4 %
Slice thickness	8.0 mm
Base resolution	192
Phase resolution	78 %
Phase partial Fourier	7/8
Trajectory	Cartesian
Interpolation	Off

Resolution - iPAT

PAT mode	GRAPPA
Accel. factor PE	2
Ref. lines PE	36
Reference scan mode	GRE/separate

Resolution - Filter Image

Image Filter	Off
Distortion Corr.	On
Mode	2D
Unfiltered images	Off
Prescan Normalize	Off
Normalize	Off
B1 filter	Off

Resolution - Filter Rawdata

Raw filter	Off
Elliptical filter	Off
POCS	Off

Geometry - Common

Slice group	1
Slices	1
Dist. factor	20 %
Position	Isocenter
Orientation	Transversal
Phase enc. dir.	A >> P
FoV read	360 mm
FoV phase	85.4 %
Slice thickness	8.0 mm
TR	341.12 ms
Multi-slice mode	Sequential
Series	Base To Apex
Concatenations	1

Geometry - AutoAlign

Slice group	1
Position	Isocenter
Orientation	Transversal
Phase enc. dir.	A >> P
AutoAlign	---
Initial Position	Isocenter
Phase	0.0 mm
Read	0.0 mm
Shift	0.0 mm
Initial Rotation	0.00 deg
Initial Orientation	Transversal

Geometry - Saturation

Fat suppr.	None
Wrap-up Magn.	None
Special sat.	None

Geometry - Navigator

System - Miscellaneous

Positioning mode	FIX
Table position	H
Table position	0 mm

System - Miscellaneous

MSMA	S - C - T
Sagittal	R >> L
Coronal	A >> P
Transversal	F >> H
Coil Combine Mode	Adaptive Combine
Save uncombined	Off
Matrix Optimization	Off
Coil Focus	Flat
AutoAlign	---
Coil Select Mode	Default

System - Adjustments

B0 Shim mode	Cardiac
Adjust with body coil	Off
Confirm freq. adjustment	Off
Assume Dominant Fat	Off
Assume Silicone	Off
Adjustment Tolerance	Auto

System - Adjust Volume

! Position	Isocenter
! Orientation	Transversal
! Rotation	0.00 deg
! A >> P	150 mm
! R >> L	150 mm
! F >> H	150 mm
Reset	Off

System - Tx/Rx

Frequency 1H	63.683250 MHz
Correction factor	1
Gain	High
Img. Scale Cor.	1.000
Reset	Off
? Ref. amplitude 1H	0.000 V

Physio - Signal1

1st Signal/Mode	ECG/Trigger
Average cycle	787 ± 129 ms
Average cycle	No Signal ms
Captured cycle	787 ± 129 ms
Acquisition window	704 ms
Trigger pulse	1
Trigger delay	0 ms
TR	341.12 ms
Concatenations	1
Segments	64
Phases	1
Adaptive Triggering	Off

Physio - Cardiac

Tagging	None
Magn. preparation	Non-sel. IR T1map
TI	260 ms
Fat suppr.	None
Dark blood	Off
FoV read	360 mm
FoV phase	85.4 %
Phase resolution	78 %
Cine	Off
Trajectory	Cartesian
Dummy heartbeats	0
Motion Correction	Standard

Physio - PACE

Resp. control	Breath-hold
Concatenations	1

Sequence - Part 1

Introduction	Off
Dimension	2D
Reordering	Linear
Asymmetric echo	Weak
Contrasts	1
Optimization	Min. TE TR
Multi-slice mode	Sequential
Sequence type	Trufi
Bandwidth	1085 Hz/Px

Sequence - Part 2

Define	Shots
Shots per slice	1
Segments	64
Trufi delta freq.	0 Hz
RF pulse type	Fast
Gradient mode	Fast
Excitation	Slice-sel.
Flip angle mode	Constant
Cine	Off

Sequence - Assistant

Mode	Off
Allowed delay	0 s

\\USER\Cardiac Research Protocols\Rapi-STRESS T1\Rapi-STRESS T1 (V2)\ShMOLLI_192i_d11_nFilt (TD=0)

TA: 7.1 s PM: FIX Voxel size: 0.9×0.9×8.0 mmPAT: 2 Rel. SNR: 1.00 : tfi

Properties

Prio recon	Off
Load images to viewer	On
Inline movie	Off
Auto store images	On
Load images to stamp segments	Off
Load images to graphic segments	On
Auto open inline display	Off
Auto close inline display	Off
Start measurement without further preparation	On
Wait for user to start	Off
Start measurements	Single measurement

Routine

Slice group	1
Slices	1
Dist. factor	25 %
Position	Isocenter
Orientation	Transversal
Phase enc. dir.	A >> P
AutoAlign	---
Phase oversampling	0 %
FoV read	360 mm
FoV phase	75.0 %
Slice thickness	8.0 mm
TR	378.98 ms
TE	1.07 ms
Averages	1
Concatenations	1
Filter	Raw filter, Distortion Corr.(2D)
Coil elements	BO2;SP2,3

Contrast - Common

TR	378.98 ms
TE	1.07 ms
Magn. preparation	Non-sel. IR
TI	260 ms
Flip angle	35 deg
Fat suppr.	None
Wrap-up Magn.	None

Contrast - Dynamic

Averages	1
Averaging mode	Short term
Reconstruction	Magn./Phase
Measurements	1
Multiple series	Off

Resolution - Common

FoV read	360 mm
FoV phase	75.0 %
Slice thickness	8.0 mm
Base resolution	192
Phase resolution	100 %
Phase partial Fourier	6/8
Trajectory	Cartesian
Interpolation	On

Resolution - iPAT

PAT mode	GRAPPA
Accel. factor PE	2
Ref. lines PE	24
Reference scan mode	Integrated

Resolution - Filter Image

Image Filter	Off
Distortion Corr.	On
Mode	2D
Unfiltered images	Off
Prescan Normalize	Off
Normalize	Off
B1 filter	Off

Resolution - Filter Rawdata

Raw filter	On
Elliptical filter	Off
POCS	Off

Geometry - Common

Slice group	1
Slices	1
Dist. factor	25 %
Position	Isocenter
Orientation	Transversal
Phase enc. dir.	A >> P
FoV read	360 mm
FoV phase	75.0 %
Slice thickness	8.0 mm
TR	378.98 ms
Multi-slice mode	Sequential
Series	Interleaved
Concatenations	1

Geometry - AutoAlign

Slice group	1
Position	Isocenter
Orientation	Transversal
Phase enc. dir.	A >> P
AutoAlign	---
Initial Position	Isocenter
Phase	0.0 mm
Read	0.0 mm
Shift	0.0 mm
Initial Rotation	0.00 deg
Initial Orientation	Transversal

Geometry - Saturation

Fat suppr.	None
Wrap-up Magn.	None
Special sat.	None

Geometry - Navigator

System - Miscellaneous

Positioning mode	FIX
Table position	H
Table position	0 mm

System - Miscellaneous

MSMA	S - C - T
Sagittal	R >> L
Coronal	A >> P
Transversal	F >> H
Coil Combine Mode	Adaptive Combine
Save uncombined	Off
Matrix Optimization	Off
Coil Focus	Flat
AutoAlign	---
Coil Select Mode	Default

System - Adjustments

B0 Shim mode	Cardiac
Adjust with body coil	On
Confirm freq. adjustment	Off
Assume Dominant Fat	Off
Assume Silicone	Off
Adjustment Tolerance	Auto

System - Adjust Volume

! Position	Isocenter
! Orientation	Transversal
! Rotation	0.00 deg
! A >> P	150 mm
! R >> L	150 mm
! F >> H	150 mm
Reset	Off

System - Tx/Rx

Frequency 1H	63.683250 MHz
Correction factor	1
Gain	High
Img. Scale Cor.	1.000
Reset	Off
? Ref. amplitude 1H	0.000 V

Physio - Signal1

1st Signal/Mode	ECG/Trigger
Average cycle	787 ± 129 ms
Average cycle	No Signal ms
Captured cycle	787 ± 129 ms
Acquisition window	379 ms
Trigger pulse	1
Trigger delay	0 ms
TR	378.98 ms
Concatenations	1
Segments	84
Phases	1
Adaptive Triggering	Off

Physio - Cardiac

Tagging	None
Magn. preparation	Non-sel. IR
TI	260 ms
Fat suppr.	None
Dark blood	Off
FoV read	360 mm
FoV phase	75.0 %
Phase resolution	100 %
Cine	Off
Trajectory	Cartesian
Dummy heartbeats	0
Motion Correction	None

Physio - PACE

Resp. control	Off
Concatenations	1

Sequence - Part 1

Introduction	Off
Dimension	2D
Reordering	Linear
Asymmetric echo	Weak
Contrasts	1
Optimization	Min. TE TR
Multi-slice mode	Sequential
Sequence type	Trufi
Bandwidth	898 Hz/Px

Sequence - Part 2

Define	Shots
Shots per slice	1
Segments	84
Trufi delta freq.	0 Hz
RF pulse type	Fast
Gradient mode	Fast
Excitation	Slice-sel.
Flip angle mode	Constant
Cine	Off

Sequence - Assistant

Mode	Off
Allowed delay	0 s

\\USER\Cardiac Research Protocols\Rapi-STRESS T1\Rapi-STRESS T1 (V2)\MidShMOLLI_192i_d11_nFilt (TD=0)

TA: 7.1 s PM: REF Voxel size: 0.9×0.9×8.0 mmPAT: 2 Rel. SNR: 1.00 : tfi

Properties

Prio recon	Off
Load images to viewer	On
Inline movie	Off
Auto store images	On
Load images to stamp segments	Off
Load images to graphic segments	On
Auto open inline display	Off
Auto close inline display	Off
Start measurement without further preparation	Off
Wait for user to start	Off
Start measurements	Single measurement

Routine

Slice group	1
Slices	1
Dist. factor	25 %
Position	Isocenter
Orientation	Transversal
Phase enc. dir.	A >> P
AutoAlign	---
Phase oversampling	0 %
FoV read	360 mm
FoV phase	75.0 %
Slice thickness	8.0 mm
TR	378.98 ms
TE	1.07 ms
Averages	1
Concatenations	1
Filter	Raw filter, Distortion Corr.(2D)
Coil elements	BO2;SP2,3

Contrast - Common

TR	378.98 ms
TE	1.07 ms
Magn. preparation	Non-sel. IR
TI	260 ms
Flip angle	35 deg
Fat suppr.	None
Wrap-up Magn.	None

Contrast - Dynamic

Averages	1
Averaging mode	Short term
Reconstruction	Magn./Phase
Measurements	1
Multiple series	Off

Resolution - Common

FoV read	360 mm
FoV phase	75.0 %
Slice thickness	8.0 mm
Base resolution	192
Phase resolution	100 %
Phase partial Fourier	6/8
Trajectory	Cartesian
Interpolation	On

Resolution - iPAT

PAT mode	GRAPPA
Accel. factor PE	2
Ref. lines PE	24
Reference scan mode	Integrated

Resolution - Filter Image

Image Filter	Off
Distortion Corr.	On
Mode	2D
Unfiltered images	Off
Prescan Normalize	Off
Normalize	Off
B1 filter	Off

Resolution - Filter Rawdata

Raw filter	On
Elliptical filter	Off
POCS	Off

Geometry - Common

Slice group	1
Slices	1
Dist. factor	25 %
Position	Isocenter
Orientation	Transversal
Phase enc. dir.	A >> P
FoV read	360 mm
FoV phase	75.0 %
Slice thickness	8.0 mm
TR	378.98 ms
Multi-slice mode	Sequential
Series	Interleaved
Concatenations	1

Geometry - AutoAlign

Slice group	1
Position	Isocenter
Orientation	Transversal
Phase enc. dir.	A >> P
AutoAlign	---
Initial Position	Isocenter
Phase	0.0 mm
Read	0.0 mm
Shift	0.0 mm
Initial Rotation	0.00 deg
Initial Orientation	Transversal

Geometry - Saturation

Fat suppr.	None
Wrap-up Magn.	None
Special sat.	None

Geometry - Navigator

System - Miscellaneous

Positioning mode	REF
Table position	H
Table position	0 mm

System - Miscellaneous

MSMA	S - C - T
Sagittal	R >> L
Coronal	A >> P
Transversal	F >> H
Coil Combine Mode	Adaptive Combine
Save uncombined	Off
Matrix Optimization	Off
Coil Focus	Flat
AutoAlign	---
Coil Select Mode	Default

System - Adjustments

B0 Shim mode	Cardiac
Adjust with body coil	On
Confirm freq. adjustment	Off
Assume Dominant Fat	Off
Assume Silicone	Off
Adjustment Tolerance	Auto

System - Adjust Volume

! Position	Isocenter
! Orientation	Transversal
! Rotation	0.00 deg
! A >> P	150 mm
! R >> L	150 mm
! F >> H	150 mm
Reset	Off

System - Tx/Rx

Frequency 1H	63.683250 MHz
Correction factor	1
Gain	High
Img. Scale Cor.	1.000
Reset	Off
? Ref. amplitude 1H	0.000 V

Physio - Signal1

1st Signal/Mode	ECG/Trigger
Average cycle	787 ± 129 ms
Average cycle	No Signal ms
Captured cycle	787 ± 129 ms
Acquisition window	379 ms
Trigger pulse	1
Trigger delay	0 ms
TR	378.98 ms
Concatenations	1
Segments	84
Phases	1
Adaptive Triggering	Off

Physio - Cardiac

Tagging	None
Magn. preparation	Non-sel. IR
TI	260 ms
Fat suppr.	None
Dark blood	Off
FoV read	360 mm
FoV phase	75.0 %
Phase resolution	100 %
Cine	Off
Trajectory	Cartesian
Dummy heartbeats	0
Motion Correction	None

Physio - PACE

Resp. control	Off
Concatenations	1

Sequence - Part 1

Introduction	Off
Dimension	2D
Reordering	Linear
Asymmetric echo	Weak
Contrasts	1
Optimization	Min. TE TR
Multi-slice mode	Sequential
Sequence type	Trufi
Bandwidth	898 Hz/Px

Sequence - Part 2

Define	Shots
Shots per slice	1
Segments	84
Trufi delta freq.	0 Hz
RF pulse type	Fast
Gradient mode	Fast
Excitation	Slice-sel.
Flip angle mode	Constant
Cine	Off

Sequence - Assistant

Mode	Off
Allowed delay	0 s

\\USER\Cardiac Research Protocols\Rapi-STRESS T1\Rapi-STRESS T1 (V2)\T1Map_ShortT1(TD=0)lowHR

TA: 0:17 PM: FIX Voxel size: 1.4×1.4×8.0 mmPAT: 2 Rel. SNR: 1.00 : tfi

Properties

Prio recon	Off
Load images to viewer	On
Inline movie	Off
Auto store images	On
Load images to stamp segments	On
Load images to graphic segments	On
Auto open inline display	Off
Auto close inline display	Off
Start measurement without further preparation	On
Wait for user to start	Off
Start measurements	Single measurement

Routine

Slice group	1
Slices	1
Dist. factor	20 %
Position	Isocenter
Orientation	Transversal
Phase enc. dir.	A >> P
AutoAlign	---
Phase oversampling	0 %
FoV read	360 mm
FoV phase	85.2 %
Slice thickness	8.0 mm
TR	359.84 ms
TE	1.13 ms
Averages	1
Concatenations	1
Filter	Distortion Corr.(2D)
Coil elements	BO2;SP2,3

Contrast - Common

TR	359.84 ms
TE	1.13 ms
Magn. preparation	Non-sel. IR T1map
TI	260 ms
Flip angle	35 deg
Fat suppr.	None
Wrap-up Magn.	None

Contrast - Dynamic

Averages	1
Averaging mode	Short term
Reconstruction	Magnitude
Measurements	1
Multiple series	Off

Resolution - Common

FoV read	360 mm
FoV phase	85.2 %
Slice thickness	8.0 mm
Base resolution	256
Phase resolution	66 %
Phase partial Fourier	7/8
Trajectory	Cartesian
Interpolation	Off

Resolution - iPAT

PAT mode	GRAPPA
Accel. factor PE	2
Ref. lines PE	36
Reference scan mode	GRE/separate

Resolution - Filter Image

Image Filter	Off
Distortion Corr.	On
Mode	2D
Unfiltered images	Off
Prescan Normalize	Off
Normalize	Off
B1 filter	Off

Resolution - Filter Rawdata

Raw filter	Off
Elliptical filter	Off
POCS	Off

Geometry - Common

Slice group	1
Slices	1
Dist. factor	20 %
Position	Isocenter
Orientation	Transversal
Phase enc. dir.	A >> P
FoV read	360 mm
FoV phase	85.2 %
Slice thickness	8.0 mm
TR	359.84 ms
Multi-slice mode	Sequential
Series	Base To Apex
Concatenations	1

Geometry - AutoAlign

Slice group	1
Position	Isocenter
Orientation	Transversal
Phase enc. dir.	A >> P
AutoAlign	---
Initial Position	Isocenter
Phase	0.0 mm
Read	0.0 mm
Shift	0.0 mm
Initial Rotation	0.00 deg
Initial Orientation	Transversal

Geometry - Saturation

Fat suppr.	None
Wrap-up Magn.	None
Special sat.	None

Geometry - Navigator

System - Miscellaneous

Positioning mode	FIX
Table position	H
Table position	0 mm

System - Miscellaneous

MSMA	S - C - T
Sagittal	R >> L
Coronal	A >> P
Transversal	F >> H
Coil Combine Mode	Adaptive Combine
Save uncombined	Off
Matrix Optimization	Off
Coil Focus	Flat
AutoAlign	---
Coil Select Mode	Default

System - Adjustments

B0 Shim mode	Cardiac
Adjust with body coil	Off
Confirm freq. adjustment	Off
Assume Dominant Fat	Off
Assume Silicone	Off
Adjustment Tolerance	Auto

System - Adjust Volume

! Position	Isocenter
! Orientation	Transversal
! Rotation	0.00 deg
! A >> P	150 mm
! R >> L	150 mm
! F >> H	150 mm
Reset	Off

System - Tx/Rx

Frequency 1H	63.683250 MHz
Correction factor	1
Gain	High
Img. Scale Cor.	1.000
Reset	Off
? Ref. amplitude 1H	0.000 V

Physio - Signal1

1st Signal/Mode	ECG/Trigger
Average cycle	787 ± 129 ms
Average cycle	No Signal ms
Captured cycle	787 ± 129 ms
Acquisition window	813 ms
Trigger pulse	1
Trigger delay	0 ms
TR	359.84 ms
Concatenations	1
Segments	72
Phases	1
Adaptive Triggering	Off

Physio - Cardiac

Tagging	None
Magn. preparation	Non-sel. IR T1map
TI	260 ms
Fat suppr.	None
Dark blood	Off
FoV read	360 mm
FoV phase	85.2 %
Phase resolution	66 %
Cine	Off
Trajectory	Cartesian
Dummy heartbeats	0
Motion Correction	Standard

Physio - PACE

Resp. control	Breath-hold
Concatenations	1

Sequence - Part 1

Introduction	Off
Dimension	2D
Reordering	Linear
Asymmetric echo	Weak
Contrasts	1
Optimization	Min. TE TR
Multi-slice mode	Sequential
Sequence type	Trufi
Bandwidth	1085 Hz/Px

Sequence - Part 2

Define	Shots
Shots per slice	1
Segments	72
Trufi delta freq.	0 Hz
RF pulse type	Fast
Gradient mode	Fast
Excitation	Slice-sel.
Flip angle mode	Constant
Cine	Off

Sequence - Assistant

Mode	Off
Allowed delay	0 s

\\USER\Cardiac Research Protocols\Rapi-STRESS T1\Rapi-STRESS T1 (V2)\ShMOLLI_192i_d11_nFilt (TD=0)

TA: 7.1 s PM: FIX Voxel size: 0.9×0.9×8.0 mmPAT: 2 Rel. SNR: 1.00 : tfi

Properties

Prio recon	Off
Load images to viewer	On
Inline movie	Off
Auto store images	On
Load images to stamp segments	Off
Load images to graphic segments	On
Auto open inline display	Off
Auto close inline display	Off
Start measurement without further preparation	On
Wait for user to start	Off
Start measurements	Single measurement

Routine

Slice group	1
Slices	1
Dist. factor	25 %
Position	Isocenter
Orientation	Transversal
Phase enc. dir.	A >> P
AutoAlign	---
Phase oversampling	0 %
FoV read	360 mm
FoV phase	75.0 %
Slice thickness	8.0 mm
TR	378.98 ms
TE	1.07 ms
Averages	1
Concatenations	1
Filter	Raw filter, Distortion Corr.(2D)
Coil elements	BO2;SP2,3

Contrast - Common

TR	378.98 ms
TE	1.07 ms
Magn. preparation	Non-sel. IR
TI	260 ms
Flip angle	35 deg
Fat suppr.	None
Wrap-up Magn.	None

Contrast - Dynamic

Averages	1
Averaging mode	Short term
Reconstruction	Magn./Phase
Measurements	1
Multiple series	Off

Resolution - Common

FoV read	360 mm
FoV phase	75.0 %
Slice thickness	8.0 mm
Base resolution	192
Phase resolution	100 %
Phase partial Fourier	6/8
Trajectory	Cartesian
Interpolation	On

Resolution - iPAT

PAT mode	GRAPPA
Accel. factor PE	2
Ref. lines PE	24
Reference scan mode	Integrated

Resolution - Filter Image

Image Filter	Off
Distortion Corr.	On
Mode	2D
Unfiltered images	Off
Prescan Normalize	Off
Normalize	Off
B1 filter	Off

Resolution - Filter Rawdata

Raw filter	On
Elliptical filter	Off
POCS	Off

Geometry - Common

Slice group	1
Slices	1
Dist. factor	25 %
Position	Isocenter
Orientation	Transversal
Phase enc. dir.	A >> P
FoV read	360 mm
FoV phase	75.0 %
Slice thickness	8.0 mm
TR	378.98 ms
Multi-slice mode	Sequential
Series	Interleaved
Concatenations	1

Geometry - AutoAlign

Slice group	1
Position	Isocenter
Orientation	Transversal
Phase enc. dir.	A >> P
AutoAlign	---
Initial Position	Isocenter
Phase	0.0 mm
Read	0.0 mm
Shift	0.0 mm
Initial Rotation	0.00 deg
Initial Orientation	Transversal

Geometry - Saturation

Fat suppr.	None
Wrap-up Magn.	None
Special sat.	None

Geometry - Navigator

System - Miscellaneous

Positioning mode	FIX
Table position	H
Table position	0 mm

System - Miscellaneous

MSMA	S - C - T
Sagittal	R >> L
Coronal	A >> P
Transversal	F >> H
Coil Combine Mode	Adaptive Combine
Save uncombined	Off
Matrix Optimization	Off
Coil Focus	Flat
AutoAlign	---
Coil Select Mode	Default

System - Adjustments

B0 Shim mode	Cardiac
Adjust with body coil	On
Confirm freq. adjustment	Off
Assume Dominant Fat	Off
Assume Silicone	Off
Adjustment Tolerance	Auto

System - Adjust Volume

! Position	Isocenter
! Orientation	Transversal
! Rotation	0.00 deg
! A >> P	150 mm
! R >> L	150 mm
! F >> H	150 mm
Reset	Off

System - Tx/Rx

Frequency 1H	63.683250 MHz
Correction factor	1
Gain	High
Img. Scale Cor.	1.000
Reset	Off
? Ref. amplitude 1H	0.000 V

Physio - Signal1

1st Signal/Mode	ECG/Trigger
Average cycle	787 ± 129 ms
Average cycle	No Signal ms
Captured cycle	787 ± 129 ms
Acquisition window	379 ms
Trigger pulse	1
Trigger delay	0 ms
TR	378.98 ms
Concatenations	1
Segments	84
Phases	1
Adaptive Triggering	Off

Physio - Cardiac

Tagging	None
Magn. preparation	Non-sel. IR
TI	260 ms
Fat suppr.	None
Dark blood	Off
FoV read	360 mm
FoV phase	75.0 %
Phase resolution	100 %
Cine	Off
Trajectory	Cartesian
Dummy heartbeats	0
Motion Correction	None

Physio - PACE

Resp. control	Off
Concatenations	1

Sequence - Part 1

Introduction	Off
Dimension	2D
Reordering	Linear
Asymmetric echo	Weak
Contrasts	1
Optimization	Min. TE TR
Multi-slice mode	Sequential
Sequence type	Trufi
Bandwidth	898 Hz/Px

Sequence - Part 2

Define	Shots
Shots per slice	1
Segments	84
Trufi delta freq.	0 Hz
RF pulse type	Fast
Gradient mode	Fast
Excitation	Slice-sel.
Flip angle mode	Constant
Cine	Off

Sequence - Assistant

Mode	Off
Allowed delay	0 s

\\USER\Cardiac Research Protocols\Rapi-STRESS T1\Rapi-STRESS T1 (V2)\T1Map_ShortT1(TD=0)highHR

TA: 8.7 s PM: FIX Voxel size: 1.9×1.9×8.0 mmPAT: 2 Rel. SNR: 1.00 : tfi

Properties

Prio recon	Off
Load images to viewer	On
Inline movie	Off
Auto store images	On
Load images to stamp segments	On
Load images to graphic segments	On
Auto open inline display	Off
Auto close inline display	Off
Start measurement without further preparation	On
Wait for user to start	Off
Start measurements	Single measurement

Routine

Slice group	1
Slices	1
Dist. factor	20 %
Position	Isocenter
Orientation	Transversal
Phase enc. dir.	A >> P
AutoAlign	---
Phase oversampling	0 %
FoV read	360 mm
FoV phase	85.4 %
Slice thickness	8.0 mm
TR	341.12 ms
TE	1.01 ms
Averages	1
Concatenations	1
Filter	Distortion Corr.(2D)
Coil elements	BO2;SP2,3

Contrast - Common

TR	341.12 ms
TE	1.01 ms
Magn. preparation	Non-sel. IR T1map
TI	260 ms
Flip angle	35 deg
Fat suppr.	None
Wrap-up Magn.	None

Contrast - Dynamic

Averages	1
Averaging mode	Short term
Reconstruction	Magnitude
Measurements	1
Multiple series	Off

Resolution - Common

FoV read	360 mm
FoV phase	85.4 %
Slice thickness	8.0 mm
Base resolution	192
Phase resolution	78 %
Phase partial Fourier	7/8
Trajectory	Cartesian
Interpolation	Off

Resolution - iPAT

PAT mode	GRAPPA
Accel. factor PE	2
Ref. lines PE	36
Reference scan mode	GRE/separate

Resolution - Filter Image

Image Filter	Off
Distortion Corr.	On
Mode	2D
Unfiltered images	Off
Prescan Normalize	Off
Normalize	Off
B1 filter	Off

Resolution - Filter Rawdata

Raw filter	Off
Elliptical filter	Off
POCS	Off

Geometry - Common

Slice group	1
Slices	1
Dist. factor	20 %
Position	Isocenter
Orientation	Transversal
Phase enc. dir.	A >> P
FoV read	360 mm
FoV phase	85.4 %
Slice thickness	8.0 mm
TR	341.12 ms
Multi-slice mode	Sequential
Series	Base To Apex
Concatenations	1

Geometry - AutoAlign

Slice group	1
Position	Isocenter
Orientation	Transversal
Phase enc. dir.	A >> P
AutoAlign	---
Initial Position	Isocenter
Phase	0.0 mm
Read	0.0 mm
Shift	0.0 mm
Initial Rotation	0.00 deg
Initial Orientation	Transversal

Geometry - Saturation

Fat suppr.	None
Wrap-up Magn.	None
Special sat.	None

Geometry - Navigator

System - Miscellaneous

Positioning mode	FIX
Table position	H
Table position	0 mm

System - Miscellaneous

MSMA	S - C - T
Sagittal	R >> L
Coronal	A >> P
Transversal	F >> H
Coil Combine Mode	Adaptive Combine
Save uncombined	Off
Matrix Optimization	Off
Coil Focus	Flat
AutoAlign	---
Coil Select Mode	Default

System - Adjustments

B0 Shim mode	Cardiac
Adjust with body coil	Off
Confirm freq. adjustment	Off
Assume Dominant Fat	Off
Assume Silicone	Off
Adjustment Tolerance	Auto

System - Adjust Volume

! Position	Isocenter
! Orientation	Transversal
! Rotation	0.00 deg
! A >> P	150 mm
! R >> L	150 mm
! F >> H	150 mm
Reset	Off

System - Tx/Rx

Frequency 1H	63.683250 MHz
Correction factor	1
Gain	High
Img. Scale Cor.	1.000
Reset	Off
? Ref. amplitude 1H	0.000 V

Physio - Signal1

1st Signal/Mode	ECG/Trigger
Average cycle	787 ± 129 ms
Average cycle	No Signal ms
Captured cycle	787 ± 129 ms
Acquisition window	704 ms
Trigger pulse	1
Trigger delay	0 ms
TR	341.12 ms
Concatenations	1
Segments	64
Phases	1
Adaptive Triggering	Off

Physio - Cardiac

Tagging	None
Magn. preparation	Non-sel. IR T1map
TI	260 ms
Fat suppr.	None
Dark blood	Off
FoV read	360 mm
FoV phase	85.4 %
Phase resolution	78 %
Cine	Off
Trajectory	Cartesian
Dummy heartbeats	0
Motion Correction	Standard

Physio - PACE

Resp. control	Breath-hold
Concatenations	1

Sequence - Part 1

Introduction	Off
Dimension	2D
Reordering	Linear
Asymmetric echo	Weak
Contrasts	1
Optimization	Min. TE TR
Multi-slice mode	Sequential
Sequence type	Trufi
Bandwidth	1085 Hz/Px

Sequence - Part 2

Define	Shots
Shots per slice	1
Segments	64
Trufi delta freq.	0 Hz
RF pulse type	Fast
Gradient mode	Fast
Excitation	Slice-sel.
Flip angle mode	Constant
Cine	Off

Sequence - Assistant

Mode	Off
Allowed delay	0 s

\\USER\Cardiac Research Protocols\Rapi-STRESS T1\Rapi-STRESS T1 (V2)\ShMOLLI_192i_d11_nFilt (TD=0)

TA: 7.1 s PM: FIX Voxel size: 0.9×0.9×8.0 mmPAT: 2 Rel. SNR: 1.00 : tfi

Properties

Prio recon	Off
Load images to viewer	On
Inline movie	Off
Auto store images	On
Load images to stamp segments	Off
Load images to graphic segments	On
Auto open inline display	Off
Auto close inline display	Off
Start measurement without further preparation	On
Wait for user to start	Off
Start measurements	Single measurement

Routine

Slice group	1
Slices	1
Dist. factor	25 %
Position	Isocenter
Orientation	Transversal
Phase enc. dir.	A >> P
AutoAlign	---
Phase oversampling	0 %
FoV read	360 mm
FoV phase	75.0 %
Slice thickness	8.0 mm
TR	378.98 ms
TE	1.07 ms
Averages	1
Concatenations	1
Filter	Raw filter, Distortion Corr.(2D)
Coil elements	BO2;SP2,3

Contrast - Common

TR	378.98 ms
TE	1.07 ms
Magn. preparation	Non-sel. IR
TI	260 ms
Flip angle	35 deg
Fat suppr.	None
Wrap-up Magn.	None

Contrast - Dynamic

Averages	1
Averaging mode	Short term
Reconstruction	Magn./Phase
Measurements	1
Multiple series	Off

Resolution - Common

FoV read	360 mm
FoV phase	75.0 %
Slice thickness	8.0 mm
Base resolution	192
Phase resolution	100 %
Phase partial Fourier	6/8
Trajectory	Cartesian
Interpolation	On

Resolution - iPAT

PAT mode	GRAPPA
Accel. factor PE	2
Ref. lines PE	24
Reference scan mode	Integrated

Resolution - Filter Image

Image Filter	Off
Distortion Corr.	On
Mode	2D
Unfiltered images	Off
Prescan Normalize	Off
Normalize	Off
B1 filter	Off

Resolution - Filter Rawdata

Raw filter	On
Elliptical filter	Off
POCS	Off

Geometry - Common

Slice group	1
Slices	1
Dist. factor	25 %
Position	Isocenter
Orientation	Transversal
Phase enc. dir.	A >> P
FoV read	360 mm
FoV phase	75.0 %
Slice thickness	8.0 mm
TR	378.98 ms
Multi-slice mode	Sequential
Series	Interleaved
Concatenations	1

Geometry - AutoAlign

Slice group	1
Position	Isocenter
Orientation	Transversal
Phase enc. dir.	A >> P
AutoAlign	---
Initial Position	Isocenter
Phase	0.0 mm
Read	0.0 mm
Shift	0.0 mm
Initial Rotation	0.00 deg
Initial Orientation	Transversal

Geometry - Saturation

Fat suppr.	None
Wrap-up Magn.	None
Special sat.	None

Geometry - Navigator

System - Miscellaneous

Positioning mode	FIX
Table position	H
Table position	0 mm

System - Miscellaneous

MSMA	S - C - T
Sagittal	R >> L
Coronal	A >> P
Transversal	F >> H
Coil Combine Mode	Adaptive Combine
Save uncombined	Off
Matrix Optimization	Off
Coil Focus	Flat
AutoAlign	---
Coil Select Mode	Default

System - Adjustments

B0 Shim mode	Cardiac
Adjust with body coil	On
Confirm freq. adjustment	Off
Assume Dominant Fat	Off
Assume Silicone	Off
Adjustment Tolerance	Auto

System - Adjust Volume

! Position	Isocenter
! Orientation	Transversal
! Rotation	0.00 deg
! A >> P	150 mm
! R >> L	150 mm
! F >> H	150 mm
Reset	Off

System - Tx/Rx

Frequency 1H	63.683250 MHz
Correction factor	1
Gain	High
Img. Scale Cor.	1.000
Reset	Off
? Ref. amplitude 1H	0.000 V

Physio - Signal1

1st Signal/Mode	ECG/Trigger
Average cycle	787 ± 129 ms
Average cycle	No Signal ms
Captured cycle	787 ± 129 ms
Acquisition window	379 ms
Trigger pulse	1
Trigger delay	0 ms
TR	378.98 ms
Concatenations	1
Segments	84
Phases	1
Adaptive Triggering	Off

Physio - Cardiac

Tagging	None
Magn. preparation	Non-sel. IR
TI	260 ms
Fat suppr.	None
Dark blood	Off
FoV read	360 mm
FoV phase	75.0 %
Phase resolution	100 %
Cine	Off
Trajectory	Cartesian
Dummy heartbeats	0
Motion Correction	None

Physio - PACE

Resp. control	Off
Concatenations	1

Sequence - Part 1

Introduction	Off
Dimension	2D
Reordering	Linear
Asymmetric echo	Weak
Contrasts	1
Optimization	Min. TE TR
Multi-slice mode	Sequential
Sequence type	Trufi
Bandwidth	898 Hz/Px

Sequence - Part 2

Define	Shots
Shots per slice	1
Segments	84
Trufi delta freq.	0 Hz
RF pulse type	Fast
Gradient mode	Fast
Excitation	Slice-sel.
Flip angle mode	Constant
Cine	Off

Sequence - Assistant

Mode	Off
Allowed delay	0 s

\\USER\Cardiac Research Protocols\Rapi-STRESS T1\Rapi-STRESS T1 (V2)\MidShMOLLI_192i_d11_nFilt (TD=0)

TA: 7.1 s PM: REF Voxel size: 0.9×0.9×8.0 mmPAT: 2 Rel. SNR: 1.00 : tfi

Properties

Prio recon	Off
Load images to viewer	On
Inline movie	Off
Auto store images	On
Load images to stamp segments	Off
Load images to graphic segments	On
Auto open inline display	Off
Auto close inline display	Off
Start measurement without further preparation	Off
Wait for user to start	Off
Start measurements	Single measurement

Routine

Slice group	1
Slices	1
Dist. factor	25 %
Position	Isocenter
Orientation	Transversal
Phase enc. dir.	A >> P
AutoAlign	---
Phase oversampling	0 %
FoV read	360 mm
FoV phase	75.0 %
Slice thickness	8.0 mm
TR	378.98 ms
TE	1.07 ms
Averages	1
Concatenations	1
Filter	Raw filter, Distortion Corr.(2D)
Coil elements	BO2;SP2,3

Contrast - Common

TR	378.98 ms
TE	1.07 ms
Magn. preparation	Non-sel. IR
TI	260 ms
Flip angle	35 deg
Fat suppr.	None
Wrap-up Magn.	None

Contrast - Dynamic

Averages	1
Averaging mode	Short term
Reconstruction	Magn./Phase
Measurements	1
Multiple series	Off

Resolution - Common

FoV read	360 mm
FoV phase	75.0 %
Slice thickness	8.0 mm
Base resolution	192
Phase resolution	100 %
Phase partial Fourier	6/8
Trajectory	Cartesian
Interpolation	On

Resolution - iPAT

PAT mode	GRAPPA
Accel. factor PE	2
Ref. lines PE	24
Reference scan mode	Integrated

Resolution - Filter Image

Image Filter	Off
Distortion Corr.	On
Mode	2D
Unfiltered images	Off
Prescan Normalize	Off
Normalize	Off
B1 filter	Off

Resolution - Filter Rawdata

Raw filter	On
Elliptical filter	Off
POCS	Off

Geometry - Common

Slice group	1
Slices	1
Dist. factor	25 %
Position	Isocenter
Orientation	Transversal
Phase enc. dir.	A >> P
FoV read	360 mm
FoV phase	75.0 %
Slice thickness	8.0 mm
TR	378.98 ms
Multi-slice mode	Sequential
Series	Interleaved
Concatenations	1

Geometry - AutoAlign

Slice group	1
Position	Isocenter
Orientation	Transversal
Phase enc. dir.	A >> P
AutoAlign	---
Initial Position	Isocenter
Phase	0.0 mm
Read	0.0 mm
Shift	0.0 mm
Initial Rotation	0.00 deg
Initial Orientation	Transversal

Geometry - Saturation

Fat suppr.	None
Wrap-up Magn.	None
Special sat.	None

Geometry - Navigator

System - Miscellaneous

Positioning mode	REF
Table position	H
Table position	0 mm

System - Miscellaneous

MSMA	S - C - T
Sagittal	R >> L
Coronal	A >> P
Transversal	F >> H
Coil Combine Mode	Adaptive Combine
Save uncombined	Off
Matrix Optimization	Off
Coil Focus	Flat
AutoAlign	---
Coil Select Mode	Default

System - Adjustments

B0 Shim mode	Cardiac
Adjust with body coil	On
Confirm freq. adjustment	Off
Assume Dominant Fat	Off
Assume Silicone	Off
Adjustment Tolerance	Auto

System - Adjust Volume

! Position	Isocenter
! Orientation	Transversal
! Rotation	0.00 deg
! A >> P	150 mm
! R >> L	150 mm
! F >> H	150 mm
Reset	Off

System - Tx/Rx

Frequency 1H	63.683250 MHz
Correction factor	1
Gain	High
Img. Scale Cor.	1.000
Reset	Off
? Ref. amplitude 1H	0.000 V

Physio - Signal1

1st Signal/Mode	ECG/Trigger
Average cycle	787 ± 129 ms
Average cycle	No Signal ms
Captured cycle	787 ± 129 ms
Acquisition window	379 ms
Trigger pulse	1
Trigger delay	0 ms
TR	378.98 ms
Concatenations	1
Segments	84
Phases	1
Adaptive Triggering	Off

Physio - Cardiac

Tagging	None
Magn. preparation	Non-sel. IR
TI	260 ms
Fat suppr.	None
Dark blood	Off
FoV read	360 mm
FoV phase	75.0 %
Phase resolution	100 %
Cine	Off
Trajectory	Cartesian
Dummy heartbeats	0
Motion Correction	None

Physio - PACE

Resp. control	Off
Concatenations	1

Sequence - Part 1

Introduction	Off
Dimension	2D
Reordering	Linear
Asymmetric echo	Weak
Contrasts	1
Optimization	Min. TE TR
Multi-slice mode	Sequential
Sequence type	Trufi
Bandwidth	898 Hz/Px

Sequence - Part 2

Define	Shots
Shots per slice	1
Segments	84
Trufi delta freq.	0 Hz
RF pulse type	Fast
Gradient mode	Fast
Excitation	Slice-sel.
Flip angle mode	Constant
Cine	Off

Sequence - Assistant

Mode	Off
Allowed delay	0 s

\\USER\Cardiac Research Protocols\Rapi-STRESS T1\Rapi-STRESS T1 (V2)\T1Map_ShortT1(TD=0)lowHR

TA: 0:17 PM: FIX Voxel size: 1.4×1.4×8.0 mmPAT: 2 Rel. SNR: 1.00 : tfi

Properties

Prio recon	Off
Load images to viewer	On
Inline movie	Off
Auto store images	On
Load images to stamp segments	On
Load images to graphic segments	On
Auto open inline display	Off
Auto close inline display	Off
Start measurement without further preparation	On
Wait for user to start	Off
Start measurements	Single measurement

Routine

Slice group	1
Slices	1
Dist. factor	20 %
Position	Isocenter
Orientation	Transversal
Phase enc. dir.	A >> P
AutoAlign	---
Phase oversampling	0 %
FoV read	360 mm
FoV phase	85.2 %
Slice thickness	8.0 mm
TR	359.84 ms
TE	1.13 ms
Averages	1
Concatenations	1
Filter	Distortion Corr.(2D)
Coil elements	BO2;SP2,3

Contrast - Common

TR	359.84 ms
TE	1.13 ms
Magn. preparation	Non-sel. IR T1map
TI	260 ms
Flip angle	35 deg
Fat suppr.	None
Wrap-up Magn.	None

Contrast - Dynamic

Averages	1
Averaging mode	Short term
Reconstruction	Magnitude
Measurements	1
Multiple series	Off

Resolution - Common

FoV read	360 mm
FoV phase	85.2 %
Slice thickness	8.0 mm
Base resolution	256
Phase resolution	66 %
Phase partial Fourier	7/8
Trajectory	Cartesian
Interpolation	Off

Resolution - iPAT

PAT mode	GRAPPA
Accel. factor PE	2
Ref. lines PE	36
Reference scan mode	GRE/separate

Resolution - Filter Image

Image Filter	Off
Distortion Corr.	On
Mode	2D
Unfiltered images	Off
Prescan Normalize	Off
Normalize	Off
B1 filter	Off

Resolution - Filter Rawdata

Raw filter	Off
Elliptical filter	Off
POCS	Off

Geometry - Common

Slice group	1
Slices	1
Dist. factor	20 %
Position	Isocenter
Orientation	Transversal
Phase enc. dir.	A >> P
FoV read	360 mm
FoV phase	85.2 %
Slice thickness	8.0 mm
TR	359.84 ms
Multi-slice mode	Sequential
Series	Base To Apex
Concatenations	1

Geometry - AutoAlign

Slice group	1
Position	Isocenter
Orientation	Transversal
Phase enc. dir.	A >> P
AutoAlign	---
Initial Position	Isocenter
Phase	0.0 mm
Read	0.0 mm
Shift	0.0 mm
Initial Rotation	0.00 deg
Initial Orientation	Transversal

Geometry - Saturation

Fat suppr.	None
Wrap-up Magn.	None
Special sat.	None

Geometry - Navigator

System - Miscellaneous

Positioning mode	FIX
Table position	H
Table position	0 mm

System - Miscellaneous

MSMA	S - C - T
Sagittal	R >> L
Coronal	A >> P
Transversal	F >> H
Coil Combine Mode	Adaptive Combine
Save uncombined	Off
Matrix Optimization	Off
Coil Focus	Flat
AutoAlign	---
Coil Select Mode	Default

System - Adjustments

B0 Shim mode	Cardiac
Adjust with body coil	Off
Confirm freq. adjustment	Off
Assume Dominant Fat	Off
Assume Silicone	Off
Adjustment Tolerance	Auto

System - Adjust Volume

! Position	Isocenter
! Orientation	Transversal
! Rotation	0.00 deg
! A >> P	150 mm
! R >> L	150 mm
! F >> H	150 mm
Reset	Off

System - Tx/Rx

Frequency 1H	63.683250 MHz
Correction factor	1
Gain	High
Img. Scale Cor.	1.000
Reset	Off
? Ref. amplitude 1H	0.000 V

Physio - Signal1

1st Signal/Mode	ECG/Trigger
Average cycle	787 ± 129 ms
Average cycle	No Signal ms
Captured cycle	787 ± 129 ms
Acquisition window	813 ms
Trigger pulse	1
Trigger delay	0 ms
TR	359.84 ms
Concatenations	1
Segments	72
Phases	1
Adaptive Triggering	Off

Physio - Cardiac

Tagging	None
Magn. preparation	Non-sel. IR T1map
TI	260 ms
Fat suppr.	None
Dark blood	Off
FoV read	360 mm
FoV phase	85.2 %
Phase resolution	66 %
Cine	Off
Trajectory	Cartesian
Dummy heartbeats	0
Motion Correction	Standard

Physio - PACE

Resp. control	Breath-hold
Concatenations	1

Sequence - Part 1

Introduction	Off
Dimension	2D
Reordering	Linear
Asymmetric echo	Weak
Contrasts	1
Optimization	Min. TE TR
Multi-slice mode	Sequential
Sequence type	Trufi
Bandwidth	1085 Hz/Px

Sequence - Part 2

Define	Shots
Shots per slice	1
Segments	72
Trufi delta freq.	0 Hz
RF pulse type	Fast
Gradient mode	Fast
Excitation	Slice-sel.
Flip angle mode	Constant
Cine	Off

Sequence - Assistant

Mode	Off
Allowed delay	0 s

\\USER\Cardiac Research Protocols\Rapi-STRESS T1\Rapi-STRESS T1 (V2)\ShMOLLI_192i_d11_nFilt (TD=0)

TA: 7.1 s PM: FIX Voxel size: 0.9×0.9×8.0 mmPAT: 2 Rel. SNR: 1.00 : tfi

Properties

Prio recon	Off
Load images to viewer	On
Inline movie	Off
Auto store images	On
Load images to stamp segments	Off
Load images to graphic segments	On
Auto open inline display	Off
Auto close inline display	Off
Start measurement without further preparation	On
Wait for user to start	Off
Start measurements	Single measurement

Routine

Slice group	1
Slices	1
Dist. factor	25 %
Position	Isocenter
Orientation	Transversal
Phase enc. dir.	A >> P
AutoAlign	---
Phase oversampling	0 %
FoV read	360 mm
FoV phase	75.0 %
Slice thickness	8.0 mm
TR	378.98 ms
TE	1.07 ms
Averages	1
Concatenations	1
Filter	Raw filter, Distortion Corr.(2D)
Coil elements	BO2;SP2,3

Contrast - Common

TR	378.98 ms
TE	1.07 ms
Magn. preparation	Non-sel. IR
TI	260 ms
Flip angle	35 deg
Fat suppr.	None
Wrap-up Magn.	None

Contrast - Dynamic

Averages	1
Averaging mode	Short term
Reconstruction	Magn./Phase
Measurements	1
Multiple series	Off

Resolution - Common

FoV read	360 mm
FoV phase	75.0 %
Slice thickness	8.0 mm
Base resolution	192
Phase resolution	100 %
Phase partial Fourier	6/8
Trajectory	Cartesian
Interpolation	On

Resolution - iPAT

PAT mode	GRAPPA
Accel. factor PE	2
Ref. lines PE	24
Reference scan mode	Integrated

Resolution - Filter Image

Image Filter	Off
Distortion Corr.	On
Mode	2D
Unfiltered images	Off
Prescan Normalize	Off
Normalize	Off
B1 filter	Off

Resolution - Filter Rawdata

Raw filter	On
Elliptical filter	Off
POCS	Off

Geometry - Common

Slice group	1
Slices	1
Dist. factor	25 %
Position	Isocenter
Orientation	Transversal
Phase enc. dir.	A >> P
FoV read	360 mm
FoV phase	75.0 %
Slice thickness	8.0 mm
TR	378.98 ms
Multi-slice mode	Sequential
Series	Interleaved
Concatenations	1

Geometry - AutoAlign

Slice group	1
Position	Isocenter
Orientation	Transversal
Phase enc. dir.	A >> P
AutoAlign	---
Initial Position	Isocenter
Phase	0.0 mm
Read	0.0 mm
Shift	0.0 mm
Initial Rotation	0.00 deg
Initial Orientation	Transversal

Geometry - Saturation

Fat suppr.	None
Wrap-up Magn.	None
Special sat.	None

Geometry - Navigator

System - Miscellaneous

Positioning mode	FIX
Table position	H
Table position	0 mm

System - Miscellaneous

MSMA	S - C - T
Sagittal	R >> L
Coronal	A >> P
Transversal	F >> H
Coil Combine Mode	Adaptive Combine
Save uncombined	Off
Matrix Optimization	Off
Coil Focus	Flat
AutoAlign	---
Coil Select Mode	Default

System - Adjustments

B0 Shim mode	Cardiac
Adjust with body coil	On
Confirm freq. adjustment	Off
Assume Dominant Fat	Off
Assume Silicone	Off
Adjustment Tolerance	Auto

System - Adjust Volume

! Position	Isocenter
! Orientation	Transversal
! Rotation	0.00 deg
! A >> P	150 mm
! R >> L	150 mm
! F >> H	150 mm
Reset	Off

System - Tx/Rx

Frequency 1H	63.683250 MHz
Correction factor	1
Gain	High
Img. Scale Cor.	1.000
Reset	Off
? Ref. amplitude 1H	0.000 V

Physio - Signal1

1st Signal/Mode	ECG/Trigger
Average cycle	787 ± 129 ms
Average cycle	No Signal ms
Captured cycle	787 ± 129 ms
Acquisition window	379 ms
Trigger pulse	1
Trigger delay	0 ms
TR	378.98 ms
Concatenations	1
Segments	84
Phases	1
Adaptive Triggering	Off

Physio - Cardiac

Tagging	None
Magn. preparation	Non-sel. IR
TI	260 ms
Fat suppr.	None
Dark blood	Off
FoV read	360 mm
FoV phase	75.0 %
Phase resolution	100 %
Cine	Off
Trajectory	Cartesian
Dummy heartbeats	0
Motion Correction	None

Physio - PACE

Resp. control	Off
Concatenations	1

Sequence - Part 1

Introduction	Off
Dimension	2D
Reordering	Linear
Asymmetric echo	Weak
Contrasts	1
Optimization	Min. TE TR
Multi-slice mode	Sequential
Sequence type	Trufi
Bandwidth	898 Hz/Px

Sequence - Part 2

Define	Shots
Shots per slice	1
Segments	84
Trufi delta freq.	0 Hz
RF pulse type	Fast
Gradient mode	Fast
Excitation	Slice-sel.
Flip angle mode	Constant
Cine	Off

Sequence - Assistant

Mode	Off
Allowed delay	0 s

\\USER\Cardiac Research Protocols\Rapi-STRESS T1\Rapi-STRESS T1 (V2)\T1Map_ShortT1(TD=0)highHR

TA: 8.7 s PM: FIX Voxel size: 1.9×1.9×8.0 mmPAT: 2 Rel. SNR: 1.00 : tfi

Properties

Prio recon	Off
Load images to viewer	On
Inline movie	Off
Auto store images	On
Load images to stamp segments	On
Load images to graphic segments	On
Auto open inline display	Off
Auto close inline display	Off
Start measurement without further preparation	On
Wait for user to start	Off
Start measurements	Single measurement

Routine

Slice group	1
Slices	1
Dist. factor	20 %
Position	Isocenter
Orientation	Transversal
Phase enc. dir.	A >> P
AutoAlign	---
Phase oversampling	0 %
FoV read	360 mm
FoV phase	85.4 %
Slice thickness	8.0 mm
TR	341.12 ms
TE	1.01 ms
Averages	1
Concatenations	1
Filter	Distortion Corr.(2D)
Coil elements	BO2;SP2,3

Contrast - Common

TR	341.12 ms
TE	1.01 ms
Magn. preparation	Non-sel. IR T1map
TI	260 ms
Flip angle	35 deg
Fat suppr.	None
Wrap-up Magn.	None

Contrast - Dynamic

Averages	1
Averaging mode	Short term
Reconstruction	Magnitude
Measurements	1
Multiple series	Off

Resolution - Common

FoV read	360 mm
FoV phase	85.4 %
Slice thickness	8.0 mm
Base resolution	192
Phase resolution	78 %
Phase partial Fourier	7/8
Trajectory	Cartesian
Interpolation	Off

Resolution - iPAT

PAT mode	GRAPPA
Accel. factor PE	2
Ref. lines PE	36
Reference scan mode	GRE/separate

Resolution - Filter Image

Image Filter	Off
Distortion Corr.	On
Mode	2D
Unfiltered images	Off
Prescan Normalize	Off
Normalize	Off
B1 filter	Off

Resolution - Filter Rawdata

Raw filter	Off
Elliptical filter	Off
POCS	Off

Geometry - Common

Slice group	1
Slices	1
Dist. factor	20 %
Position	Isocenter
Orientation	Transversal
Phase enc. dir.	A >> P
FoV read	360 mm
FoV phase	85.4 %
Slice thickness	8.0 mm
TR	341.12 ms
Multi-slice mode	Sequential
Series	Base To Apex
Concatenations	1

Geometry - AutoAlign

Slice group	1
Position	Isocenter
Orientation	Transversal
Phase enc. dir.	A >> P
AutoAlign	---
Initial Position	Isocenter
Phase	0.0 mm
Read	0.0 mm
Shift	0.0 mm
Initial Rotation	0.00 deg
Initial Orientation	Transversal

Geometry - Saturation

Fat suppr.	None
Wrap-up Magn.	None
Special sat.	None

Geometry - Navigator

System - Miscellaneous

Positioning mode	FIX
Table position	H
Table position	0 mm

System - Miscellaneous

MSMA	S - C - T
Sagittal	R >> L
Coronal	A >> P
Transversal	F >> H
Coil Combine Mode	Adaptive Combine
Save uncombined	Off
Matrix Optimization	Off
Coil Focus	Flat
AutoAlign	---
Coil Select Mode	Default

System - Adjustments

B0 Shim mode	Cardiac
Adjust with body coil	Off
Confirm freq. adjustment	Off
Assume Dominant Fat	Off
Assume Silicone	Off
Adjustment Tolerance	Auto

System - Adjust Volume

! Position	Isocenter
! Orientation	Transversal
! Rotation	0.00 deg
! A >> P	150 mm
! R >> L	150 mm
! F >> H	150 mm
Reset	Off

System - Tx/Rx

Frequency 1H	63.683250 MHz
Correction factor	1
Gain	High
Img. Scale Cor.	1.000
Reset	Off
? Ref. amplitude 1H	0.000 V

Physio - Signal1

1st Signal/Mode	ECG/Trigger
Average cycle	787 ± 129 ms
Average cycle	No Signal ms
Captured cycle	787 ± 129 ms
Acquisition window	704 ms
Trigger pulse	1
Trigger delay	0 ms
TR	341.12 ms
Concatenations	1
Segments	64
Phases	1
Adaptive Triggering	Off

Physio - Cardiac

Tagging	None
Magn. preparation	Non-sel. IR T1map
TI	260 ms
Fat suppr.	None
Dark blood	Off
FoV read	360 mm
FoV phase	85.4 %
Phase resolution	78 %
Cine	Off
Trajectory	Cartesian
Dummy heartbeats	0
Motion Correction	Standard

Physio - PACE

Resp. control	Breath-hold
Concatenations	1

Sequence - Part 1

Introduction	Off
Dimension	2D
Reordering	Linear
Asymmetric echo	Weak
Contrasts	1
Optimization	Min. TE TR
Multi-slice mode	Sequential
Sequence type	Trufi
Bandwidth	1085 Hz/Px

Sequence - Part 2

Define	Shots
Shots per slice	1
Segments	64
Trufi delta freq.	0 Hz
RF pulse type	Fast
Gradient mode	Fast
Excitation	Slice-sel.
Flip angle mode	Constant
Cine	Off

Sequence - Assistant

Mode	Off
Allowed delay	0 s

\\USER\Cardiac Research Protocols\Rapi-STRESS T1\Rapi-STRESS T1 (V2)\ShMOLLI_192i_d11_nFilt (TD=0)

TA: 7.1 s PM: FIX Voxel size: 0.9×0.9×8.0 mmPAT: 2 Rel. SNR: 1.00 : tfi

Properties

Prio recon	Off
Load images to viewer	On
Inline movie	Off
Auto store images	On
Load images to stamp segments	Off
Load images to graphic segments	On
Auto open inline display	Off
Auto close inline display	Off
Start measurement without further preparation	On
Wait for user to start	Off
Start measurements	Single measurement

Routine

Slice group	1
Slices	1
Dist. factor	25 %
Position	Isocenter
Orientation	Transversal
Phase enc. dir.	A >> P
AutoAlign	---
Phase oversampling	0 %
FoV read	360 mm
FoV phase	75.0 %
Slice thickness	8.0 mm
TR	378.98 ms
TE	1.07 ms
Averages	1
Concatenations	1
Filter	Raw filter, Distortion Corr.(2D)
Coil elements	BO2;SP2,3

Contrast - Common

TR	378.98 ms
TE	1.07 ms
Magn. preparation	Non-sel. IR
TI	260 ms
Flip angle	35 deg
Fat suppr.	None
Wrap-up Magn.	None

Contrast - Dynamic

Averages	1
Averaging mode	Short term
Reconstruction	Magn./Phase
Measurements	1
Multiple series	Off

Resolution - Common

FoV read	360 mm
FoV phase	75.0 %
Slice thickness	8.0 mm
Base resolution	192
Phase resolution	100 %
Phase partial Fourier	6/8
Trajectory	Cartesian
Interpolation	On

Resolution - iPAT

PAT mode	GRAPPA
Accel. factor PE	2
Ref. lines PE	24
Reference scan mode	Integrated

Resolution - Filter Image

Image Filter	Off
Distortion Corr.	On
Mode	2D
Unfiltered images	Off
Prescan Normalize	Off
Normalize	Off
B1 filter	Off

Resolution - Filter Rawdata

Raw filter	On
Elliptical filter	Off
POCS	Off

Geometry - Common

Slice group	1
Slices	1
Dist. factor	25 %
Position	Isocenter
Orientation	Transversal
Phase enc. dir.	A >> P
FoV read	360 mm
FoV phase	75.0 %
Slice thickness	8.0 mm
TR	378.98 ms
Multi-slice mode	Sequential
Series	Interleaved
Concatenations	1

Geometry - AutoAlign

Slice group	1
Position	Isocenter
Orientation	Transversal
Phase enc. dir.	A >> P
AutoAlign	---
Initial Position	Isocenter
Phase	0.0 mm
Read	0.0 mm
Shift	0.0 mm
Initial Rotation	0.00 deg
Initial Orientation	Transversal

Geometry - Saturation

Fat suppr.	None
Wrap-up Magn.	None
Special sat.	None

Geometry - Navigator

System - Miscellaneous

Positioning mode	FIX
Table position	H
Table position	0 mm

System - Miscellaneous

MSMA	S - C - T
Sagittal	R >> L
Coronal	A >> P
Transversal	F >> H
Coil Combine Mode	Adaptive Combine
Save uncombined	Off
Matrix Optimization	Off
Coil Focus	Flat
AutoAlign	---
Coil Select Mode	Default

System - Adjustments

B0 Shim mode	Cardiac
Adjust with body coil	On
Confirm freq. adjustment	Off
Assume Dominant Fat	Off
Assume Silicone	Off
Adjustment Tolerance	Auto

System - Adjust Volume

! Position	Isocenter
! Orientation	Transversal
! Rotation	0.00 deg
! A >> P	150 mm
! R >> L	150 mm
! F >> H	150 mm
Reset	Off

System - Tx/Rx

Frequency 1H	63.683250 MHz
Correction factor	1
Gain	High
Img. Scale Cor.	1.000
Reset	Off
? Ref. amplitude 1H	0.000 V

Physio - Signal1

1st Signal/Mode	ECG/Trigger
Average cycle	787 ± 129 ms
Average cycle	No Signal ms
Captured cycle	787 ± 129 ms
Acquisition window	379 ms
Trigger pulse	1
Trigger delay	0 ms
TR	378.98 ms
Concatenations	1
Segments	84
Phases	1
Adaptive Triggering	Off

Physio - Cardiac

Tagging	None
Magn. preparation	Non-sel. IR
TI	260 ms
Fat suppr.	None
Dark blood	Off
FoV read	360 mm
FoV phase	75.0 %
Phase resolution	100 %
Cine	Off
Trajectory	Cartesian
Dummy heartbeats	0
Motion Correction	None

Physio - PACE

Resp. control	Off
Concatenations	1

Sequence - Part 1

Introduction	Off
Dimension	2D
Reordering	Linear
Asymmetric echo	Weak
Contrasts	1
Optimization	Min. TE TR
Multi-slice mode	Sequential
Sequence type	Trufi
Bandwidth	898 Hz/Px

Sequence - Part 2

Define	Shots
Shots per slice	1
Segments	84
Trufi delta freq.	0 Hz
RF pulse type	Fast
Gradient mode	Fast
Excitation	Slice-sel.
Flip angle mode	Constant
Cine	Off

Sequence - Assistant

Mode	Off
Allowed delay	0 s
