# Supplementary material for: Cardiac stress T1-mapping response and extracellular volume stability of MOLLI-based T1-mapping methods
Source: Sci Rep. 2021 Jun 30;11:13568. doi: 10.1038/s41598-021-92923-4 (PMC8245629; doi:10.1038/s41598-021-92923-4)
Supplement: Supplementary file 2 — Supplementary Information 2. [file 41598_2021_92923_MOESM2_ESM.docx]

**Supplementary Material**


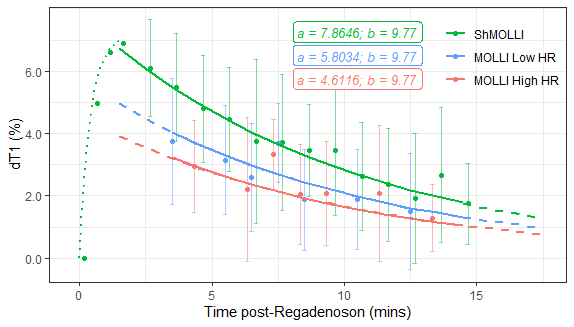


Figure S1 – Regadenoson stress effects on dT1 with overlaid mono-exponential decay models$y = a * exp(-x/b)$. The decay time b has been established for ShMOLLI only, and only amplitudes scaled to best fit other MOLLI variants; the dashed lines mark the extrapolation of the MOLLI models beyond the respective data changes. The rising part of the T1 response to stress (dotted green line) is to guide the eye only. This is similar to Figure 3C from the main manuscript, with error bars provided to show the data range for dT1 across subjects. Data points are now offset for the purpose of visualization to prevent overlap of error bars.


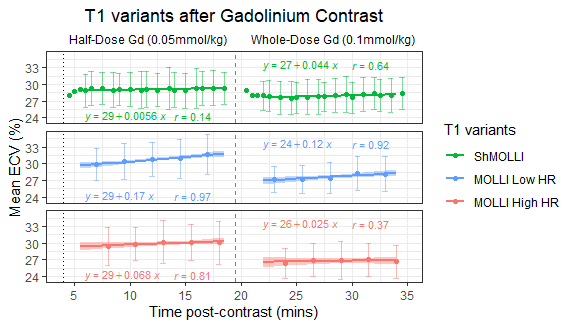


Figure S2 – Effect of time and gadolinium dose on ECV. Linear regressions are shown for half-dose and whole-dose Gd epochs. Stress was reversed at 4 minutes (dotted vertical line); Gd was given at 0 minutes and a second dose at 19 minutes (dashed vertical line). The early rapid variation in ECV estimates after Gd seen in the ShMOLLI panel (data points outside the ranges marked by regression models) were excluded from analysis to allow fair inter-method comparisons. ShMOLLI ECV demonstrates little change in ECV with time, whereas MOLLI ECV (particularly MOLLI Low HR) is subject to linear time drift. This is similar to Figure 5 from the main manuscript, with error bars provided to show the data range for ECV across all subjects. Note that the error bars represent mixed variability and inform more about pathophysiological variability than for the individual measurements.
